# Interventions for social and community participation for adults with intellectual disability, psychosocial disability or on the autism spectrum: An umbrella systematic review

**DOI:** 10.3389/fresc.2022.935473

**Published:** 2022-08-19

**Authors:** Melita J. Giummarra, Ivana Randjelovic, Lisa O’Brien

**Affiliations:** ^1^ Research and Evaluation Branch, Digital Design and Strategy Division, National Disability Insurance Agency, Melbourne Victoria, Australia; ^2^ Central Clinical School, Monash University, Melbourne, Victoria, Australia; ^3^ Department of Nursing and Allied Health, Swinburne University of Technology, Hawthorn, Victoria, Australia

**Keywords:** disability, community, participation, inclusion, belonging

## Abstract

**Objective:**

This umbrella systematic review examined the effectiveness, facilitators, and barriers of interventions for social, community and civic participation for adults on the autism spectrum, or with intellectual or psychosocial disability.

**Data Sources:**

Eight databases were searched to identify eligible reviews defined by the: Sample (≥50% adults on the autism spectrum or with intellectual or psychosocial disability), Phenomena of Interest (interventions in community settings that aimed to improve social, community or civic participation, or capacity to participate), Design (any), Evaluation (any method that evaluated impacts on participation or capacity to participate), and Research type (reviews as journal articles, dissertations or in grey literature, in English, published 2010-2020).

**Review Methods:**

Rapid review methods were used. One researcher screened 27,890 records and 788 potentially eligible full texts. A second reviewer independently screened 20% of records, and ambiguous full text publications. Study quality was extracted, and review quality was assessed with the Assessing Methodological Quality of Systematic Reviews (AMSTAR) checklist. Data from 522 studies in 57 eligible systematic reviews were extracted for narrative synthesis. The Corrected Covered Area (CCA) was calculated to indicate overlap between reviews.

**Results:**

There was a pooled sample of 28,154 study participants, predominantly from studies in North America, the UK and Europe. There was very low overlap between reviews (CCA = 0.3%). Reviews were predominantly low quality: 77.2% of reviews met <50% of AMSTAR criteria. Most studies were low (45.4%) or moderate (38.3%) quality. Three broad intervention categories improved participation, inclusion and belonging outcomes: (1) interventions to help people identify and connect with participation opportunities (e.g., person centred planning); (2) participation opportunities or activities (e.g., joining a community group, sports or outdoor activities, or arts-based activities); and (3) supports to build skills and capacity to participate socially and in the community.

**Conclusions:**

The evidence highlighted that improved social and community participation requires purposeful strategies that identify meaningful participation preferences (e.g., where, when, how, and with whom) and provide support to build capacity or enable ongoing participation. Community capacity building, peer support and advocacy may also be needed to make the community more accessible, and to enable people to exercise genuine choice.

## 1. Introduction

Social, Community and Civic participation have important benefits for people with disabilities, as well as for their family and carers, including improved wellbeing (1) and increased study, volunteering or paid employment opportunities (2). There are also likely to be broader social benefits including improved social capital and accessibility for all members of the community when social settings are more accessible and welcoming to everyone in society, including people with disabilities (3).

Social, Community and Civic participation is considered one of the core domains of the International Classification of Functioning (ICF), Disability, and Health framework (4), which recognizes the important relationships between disability, function, the environment, and health. While most of the specified domains in the ICF framework focus on individual activities or functions rather than participation, Chapter 7 (Interpersonal Interactions and Relationships) and Chapter 8 (Major Life Areas) outline key aspects of participation (5). For this review, we considered participation in line with the Convention on the Rights of Persons with Disabilities (CRPD) (6), defined as the rights to: full inclusion and participation of people with disability in the community (**Article 28**); effective and full participation in political and public life (**Article 29**); participation in mainstream and disability-specific sporting and recreational activities at all levels to the fullest extent possible (**Article 30**); and access to sporting, recreational and tourism venues or services for organizing recreational, tourism, leisure and sporting activities (**Article 30**). Participation and related outcomes were conceptualized as activities that: (a) are ideally chosen or desired by the individual with a disability; (b) occur in a social, community or civic setting; and (c) enable people with disabilities to participate alongside and/or with people without disabilities, or to build the skills, self-efficacy, or social networks to enable participation alongside/with people without disabilities (7).

In Australia, the most prevalent barriers to social and community participation are experienced by people living with Autism Spectrum Disorder, Intellectual and Psychosocial Disabilities (8). *Autism Spectrum Disorder* is a developmental condition that includes persistent deficits in social communication and interaction across multiple contexts; restricted, repetitive patterns of behavior, interests, or activities; and disturbances that cause clinically significant impairments (9). *Intellectual Disability* is defined as a disability that originates before the age of 18 with significant limitations in intellectual functioning, with an IQ < 70, and impairments in adaptive behavior related to many everyday social and practical skills (10). *Psychosocial disability* is a term used to describe disabilities arising from mental health conditions that are “multi-axial” comprising psychological, social, and occupational impacts of psychiatric, psychological, or developmental disorders (11). Co-occurrence of two or more of these disabilities is common. For instance, adults on the autism spectrum have higher rates of psychiatric comorbidity (12), and 50%–60% of people on the autism spectrum also have an intellectual disability (13).

Consistent with the social model of disability (14), difficulties with participation often arise due to both societal and environmental factors including availability and access to transport in the community, accessibility of information and buildings, and community perceptions, actions and attitudes. Moreover, individual factors play a role, including health, mobility, poverty, communication, support from family/carers or friends, confidence, life experience, and interests (15). Finally, participation can be obstructed by the systemic exclusion of people with disabilities, availability of supports for disability needs, and lack of support for, or access to, education or employment (15).

To enhance social, community and civic participation for people on the autism spectrum, or with intellectual or psychosocial disability, we must identify and enable access to interventions that overcome the social, individual, and systemic barriers to participation. Such interventions may work by improving the fit between the person and their physical, social, or institutional environments (e.g., by making the environment more accessible to enable their participation), or by building the capacity of the individual with a disability to participate. Therefore, this review sought to systematically identify and synthesize the available evidence for the effectiveness of interventions or supports that aim to improve social, community and civic participation of adults on the autism spectrum, or who have intellectual or psychosocial disabilities. Broad inclusion criteria were defined; however, in accordance with the registered protocol, the study was conducted as an umbrella review given that many systematic reviews were identified. The review aimed to answer three overarching research questions:

1. What interventions are effective for who, how, under what conditions, for which activities, and for what outcomes?
2. Where the evidence is sufficiently strong and consistent for implementation: (a) what is the acceptability of the interventions; (b) what are the barriers and facilitators of intervention implementation; (c) what resources are required for implementation; and (d) is there evidence of cost-effectiveness?
3. What are the gaps in evidence?

## 2. Method

The protocol for the review was registered to PROSPERO on 6th January 2020 (CRD42021229580). Minor protocol deviations are outlined in **Supplementary File 1**.

### 2.1. Eligibility criteria

As this review sought to synthesise evidence from studies that used a broad range of methods, we used the Sample, Phenomena of Interest, Design, Evaluation, Research type (SPIDER) framework (16). Detailed eligibility criteria are provided in Supplementary File 1. Publications were eligible for inclusion if they met the criteria outlined in Table 1, and if they were published between 2010 and 2020. This timeframe coincides with the increased use of systematic review methods and allows for the identification of contemporary empirical evidence as well as older studies published since deinstitutionalization and the independent living movement.

**Table 1:** SPIDER eligibility criteria for the umbrella review.

Domain	Inclusion criteria	Exclusion Criteria
Sample	• ≥50% of participants aged 18 + years on autism spectrum, with intellectual or psychosocial disability • Living in the community, including small group homes/supported living	• People living in large group homes • People with acquired intellectual or cognitive disabilities • ≥50% of participants were secondary school students.
Phenomena of Interest	• Interventions, supports or programs in a community setting or that aimed to influence social, community or civic participation or capacity to participate.	• Interventions with a medical basis or focused on management of symptoms for delivery in the health system, consistent with previous reviews (17). • Reviews of Cognitive Remediation interventions as a separate evidence snapshot specific to this topic was already underway. • Reviews of employment or residential interventions that did not include an active social or community participation component as separate reviews were already commissioned to evaluate that evidence. • Interventions targeting the environment or community, which were beyond the scope of this review.
Design	• Systematic, scoping, rapid reviews of quantitative or qualitative research • Studies measured ≥1 capacity or participation outcome or intervention acceptability/feasibility	• Primary research studies not included in the systematic and scoping reviews.
Evaluation	• Quantitative and case study designs must evaluate a within or between group change in participation or capacity • Qualitative studies explored participation experiences, acceptability, barriers and facilitators or outcomes	
Research type	• Published in English from 2010 to 2020 • Journal articles, dissertations/theses, and grey literature	• Books, book chapters, editorials, letters, conference abstracts, organizational website content, or publicity materials from disability services due to potential conflicts of interest.

Publications could use umbrella, systematic, scoping, or rapid review methods, if they (a) included a clear statement of the purpose of the review; (b) described the search strategy, searched two or more databases, described the search terms used and the inclusion/exclusion criteria; (c) presented data on search and screening results, and presented all findings relevant to the main purpose of the review.

*Primary outcomes* were aspects of social participation (e.g., communication, social relationship maintenance, participation through telecommunications or online platforms, convivial encounters); social networking (e.g., friendships, relationships, networks); navigating or accessing the community (e.g., access or skills to use public or private transport); participation in recreation, sports and leisure activities in the community (e.g., sports, art, music, community or cultural events, libraries, tourism); or civic involvement (e.g., voting, volunteer work, advocacy, committee or club memberships, or political engagement). Studies that only measured housing or employment outcomes, or leisure activity participation with no social and community participation potential were not eligible. *Secondary outcomes* included aspects of psychosocial functioning (e.g., self-determination, autonomy, choice, decision-making, self-advocacy), physical or mental health, or quality of life.

### 2.2. Search strategy: Databases and search terms

**Figure 1:**
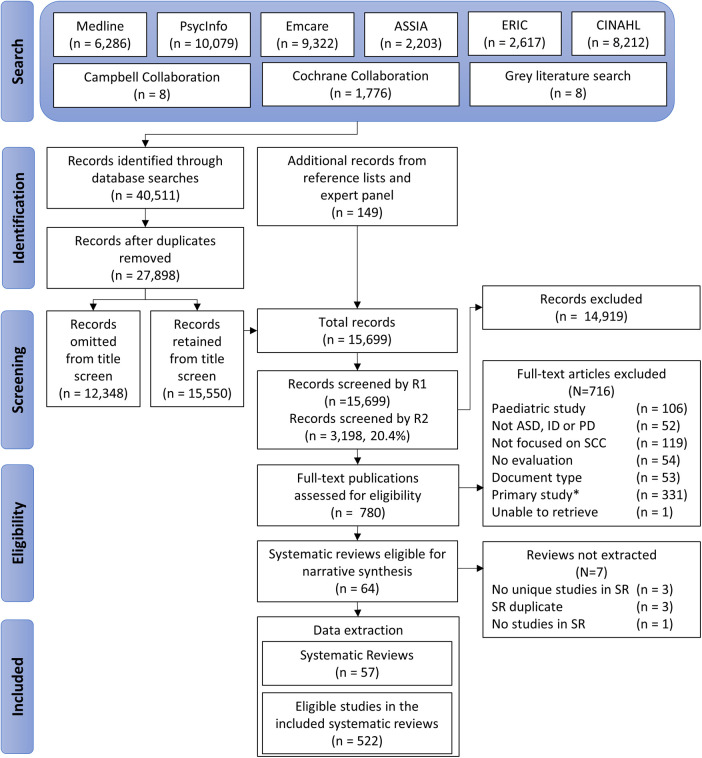
PRISMA chart. * These studies met the SPIDER study design eligibility criteria but did not meet the criteria for inclusion in an umbrella review.]

Medical Sub-Heading (MeSH) and keyword search terms for autism spectrum disorder, intellectual disability, and psychosocial disability and social, community and civic participation were adapted for eight search engines (Figure 1; the search terms are available in **Supplementary File 2**). Grey literature was identified using the DuckDuckGo search engine, which does not track search terms, and reduces the chance that reviewers are presented with biased website results based on previously viewed sites. Web screening was limited to the top 50 results for each disability population. Reference lists of included publications were hand searched to identify additional reviews, and an expert panel was consulted to identify any missed literature.

### 2.3. Study selection

Screening was conducted using Endnote, Covidence and Abstrackr. Abstrackr is a web-based platform that uses an active machine learning algorithm of reviewer judgements to predict the relevance of remaining citations, which are then sorted by predicted relevance to enable rapid identification of relevant records (18, 19). Search results were first consolidated in an Endnote library, and duplicates were removed. Due to the breadth of the review results once duplicates were removed, clearly ineligible citations were omitted when reviewing citation title, based on studies with ineligible disorders, paediatric populations, questionnaire validation methods, biomarker and neurophysiology studies, and document type, consistent with previous large-scale reviews (20). Study selection was undertaken in accordance with Cochrane Rapid Review methods (21) as follows. Reviewer 1 screened all citations in Endnote, and all full text articles in Covidence. Reviewer 2 screened in Abstrackr for the first 20% of citations predicted to be relevant, or until no further citations had > 50% relevance, whichever threshold was reached first. Systematic Review authors were contacted for additional information to determine eligibility of full texts, where necessary. If the reviewers were unsure about full text eligibility, a final decision was made in consultation with a third reviewer.

### 2.4. Data extraction

Data were extracted into excel spreadsheets (**Supplementary Data file 1** and **2**). Consistent with rapid review methods data extraction was completed by a single reviewer, and extraction accuracy and completeness were discussed between authors. Original study papers were accessed, or authors contacted, if key details were not provided in the systematic review.

Data extraction was conducted in two phases. In phase one summary information on the systematic reviews was extracted including: the review aim, design, key theoretical frameworks, and review inclusion and exclusion criteria; study selection and the number of studies that met our SPIDER criteria; pooled sample characteristics in each review for eligible studies (number, age, sex, disability types, countries); summary of interventions; design of eligible studies; type of control groups; and overall quality or risk of bias of the included studies. Overall effects on participation capacity, participation, quality of life, and secondary outcomes were recorded, and summarised as positive effects if ≥60% of studies had positive effect, negative effects if ≥60% of studies had negative effect, null effects if ≥60% of studies had null effect or inconsistent effects if no effect direction met the threshold for positive, negative or null effects. For meta-analyses, the inclusion of sensitivity analyses and identified biases were recorded. Study heterogeneity and whether review authors disclosed funding sources and conflicts of interest were recorded.

In phase two, information about the individual studies included in the systematic reviews was extracted, including: first author, publication year and country; study recruitment strategy and sample demographics (e.g., sample size, age, sex, disability or diagnoses); study design (e.g., descriptive or cross-sectional, mixed or multi-methods, multiple baseline case study, randomised controlled trial (RCT) or quasi RCT, non-randomised controlled trial with (NRCT-CG) or without a control group (NRCT-NoCG), qualitative, or review); intervention and control conditions (e.g., design, mode of delivery, the agent providing the intervention, the services provided, the duration and frequency of sessions in the intervention); and whether the intervention was in a disability-specific or mainstream setting. Effects of each intervention were extracted for social participation, capacity, and “other” outcomes, with complete data on the measures used and any effects of the intervention (available in Supplementary Data File 2). Data regarding cost-effectiveness and barriers or facilitators were documented where possible.

### 2.5. Quality assessments

Study quality, certainty, or risk of bias were extracted from included reviews where possible. Review quality was assessed using the 16 quality criteria in the checklist for Assessing the Methodological Quality of Systematic Reviews Version 2 (AMSTAR; 22). The proportion of relevant AMSTAR criteria that were met was calculated to summarise overall review quality. Review and study quality were generally classified as low, moderate, or high according to the original study classification, or based on tertiles of AMSTAR summary scores, respectively (e.g., studies that met <33.3% of quality criteria were considered low quality, but those that met >66.7% of quality criteria were considered high quality).

### 2.6. Data synthesis

The findings across systematic reviews are presented in a narrative synthesis of the characteristics of the included reviews, interventions, outcomes and effects on outcomes, and evidence quality (Research Question 1). To determine whether there was sufficiently strong and consistent evidence to support each intervention type we considered the consistency in the effectiveness of studies for the respective intervention category relative to the quality of the studies (Research Question 2).

As multiple systematic reviews may have included data from the same original studies, the Corrected Covered Area (CCA) was calculated across all studies, and studies on similar broad topic areas (e.g., social skills training) to provide insight into the level of overlap of original publications (23). The level of overlap was considered slight (<5%), moderate (6%–10%), high (11%–15%) or very high (>15%; 23). The following formula was used to calculate the CCA:

$$\begin{array}{c} CCA=100\;\times \frac{\left(N-R\right)}{\left(\left(R\;\times C\right)-R\right)}\\ \text{N = number of publications including double counting; R = number of index publications; C = number of reviews} \end{array}$$

## 3. Results

### 3.1. Study selection

Study selection is summarised in Figure 1. A total of 40,644 records were identified from the search that was executed on 22 December 2020, including 149 records from reference lists and expert guidance. Data were extracted from 57 reviews that did not completely overlap with other included reviews (Supplementary Table 1). These reviews included a total of 1,170 original studies, of which 522 met the SPIDER inclusion criteria. Most included publications were systematic reviews (40 reviews), followed by meta-ethnographies (6 reviews), meta-analyses (5 reviews), Cochrane reviews (2 reviews), scoping reviews (2 reviews), and umbrella reviews (2 reviews).

The studies included in each review included various research designs, including: RCT or quasi RCT (167 studies); qualitative (112 studies), mixed or multi-methods (43 studies); non-randomised controlled trials with (42 studies) or without a control group (64 studies); cross-sectional studies (13 studies); and descriptive (17 studies) or multiple baseline case studies (35 studies). Sixteen of the 522 studies were systematic reviews from the two umbrella reviews (24, 25). Study design was not clear for 13 studies.

### 3.2. Study characteristics

#### 3.2.1. Corrected covered area (CCA)

The overall CCA was 0.29%. Studies in four intervention categories had no overlap (i.e., travel and navigation training, art interventions for psychosocial disability and intellectual disability, parenting role training and vocation focused interventions). The remaining interventions had slight overlap (CCA median = 1.8%). Three topics that had high or very high overlap were social skills training for people on the autism spectrum (CCA = 10.2%; 17/44 studies included in 2–5 reviews), transition programs for people with intellectual disability or on the autism spectrum (CCA = 16.7%, 2/3 studies included in two reviews), and animal interventions for people with psychosocial disability (CCA = 33.3%, 2/6 studies included in two reviews).

#### 3.2.2. Intervention settings and outcomes

The 522 eligible studies included a pooled sample of 28,154 people with disability. Interventions focused on psychosocial disability (31 reviews, 311 studies), intellectual disability (23 reviews, 139 studies), or the autism spectrum (15 reviews, 85 studies). Eleven reviews (13 studies) included people with more than one disability type.

Thirty-nine (68.4%) reviews reported the country of 346 studies. The most common global regions were North America (31 reviews; 153 studies), the United Kingdom and Ireland (26 reviews; 96 studies), Europe (22 reviews; 38 studies), Australia (18 reviews; 37 studies), Asia (10 reviews; 7 studies), Middle East (7 reviews; 11 studies), South America (1 review; 1 study) and New Zealand (1 review; 1 study).

The most common social and community functioning outcomes were: loneliness (22 studies), isolation (11 studies) or inclusion (13 studies); social functioning (17 studies), social disability (3 studies), social acceptance (3 studies), socialisation (6 studies); social networks, including network size (17 studies) or composition (4 studies), interpersonal, social or peer relations (33 studies) friendships (22 studies), contact with friends (6 studies) or other social interactions (15 studies), and social support (21 studies). Assessment of actual participation in the community was less common, but included social (7 studies), leisure (5 studies) or community activity participation (3 studies); community involvement or participation (8 studies), access to community venues (4 studies), and confidence to be in the community (3 studies).

The most common capacity-focused outcomes were theory of mind (i.e., the ability to recognize and understand the mental states of others; 22 studies), affect recognition (20 studies), attribution style (9 studies), and empathy (eight studies); social (15 studies) and communication skills (six studies); and dating knowledge or sex-related behaviours (18 studies), and social knowledge (eight studies).

The most common “other” outcomes were: psychiatric (50 studies), depression (31 studies) or anxiety symptoms (9 studies); quality of life (43 studies) or general wellbeing (9 studies); mental health (10 studies) or emotional wellbeing (7 studies); physical health (5 studies); self-esteem (20 studies), confidence (17 studies); self-value (11 studies); self-efficacy (10 studies); self-determination (8 studies); empowerment (8 studies); choice (4 studies); challenging behaviours (13 studies); adaptive behaviour (4 studies); cognitive functioning (6 studies); employment (5 studies); and fitness (4 studies), sporting skill (4 studies) or other health-related outcomes (4 studies).

No studies examined cost-effectiveness.

#### 3.2.3. Quality of the evidence

Study quality is summarised in Table 2–4, and Supplementary Table 2. Forty-four reviews (77.2%) met <50% of AMSTAR quality criteria (median proportion of criteria met = 0.41; Q1 = 0.19, Q3 = 0.46). Nineteen reviews (33.3%) met <25% of quality domains, 25 (43.9%) met 25%–49% of domains, seven (12.3%) met 50–74% of domains and four met 75%–100% of quality domains. Quality domains that were most often not described or that were low quality were: lacking a-priori protocol (51 reviews, 89.5%); poor or lacking explanation of study selection procedures (31 reviews, 54.4%); single author screening records (31 reviews, 54.4%) or extracting data (44 reviews, 77.2%); no report of the full text records excluded (50 reviews, 87.7%); no report of the study funders (55 reviews, 96.5%); no risk of bias assessment or failure to account for risk of bias in the synthesis (45 reviews, 78.9%); and no examination or discussion of heterogeneity (32 reviews, 57.9%).

**Table 2:** Overall effects of intervention processes and supports to help people connect with social, community or civic participation opportunities, including AMSTAR quality rating of the SRs and quality of the original studies.

Intervention • *No. of studies* • A* for each SR reporting studies	Disability cohort, pooled sample size, age range, sex	Typical intervention duration and/or frequency	Effects on outcomes [study quality]		
			Social functioning	Social Skills	Other outcomes
1. Person Centred Planning • 10 studies • A*: L L L M	ID, *N* = 640, 13–85 years, sex nr	≥1 individual or group meeting, no set frequency	+** [L-H]		+** [L-H]
2. Individualised behaviour and participation support • 5 studies • A*: L M	ID, *N* = 65, 14–39 years, sex nr	As needed to generate & review plans	+ [nr]	o/+ [nr]	+ [nr]
3. Choice-making and “asset-based” approaches • 1* study • A*: L L	ID, *N* = 2*, 69 years, sex nr	Nr	+ [M]	o/+ [M]	+ [M]
4. Animal companionship with dog walking or in home • 4 study • A*: L L H	ID, *N* = 106, aged 18–64 years, sex nr	14 × 1-hr walking sessions Continuous or 50 min to 3-hrs p/wk	o/+ [L-H]		+ [M]
5. Community group participation linkage supports • 4 studies • A*: L L M M	ID or PSD, *N* = 13, *N* nr for 2 studies, age, sex	30-min staff introduction or 30 h of meetings with a recreational therapist over 9-10 weeks	+ [M]		
6. Social prescriber and “connecting people” interventions • 20 studies • A*: L L M M	PSD, *N* = 357, sample was nr for 16 studies, age, and sex nr	≥1 assessment plus 1–5 additional contacts, over 3–18 months	+ [L-H]	+ [nr]	+** L-H]
7. Befriending interventions with a non-disabled volunteer • 2 studies (ID), 5 studies (PSD) • A*: M M (ID), L M M (PSD)	ID, *N* = 38, age, and sex nr	nr	-/o/+ [L]		
	PSD, *N* = 637, age and sex nr	2 h/wk for 6wks to 12 months	o/+ [M-H]		o/+ [M-H]
8. Peer-based friendship programs • 5 studies • A*: L L M	PSD, *N* = 489 with a range of mental health diagnoses, age nr, 60% male	35–38 × 3-hr sessions (nr for most studies)	o/+ [L-M]		o/+ [L-M]
9. Peer support in the community • 9 studies (PSD), 1 study (ASD), 1 study (ID) • A*: L L M M M M H H (PSD), M (ASD), M (ID)	PSD, *N* = 1,337, age and sex nr	1.5 to 2-hr sessions for 4-weeks up to 12-months	o/+ [M-H]		o/+ [M-H]
	ASD, *N* = 35, 24–77 years, 69% male		+ [H]		
	ID, *N* = 10, 19–48 years & 30% male		+ [M]		
10. Peer support in mental health services • 36 studies • A*: L M H	PSD, *N* = 340 PSD mentors, *N* = 2,152 PSD mentees, *N* = 138 staff, age, and sex nr	Typically 2.5 h per week	o/+ [L-H]		o/+ [L-H]
11. Transition to young adulthood • 13 studies • A*: L L M M	ID or ASD, *N* = 210, aged 17–33 years, sex nr	Camp Campus for 1 wk or 10-month program (frequency & length nr)	+ [L-H]		
12. Transition to retirement • 2 studies • A*: L M	ID, *N* = 17, aged 48–62 years	weekly for 5–10 months (session length nr)	+** [M]		o/+ M]

Notes: Blank cells indicate no evidence available. Detailed summaries of the study and sample characteristics and outcomes are provided in **Supplementary Table 4**, the numbered intervention categories in column one corresponds to the numbered paragraphs below and the numbered rows in **Supplementary Table 4**.

Abbreviations: L, low quality; M, moderate quality; H, high quality; nr, not reported; A*, AMSTAR (A MeaSurement Tool to Assess systematic Review); ASD, autism spectrum disorder; ID, Intellectual Disability; PSD, Psychosocial Disability; SR, systematic review; UR, Umbrella Review, h, hour; hrs, hours; min, minutes; nr, not reported; N, number; wk, week.

Symbols:+positive effect (green); o null effect (red); - harmful effect (red); / indicates mixed effects (amber); * sample size does not include the participants included in SRs within URs; ** effects in most studies were positive, but some studies showed no effect.

**Table 3:** Overall effects of taking up opportunities for participation on participation and other outcomes, including AMSTAR quality rating of the SRs and quality of the original studies.

Intervention • *No. of studies* • A* for each SR reporting studies	Disability cohort, pooled sample size, age range, sex	Typical intervention duration and/or frequency	Effects on outcomes [study quality]		
			Social functioning	Social skills	Other outcomes
Participation in existing groups, activities or programs in the community					
13. Community group participation • 4 studies* • A*: L L M M	ID or PSD, *N* = 58, mean age 56–59 years, 72%–100% male	1-3 times per week for an average of 3.6 h per week	+ [L]		
14. Music • 1 study (ASD), 30 studies (PD) • A*: M (ASD), M H (PSD)	ASD, *N* = 22, age, and sex nr	nr	+ [nr]		
	PSD, *N* = 1819, 15–60 years, 56.5% male	1 to 6 sessions for a total of 45 min to 2 h p/wk	+** [L-M]		+** [L-M]
15. Dance • 6 studies • A*: L L M M M	ASD, *N* = 233 (ASD) including 151 with ID and 3 with PSD, 14–53 years, 72% male	weekly for 1 to 1.5-hrs p/wk for 7-10 wks		o/+ [M-H]	o/+ [M-H]
16. Drama • 13 studies • A*: M M	ID or PSD, *N* = 31 (ID), *N* = 171 (PSD), age and sex nr, but 4 programs for men only	Most 10–11 sessions or over 4–6 months (session length & frequency nr).	+ [nr]	+ [nr]	+ [nr]
17. Art • 1 study (ID), 8 studies* (PSD) • A*: M (ID), L L M (PSD)	ID, *N* = 5, aged 21–27 years	Two days p/wk	+ [L]		+ [L]
	PSD, *N* = 60, age, sex, and quality nr	2-hr p/wk to unlimited access to an open studio	+ [nr]		+ [nr]
18. Farm, ecotherapy, gardening and horticulture interventions • 14 studies • A*: L L L L H H	PSD, *N* = 405, aged 20s to 70s, 22% male	1 to 3 sessions of 1.5–3 h p/wk 2 × to 3-hr sessions p/wk for 12 wks	+ [M]		+** [M]
19. Outdoor nature experiences and camps • 7 studies • A*: M H H	PSD, *N* = 211, 18–65 years, 20%–39% male	1-5 weekly 1-3 h sessions	+ [M-H]		+ [M-H]
Sport or physical activity interventions					
20. Motivations to participate in sport or physical activity • 37 studies • A*: M M M	PSD, *N* = 6,466 psychosocial disability, N = 80 clinicians, aged 19-67 years, 71% male	1–2 weekly sessions of 45 min to 2-hrs	o/+ [M]		o/+ [L-H]
21. Sport or physical activity programs • 11 studies • A*: M	PSD, *N* = 552, mean age 25-45 years, sex nr	2-3 × 1–2 h p/wk for 8-24 wks	o/+ [M]		o/+ [L-H]
22. Mainstream sport/ physical activity in community • 5 studies • A*: L L M M	ID, *N* = 356, aged 11–83 years, sex nr	Nr	o/+ [L-H]		+** [L-H]
23. Unified Special Olympics participation • 6 studies • A*: L M M	ID, *N* not clear, average age 25–31 years, sex nr	Nr	+ [L]		+ [L-M]
24. Disability-specific physical activity programs • 12 studies • A*: L L L M M	ID or ASD, *N* = 448 (ID) & *N* = 89 (ASD), 13–77 years, 56.3% male	2-3 × wk for total of 1.5-hrs to 3-hrs p/day for 8 wks to 10 months	+** [L-H]		+** [L-H]
25. Disability-specific Special Olympics participation • 7 studies • A*: M M M	ID, *N* = 181 intellectual disability, *N* = 101 parents, siblings coaches or caregivers; aged 12–50 years, 52% male	3 × 1.5 h p/wk	+ [L]		+ [L]
26. Special Olympics participation (setting nr) • 10 studies • A*: L M	ID, *N* = 1,247 intellectual disability, *N* = 746 other people, aged 9–-69 years, 50% male	Nr	+ [L]		+ [L]

Notes: Blank cells indicate no evidence available. Detailed summaries of the study and sample characteristics and outcomes are provided in **Supplementary Table 4**, the numbered intervention categories in column one corresponds to the numbered paragraphs below and the numbered rows in **Supplementary Table 4**.

Abbreviations: L, low quality; M, moderate quality; H, high quality; A*, AMSTAR (A MeaSurement Tool to Assess systematic Review); AAT, Animal Assisted Therapy; ASD, autism spectrum disorders; ID, Intellectual Disability; PSD, Psychosocial Disability; SR, systematic review; UR, Umbrella Review, h, hour; hrs, hours; min, minutes; nr, not reported; N, number; wk, week.

Symbols:+positive effect (green) o null effect (red) - harmful effect (red) / indicates mixed effects (amber); * sample size does not include the participants included in SRs within URs; ** effects in most studies were positive, but some studies showed no effect; *** study examined acceptability and experiences only, not effects on participation or skills.

**Table 4:** Overall effects of interventions to build skills, psychosocial wellbeing, and broader capacity to participate socially and in the community, including AMSTAR quality rating of the SRs and quality of the original studies.

Intervention • *No. of studies* • A* for each SR reporting studies	Disability cohort, pooled sample size, age range, sex	Typical intervention duration and/or frequency	Effects on outcomes [study quality]		
			Social functioning	Social Skills	Other outcomes
Social skill and communication interventions					
27. Social Skills Training that did not include a “cognitive” focus • 14* studies • A*: L L L M M M	PSD, *N* = 549, 17–51 years, 76% male	45 min to 1.75 h/wk for up to 18 months	+** [L]	+ [L]	o/+ [L]
28. Social Cognition training focused on loneliness & self-control • 3 studies • A*: L M	PSD, *N* = 269, mean age 20–50 years, 64% male	30–60 min/wk for 9-12 wks	+ [M]	o/+ [M]	
29. Individual or group Social Cognition and Interaction Training (SCIT) • 13 studies • A*: L M M M	PSD, *N* = 719, M = 33–51 years, 68% male	1–2 × 1.5 h p/wk for 16–24 wks	+ [L-M]	o/+ [M]	
30. Group social skills training • 4 studies (ID), 7 studies (ASD) • A*: L L M M (ID), L L L M M (ASD)	ID, *N* = 71, 17–48 years, 38% male	2 h/wk for 12–14 wk s	+ [M]	+ [M]	
	ASD, *N* = 78, 16–55 years, 85% male	30 min to 3-hrs sessions over 4–6 wks or up to 18-wks	o [nr]	o/+ [L]	
31. PEERS-YA social skills training program • 4 studies • A*: L L M M	ASD, *N* = 97, 20–24 years, 80% male	1.5 h sessions over 14–16 wks	o/+ [L]	o/+ [L]	
32. Individual social skills training • 7 studies • A*: L L M M	ASD, *N* = 31, aged 17–20 years, 77% male	40 min to 1-hr for up to 33 wks	+ [L]	+ [L-H]	o/+ [L]
33. Intensive Interaction Support for specific communication skills • 8 studies (ID), 6 studies (ASD) • A*: L M (ID), L L M M M (ASD)	ID, *N* = 27, 28–53 years, 59% male	Frequent, usually daily, short-interval training	+ [L-H]	+ [L-H]	
	ASD, *N* = 57, 17–32 years, sex nr	10-50 min 1–2 times each week for 4-9 wks	+ [H]	+ [L]	
34. Theory of mind/ emotion/ social cognition training • 5 studies (ASD) 11 studies (PSD) • A*: L M (ASD), M (PSD)	ASD, *N* = 146, age and sex nr	30-min to 2 h/wk for 5–10 wks	o [L]		
	PSD, *N* = 495, mean age 25–44 years, 64% male	12 to 20 × 1-hr sessions	+ [M]	+ [M-H]	
Psychosocial wellbeing and capacity building support					
35. Telehealth-based supports or SMS prompting • 4 studies • A*: M M M	PSD, *N* = 178, 61–92 years, 15% male (nor for 2 studies)	12 weeks to 9 months (frequency nr)	+** [nr]		o/+ [nr]
36. Psychoeducation • 9 studies • A*: L L M M M	PSD (1 study PSD + ASD), *N* = 912, mean 32–38 years 85% male	1-2 sessions per week for 8 weeks to 2 years, from 30-40mins to 1.5-3 h p/week	o/+ [L-H]		o [L-H]
37. Mindfulness for social anxiety • 2 studies • A*: L	ASD, *N* = 91, age, and sex nr	2.5 h/wk for 9 wks	+ [L-M]		+ [L-M]
38. Cognitive Behavioural Therapy (CBT) based interventions targeting social functioning • 5 studies (ASD) 2 studies (PSD) • A*: L M M M	PSD, *N* = 87, 14–45 years, 100% female	Weekly or monthly 1-4hrs sessions for 6 to 24 weeks	o/+ [L-M]		
	ASD, *N* = 147, age and sex nr		+ [M]		+ [M]
39. Cognitive reframing • 4 studies • A*: L M M	PSD, *N* = 204, age and sex nr	1–2 or 14–22 sessions for 1–2 h each	+** [L-M]	+ [M]	+ [L-M]
40. Meta-cognitive training, Cognitive Enhancement Therapy • 4 studies (PSD) 2 studies (ASD) • A*: L M M	PSD, *N* = 143, mean 26–40 years, 66% male	36–45 sessions		+ o/+ [L-M]	+ [M]
	ASD, *N* = 68 ASD, mean 25 years, 86% male	18 months (session frequency/length nr)		+ [L-M]	+ [M]
41. Behaviour activation • 2 studies • A*: M	PSD, *N* = 113, age and sex nr	Up to 12 sessions	o [M]		o/+ [M]
42. Recovery-oriented clinical therapy • 2 studies • A*: L M	PSD, *N* = 56, mean 37–43 years, 45% male	20–45 min 1–2 times per week for up to 21 sessions	+ [L/nr]		
Vocational social skills interventions of supports					
43. Vocational internships and training and volunteering • 2 studies, 1 SR from an UR • A*: L L M	PSD, *N* = 112, M = 28–31 years, 64% male	nr	-/o/+ [M]		+ [M]
44. Vocational social skills or coaching programs • 3 studies • A*: L	ID, *N* = 15, 18–26 years, 60% male	3 h/wk for 12 weeks or during unpaid internships for 4–8 h/wk	+ [H]	+ [H]	+ [H]
45. Aspirations Program – vocational skills • 3 studies • A*: L L L M M	ASD, *N* = 71, 19–22 years, 86% male	4 to 20-hrs p/wk until independent in the workplace	o/+ [L]	+ [L]	+ [L]
46. Job interview and conversation skills training • 3 studies • A*: L L M	ASD, *N* = 150, M = 24–25 years, 86% male	1.5 to 2-hrs p/wk for up to 12 weeks		o/+ [L-M]	o [L]
47. Broad vocational social skills programs • 7 studies • A*: L L L M M	ASD, *N* = 153, 18–27 years, 85% male	∼15-min sessions to learn specific skills, 6-26 × 1–1.5-hrs sessions, or daily support for up to 6m		+ [L-M]	+ [L]
Relationship-focused interventions					
48. Dating, sex, and relationship programs, mostly in group settings • 7 studies • A*: L L L M M M M M	ASD, *N* = 201, 18–60 years, 62% male	1–2 or 1.5–2 h × 10–20 sessions	+ [L-M]	+ [L-M]	
49. Sex, relationship & family planning (group or individual programs) • 15 studies • A*: L M M	ASD or ID, *N* = 722, 12-59 years, sex nr	25–30 min to 2.5–3 h sessions for a total of 6–30 group sessions or 13–30 individual sessions	+ [L]	+** [L]	o [L]
50. Abuse prevention programs • 8 studies • A*: L M	ASD or ID, *N* = 175, 11-57 years, 50% male	40–60 min for 2–5 or 18–40 sessions		+** [L]	
51. SexG program on sexual health and responsibility • 4 studies • A*: H	PSD, *N* = 595, M = 37-40 years, 100% male	brief (6 × 1-hr session) and enhanced (13–15 × 1-hr sessions)		o/+ [M-H]	
52. Relationship and AIDS/HIV-prevention interventions • 9 studies • A*: H	PSD, *N* = 1,268, aged 22–59 years, 47% male	1.5–2 h × 4–10 sessions		+** [L-M]	
Life skill focused interventions					
53. Life Skills Training • 2* studies • A*: L M	PSD, *N* = 272, M = 48–52 years, 65% male	two × 2-hrs p/wk for 12 weeks		+ [L]	+ [L-M]
54. Parenting skills, knowledge, and safety training • 6 studies • A*: L H	ID, *N* = 155, aged 16-49 years	1–2-hrs × 4–5 or 10-26 sessions with home-visits in one study	+** [M-H]		
55. Digital literacy skills training • 4 studies • A*: L M	ID, *N* = 67, 18-23 years, sex nr	Duration/ frequency nr	o/+ [L-H]	+ [H]	
56. Navigation and travel training interventions • 11 studies • A*: L	ASD or ID, *N* = 171, M = 19-32 years, sex nr	Frequent short sessions for a total of 30–60 min	o/+ [nr]		
57. Life Story work • 2 studies • A*: L	ID, *N* = 71, aged 55–63 years, sex nr	2 individual 1hr sessions or 16 individual + group 1.5–2 h sessions	+ [M]		o/+ [M]

Notes: Blank cells indicate no evidence available. Detailed summaries of the study and sample characteristics and outcomes are provided in **Supplementary Table 4**, the numbered intervention categories in column one corresponds to the numbered paragraphs in the main text, and the numbered rows in **Supplementary Table 4**.

Abbreviations: L, low quality; M,  moderate quality; H, high quality; A*, AMSTAR (A MeaSurement Tool to Assess systematic Review); ASD, autism spectrum disorder; ID, Intellectual Disability; PSD, Psychosocial Disability; PEERS-YA, Program for the Education and Enrichment of Relational Skills for Young Adults; SR, systematic review; UR, Umbrella Review; h, hour; hrs, hours; min, minutes; nr, not reported; N, number; wk, week.

Symbols:+positive effect (green); o null effect (red); - harmful effect (red); / indicates mixed effects (amber); * sample size does not include the participants included in SRs within URs; ** effects in most studies were positive, but some studies showed no effect.

The evidence included in each review was predominantly low (22 reviews; 51.2% of reviews reporting study quality) or moderate quality (14 reviews; 32.6%). Only seven reviews predominantly included high quality evidence. Studies evaluating interventions for people with psychosocial disabilities were generally higher quality than studies of interventions for people with intellectual disability or on the autism spectrum. Quality was not assessed in 14 reviews and was unclear in one review (26).

### 3.3. Intervention design and outcomes

There were three broad types of intervention: (1) 12 interventions that helped people identify and connect with participation opportunities; (2) 14 interventions that were a participation opportunity; and (3) 31 interventions that focused on building skills or capacity to participate. Intervention outcomes are summarised in Tables 2–4, and detailed information on each intervention, study design and quality, and effects are provided in **Supplementary Table 3**. Facilitators and barriers are summarised in **Supplementary Table 4**. Category numbers below correspond to the intervention category in all tables.

#### 3.3.1. Interventions to help people connect with social, community or civic participation opportunities

Interventions focused on supporting people to connect with social or community participation opportunities used processes like person centred planning and individualized support for social functioning and participation; befriending or peer-based supports to broaden social networks; and transition supports for younger and older adults (Table 2).

*Person centred planning* (PCP) involves developing individualised plans with the person in partnership with their circle of support, with a focus on meaningful participation and goals (27). The PCP interventions (Category 1, low-high study quality) were primarily assessed in people with intellectual disability or on the autism spectrum in residential or day centre settings. PCP reduced loneliness and improved self-determination, interpersonal relations, social inclusion, contact with friends, and sense of connection and social contact, participation and involvement in community settings (e.g., restaurants, museums) and in community activities. PCP increased the variety of community locations visited, and level of access to community settings. There were inconsistent effects on social network size, no impacts on friendships with peers or social networks beyond close family and staff. There were 2.8-fold higher rates of participation in choice-making for short-term goals (e.g., whether to participate in specific activities) (28), but no impact on involvement in major life decisions (29). Plans were not developed for 30% of people in one study (28). Study quality varied enormously, and successful implementation required support from frontline staff providing individualized support through to service planners and managers (29).

*Skilled individualised supports* (Category 2, study quality not reported) for people on the autism spectrum or with intellectual disability encompassed *active support*, positive behaviour support, behavioural and residential assessments and modifications, functional communication training, planning, respite care, and crisis responses. Individualised support led to increased time in community settings and activities, facilitated convivial encounters in community settings, and reduced barriers to community interactions. Individualised support increased participation in employment or training but did not affect the incidence of challenging behaviour.

A previous umbrella review (25) summarised effects of interventions supporting *choice-making and asset-based approaches, social skills, setting goals and peer support* from one systematic review (Category 3, moderate quality study; frequency n/a). These interventions enhanced social inclusion, connectedness and quality of life, and reduced depression. Asset-based approaches improved self-esteem and health outcomes but had inconsistent effects on self-determination. Goal setting interventions required strong relationships between staff and participants, and were not as effective as interventions with asset-based approaches or that built social skills (30).

Animal-based interventions (Category 4) included a dog walking program alongside a dog handler for people with intellectual disability (high quality study), and short-term animal *companionship* for people with schizophrenia or depression (low-moderate study quality). Walking a dog increased convivial encounters and confidence to engage socially. One passive animal companionship study found improvements in social-adaptive functioning, and two others reported reduced depression symptoms or improved self-esteem, self-determination, and psychiatric symptoms. Animal companionship did not improve social support or loneliness. It was not clear if benefits were specific to animal companionship or participation in structured activities (31).

Two types of intervention focused on *enhancing community linkage* for people with psychosocial disability or intellectual disability by linking the person with community-based recreation or interest-based activities (Category 5, study quality nr). For these “*connecting people*” or “*social prescriber*” interventions people at risk of chronic health conditions, including psychosocial disabilities, were referred to a “navigator” who assessed their social participant and/or mental health needs, interests, and preferences and helped them connect with community programs or activities (Category 6, low-high study quality). Community linkage and social prescribing interventions led to increased social network size, including non-paid contacts, social connections and friendships, and reduced loneliness. Linkage supports improved community involvement and participation but had little effect on social activity and interactions in community settings. These interventions improved interpersonal skills, self-esteem, confidence and feeling worthwhile, but had inconsistent effects on mental health and general wellbeing. Building trust was vital in establishing relationships (32). Few evaluations compared linkage support interventions with control conditions, and low uptake in several studies suggests low acceptability.

*Befriending interventions* matched a person with psychosocial (study quality not reported) or intellectual disability (moderate-high study quality) with a volunteer befriender from the community (Category 7). Most befriending matches were based on shared characteristics and interests for people with psychosocial disability, and some also included stipends to support activity participation costs. For people with psychosocial disability befriending increased perceptions of social support, but did not affect loneliness, social functioning, social networks, general wellbeing, or psychiatric symptoms. A monthly stipend to the group receiving befriending support and a control group that received a stipend without befriending had similar increases in social functioning and network size. There was limited engagement for some participants, however, given that 23% (33) to 36% (34) of people with psychosocial disability never met their befriender. People with intellectual disability experienced few benefits to community participation and social network size, had little choice about the nature and frequency of interactions, and some reported negative effects on existing social networks when befriending activities interrupted regular schedules.

Other *friendship interventions* matched people with psychosocial disability to a peer with psychosocial disability (Category 8; e.g., the “Buddy Care” intervention), or focused on re-establishing connections with existing friends. Peer-based befriending increased social contacts and perceived social support, and improved overall mental health but had no effect on loneliness or social network size, psychiatric symptoms or service use. Friend-oriented psychoeducation successfully re-established social networks and increased social contacts.

*Peer support groups* in the community facilitated access to peers through the internet, mobile applications or face-to-face settings. Some peer support programs included a mental health professional facilitator alongside routine case management (Category 9). Community-based peer support groups were reported to be a welcoming community where people with disability could be themselves, share coping strategies, fill their free time, and interact with others. People with psychosocial disability experienced improvements in social belonging, connectedness, wellbeing, empowerment, hope and self-efficacy. Some studies reported limited impacts on social relationships with peers, no change in isolation, loneliness or connections with friends or family, and only short-term improvements in satisfaction with getting along with others that was not maintained to 6-months post-intervention. Peer support had inconsistent effects on quality of life and psychological wellbeing. While positive effects were observed for people who attended regularly in some studies, an internet-based peer support program found higher participation was associated with higher levels of distress (35). In that study, adverse effects on distress were attributed to potential overwhelm from the volume of interactions or “absorbing” distress from others *via* online discussion pages. Alternatively, people with higher distress may have engaged more with the peer group. Effectiveness was driven by having opportunities to participate in activities in the community (36). While only four of 10 studies had a quality appraisal, all were moderate to high quality, and seven studies compared peer support with control conditions.

Thirty-six studies assessed *peer support programs integrated into statutory mental health services* where peer mentors worked alongside clinicians to support people with psychosocial disability (Category 10, low-high study quality). A meta-ethnography by Walker and Bryant (37) examined mentor, mentee, staff, and service provider experiences, and other studies examined social and health effects of statutory peer supports. For service users with psychosocial disability, peer support reduced feelings of alienation, improved community reintegration, recovery, wellbeing, hope, motivation, friendships and social networks, and illness management skills. Peer support had no impact on social network support, social functioning, psychiatric symptoms, or quality of life. For peer workers with a psychosocial disability, providing peer support improved their own recovery, increased social networks, and led to other opportunities. Non-peer staff reported that peer workers could help service users belong in the community beyond being a “patient”.

*Transition programs* for young adults with intellectual disability or on the autism spectrum predominantly focused on adjustment to post-secondary education or learning social and academic skills, and goal setting (Category 11, low-high study quality). Transition support improved social participation with friends and other people both with and without disabilities. Transition programs also improved participation in leisure activities, and learning self-advocacy skills.

Transition programs for older adults with intellectual disability supported *transitions into retirement* through active participation in community groups aligned with the older adult's interests (Category 12, moderate study quality). Transition to retirement supports improved intimate relationships and awareness of rights, but had inconsistent effects on interpersonal relationships, social inclusion, self-determination, and emotional wellbeing. Effects were limited if programs did not support maintenance of existing networks or building new networks, or if people had insufficient resources to continue to participate after the research project ended (38).

#### 3.3.2. Interventions offering participation opportunities

These interventions included direct opportunities to participate socially or in the community (Table 3). Most activities were in disability specific settings, except for community groups (e.g., men's shed; category 13), some gardening interventions (category 19), and some sport-based interventions (categories 20–23). Effectiveness did not appear to differ between mainstream and disability-specific settings.

Three studies supported people to join *existing community group*s (e.g., Men's Sheds) that matched the participant's interests, sometimes training existing group members to support the participation of the person with a disability (Category 13, low study quality). Community group participation improved social satisfaction, social network size and time spent with new social contacts, but did not change loneliness, depression, physical health, or quality of life, possibly because the new relationships typically did not extend beyond the group setting (39, 40). Men's sheds offered the opportunity for meaningful participation and establishment of camaraderie to build a support network (41). Effects were enhanced if groups enabled genuine involvement in activities and social interactions with other group members through active mentoring (39).

*Music* programs (Category 14, low-moderate study quality) included the *Soundscape* program for people on the autism spectrum, which enhanced peer relations and self-esteem. Music activities for people with psychosocial disability included group singing in the community and music therapy focused on receptive (e.g., music appreciation and discussion) and/or active processes (e.g., music production or improvisation, singing, playing instruments). Group-based singing improved social functioning, belonging and connection to community. Attrition from choirs was influenced by changes in employment, worsening mental health, family problems, accommodation issues, and anxiety about singing ability (42). Music programs increased short, medium, and long-term social functioning, and had inconsistent effects on perceived social support, with superior effects from programs comprising group processes compared with education-focused programs. Music programs reduced anxiety, but had inconsistent effects on depression, cognitive functioning, psychiatric symptoms, and quality of life. Music therapy was particularly effective at improving negative symptoms such as affective flattening and blunting (i.e., a lack of emotional reactions), social relationships, and motivation (26). However, most of these interventions were provided in clinical inpatient or outpatient settings, and their acceptability and effectiveness in community-based settings was not clear.

*Dance programs* for people on the autism spectrum with or without an intellectual disability, and for people with psychosocial disability, focused on social skills (e.g., perspective taking or mirroring others; Category 15, moderate-high study quality). Dance programs improved interaction, imitation, emotion expression and regulation but did not affect social skills, self-other awareness, empathy, cognition, communication, or psychological wellbeing. People with psychosocial disability in dance programs felt valued by others and reported feeling empathy from others.

*Drama* activities for people with intellectual disability and/or psychosocial disability were predominantly provided in therapeutic programs that fostered storytelling, self-awareness, and building positive relationships with others. Two programs developed a performance presented to mainstream audiences (Category 16, high study quality or nr), which increased social acceptance and relationships with other participants and community members. The social aspects of group drama programs improved personal organisational and social development and established group harmony. Drama group participation increased social inclusion, acceptance, relationships, and friendships with other participants and community members; increased engagement with others and leisure activity participation; and reduced isolation. Drama group participants reported motivation to continue meeting other participants in a peer support group after completing the program. Drama participation also led to improved communication and social skills, self-awareness, awareness of others, and impulse control; and reduced challenging behaviours. Participants reported increased creativity, empowerment, confidence, self-worth, self-esteem, resilience, quality of life, mood, and recovery, and reduced perception of discrimination and self-stigma. As no evaluations compared drama with a control condition the mechanisms of benefit could have simply been related to the group setting or social interactions and not drama *per se* (43, 44).

Qualitative evaluations suggested that drama groups affected social functioning in two ways. First, group settings and activities enhanced *support and trust* (43), including fostering the ability to share and collaborate with others (45) and building relationships (46). Participants enjoyed observing others' resilience and resourcefulness in a crisis (47). Participating with others with similar experiences was helpful (47, 48) and resulted in a sense of safety to explore socially inappropriate behaviours (49), although drama groups led to increased sense of vulnerability for some (50). Second, through drama people *learned more about themselves and built their self-concept, confidence and empowerment* (45, 46, 50, 51). Participants explored their individual experiences to learn more about themselves (47), which improved their self-awareness and sense of control (52).

*Arts-based activities* (Category 17, low study quality) for people with intellectual or psychosocial disabilities focused on developing artistic skills and creating art. Some programs included community exhibitions to display and sell artwork. Participants typically created art alongside artists without disabilities or received instruction and guidance from an instructor. Programs for people with psychosocial disability included art studio programs in community centres or psychiatric rehabilitation settings for people with a mental health problem or as “arts on prescription”. Art studio participation enabled creation of a “community of artists” that fostered links with the broader community, including convivial encounters with community members (53). Participation increased social inclusion, sense of belonging, engagement, mutual support, social connections, friendships, meaning in life, self-esteem, happiness, and confidence. Participants enjoyed receiving praise for their work from community members. Selling artworks gave artists a presence and voice in the community, fostered a sense of achievement, and positively contributed to identity-related outcomes. Art participation led to broader positive life outcomes (e.g., employment, housing, recovery, quality of life, wellbeing), and reduced distress and psychiatric symptoms. Studio facilitators who worked alongside participants fostered a sense of equality, inclusion, belonging and intersubjectivity (54). People enjoyed being able to offer and share with others (54). However, arts interventions were not compared with a control condition, and the social inclusion benefits may be due to the broader collaborative and creative group settings rather than art-based processes (55).

*Farm, ecotherapy, gardening and horticultural interventions and groups* for people with psychosocial disabilities (Category 18, low-high study quality) included short-term interventions, vocational training programs or long-standing community “allotment” programs. Farm-based programs involved working with farm animals including feeding and grooming animals, milking cows, and riding horses. Interventions led to reduced loneliness, increased social participation, new friendships, and improved self-efficacy, coping, mood and general mental health. The evidence was weak and limited as no studies compared the interventions with a control condition and studies were predominantly low-moderate quality.

*Outdoor recreation and leisure programs* for people with psychosocial disability included structured programs (e.g., information sessions, personal development workshops, self-help groups, community walks and forums), and nature-based programs (e.g., camps or dolphin therapy; Category 19, high study quality). Interventions led to improved social connectedness, relationships, interpersonal relationships, personal growth, confidence, wellbeing, self-determination, and empowerment, and reduced loneliness and depression.

*Sports and physical activity participation* opportunities were evaluated primarily for people with psychosocial disability and intellectual disability. Three meta-ethnographic reviews examined motivations and barriers to physical activity participation for people with schizophrenia (56), participant experiences when starting community-based group physical activity (57), and physical activity participation experiences (58) (Category 20, moderate-high study quality). Other studies evaluated social outcomes after *physical activity programs* or *sport participation* for people with psychosocial disability such as soccer training and games, learning and practicing yoga, outdoor or nature-based recreation (e.g., white-water rafting) and fitness programs (e.g., aerobic, interval, resistance, and strength training; Category 21, low-high study quality).

Only 27% of people endorsed that the social aspect of exercise was a motivator (socio-ecological motivators) (56). Physical activity participation led to several outcomes, including:

1. **Psychosocial**: Improved socialization, social/emotional support, empathy, sense of warmth, companionship, sense of control, sense of achievement, self-appreciation, confidence, self-esteem to engage in the community, and autonomy. Yoga led to improved psychosocial functioning, and nature-based therapeutic recreation and soccer and football participation had positive impacts on relationships, social inclusion, and isolation; however, interval training had no effect on psychosocial functioning.
2. **Mental health and recovery**: Fewer hallucinations, psychiatric symptoms and improved overall functioning, but only for participants who attended ≥50% of exercise sessions (59–61); improved mood, relaxation, and mental health, but only for studies with aerobic with resistance training methods with ≥90 min per week of moderate-vigorous exercise (62, 63). Programs that enhanced cohesion and relatedness between participants led to reduced anxiety.
3. **General health**: Improved fitness, sleep, and quality of life. Weight loss was motivating and considered “a yardstick for recovery” (58).

*Mainstream sports programs or physical activity* in the community for people with intellectual disability included team sports, active recreation, or walking with a person without intellectual disability (Category 22; low-high study quality). *Unified Special Olympics* (SO) programs included athletes with intellectual disability and age and ability matched people without intellectual disability who played in the same teams (Category 23, low-moderate study quality). Mainstream sport participation led to increased opportunities for convivial encounters but had inconsistent effects on interpersonal relations. Unified SO team participation led to improved friendships, social inclusion, access to community venues and sense of community belonging, and alliances within local communities. Programs provided “a platform for the development of social relationships”, and participants reported that they felt like they were “a part of society” (64). Wilhite and Kleiber (65) found more improvement in community involvement for people with moderate-severe intellectual disability, perhaps because people with mild intellectual disability already had relatively good community involvement. Participants enjoyed learning and playing sports and receiving praise or acknowledgement from others, and reported improved emotional wellbeing and physical activity levels. Participation in unified SO led to better social self-perception and acceptance and reduced maladaptive behaviours.

*Disability-specific exercise, physical activity, and leisure programs* for people on the autism spectrum or with intellectual disability primarily focused on strength, balance, fitness, and health (Category 24, low-moderate study quality). A leisure program for people on the autism spectrum used a PCP approach and focused on fostering social collaboration and support, and provided 2-hours of leisure activities in the community five days each week. The intervention led to improved interpersonal relationships, social support, belonging, life satisfaction, quality of life, self-efficacy, psychosocial wellbeing, quality of life, empowerment, and employment. There were inconsistent effects on community integration and adaptive behaviour, and no effect on social integration, leisure needs, engagement, or satisfaction. A lack of transport and psychosocial support limited continued participation (66).

Seventeen studies evaluated *traditional SO* training and participation for people with intellectual disability (Category 25, low study quality), or did not report whether the SO setting was unified or disability-specific (Category 26, low study quality). Traditional SO participation was associated with improved social self-perception, meeting people, making friends, community awareness, inclusion and involvement, independence in the community, social behaviour; and reduced challenging behaviour. Participation was associated with improved social skills, exercising choice, receiving social approval and acceptance; having fun, happiness and enjoyment; and physical health and sport skills. SO participation was described as playing an important role in the lives of individuals with intellectual disability, their families and the community (67).

#### 3.3.3. Interventions to develop skills or psychosocial capacity to participate

Capacity-focused interventions aimed to improve social, communication and relationship skills, psychosocial wellbeing and life skills, and navigation of digital information or the community (Table 4). Most interventions were in disability-specific settings except for vocational social skills interventions in the workplace (category 43–47). Interventions targeting psychosocial wellbeing were included only if they addressed social participation, linkage, capacity, or outcomes.

##### 3.3.3.1. Group-based social and communication skills training

For people with psychosocial disabilities, studies evaluating group-based social skills interventions were low-moderate quality and included:

• *Social Skills Training* (SST; Category 27) of interpersonal skills, social problem solving, social perception, theory of mind, social information processing, interaction skills, understanding social norms, and applying skills to everyday situations;
• *Social Cognitive Training* (SCT; Category 28) to reframe loneliness perceptions, and build self-control, coping strategies, sense of belonging and stress management; and
• *Social Cognition and Interaction Training* (SCIT; Category 29) of social cognitive dysfunction, sometimes using augmented reality simulation and cognitive remediation approaches.

Most social skill interventions were delivered alongside other clinical supports (e.g., case management, illness management, family-oriented psychoeducation). SST and SCIT improved social functioning, role functioning, social relations, and social activity participation, and reduced social isolation. SCT improved affect recognition, but only reduced loneliness after more intensive programs. SST improved behavioural skills, social skills, theory of mind, goal attainment and recovery, had small effects on non-verbal social skills, and no effects on perceived social support. SCIT had inconsistent effects on social capacity, theory of mind, affect recognition and attribution style and no effect on interpersonal communication. Interventions that provided frequent contact with a therapist (68), used a range of methods to enable transfer of learned skills into everyday life (69), and provided elements of training in community settings (70) were most effective at helping people to apply learned social skills.

Group-based social skills training for people with intellectual disability targeted social awareness and competencies including interpersonal communication and listening skills (Category 30; e.g., a TEACCH-based program; moderate-high quality studies; SCIT program, “Putting feet on my dreams” and “Problem Solving Skills 101”; low study quality). Training reduced social withdrawal and improved relationships with partners and friends; increased confidence and knowledge to participate in the community and joining or establishing support/social groups; and improved self-concept and quality of life in people whose understanding of civil rights and engagement also improved after group training.

Group-based programs for people on the autism spectrum used instruction, discussion, and rehearsal of social and communication skills with video feedback, including the *PEERS-YA* program (Category 31; low study quality). Interventions increased invitations to social get-togethers, but had inconsistent effects on social responsiveness, socialisation, social skills and behaviours; conversation skills (e.g., initiating and maintaining conversation, reducing inappropriate utterances, attention, and feedback to questions), and emotion identification. There were similar improvements in social functioning and theory of mind in the intervention and control groups who also participated in a social interaction group without training, and there were no effects on hosting get-togethers, loneliness, broad social communication skills, social performance, empathy, and social body language (e.g., eye contact, gestures).

##### 3.3.3.2. Individual social skills training

*Individual multifaceted social skills interventions* for people on the autism spectrum or with intellectual disability provided psychoeducation, coaching and training to use tools like a digital planner to schedule activities (Category 32, low-high study quality). Interventions increased social event attendance, peer interaction satisfaction; social skills (e.g., initiation and maintenance of interactions, social skill performance, and timely responses to questions), and employment and quality of life.

Fourteen studies evaluated individualised interaction support training of *specific communication impairments* for people with intellectual disability or on the autism spectrum (Category 33, low-high study quality). Interventions improved social behaviour and the targeted social skills, while also reducing challenging behaviours. There were limited effects for people with severe and chronic challenging behaviours following short-term interventions (71), and gains were not consistently maintained post-intervention for people with more severe intellectual disabilities. One RCT compared a Virtual Reality (VR)-integrated computerised training program with an active control group who also received computerised training and found no differences in improvement between groups. Barriers to implementation included inconsistent capacity or maintenance of individualised interaction support by support workers over time (72–75).

Sixteen studies evaluated interventions targeting *specific social competencies* like theory of mind, emotion perception, and social perception (Category 34) for people on the autism spectrum (low study quality) or with psychosocial disability (moderate-high study quality). Interventions did not affect social functioning for people on the autism spectrum, but intensive interventions improved social and occupational functioning, social perception, theory of mind and affect recognition for people with psychosocial disability.

##### 3.3.3.3. Psychosocial wellbeing interventions to enhance participation capacity

*ehealth interventions* provided people with schizophrenia, schizoaffective disorder, or depression, and people on the autism spectrum support to manage symptoms and enhance socialization through telephone or SMS-based prompting (Category 35, study quality nr). Interventions increased social interactions and leisure activity participation but did not change loneliness.

*Psychoeducation* for people with psychosocial disability, including people on the autism spectrum who also had a psychosocial disability, to learn problem solving and coping skills, illness management and encouraged social participation through computer or web-based programs or in-person programs (Category 36, low-high study quality). Some interventions also included family therapy. Interventions improved social functioning, social contacts, and loneliness, but effects were not consistently maintained. There were inconsistent effects on quality of life and no effects on psychological wellbeing, depression, or perceived social support.

Group-based *mindfulness* programs for people on the autism spectrum focused on awareness and management of social anxiety (Category 37) and led to reduced anxiety, depression, rumination, agoraphobia, and somatisation, and improved positive affect.

Individual or group-based *Cognitive Behavioural Therapy* interventions focused on behaviour activation, social interactions, and social anxiety for people with psychosocial disability or on the autism spectrum (Category 38, low-moderate study quality). *Cognitive reframing and remediation* interventions for people with psychosocial disability targeted cognitive strategies to analyse social situations and increase social interactions (Category 39, low-moderate study quality). Interventions did not influence loneliness after brief interventions (e.g., two 30-minute cognitive reframing sessions), but did reduce loneliness after for a more intensive intervention (e.g., five 4 h sessions). Interventions improved social cognitive processes, attribution style, empathy, theory of mind; schizophrenia, depression and anxiety symptoms, and daily functioning; and personal and social performance. One intervention led to reduced perceptions of social support in ex-military officers with PTSD (76), which may have been a spurious finding given that participants also reported improved reactivity to criticism of family members; however, these poorer outcomes suggest that interpersonal skills interventions may require more supported practice than what the brief intervention offered.

*Cognitive Enhancement Therapy* and *meta-cognitive training* for people with schizophrenia or on the autism spectrum were delivered in individual and group sessions targeting impairments in social and non-social information processing, cognitions and problem solving (Category 40, low-moderate study quality). These interventions improved global social functioning and perception, cognitive style and social cognition, and reduced disability, but had inconsistent effects on theory of mind and affect recognition.

*Behaviour activation* interventions taught people with depression to assess, prioritise and practice their values and goals (Category 41, moderate study quality), and led to decreased depression symptoms but did not change perceived support.

*Integrated Psychological Therapy for Schizophrenia* and *Interpersonal Community Psychiatric Treatment* are clinical therapies that focus on recovery and enhancing community participation (Category 42, study quality low or not reported). Treatment led to improved social perception knowledge, social networks, and social activity.

##### 3.3.3.4. Vocational social skills training

For people with psychosocial disability, vocational interventions focused on creating occupational opportunities for people with psychosocial disability to work in mental health services (e.g., the *Empowerment of Mental Illness service users: lifelong Learning, Integration and Empowerment* project; Category 43, moderate study quality), or to do volunteer work (Category 43). The internship intervention improved social life, social contacts, and networks for most people, but maintaining relationships was difficult. Volunteer work increased social inclusion, social ties, and social engagement opportunities, but also put people at risk of stigmatising experiences in the community (77).

For people with intellectual disability vocational interventions targeted social skills at work (Category 44; e.g., Walker Social Skills Curriculum, covert job coaching or video-based instruction; high study quality). Interventions increased social interactions over time; improved social competence, interpersonal skills, social skill mastery and social participation; improved employment rates, job security, and ability to perform work roles; and reduced challenging behaviours.

Programs for people on the autism spectrum focused on *social and vocational skills education*, and support to find and maintain employment (the Aspirations Program; Category 45, low study quality), *job interview conversation skills* for people on the autism spectrum (Category 46; e.g., The Molly Porter Job Interview VR training program, or Social Skills Curriculum for job interview-related skills, low-moderate study quality), or *training of social skills* for vocational settings (Category 47; e.g., social skills required for a work role, such as gestures like waving, while dressed as a mascot; low-moderate study quality). Training improved empathy but did not improve peer relations or socialisation despite anecdotal reports of improvements. Job interview training improved interview communication skills, but did not improve interview performance in one study, and did not affect confidence or adaptive behaviour.

##### 3.3.3.5. Relationship-focused skills, knowledge, and behaviour training

Relationship programs for people on the autism spectrum without intellectual disability (e.g., Ready for Love), or for people with intellectual disability (e.g., Friendships and Dating Program, Early Dating Skills Training, or Dating Skills Program; Category 48, low-moderate study quality) were predominantly group-based programs. Interventions improved social skills, dating skills and knowledge; and increased empathy, social responsiveness (i.e., autism-specific social impairments and skills), social functioning, and endorsement of dating behaviours (e.g., kissing, gay and lesbian relationships, sexual intercourse values and morals, keeping secrets). While social network size increased there were no changes in network composition. Participants wanted training that was relevant to their own relationship and sexuality aspirations including lesbian, gay, bisexual and transgender (LGBT) issues and concerns (78), and that included their partner if they were already in a relationship (79).

Fifteen studies evaluated *sex, relationship and family planning* for people on the autism spectrum and with intellectual disability in group or individual programs (Category 49, low study quality). Interventions had broad curricula, including anatomy, puberty, reproduction, sexually transmitted diseases, sexual intercourse, relationships, dating/romantic skills, safety/consent/abuse, self/other in sexuality and relationships, and private/public appropriate/inappropriate behaviours. Program participation improved “social entertainment”; understanding of friendships, interactions with people of the opposite sex; dating problem solving skills; knowledge of sexuality rights, responsibilities, and vocabulary; and endorsement of dating behaviours. There were inconsistent effects on sexual knowledge, improved social skills, and self-protection skills. All studies were low quality, eight of which did not have a control group, and the control group conditions were not described for four RCTs.

*Relationship abuse prevention* interventions (Category 50, study quality low or not reported) for people on the autism spectrum or with intellectual disability taught decision-making strategies to resist sexual, physical, and verbal abuse. Training increased knowledge of abuse concepts, empowerment, and recognition of inappropriate touching requests. There were inconsistent but mostly positive effects on decision making ability, and no effects on appropriate touching requests. Some people required booster training to maintain and generalise abuse prevention skills. Younger people and those who found the program more difficult had the biggest improvements in relationship knowledge and behaviour (80).

Sex and relationship programs for people with psychosocial disability focused on increasing safe and responsible sex behaviours and attitudes (e.g., SexG group-based interventions; Category 51, moderate-high study quality) or targeted prevention of AIDS and HIV risks (Category 52, low-moderate study quality). In the SexG interventions with men, discussion and role play of safe sex, responsibility, and knowledge, confidence, and motivation to use condoms had inconsistent (but mostly positive) effects on risky sexual behaviours. Interventions targeting knowledge and behaviour to prevent HIV and AIDS increased sexual assertiveness, knowledge and confidence to deal with high-risk situations, and contraceptive use, and reduced risky sex acts, the number of casual sex partners, total number of sex partners and unprotected sex.

##### 3.3.3.6. Life skill interventions

*Life skills training* interventions included broad programs on medication management, organisation and planning, transportation, and financial management for people with psychosocial disability (Category 53, low-moderate study quality; e.g., Functional Adaptations and Skills Training program). Life skills training improved social skills but did not affect quality of life.

*Parenting skill, knowledge, and confidence training programs* for people with intellectual disability (Category 54, moderate-high study quality) were delivered individually to improve parenting safety and interpersonal and communication capability and led to improved childcare skills that were maintained over time, and health knowledge (e.g., life threatening emergencies and using medicine). The evidence was moderate to high quality; however, two studies did not include control groups.

Four studies evaluated *digital literacy skills training* for people with intellectual disability to use email or participate in social media (Category 55, most studies high quality). Training improved participants' ability to complete tasks in social media platforms (i.e., Facebook) and email training reduced social isolation. Blogging training did not affect social capital (i.e., the resources that one can access through their social connections).

Independent *travel and navigation skills* training for people on the autism spectrum or with intellectual disability (Category 56, quality not reported) was provided using augmented reality, multimedia, smartphone applications and maps. Augmented reality training led to reduced travel planning time, and improved navigation skills and public transport use. Multimedia and video-based travel skill training improved pedestrian bus route navigation skills that were maintained over time. It was not clear whether skills learned in virtual environments would transfer to natural environments (81, 82), or when a support person is not present (83). Participants benefited more from interventions that meaningfully blended real world experiences with digital information (84).

People with intellectual disability were supported to share their personal history using *Life Story* work (Category 57, moderate study quality) when changing residential locations or joining a new social group. Life Story work improved interpersonal relationships, rights, social inclusion, and self-determination, but had inconsistent effects on emotional and physical wellbeing.

### 3.4. Intervention implementation considerations

#### 3.4.1. Acceptability, implementation, and maintenance

The literature highlighted that existing staff skills, attitudes and policies can negatively affect implementation (85, 86), and programs in residential or community settings needed to be embraced at all levels of the organisations from frontline support workers to service planners or managers (29). Staff need training (72, 73) and dedicated time and resources to provide (28) and maintain planning support over time (71–74). Moreover, staff or family members sometimes ignore, reinterpret or misinterpret the preferences of people with intellectual disability (87), so focus on the individual and their changing needs and preferences over the lifespan must remain a central focus (88).

To enhance intervention acceptability and maintenance both the intervention facilitators (89) and people with lived experience should contribute to intervention development and delivery (89, 90). A codesign approach can help ensure that the content is relevant to participants’ needs or aspirations (78). As participants with disability attending training opportunities may know more (or less) than they seem to, information should be presented in multiple formats using simplified and accessible language (91–94), with information and questions read aloud to improve program acceptability and effectiveness (93). It is important that facilitators gain an understanding of existing relationship skills, knowledge, and interests of people with intellectual disability (86). People with poor digital literacy skills face greater barriers in connecting with others (94). Therefore, programs should provide digital and text-based literacy support and adapt materials for people with different levels and types of impairments (91–93, 95). People with no experience with computers or gaming may find it difficult to use virtual and augmented reality-based interventions (96), and rarely used video prompts (83). Interventions need to address essential life skills (e.g., social skills, literacy, time management, problem-solving, and other cognitive skills) that are needed for participation and establishment of relationships that extend into everyday life (97–99). Moreover, to participate in the community (e.g., in sport or physical activity), people needed to feel “well enough”, the activity must be affordable and in an appropriate location for “people like us”, and people may only participate if they expect positive outcomes (e.g., access to support, talking with others with similar experiences, seeing/making friends) (57).

#### 3.4.2. Facilitators and barriers

The key facilitators and barriers that generally applied to all three disability cohorts were predominantly related to (a) attributes of the program or intervention; (b) Carer, staff, facilitator, or peer mentor attributes; (c) participant attributes; and (d) community-specific characteristics.

##### 3.4.2.1. Attributes of the program or intervention

Success of participating in group programs often depended on the skills of the facilitator. Programs that effectively engaged participants had facilitators that provided multiple types of support, such as active mentoring to support participation in activities and social interactions (39, 100), and positive leadership and acceptance of people with disability (101). Participants liked programs with structured approaches, rules or policies (102), and homogeneous group characteristics (e.g., similar age) (103) with minimal participant turnover (104, 105). Participants liked having choice about which activities they could participate in, and having regular breaks, rewards, and positive feedback (38, 105–107). People often need support to maintain existing networks or to build new networks (38). Some people with psychosocial disabilities preferred individual over group formats and reported that attending new environments was challenging (58). Adherence was enhanced when facilitators had lived experience of the same disability (108), and being around others with similar disabilities could enhance the sense of community and opportunity to interact with and learn from other adults with similar experiences (47, 48, 98).

Participation was facilitated in programs that use person-centred, strengths-based approaches, and included supports to enable people to have freedom of choice (106, 107), and to set their own goals (90, 104, 106, 107). It was important that needs and priorities were reviewed over time to ensure participation opportunities remained relevant (88). Relationship-focused interventions benefited from tailoring interventions to participant needs (79), and adapting content to each person's circumstances (109). Fostering choice was a facilitator of outcomes; however, several studies noted that choice making for people with intellectual disability was often ignored, misinterpreted (87), or overpowered by caregivers, staff, family (87, 110) or community volunteers (111).

Participation was enhanced when people could have frequent contact with the intervention provider (68). Interventions were more effective when they incorporated real world contexts (84) including opportunities to practice skills or participate in community settings (68–70, 104, 112–115) with a trained facilitator (69, 112). While a single session might be enough for some participants to learn new social skills, other people required booster support or continued training to maintain skills (116). Moreover, homework was considered to be helpful by participants in some programs (117), and helped to generalize skills into everyday life (91, 92).

Participation in sport or community groups was diminished for people with poor access to transport, lack of psychosocial supports or staff to encourage participation, and limited financial resources to continue to participate (38, 57, 58, 66, 105). For people with psychosocial disability feeling dependent on others (e.g., needing reminders) was also a significant barrier to participation (57, 58).

##### 3.4.2.2. Supporter, staff, facilitator and mentor attributes

Staff were one of the most important sources of emotional and instrumental support to facilitate goal attainment in person-centred planning interventions (118). In art programs, moderators working side-by-side with participants facilitated inclusion and belonging (54). Physical activity participation was more successful if supporters consistently encouraged participation (56, 105–107, 119).

Befriending and peer mentorship were more successful when volunteers were matched to the participant attributes including personality, hobbies and interests (e.g., sports), age and gender (120). Successful peer mentors were understanding, empathic, punctual, flexible, and professional. Participation was improved when mentors took time to get to know their mentee and to establish a comfortable relationship (121), and set boundaries where necessary (e.g., in the study by Curtin, Humphrey (122) one mentee thought that they were dating their mentor). For some people it was important to be matched to a mentor with or without the same type of disability (123).

Building trust was integral to establishing a sense of safety in group programs (49, 50) and when working with social prescribers (32, 124, 125). People with psychosocial disability reported disengaging from activities if they felt unsafe, feared injury (32), had social anxiety, were apprehensive of strangers, or if they had negative expectations (e.g., feeling vulnerable, embarrassed, disliking feeling controlled by others, having to interact with others, or pain) (57, 58).

##### 3.4.2.3. Participant attributes

Community and social participation was reported to be easier for people with friendly dispositions and relatively good social skills (101) or literacy (94, 96), but was hampered for people with low social capital, including low levels of education, literacy, and family finances (126). Conflicts with existing personal commitments or valued activities can impede physical activity participation in new programs (57, 58, 127). Some people reported being teased by other participants (102) or peers (128), which diminished their sense of belonging. Social prescribing interventions needed to establish realistic expectations as failure to achieve expected benefits could have negative impacts on confidence (129). Lower participation in physical activity for people with psychosocial disability was affected by lifestyle factors (e.g., smoking, diet, sleeping patterns, fitness level and confidence), intrusive or fluctuating psychiatric symptoms, fatigue, sedative effects of medications, and low self-esteem (56–58). For people with intellectual disability, continued physical activity participation can be hampered by advancing age of the participant or ageing parents (130).

##### 3.4.2.4. Community-specific characteristics

Planning, linkage and befriending programs often faced difficulties with engaging people in the community who could foster community connections (131), or could not reliably recruit volunteers who could provide befriending support (111), leading to lower levels of engagement (132). Peer support workers (37), and volunteers (77) with psychosocial disability are at risk of experiencing stigmatising attitudes in the community, strain from over-commitment, and social exclusion.

## 4. Discussion

Interventions were identified that (a) support connection with social, community or civic participation opportunities, (b) provide participation opportunities that increase the sense of inclusion, belonging and participation, and (c) build capacity to enhance social skills and wellbeing to enable social and community participation. While most interventions successfully improved capacity and skills to participate, or actual levels of participation, some interventions had the potential to lead to worse participation or had negative impacts on quality of life. The findings reinforce the importance of individualised planning and support to identify and link people with participation opportunities, and to account for existing skills, social networks, and confidence to participate socially or in the community, as per the socio-ecological (133) and Quality of Life models (1).

### 4.1. Interventions for people with intellectual disability

For people with intellectual disability, interventions that consistently improved participation used individualised and person-centred approaches. While asset-based approaches provided excellent opportunities to enhance participation, other successful interventions targeted specific deficits, such as communication or social skills, or important life domains such as dating or parenting roles. The following interventions and supports effectively supported social and community participation for people with intellectual disability:

• Strengths or asset-based interventions to support choice making
• Person centred planning
• Skilled individualised interaction support
• Receiving support to link with or participate in community groups
• Peer support and transition programs both for youths transitioning into post-secondary opportunities and older people transitioning to retirement
• Group-based social skills interventions
• Vocational social skills support
• Relationship and family planning programs
• Parenting skills and knowledge interventions
• Helping people to create a “Life Story” to share their history
• Dog walking in the community
• Art and drama participation
• Participation in physical activity (e.g., Special Olympics programs)

Interventions with inconsistent effects on participation included digital literacy and participation programs, abuse prevention training, and travel or navigation training. Participating in physical activity and sports events helped people to build acceptance, confidence, sharing and friendships (134); however, there were inconsistent effects for sport-based activities in several studies. Befriending interventions were poorly implemented for people with intellectual disability, with the individual having limited choice and control over their interactions and experiencing potential negative impacts on existing social networks. To enhance effectiveness, befriending programs need to (a) define the target population; (b) balance frequency, length and modality of befriending activities; and (c) ensure there is appropriate infrastructure in the befriending services to support training and maintenance (132). Most of the evidence for interventions for people with intellectual disability was low or moderate quality, and many programs or supports needed to be adapted to each person's individual impairments, comorbid conditions, needs and preferences.

### 4.2. Interventions for people on the autism spectrum

Most interventions for people on the autism spectrum focused on building social, communication and relationship skills. The following interventions effectively supported social skills and participation of people on the autism spectrum:

• One-on-one training in social, conversation and communication skills
• Dating and relationship skills and knowledge programs
• Vocational social skills programs targeting social behaviours at work, including daily coaching
• Peer support, mentoring and support to transition into post-secondary education
• Music programs, and mindfulness training to reduce social anxiety

While people on the autism spectrum liked meeting with other people with similar disabilities and experiences, group-based social skills training had limited effects on social and communication skills. Instead, one-on-one training and individualised strategies targeting social and communication impairments were more effective. Interventions targeting higher order social competencies, such as theory of mind or social cognition, were not effective. Other interventions with inconsistent effects on social skills and participation included dance-based programs, and some vocational social skills programs (e.g., the Aspirations program, or training in job interview or conversational skills). Therefore, our findings oppose the NICE (135) guideline recommendation for social skills groups as a first-line treatment for people on the autism spectrum given that only individualized programs were consistently effective for adults. Finally, there were inconsistent effects on participation from disability-specific sports and physical activity programs, use of telehealth or SMS-based supports, psychoeducation, and interventions targeting meta-cognition (i.e., thinking about thinking) for people on the autism spectrum. Most studies were low quality, and few compared interventions with control conditions.

### 4.3. Interventions for people with psychosocial disability

For people with psychosocial disability, interventions enabling linkage with participation opportunities and building skills and psychosocial wellbeing to enable participation were effective, particularly when provided alongside illness management in line with the recovery framework (136). Interventions that effectively supported social and community participation of people with psychosocial disability included:

• Social prescribing, community linkage and “connecting people” interventions
• Befriending when matched with volunteers with common characteristics and interests
• Peer support, as a mentor or mentee, in community and clinical settings
• Social skill training in individual or group settings
• Training specific social competencies (e.g., theory of mind)
• Sex, relationship, and life skills training
• Art and music participation
• Ecotherapy, gardening, horticulture and outdoor nature-based activities and camps
• Sport and physical activity participation
• Vocational and internship programs with a focus on social skills or participation
• Psychoeducation and ehealth or SMS-based supports
• Recovery-oriented supports (e.g., psychoeducation) with a focus on social functioning and participation

While peer support and volunteer participation had several benefits, being a peer mentor in a clinical setting, or a volunteer in the community, could also lead to the experience of prejudice and stigma. Therefore, these opportunities need careful facilitation to minimise potential negative impacts.

Social skills training was not recommended in the most recent NICE guidelines for people with schizophrenia due to insufficient robust RCT evidence (137). However, we found consistent evidence of positive effects of social skills training on social functioning and social skills for people with psychosocial disability, with most evaluations published after 2017 using RCT designs. Unlike studies with people on the autism spectrum, training higher order social competencies in people with psychosocial disability improved social skills. While most of the studies in both populations used RCT methods, the autism studies had small samples and were predominantly low quality whereas the psychosocial disability studies had large samples and were predominantly moderate-high quality, which may explain the different outcomes in each population. Behaviour activation, which is an approach that emphasises scheduling enjoyed activities, had no effects on social functioning.

### 4.4. Practical considerations for implementing interventions

Overall, the effectiveness of interventions was impacted by a range of factors. In particular the setting within which the intervention or support was provided, and the attributes both of the program and the attributes and behaviours of the supporters, staff, and/or facilitators. In brief, programs were more effective and acceptable if they were person-centred, used strengths-based strategies with supporters or facilitators who worked in partnership with the individual to enable them to exercise choice and self-direction. Moreover, it is important to note that the attitudes and behaviours of people in the community can impact positively (e.g., feeling like they are welcome and valued in a community group) or negatively (e.g., experiencing stigma or social exclusion when in community settings) on the experiences of people living with disability. Strategies targeting both specific settings (e.g., training and mentorship for community or sporting groups) through to broader education and integration of people with a range of disabilities into civic life could help to overcome some of these experiences.

Participant attributes also need to be considered when building social and community participation given that people with relatively good social skills, friendly disposition or literacy skills were better able to benefit from the supports offered. Therefore, it may be that people need multiple sources of support to build their capacity, or to ensure that community settings are welcoming, in order to enable people on the autism spectrum, and those with intellectual or psychosocial disabilities, to participate meaningfully in social or community settings.

### 4.5. Evidence gaps

Several types of intervention were not included in systematic reviews for some or all disability cohorts, despite growing evidence of their effectiveness. These include transition programs focused on independent living, supported education/transition support (138) and person-centred planning (139) for people with psychosocial disability. The utility of training to use communication support tools was limited to social media or email use. The effectiveness of augmentative and alternative communication aids and strategies has been studied and reviewed extensively in paediatric populations (140), but not in adults and therefore could not be included in this umbrella review. Interventions to support civic participation were limited to volunteering for people with psychosocial disability and building civic rights awareness in people with intellectual disability. Interventions targeting other types of civic participation such as voting or advocacy were not identified. Finally, interventions targeting inclusive community settings or environments (141, 142) are important in the social model of disability (14), but were not identified.

### 4.6. Limitations

This umbrella review was limited by the level and type of details reported in the respective systematic reviews, which were predominantly low to moderate quality. Most reviews provided little information about factors affecting the feasibility, acceptability, and effectiveness of different interventions. Most reviews did not report specific outcome measures used in each study, or the magnitude of effects. Moreover, few reviews described the resources or funding required to deliver the interventions. Cost-related impacts were only noted for two of the 522 studies, which highlighted that a social skills program for people on the autism spectrum was not expensive or time consuming (143), and that providing participation support did not increase overall support costs (144). An additional 260 studies published between 2010 and 2020 were identified that evaluated interventions that met our inclusion criteria, but had not been included in the systematic reviews, suggesting that some systematic reviews missed eligible studies.

The low-quality evidence in this review is likely to have been driven by several factors. First, disability research has historically been under-resourced, making it difficult to conduct large-scale robust RCTs. Moreover, social, communication and participation-related impairments in intellectual disability, the autism spectrum and psychosocial disability often vary substantially both within and between cohorts, and many people need individually tailored supports. Therefore, designs such as multiple baseline or case study approaches are often more suitable than RCT evaluations of standardised interventions. Studies evaluating interventions for people with psychosocial disability were typically better quality, and more often used RCT designs.

### 4.7. Conclusions

Overall, interventions that support people to have both the capacity and access to social and community participation opportunities improved participation for adults on the autism spectrum, with intellectual disability, and psychosocial disabilities. It is important that people have access to personalised supports, where possible, and that they are given the opportunity to practice skills with active support or mentoring in the community in real-life settings.

## Data Availability

The datasets for this study are included in the article/**Suplementary Material**. Further inquiries can be directed to the corresponding author/s. The study only included existing published data, and therefore did not undergo review by an ethics committee.

## Supplementary material

Supplementary Data 1 Data extraction of SRs and AMSTAR ratings.

Supplementary Data 2 Data extraction of studies

Supplementary File 1 Detailed eligibility criteria, including deviations from protocol

Supplementary File 2 Search strategies for each database

Supplementary Table 1 Review characteristics

Supplementary Table 2 AMSTAR quality appraisal

Supplementary Table 3 Intervention characteristics and effects

Supplementary Table 4 Facilitators and barriers to participation

## References

[1] Schalock RL. The concept of quality of life: what we know and do not know. J Intellect Disabil Res. (2004) 48(Pt 3):203–16. 10.1111/j.1365-2788.2003.00558.x 15025663

[2] Varahra A Ahmed H Lindsay S. Exploring direct and indirect associations of exercise and sport participation with employment among individuals with disabilities: a scoping review. J Occup Rehabil. (2022) 32(1):44–54. 10.1007/s10926-021-09962-x 33956265

[3] Erkilic M. Conceptual challenges between universal design and disability in relation to the body, impairment, and the environment: where does the issue of disability stand in the philosophy of UD?/Evrensel tasarim ve engellilik iliskisinde insan, yeti eksikligi ve cevresel etmenler baglamini gozeten kavramsal zorluklar: engellilik konusu evrensel tasarim felsefesi icinde nerede durur? METU J Fac Archit. (2011) 28:181. 10.4305/METU.JFA.2011.2.9

[4] WHO. International Classification of Functioning, Disability, and Health: ICF. World Health Organization (WHO). (2001).

[5] Whiteneck G Dijkers MP. Difficult to measure constructs: conceptual and methodological issues concerning participation and environmental factors. Arch Phys Med Rehabil. (2009) 90(11 Suppl):S22–35. 10.1016/j.apmr.2009.06.009 19892071

[6] United Nations (UN) Department of Economic and Social Affairs Disability. Convention on the Rights of Persons with Disabilities (CRPD). United Nations (UN) Department of Economic and Social Affairs Disability. (2006).

[7] Gross JMS Monroe-Gulick A Nye C Davidson-Gibbs D Dedrick D. Multifaceted interventions for supporting community participation among adults with disabilities: a systematic review. Campbell Syst Rev. (2020) 16(2):e1092. 10.1002/cl2.1092 PMC8356358 37131415

[8] NDIA. National Disability Insurance Agency 2019–20 Annual Report. National Disability Insurance Agency (NDIA). (2020).

[9] APA. Diagnostic and Statistical Manual of Mental Disorders. 5th ed. Arlington, VA: American Psychiatric Association (APA) (2013).

[10] Schalock RL Luckasson RA Shogren KA Borthwick-Duffy S Bradley V Buntinx WHE The renaming of mental retardation: understanding the change to the term intellectual disability. Intellect Dev Disabil. (2007) 45(2):116–24. 10.1352/1934-9556(2007)45[116:TROMRU]2.0.CO;2 17428134

[11] NMHCCF. Unravelling Psychosocial Disability: a Position Statement by the National Mental Health Consumer and Carer Forum in Psychosocial Disability Associated with Mental Health Conditions. Canberra, Australia: National Mental Health Consumer and Carer Forum (NMHCCF) (2011).

[12] Joshi G Wozniak J Petty C Martelon MK Fried R Bolfek A Psychiatric comorbidity and functioning in a clinically referred population of adults with autism spectrum disorders: a comparative study. J Autism Dev Disord. (2013) 43(6):1314–25. 10.1007/s10803-012-1679-5 23076506

[13] Kim YS Leventhal BL Koh YJ Fombonne E Laska E Lim EC Prevalence of autism spectrum disorders in a total population sample. Am J Psychiatry. (2011) 168(9):904–12. 10.1176/appi.ajp.2011.10101532 21558103

[14] Ware NC Hopper K Tugenberg T Dickey B Fisher D. Connectedness and citizenship: redefining social integration. Psychiatr Serv. (2007) 58(4):469–74. 10.1176/ps.2007.58.4.469 17412847

[15] National Disability Services. A snapshot of community participation and centre based supports. Natl Disabil Serv (NDS). (2018. Retrieved from: https://www.nds.org.au/news/media-releases/survey-on-community-participationand-centre-based-supports-raises-questions-about-growth [Accessed: 10 July 2020].

[16] Cooke A Smith D Booth A. Beyond PICO: the SPIDER tool for qualitative evidence synthesis. Qual Health Res. (2012) 22(10):1435–43. 10.1177/1049732312452938 22829486

[17] Palmen A Didden R Lang R. A systematic review of behavioral intervention research on adaptive skill building in high-functioning young adults with autism spectrum disorder. Res Autism Spectr Disord. (2012) 6(2):602–17. 10.1016/j.rasd.2011.10.001

[18] Wallace BC Small K Brodley CE Lau J Trikalinos TA. Deploying an Interactive Machine Learning System in an Evidence-Based Practice Center: abstrackr. Miami, Florida, USA: The 2nd ACM SIGHIT International Health Informatics Symposium (2012).

[19] Giummarra MJ Lau G Gabbe BJ. Evaluation of text mining to reduce screening workload for injury-focused systematic reviews. Inj Prev. (2020) 26(1):55–60. 10.1136/injuryprev-2019-043247 31451565

[20] Fleming P McGilloway S Hernon M Furlong M O'Doherty S Keogh F Individualized funding interventions to improve health and social care outcomes for people with a disability: a mixed-methods systematic review. Campbell Syst Rev. (2019) 15(1-2):e1008. 10.4073/csr.2019.3 PMC8356501 37131462

[21] Plüddemann A Aronson JK Onakpoya I Heneghan C Mahtani KR. Redefining rapid reviews: a flexible framework for restricted systematic reviews. BMJ Evidence-Based Medicine. (2018) 23(6):201–3. 10.1136/bmjebm-2018-110990 29950313

[22] Shea BJ Reeves BC Wells G Thuku M Hamel C Moran J AMSTAR 2: a critical appraisal tool for systematic reviews that include randomised or non-randomised studies of healthcare interventions, or both. Br Med J. (2017) 358:j4008. 10.1136/bmj.j4008 28935701 PMC5833365

[23] Pieper D Antoine SL Mathes T Neugebauer EA Eikermann M. Systematic review finds overlapping reviews were not mentioned in every other overview. J Clin Epidemiol. (2014) 67(4):368–75. 10.1016/j.jclinepi.2013.11.007 24581293

[24] Morin L Franck N. Rehabilitation interventions to promote recovery from schizophrenia: a systematic review. Front Psychiatry. (2017) 8:ArtID 100. 10.3389/fpsyt.2017.00100 28659832 PMC5467004

[25] Roche B Tuck A Ware E McKenzie K. Promoting Health and Well-Being Through Social Inclusion in Toronto: a Scoping Review of Literature Reviews of Interventions to Promote Social Inclusion. Toronto, Canada: Wellesley Institute and Toronto Public Health (2019).

[26] Geretsegger M Mossler KA Bieleninik L Chen XJ Heldal TO Gold C. Music therapy for people with schizophrenia and schizophrenia-like disorders. Cochrane Database of Systematic Reviews. (2017) 5:CD004025. 10.1002/14651858.CD004025.pub4 28553702 PMC6481900

[27] Ratti V Hassiotis A Crabtree J Deb S Gallagher P Unwin G. The effectiveness of person-centred planning for people with intellectual disabilities: a systematic review. Res Dev Disabil. (2016) 57:63–84. 10.1016/j.ridd.2016.06.015 27394053

[28] Robertson J Emerson E Hatton C Elliott J McIntosh B Swift P Longitudinal analysis of the impact and cost of person-centered planning for people with intellectual disabilities in England. Am J Ment Retard. (2006) 111(6):400–16. 10.1352/0895-8017(2006)111[400:LAOTIA]2.0.CO;2 17029498

[29] Parley FF. Person-Centred outcomes: are outcomes improved where a person-centred care model is used? J Learn Disabil. (2001) 5(4):299–308. 10.1177/146900470100500402

[30] Newlin M Webber M Morris D Howarth S. Social participation interventions for adults with mental health problems: a review and narrative synthesis. Soc Work Res. (2015) 39(3):167–80. 10.1093/swr/svv015

[31] Chu C-I Liu C-Y Sun C-T Lin J. The effect of animal-assisted activity on inpatients with schizophrenia. J Psychosoc Nurs Ment Health Serv. (2009) 47(12):42–8. 10.3928/02793695-20091103-96 20000282

[32] Friedlie L Themessl-huber M Butchart M. Evaluation of dundee equally well sources of support: social prescribing in Maryfield. (2012).

[33] Harris T Brown GW Robinson R. Befriending as an intervention for chronic depression among women in an inner city: 1: randomised controlled trial. Br J Psychiatry. (1999) 174(3):219–24. 10.1192/bjp.174.3.219 10448446

[34] Davidson L Shahar G Stayner DA Chinman MJ Rakfeldt J Tebes JK. Supported socialization for people with psychiatric disabilities: lessons from a randomized controlled trial. J Community Psychol. (2004) 32(4):453–77. 10.1002/jcop.20013

[35] Kaplan K Salzer MS Solomon P Brusilovskiy E Cousounis P. Internet peer support for individuals with psychiatric disabilities: a randomized controlled trial. Soc Sci Med. (2011) 72(1):54–62. 10.1016/j.socscimed.2010.09.037 21112682

[36] Rivera JJ Sullivan AM Valenti SS. Adding consumer-providers to intensive case management: does it improve outcome? Psychiatr Serv. (2007) 58(6):802–9. 10.1176/ps.2007.58.6.802 17535940

[37] Walker G Bryant W. Peer support in adult mental health services: a metasynthesis of qualitative findings. Psychiatr Rehabil J. (2013) 36(1):28–34. 10.1037/h0094744 23477647

[38] Bigby C Wilson NJ Balandin S Stancliffe RJ. Disconnected expectations: staff, family, and supported employee perspectives about retirement. J Intellect Dev Disabil. (2011) 36(3):167–74. 10.3109/13668250.2011.598852 21843031

[39] Stancliffe RJ Bigby C Balandin S Wilson NJ Craig D. Transition to retirement and participation in mainstream community groups using active mentoring: a feasibility and outcomes evaluation with a matched comparison group. J Intellect Disabil Res. (2015) 59(8):703–18. 10.1111/jir.12174 25496307

[40] Bigby C Wilson NJ Stancliffe RJ Balandin S Craig D Gambin N. An effective program design to support older workers with intellectual disability to participate individually in community groups. J Policy Pract Intellect Disabil. (2014) 11(2):117–27. 10.1111/jppi.12080

[41] Wilson NJ Bigby C Stancliffe RJ Balandin S Craig D Anderson K. Mentors’ experiences of using the active mentoring model to support older adults with intellectual disability to participate in community groups. J Intellect Dev Disabil. (2013) 38(4):344–55. 10.3109/13668250.2013.837155 24279787

[42] Williams E Dingle GA Clift S. A systematic review of mental health and wellbeing outcomes of group singing for adults with a mental health condition. Eur J Public Health. (2018) 28(6):1035–42. 10.1093/eurpub/cky115 29982515

[43] Holloway P. Surviving suicide: the book of life and death: pete holloway. In: Dokter D Pete Holloway P Henri S, editors. Dramatherapy and destructiveness. Routledge (2012). p. 166–84.

[44] Orkibi H Bar N Eliakim I. The effect of drama-based group therapy on aspects of mental illness stigma. Arts Psychother. (2014) 41(5):458–66. 10.1016/j.aip.2014.08.006

[45] Foloştină R Tudorache L Michel T Erzsébet B Duţă N. Using drama therapy and storytelling in developing social competences in adults with intellectual disabilities of residential centers. Procedia Soc Behav Sci. (2015) 186:1268–74. 10.1016/j.sbspro.2015.04.141

[46] Gardner-Hynd N. Dramatherapy, learning disabilities and acute mental health. In: Jones P, editor. Drama as Therapy Volume 2: Clinical Work and Research into Practice. Routledge (2010). p. 192–208.

[47] Lahad M. The use of drama therapy with crisis intervention groups, following mass evacuation. Arts Psychother. (1999) 26(1):27–33. 10.1016/S0197-4556(98)00045-8

[48] Jaaniste J. A new beginning–A dramatherapy group for participants with co-occurring mental illness and substance abuse in a mental health setting. Dramatherapy. (2008) 30(2):17–22. 10.1080/02630672.2008.9689747

[49] McAlister M. From transitional object to symbol: spiderman in a dramatherapy group with mentally disordered offenders. In: Ditty Dokter D Pete Holloway P Henri Seebohm H, editors. Dramatherapy and destructiveness. London: Routledge (2011) p. 145–56.

[50] Dent-Brown K Wang M. The mechanism of storymaking: a grounded theory study of the 6-part story method. Arts Psychother. (2006) 33(4):316–30. 10.1016/j.aip.2006.04.002

[51] Hackett S Bourne J. The get going group: dramatherapy with adults who have learning disabilities and mental health difficulties. Dramatherapy. (2014) 36(1):43–50. 10.1080/02630672.2014.909981

[52] Grainger R. Dramatherapy and thought-disorder. Dramatherapy. (1992) 2(3):164.

[53] Darragh JA Ellison CJ Rillotta F Bellon M Crocker R. Exploring the impact of an arts-based, day options program for young adults with intellectual disabilities. Res Pract Intellect Dev Disabil. (2016) 3(1):22–31. 10.1080/23297018.2015.1075416

[54] Allan J Barford H Horwood F Stevens J Tanti G. ATIC: developing a recovery-based art therapy practice. Int J Art Ther. (2015) 20(1):14–27. 10.1080/17454832.2014.968597

[55] Bungay H Clift S. Arts on prescription: a review of practice in the U.K. Perspect Public Health. (2010) 130(6):277–81. 10.1177/1757913910384050 21213564

[56] Firth J Rosenbaum S Stubbs B Gorczynski P Yung AR Vancampfort D. Motivating factors and barriers towards exercise in severe mental illness: a systematic review and meta-analysis. Psychol Med. (2016) 46(14):2869–81. 10.1017/S0033291716001732 27502153 PMC5080671

[57] Quirk H Crank H Harrop D Hock E Copeland R. Understanding the experience of initiating community-based physical activity and social support by people with serious mental illness: a systematic review using a meta-ethnographic approach. Syst Rev. (2017) 6(1):214. 10.1186/s13643-017-0596-2 29070081 PMC5655959

[58] Soundy A Freeman P Stubbs B Probst M Coffee P Vancampfort D. The transcending benefits of physical activity for individuals with schizophrenia: a systematic review and meta-ethnography. Psychiatry Res. (2014) 220(1-2):11–9. 10.1016/j.psychres.2014.07.083 25149128

[59] Scheewe TW Backx FJG Takken T Jörg F van Strater ACP Kroes AG Exercise therapy improves mental and physical health in schizophrenia: a randomised controlled trial. Acta Psychiatr Scand. (2013) 127(6):464–73. 10.1111/acps.12029 23106093

[60] Scheewe TW Takken TIM Kahn RS Cahn W Backx FJG. Effects of exercise therapy on cardiorespiratory fitness in patients with schizophrenia. Med Sci Sports Exerc. (2012) 44(10):1834–42. 10.1249/MSS.0b013e318258e120 22525773

[61] Scheewe TW van Haren NEM Sarkisyan G Schnack HG Brouwer RM de Glint M Exercise therapy, cardiorespiratory fitness and their effect on brain volumes: a randomised controlled trial in patients with schizophrenia and healthy controls. Eur Neuropsychopharmacol. (2013) 23(7):675–85. 10.1016/j.euroneuro.2012.08.008 22981376

[62] Battaglia G Alesi M Inguglia M Roccella M Caramazza G Bellafiore M Soccer practice as an add-on treatment in the management of individuals with a diagnosis of schizophrenia. Neuropsychiatr Dis Treat. (2013) 9:595–603. 10.2147/NDT.S44066 23662058 PMC3647379

[63] Marzolini S Jensen B Melville P. Feasibility and effects of a group-based resistance and aerobic exercise program for individuals with severe schizophrenia: a multidisciplinary approach. Ment Health Phys Act. (2009) 2(1):29–36. 10.1016/j.mhpa.2008.11.001

[64] Harada CM Siperstein GN Parker RC Lenox D. Promoting social inclusion for people with intellectual disabilities through sport: special olympics international, global sport initiatives and strategies. Sport Soc. (2011) 14(9):1131–48. 10.1080/17430437.2011.614770

[65] Wilhite B Kleiber DA. The effect of special olympics participation on community integration. Ther Recreation J. (1992) 26(4):9–20.

[66] Heller T Hsieh K Rimmer JH. Attitudinal and psychosocial outcomes of a fitness and health education program on adults with down syndrome. Am J Ment Retard. (2004) 109(2):175–85. 10.1352/0895-8017(2004)109<175:AAPOOA>2.0.CO;2 15000672

[67] Glidden LM Bamberger KT Draheim AR Kersh J. Parent and athlete perceptions of special olympics participation: utility and danger of proxy responding. Intellect Dev Disabil. (2011) 49(1):37–45. 10.1352/1934-9556-49.1.37 21338311

[68] Pilling S Bebbington P Kuipers E Garety P Geddes J Martindale B Psychological treatments in schizophrenia: iI. Meta-analyses of randomized controlled trials of social skills training and cognitive remediation. Psychol Med. (2002) 32(5):783–91. 10.1017/S0033291702005640 12171373

[69] Elis O Caponigro JM Kring AM. Psychosocial treatments for negative symptoms in schizophrenia: current practices and future directions. Clin Psychol Rev. (2013) 33(8):914–28. 10.1016/j.cpr.2013.07.001 23988452 PMC4092118

[70] Glynn SM Marder SR Liberman RP Blair K Wirshing WC Wirshing DA Supplementing clinic-based skills training with manual-based community support sessions: effects on social adjustment of patients with schizophrenia. Am J Psychiatry. (2002) 159(5):829–37. 10.1176/appi.ajp.159.5.829 11986138

[71] Elgie S Maguire N. Intensive interaction with a woman with multiple and profound disabilities: a case study. Tizard Learn Disabil Rev. (2001) 6(3):18–24. 10.1108/13595474200100024

[72] Zeedyk MS Caldwell P Davies CE. How rapidly does intensive interaction promote social engagement for adults with profound learning disabilities? Eur J Spec Needs Educ. (2009) 24(2):119–37. 10.1080/08856250902793545

[73] Zeedyk MS Davies C Parry S Caldwell P. Fostering social engagement in Romanian children with communicative impairments: the experiences of newly trained practitioners of intensive interaction. Br J Learn Disabil. (2009) 37(3):186–96. 10.1111/j.1468-3156.2009.00545.x

[74] Leaning B Watson T. From the inside looking out–an intensive interaction group for people with profound and multiple learning disabilities. Br J Learn Disabil. (2006) 34(2):103–9. 10.1111/j.1468-3156.2005.00374.x

[75] Samuel J Nind M Volans A Scriven I. An evaluation of intensive interaction in community living settings for adults with profound intellectual disabilities. J Intellect Disabil. (2008) 12(2):111–26. 10.1177/1744629508090983 18492714

[76] Interian A Kline A Perlick D Dixon L Feder A Weiner MD Randomized controlled trial of a brief internet-based intervention for families of veterans with posttraumatic stress disorder. J Rehabil Res Dev. (2016) 53(5):629–40. 10.1682/JRRD.2014.10.0257 27898154

[77] Farrell C Bryant W. Voluntary work for adults with mental health problems: an exploration of the perspectives of recruiters. Br J Occup Ther. (2009) 72(5):188–96. 10.1177/030802260907200502

[78] Dukes E McGuire BE. Enhancing capacity to make sexuality-related decisions in people with an intellectual disability. J Intellect Disabil Res. (2009) 53(8):727–34. 10.1111/j.1365-2788.2009.01186.x 19527433

[79] Cunningham A Sperry L Brady MP Peluso PR Pauletti RE. The effects of a romantic relationship treatment option for adults with autism spectrum disorder. Couns Outcome Res Eval. (2016) 7(2):99–110. 10.1177/2150137816668561

[80] Dekker LP van der Vegt EJM Visser K Tick N Boudesteijn F Verhulst FC Improving psychosexual knowledge in adolescents with autism Spectrum disorder: pilot of the tackling teenage training program. J Autism Dev Disord. (2015) 45(6):1532–40. 10.1007/s10803-014-2301-9 25399394

[81] Mengue-Topio H Courbois Y Farran EK Sockeel P. Route learning and shortcut performance in adults with intellectual disability: a study with virtual environments. Res Dev Disabil. (2011) 32(1):345–52. 10.1016/j.ridd.2010.10.014 21084172

[82] Purser HRM Farran EK Courbois Y Lemahieu A Sockeel P Mellier D The development of route learning in down syndrome, williams syndrome and typical development: investigations with virtual environments. Dev Sci. (2015) 18(4):599–613. 10.1111/desc.12236 25284087

[83] Mechling LC Seid NH. Use of a hand-held personal digital assistant (PDA) to self-prompt pedestrian travel by young adults with moderate intellectual disabilities. Educ Train Autism Dev Disabil. (2011) 46:220–37.

[84] McMahon D Cihak DF Wright R. Augmented reality as a navigation tool to employment opportunities for postsecondary education students with intellectual disabilities and autism. J Res Technol Educ. (2015) 47(3):157–72. 10.1080/15391523.2015.1047698

[85] Lowe K Felce D Blackman D. Challenging behaviour: the effectiveness of specialist support teams. J Intellect Disabil Res. (1996) 40(4):336–47. 10.1111/j.1365-2788.1996.tb00639.x 8884589

[86] Gardiner T Braddon E. A right to know’. Facilitating a relationship and sexuality programme for adults with intellectual disabilities in donegal. Br J Learn Disabil. (2009) 37(4):327–9. 10.1111/j.1468-3156.2009.00591.x

[87] Hagner D Helm DT Butterworth J. This is your meeting": a qualitative study of person-centered planning. Ment Retard. (1996) 34(3):159. 8684284

[88] Carr EG Levin L McConnachie G Carlson JI Kemp DC Smith CE Comprehensive multisituational intervention for problem behavior in the community: long-term maintenance and social validation. J Posit Behav Interv. (1999) 1(1):5–25. 10.1177/109830079900100103

[89] McConnell D Dalziel A Llewellyn G Laidlaw K Hindmarsh G. Strengthening the social relationships of mothers with learning difficulties. Br J Learn Disabil. (2009) 37(1):66–75. 10.1111/j.1468-3156.2008.00526.x

[90] Ashman R Banks K Philip RCM Walley R Stanfield AC. A pilot randomised controlled trial of a group based social skills intervention for adults with autism spectrum disorder. Res Autism Spectr Disord. (2017) 43-44:67–75. 10.1016/j.rasd.2017.08.001

[91] Feldman MA Case L. Teaching child-care and safety skills to parents with intellectual disabilities through self-learning. J Intellect Dev Disabil. (1999) 24(1):27–44. 10.1080/13668259900033861

[92] Feldman MA Ducharme JM Case L. Using self-instructional pictorial manuals to teach child-care skills to mothers with intellectual disabilities. Behav Modif. (1999) 23(3):480–97. 10.1177/0145445599233007 10467893

[93] Garwood M McCabe MP. Impact of sex education programs on sexual knowledge and feelings of men with a mild intellectual disability. Educ Train Mental Retard Dev Disabil. (2000) 35(3):269–83.

[94] Iconaru EI Ciucurel C. Developing social and civic competencies in people with intellectual disabilities from a family center through an adapted training module. Procedia Soc Behav Sci. (2014) 116:3303–7. 10.1016/j.sbspro.2014.01.752

[95] Davies DK Stock SE King LR Brown RB Wehmeyer ML Shogren KA. An interface to support independent use of Facebook by people with intellectual disability. Intellect Dev Disabil. (2015) 53(1):30–41. 10.1352/1934-9556-53.1.30 25633380

[96] Courbois Y Farran EK Lemahieu A Blades M Mengue-Topio H Sockeel P. Wayfinding behaviour in down syndrome: a study with virtual environments. Res Dev Disabil. (2013) 34(5):1825–31. 10.1016/j.ridd.2013.02.023 23528440

[97] Davies DK Stock SE Holloway S Wehmeyer ML. Evaluating a GPS-based transportation device to support independent bus travel by people with intellectual disability. Intellect Dev Disabil. (2010) 48(6):454–63. 10.1352/1934-9556-48.6.454 21166550

[98] Gantman A Kapp SK Orenski K Laugeson EA. Social skills training for young adults with high-functioning autism spectrum disorders: a randomized controlled pilot study. J Autism Dev Disord. (2012) 42(6):1094–103. 10.1007/s10803-011-1350-6 21915740

[99] Walsh E Holloway J Lydon H. An evaluation of a social skills intervention for adults with autism Spectrum disorder and intellectual disabilities preparing for employment in Ireland: a pilot study. J Autism Dev Disord. (2018) 48(5):1727–41. 10.1007/s10803-017-3441-5 29224188

[100] Craig D Bigby C. “She's been involved in everything as far as I can see”: supporting the active participation of people with intellectual disability in community groups.J Appl Res Intellect Disabil. (2015) 40(1):12–25. 10.3109/13668250.2014.977235

[101] Bigby C Anderson S Cameron N. Identifying conceptualizations and theories of change embedded in interventions to facilitate community participation for people with intellectual disability: a scoping review. J Appl Res Intellect Disabil. (2018) 31(2):165–80. 10.1111/jar.12390 28799696

[102] Farrell RJ Crocker PRE McDonough MH Sedgwick WA. The driving force: motivation in special olympians. Adapt Phys Activ Q. (2004) 21(2):153–66. 10.1123/apaq.21.2.153

[103] Goodwin DL Fitzpatrick DA Thurmeier R Hall C. The decision to join special olympics: parents? Perspectives. Adapt Phys Activ Q. (2006) 23(2):163–83. 10.1123/apaq.23.2.163

[104] Jantz KM. Support groups for adults with asperger syndrome. Focus Autism Other Dev Disabil. (2011) 26(2):119–28. 10.1177/1088357611406903

[105] van Schijndel-Speet M Evenhuis HM van Wijck R van Empelen P Echteld MA. Facilitators and barriers to physical activity as perceived by older adults with intellectual disability. Intellect Dev Disabil. (2014) 52(3):175–86. 10.1352/1934-9556-52.3.175 24937743

[106] Melville CA Mitchell F Stalker K Matthews L McConnachie A Murray HM Effectiveness of a walking programme to support adults with intellectual disabilities to increase physical activity: walk well cluster-randomised controlled trial. IntJ Behav Nutr Phys Activ. (2015) 12(1):1–11. 10.1186/s12966-015-0290-5 PMC4587575 26416606

[107] Matthews L Mitchell F Stalker K McConnachie A Murray H Melling C Process evaluation of the walk well study: a cluster-randomised controlled trial of a community based walking programme for adults with intellectual disabilities. BMC public health. (2016) 16(1):1–11. 10.1186/s12889-016-3179-6 27387203 PMC4936049

[108] Proudfoot J Parker G Manicavasagar V Hadzi-Pavlovic D Whitton A Nicholas J Effects of adjunctive peer support on perceptions of illness control and understanding in an online psychoeducation program for bipolar disorder: a randomised controlled trial. J Affect Disord. (2012) 142(1-3):98–105. 10.1016/j.jad.2012.04.007 22858215

[109] Mildon R Wade C Matthews J. Considering the contextual fit of an intervention for families headed by parents with an intellectual disability: an exploratory study. J Appl Res Intellect Disabil. (2008) 21(4):377–87. 10.1111/j.1468-3148.2008.00451.x

[110] Treece A Gregory S Ayres B Mendis K. ‘I always do what they tell me to do': choice-making opportunities in the lives of two older persons with severe learning difficulties living in a community setting. Disabil Soc. (1999) 14(6):791–804. 10.1080/09687599925894

[111] Heslop P. Good practice in befriending services for people with learning difficulties. Br J Learn Disabil. (2005) 33(1):27–33. 10.1111/j.1468-3156.2004.00310.x

[112] Hillier A Fish T Cloppert P Beversdorf DQ. Outcomes of a social and vocational skills support group for adolescents and young adults on the autism Spectrum. Focus Autism Other Dev Disabil. (2007) 22(2):107–15. 10.1177/10883576070220020201

[113] Kurtz MM Mueser KT. A meta-analysis of controlled research on social skills training for schizophrenia. J Consult Clin Psychol. (2008) 76(3):491–504. 10.1037/0022-006X.76.3.491 18540742

[114] Mueser KT Penn DL. Pilling and colleagues (2002) recently published a meta-analysis examining the effects of social skills training on schizophrenia (this review also included a meta-analysis of research on cognitive remediation for schizophrenia which is not discussed in this comment). Psychol Med. (2004) 34(7):1365–7. 10.1017/S0033291704213848 15697062

[115] Pfammatter M Junghan UM Brenner HD. Efficacy of psychological therapy in schizophrenia: conclusions from meta-analyses. Schizophr Bull. (2006) 32(suppl_1):S64–80. 10.1093/schbul/sbl030 16905634 PMC2632545

[116] Egemo-Helm KR Miltenberger RG Knudson P Finstrom N Jostad C Johnson B. An evaluation of in situ training to teach sexual abuse prevention skills to women with mental retardation. Behav Interv. (2007) 22(2):99–119. 10.1002/bin.234

[117] Brisson NA. Parent Training and its Effect on Attunement of Mothers with Intellectual Disabilities. Union Institute & University (2009).

[118] Heller T Miller AB Hsieh K Sterns H. Later-life planning: promoting knowledge of options and choice-making. Ment Retard. (2000) 38(5):395–406. 10.1352/0047-6765(2000)038<0395:LPPKOO>2.0.CO;2 11060981

[119] Frey GC Buchanan AM Rosser Sandt DD. “I'd rather watch TV”: an examination of physical activity in adults with mental retardation. Ment Retard. (2005) 43(4):241–54. 10.1352/0047-6765(2005)43[241:IRWTAE]2.0.CO;2 16000025

[120] Hamilton J Stevens G Girdler S. Becoming a mentor: the impact of training and the experience of mentoring university students on the autism Spectrum. PLOS ONE. (2016) 11(4):e0153204. 10.1371/journal.pone.0153204 27070418 PMC4829264

[121] Roberts N Birmingham E. Mentoring university students with ASD: a mentee-centered approach. J Autism Dev Disord. (2017) 47(4):1038–50. 10.1007/s10803-016-2997-9 28132120

[122] Curtin C Humphrey K Vronsky K Mattern K Nicastro S Perrin EC. Expanding horizons: a pilot mentoring program linking college/graduate students and teens with ASD. Clin Pediatr (Phila). (2015) 55(2):150–6. 10.1177/0009922815588821 26016838 PMC4662633

[123] Hotez E Shane-Simpson C Obeid R DeNigris D Siller M Costikas C Designing a summer transition program for incoming and current college students on the autism Spectrum: a participatory approach. Front Psychol. (2018) 9:46. 10.3389/fpsyg.2018.00046 29487547 PMC5816926

[124] Brandling J House W Howitt D Sansom A. “New routes”: Pilot research project of a new social prescribing service provided in Keynsham. (2011).

[125] Moffatt S Steer M Lawson S Penn L O’Brien N. Link worker social prescribing to improve health and well-being for people with long-term conditions: qualitative study of service user perceptions. BMJ Open. (2017) 7(7):e015203. 10.1136/bmjopen-2016-015203 28713072 PMC5541496

[126] McClimens A Gordon F. People with intellectual disabilities as bloggers: what's Social capital got to do with it anyway? J Intellect Disabil. (2009) 13(1):19–30. 10.1177/1744629509104486 19332506

[127] Lante KA Walkley JW Gamble M Vassos MV. An initial evaluation of a long-term, sustainable, integrated community-based physical activity program for adults with intellectual disability. J Intellect Dev Disabil. (2011) 36(3):197–206. 10.3109/13668250.2011.593163 21843034

[128] Harada CM Siperstein GN. The sport experience of athletes with intellectual disabilities: a national survey of special olympics athletes and their families. Adapt Phys Activ Q. (2009) 26(1):68–85. 10.1123/apaq.26.1.68 19246774

[129] ERS Research Consultancy. Newcastle social prescribing project. Final Report. (2013).

[130] Tedrick T. Growing older in special olympics: meaning and benefits of participation—selected case studies. Act Adapt Aging. (2009) 33(3):137–60. 10.1080/01924780903148169

[131] Espiner D Hartnett FM. “I felt I was in control of the meeting”: facilitating planning with adults with an intellectual disability. Br J Learn Disabil. (2012) 40(1):62–70. 10.1111/j.1468-3156.2011.00684.x

[132] Siette J Cassidy M Priebe S. Effectiveness of befriending interventions: a systematic review and meta-analysis. BMJ Open. (2017) 7(4):e014304. 10.1136/bmjopen-2016-014304 28446525 PMC5594212

[133] Shogren K Wehmeyer M Martinis J Blanck P. Social-Ecological models of disability. In: Martinis J Shogren KA Wehmeyer ML Blanck P, editors. Supported decision-making: theory, research, and practice to enhance self-determination and quality of life. Cambridge disability law and policy series. Cambridge: Cambridge University Press (2018). p. 29–45.

[134] Bota A Teodorescu S Şerbănoiu S. Unified sports – A social inclusion factor in school communities for young people with intellectual disabilities. Procedia Soc Behav Sci. (2014) 117:21–6. 10.1016/j.sbspro.2014.02.172

[135] NICE. Autism spectrum Disorder in Adults. London: National Institute for Health and Care Excellence (NICE) (2016). 31869047

[136] Commonwealth of Australia. A national framework for recovery-oriented mental health services: A Guide for practitioners and providers. (2013).

[137] NICE. Psychosis and schizophrenia in adults: treatment and management. NICE Clinical guideline 178. National Institute for Health and Care Excellence (NICE) (2014). 25340235

[138] Ringeisen H Langer Ellison M Ryder-Burge A Biebel K Alikhan S Jones E. Supported education for individuals with psychiatric disabilities: state of the practice and policy implications. Psychiatr Rehabil J. (2017) 40(2):197–206. 10.1037/prj0000233 28182470

[139] Miller E Stanhope V Restrepo-Toro M Tondora J. Person-centered planning in mental health: a transatlantic collaboration to tackle implementation barriers. Am J Psychiatr Rehabil. (2017) 20(3):251–67. 10.1080/15487768.2017.1338045 31632212 PMC6800658

[140] Morin KL Ganz JB Gregori EV Foster MJ Gerow SL Genç-Tosun D A systematic quality review of high-tech AAC interventions as an evidence-based practice. Augment Altern Commun. (2018) 34(2):104–17. 10.1080/07434618.2018.1458900 29697288

[141] Carnemolla P Robinson S Lay K. Towards inclusive cities and social sustainability: a scoping review of initiatives to support the inclusion of people with intellectual disability in civic and social activities. City Cult Soc. (2021) 25:100398. 10.1016/j.ccs.2021.100398

[142] Doroud N Fossey E Fortune T. Place for being, doing, becoming and belonging: a meta-synthesis exploring the role of place in mental health recovery. Health Place. (2018) 52:110–20. 10.1016/j.healthplace.2018.05.008 29885554

[143] Howlin P Yates P. The potential effectiveness of social skills groups for adults with autism. Autism. (1999) 3(3):299–307. 10.1177/1362361399003003007

[144] Ouellette L Horner RH Stephen Newton J. Changing activity patterns to improve social networks: a descriptive analysis. Behav Interv. (1994) 9(1):55–66. 10.1002/bin.2360090106

