# Supplementary material for: Interventions for social and community participation for adults with intellectual disability, psychosocial disability or on the autism spectrum: An umbrella systematic review
Source: Front Rehabil Sci. 2022 Aug 19;3:935473. doi: 10.3389/fresc.2022.935473 (PMC9397886; doi:10.3389/fresc.2022.935473)
Supplement: Supplementary File 1 [file Table_3_v1.docx]

**Supplementary File 1**

Detailed eligibility criteria from the review protocol and deviations to protocol

**Screening**

**Deviation to protocol**: In addition to the literature databases and grey literature search, PROSPERO was manually searched to identify registered protocols of systematic reviews. Full text articles for any that met the inclusion criteria were retrieved for eligibility screening.

**Inclusion criteria**

*Sample*

Studies were eligible if they included:

- Adult participants aged ≥18 years of age. Studies that include adolescents will be eligible if ≥50% of participants are aged ≥18 years of age.
- Participants who had left secondary education or if the intervention is not linked to their status as a student or delivered within a secondary education setting.
- A participant sample in which ≥50% had a diagnosis of either autism spectrum disorder, intellectual disability or psychosocial disability, and outcomes were reported for these groups separately from other disability groups. Studies of learning disabilities were only included if ≥50% of participants had a diagnosed intellectual disability.

Studies were excluded if they evaluated interventions provided to people with acquired intellectual or cognitive disabilities that do not correspond to the AAIDD definition of intellectual disability.

*Phenomena of Interest / Interventions*

Intervention/s reported in each study could targeted at the individual, group or broader population, and had to meet the following criteria:

- The intervention sought to affect at least one personal characteristic or resource, or an environmental factor in order to improve SCC participation;
- The intervention aimed to change one or more SCC participation outcome/s;
- The intervention was provided to people living in the community and/or in a community-based setting;
- The entire sample (or a subsection of the entire sample, such as in a randomised controlled trial) received the intervention and outcomes are reported specific to that sample.

As the NDIS does not provide services that substitute for those supplied appropriately by the health system (NDIS Act, 2016), this review will not include studies evaluating interventions delivered through the health system, or that exclusively include treatment of symptoms, and only measure changes in symptoms (e.g., depression) but do not measure impacts of the intervention or treatment on SCC participation or related outcomes (e.g., social cognition, self-efficacy). Studies that evaluated the impacts of large-scale moves of PWD from institutional residential locations to the living in the community on SCC were excluded as these are no longer relevant to the social model of disability that underpins the disability support sector in Australia.

**Clarifications added to protocol for determining eligibility**:

1. However, interventions delivered primarily by allied health practitioners that could feasibly be delivered by social and community services to enhance SCC participation or capacity for SCC participation were eligible.
2. Interventions focused on physical activity or exercise were only eligible if they comprised a social or community element in the intervention design (e.g., group setting with peers or delivered in a community setting) or if they measured SCC participation outcomes. Physical activity interventions that only measure impacts on health outcomes, or psychological characteristics, such as anxiety, depression or self-efficacy, did not meet these criteria. Interventions were ineligible if they focused on a personal characteristic not directly within the scope of social or community skills, behaviour or participation, including those targeting another psychological function (e.g., cognitive functioning) or symptom management (e.g., anxiety, obsessive compulsive disorder, psychosis, depression symptoms), consistent with previous reviews; Palmen, 2012).

*Design*

Studies could have used any theoretical framework or study design to develop, implement or evaluate SCC interventions. In accordance with the GRADE of evidence (see section 8.0), the narrative synthesis will emphasise findings from randomised controlled trials (RCTs), quasi‐experimental and controlled before and after studies, well-designed cohort studies in which participant groups are comparable, and trustworthy qualitative studies that seek to understand the experience or acceptability of interventions, including facilitators and barriers to intervention success. All included studies must report quantitative or qualitative data regarding outcomes or the effects of the intervention, which could show a lack of effect, negative effect or positive effect on SCC participation.

*Evaluation and outcomes*

Studies must measure a change in SCC participation (e.g., before and after receiving the intervention, and/or that measure differences in participation between groups who did/did not receive the intervention) or SCC capacity (e.g., self-efficacy, social cognition). Qualitative studies that explore the experience, acceptability, barriers or facilitators of SCC-focused interventions will also be eligible.

A broad range of primary outcomes were included if they characterised *direct access to or participation in social, community or civic activities*, or SCC *participation capacity* (e.g., social cognition, self-efficacy), including but not limited to:

- **Social participation** (e.g., communication, social relationship maintenance, participation through telecommunications or online platforms, convivial encounters)
- **Social networking** (e.g., friendships, relationships – boy/girlfriend or spouse, church or other faith related attendance)
- **Navigating/accessing the community** (e.g., accessing public or private transport)
- **Recreation, sports and leisure activity participation in the community** (e.g., sports, art, music, community or cultural events, libraries, tourism)
- **Continued learning** (e.g., engaging in college or technical training – classes or diploma or certificate completion)
- **Civic involvement** (e.g., voting, volunteer work, advocacy, committees/leadership club/organisational membership, political engagement, activism, voting in elections or referendums)

While SCC may be considered to include housing in the ICF framework (e.g., independent living in a home of choice, usability of the home, housemates/co-residents) these were not included in the present review given that home and living options will be reviewed in a separate commissioned systematic review described in Section 1.2.

We also identified whether and how interventions impacted on **secondary outcomes** as *dimensions of community participation*, such as:

- Increased self-determination (e.g., autonomy, choice, decision-making, self-advocacy)
- Improved physical or mental health
- Improved quality of life
- Increased family support/activities in the home (e.g., caregiving, supporting children/parenting, household chores/care)

*Research Type and publication limits*

Systematic reviews, scoping reviews, qualitative, quantitative, mixed and multi-method research was eligible for inclusion. Publications were eligible if they were published in English between 2010-present to capture contemporary empirical evidence. Systematic reviews published within this timeframe were included to provide evidence from historical literature. Publications were eligible if they were peer reviewed, dissertations/theses, or evaluations of policy, programs and interventions in the grey literature.

**Deviation from protocol**: As scoping reviews typically use systematic methods to identify and synthesise literature these were added to the eligible research types for the umbrella review. Publications were considered to be an eligible systematic review if they:

1. includes a clear statement of the purpose of the review;
2. described the search strategy, including naming two or more databases that were included, search terms, and inclusion/exclusion criteria;
3. presents all findings relevant to the main purpose of the review, including those that did not favour the intervention; and

**Deviation to protocol:** While evidence from all case studies included in the systematic reviews were eligible, the additional primary studies were excluded if they used a descriptive case study method rather than a rigorous case study method (e.g., multiple baseline).

**Deviation to protocol:** For supplementary review, publications reporting individual study findings were included for data extraction if they were not already included in the eligible systematic or scoping reviews.

Publications were excluded if they were editorials, letters, conference abstracts, organisational website content or publicity materials from disability services due to potential conflicts of interest.

**Deviation to protocol**: books and book chapters were also considered to be ineligible.

**Data extraction**

**Deviation from protocol**. Due to staffing annual leave timing, the data extraction forms were not piloted by two reviewers prior to data extraction.
