# Supplementary material for: Interventions for social and community participation for adults with intellectual disability, psychosocial disability or on the autism spectrum: An umbrella systematic review
Source: Front Rehabil Sci. 2022 Aug 19;3:935473. doi: 10.3389/fresc.2022.935473 (PMC9397886; doi:10.3389/fresc.2022.935473)
Supplement: Supplementary File 2 [file Table_5_v1.docx]

1. **Search strategy in OVID for the following databases**

- **Ovid MEDLINE(R) and Epub Ahead of Print, In-Process & Other Non-Indexed Citations, Daily and Versions(R)**1946 to December 01, 2020
- **Ovid Emcare**1995 to 2020 Week 47
- **APA PsycInfo**1806 to November Week 4 2020

**The search was executed for each database separately, then duplicates were removed.**

| **Category** | **Step** | **OVID Search terms - to enter into OVID** |
| --- | --- | --- |
| ASD | 1 | Autism Spectrum Disorder/ or Autistic Disorder/ |
| ASD | 2 | ((Autis* or Asperg*) adj1 (disord* or disab*)) |
| ID | 3 | Intellectual Disability/ |
| ID | 4 | Developmental Disabilities/ |
| ID | 5 | (Intellectual disability* or Mental* retard* or Cognitive* impair*) |
| ID | 6 | Learning disabilities/ |
| PD | 7 | (disab* or handicap* or impair*) |
| PD | 8 | Mentally Ill Persons/ |
| PD | 9 | Mental Disorders/ |
| PD | 10 | Mentally Disabled Persons/ |
| PD | 11 | ((mental* or psych*) adj1 (abnormal* or ailment* or condition* or deficien* or derange* or disab* or disease* or disorder* or handicap* or ill* or infirm* or impair* or malad* or problem* or sick* or syndrome* or patholog*)) |
| INELIGIBLE DISABILITIES | 12 | exp dementia/ |
| INELIGIBLE DISABILITIES | 13 | (exp Immunologic Disorders/) or (exp Multiple Sclerosis) or (exp Parkinson's Disease/) |
| INELIGIBLE DISABILITIES | 14 | (exp HIV/) or (exp AIDS/) |
| COMMUNITY | 15 | Community Integration/ |
| COMMUNITY | 16 | social participation/ |
| COMMUNITY | 17 | ((access* or navigat* or Usability) adj2 (service* or facilities or resources or activit* or advice or community or environment)) |
| COMMUNITY | 18 | (sense adj2 (belonging or community)) |
| COMMUNITY | 19 | ((psychosocial or social or community or civic) adj2 (access* or activ* or capital or cohesion or contact or engagement or functioning or group or inclu* or interact* or intervention or integrat* or involve* or isolation or life or navigate* or network or participat* or prescribe* or program* or rehabilitat* or service* or skills or support or ties)) |
| COMMS | 20 | assistive device/ |
| COMMS | 21 | Social media/ |
| COMMS | 22 | Blogging/ |
| COMMS | 23 | Communication aids for disabled/ |
| ARTS/THEATRE | 24 | ((cultur* or music* or orchestra or Art or arts) adj3 (event or activit* or ceremony* or participat* or concert*)) |
| BUILT ENVIRONMENT | 25 | environment design/ or Built Environment/ |
| BUILT ENVIRONMENT | 26 | information centers/ or libraries/ |
| BUILT ENVIRONMENT | 27 | garden/ |
| BUILT ENVIRONMENT | 28 | public facilities/ |
| BUILT ENVIRONMENT | 29 | restaurants/ |
| BUILT ENVIRONMENT | 30 | toilet facilities/ |
| BUILT ENVIRONMENT | 31 | (worship* or (relig* adj participat*) or church or mosque or (temple not (head or injur* or surg*)) or synagogue or chapel) |
| BUILT ENVIRONMENT | 32 | (community adj (hub* or centre or center or event*)) |
| BUILT ENVIRONMENT | 33 | (public adj (place* or space*)) |
| BUILT ENVIRONMENT | 34 | (park* or (play ground*) or playground* or sport field* or recreation area* or (public adj (ground or park*)) or outdoor* or garden* or beach* or mountain*) |
| LEISURE/RECREATION | 35 | recreation therapy/ |
| LEISURE/RECREATION | 36 | exp sports/ or recreational facilities/ |
| LEISURE/RECREATION | 37 | exp leisure activities/ |
| LEISURE/RECREATION | 38 | exp transportation facilities/ |
| LEISURE/RECREATION | 39 | air travel/ |
| LEISURE/RECREATION | 40 | expeditions/ |
| LEISURE/RECREATION | 41 | tourism/ |
| LEISURE/RECREATION | 42 | (leisure* or (recreation not (inj* or vehicle*)) or sport* or exercise or (physical* adj activ*) or exercise therapy) |
| LEISURE/RECREATION | 43 | (mobility or transport or train or tram or taxi or bus or scooter or ((air or sea) and travel) or cruis* or ship or boat airplane*) |
| POLITICAL/CIVIC | 44 | exp stakeholder participation/ |
| POLITICAL/CIVIC | 45 | political activism/ |
| POLITICAL/CIVIC | 46 | ((Politic* or civi* or election) adj3 (participat* or engage* or inclu*or interven* or integrat* or involve*)) |
| POLITICAL/CIVIC | 47 | ((election adj3 voter) or voting) |
| SOCIAL/RELATIONSHIPS | 48 | social environment/ |
| SOCIAL/RELATIONSHIPS | 49 | community networks/ |
| SOCIAL/RELATIONSHIPS | 50 | social support/ |
| SOCIAL/RELATIONSHIPS | 51 | public assistance/ |
| SOCIAL/RELATIONSHIPS | 52 | interpersonal relations/ |
| SOCIAL/RELATIONSHIPS | 53 | social integration/ |
| SOCIAL/RELATIONSHIPS | 54 | intergenerational relations/ |
| SOCIAL/RELATIONSHIPS | 55 | social isolation/ or loneliness/ |
| SOCIAL/RELATIONSHIPS | 56 | (advisor*or befriend* or boyfriend or broker* buddy or buddies or coach* or co-resident* or friend* or girlfriend or housemate* or intimate partner or mentor* or spouse) |
| SOCIAL/RELATIONSHIPS | 57 | (peer adj2 (training or mediat* or advoca* or support or advis*)) |
| SOCIAL/RELATIONSHIPS | 58 | interpersonal interaction* |
| SOCIAL/RELATIONSHIPS | 59 | ((family or formal or informal or interpersonal or intimate) adj2 relationship*) |
| SOCIAL/RELATIONSHIPS | 60 | (sex* adj1 (support or participa* or activity)) |
| SOCIAL/RELATIONSHIPS | 61 | mentoring/ |
| SOCIAL/RELATIONSHIPS | 62 | friends/ |
| SOCIAL/RELATIONSHIPS | 63 | grandparents/ |
| SOCIAL/RELATIONSHIPS | 64 | legal guardians/ |
| SOCIAL/RELATIONSHIPS | 65 | mentors/ |
| SOCIAL/RELATIONSHIPS | 66 | parents/ |
| SOCIAL/RELATIONSHIPS | 67 | spouses/ |
| SOCIAL/RELATIONSHIPS | 68 | sexual partners/ |
| SOCIAL/RELATIONSHIPS | 69 | sex workers/ |
| SOCIAL/RELATIONSHIPS | 70 | (loneliness or lonely) |
| EDUCATION | 71 | exp education, nonprofessional/ |
| EDUCATION | 72 | (continu* adj learning) |
| EDUCATION | 73 | (vocational adj (independent* or engage* or train* or program*)) |
| INTERVENTION/EVALUATION | 74 | exp clinical trial/ |
| INTERVENTION/EVALUATION | 75 | (RCT or intervention or evaluat* or assess* or trial or repeated measure* or between group* or modulat* or effect* or (clinical adj trial)) |
| AUTISM | 76 | Or/1-2 |
| ID | 77 | Or/3-6 |
| PD | 78 | (Or/8-11) and 7 |
| INELIGIBLE DISABILITIES | 79 | Or/12-14 |
| **ANY DISABILITY AND EVALUATION** | 80 | ((76 or 77 or 78) and (74 or 75)) not 79 |
| COMMUNITY | 81 | OR/15-19 |
| COMMS | 82 | Or/20-23 |
| ARTS/THEATRE | 83 | 24 |
| BUILT ENVIRONMENT | 84 | OR/25-34 |
| LEISURE/RECREATION | 85 | OR/35-43 |
| POLITICAL/CIVIC | 86 | Or/44-47 |
| SOCIAL/RELATIONSHIPS | 87 | Or/48-70 |
| EDUCATION | 88 | Or/71-73 |
| **ANY PARTICIPATION** | 89 | Or/81-88 |
| **DISABILITY AND PARTICIPATION AND EVALUATION** | 90 | 80 and 89 |
| LIMITS | 91 | limit 90 to english language |
| LIMITS | 92 | limit 91 to human |
| LIMITS | 93 | limit 92 to humans |
| LIMITS | 94 | limit 93 to yr="1986 -Current" |
| LIMITS | 95 | Limit 94 to ("all adult (19 plus years)" or "young adult (19 to 24 years)" or "adult (19 to 44 years)" or "young adult and adult (19-24 and 19-44)" or "middle age (45 to 64 years)" or "middle aged (45 plus years)" or "all aged (65 and over)" or "aged (80 and over)") |
| LIMITS | 96 | limit 95 to yr="2000 -Current" |
| LIMITS | 97 | limit 95 to yr="2010 -Current" |

1. **ProQuest search strategy for ASSIA and ERIC**

**Notes:** Searches need to be executed separately for ASSIA, but they all use the same search formatting (i.e., noft = anywhere except full text).

**ASSIA**

- **Additional limits:**
  - **Source type:** Dissertations & Theses OR Scholarly Journals
  - **Language**: English
  - **Exclude:**
    - Duplicate documents
  - **Don’t include:**
    - Spelling variants for your search terms
    - Form variants for search terms

**ERIC**

- **Additional limits:**
  - Date: After 31 December 1986
  - Document type: 080: Journal Articles OR Article OR 143: Reports - Research OR 142: Reports - Evaluative OR Dissertation/Thesis OR 041: Dissertations/Theses - Doctoral Dissertations OR 040: Dissertations/Theses OR Review
  - Language: English

**Policy File – dropped due to low number of citations that were all irrelevant**

- **Additional limits**:
  - Date: After 31 December 1986;
  - Language: English

**Enter search terms below into command line**

noft(Autism Spectrum Disorder) or noft(Autistic Disorder) or ((noft(Autis*) or noft(Asperg*)) N/1 (noft(disord*) or noft(disab*)))) OR (noft(Intellectual Disability) or noft(Developmental Disab*) or noft(Intellectual disab*) or noft(Mental* retard*) or noft(mental* handicap*) or (noft(Learn*) N/1 noft(disab*)) or ((**(**noft(mental*) or noft(psych*)**)** N/1 (noft(abnormal*) or noft(ailment*) or noft(condition*) or noft(deficien*) or noft(derange*) or noft(disab*) or noft(disease*) or noft(disorder*) or noft(handicap*) or noft(health) or noft(ill*) or noft(infirm*) or noft(impair*) or noft(malad*) or noft(problem*) or noft(sick*) or noft(syndrome*) or noft(patholog*))) AND (noft(disab*))) OR ((noft(Mentally Ill Person*) or noft(Mental Disorder*) or noft(Mentally Disabled Person*) or noft(Psychiatric disorder*)))

AND

(noft(Community Integration) OR noft(social participation) OR ((noft(access*) OR noft(navigat*) OR noft(Usability)) NEAR/2 (noft(service*) OR noft(facilities) OR noft(resources) OR noft(activit*) OR noft(advice) OR noft(community) OR noft(environment))) OR (noft(sense) NEAR/2 (noft(belonging) OR noft(community))) OR ((noft(psychosocial) OR noft(social) OR noft(community) OR noft(civic)) NEAR/2 (noft(access*) OR noft(activ*) OR noft(capital) OR noft(cohesion) OR noft(contact) OR noft(engagement) OR noft(functioning) OR noft(group) OR noft(inclu*) OR noft(interact*) OR noft(intervention) OR noft(integrat*) OR noft(involve*) OR noft(isolation) OR noft(life) OR noft(navigate*) OR noft(network) OR noft(participat*) OR noft(prescribe*) OR noft(program*) OR noft(rehabilitat*) OR noft(service*) OR noft(skills) OR noft(support) OR noft(ties)))) OR (noft(assistive device) or noft(Social media) or noft(Blog*) or noft(Communication aids)) OR (((noft(cultur*) OR noft(music*) OR noft(orchestra) OR noft(Art) OR noft(arts)) NEAR/2 (noft(event) OR noft(activit*) OR noft(ceremon*) OR noft(participat*) OR noft(concert*)))) OR (noft("environment design") OR noft("Built Environment") OR noft("libraries") OR noft("garden") OR noft("public facilities") OR noft("restaurants") OR noft("toilet facilities") OR (noft(worship*) OR (noft(relig*) n/ noft(participat*)) OR noft(church) OR noft(mosque) OR (noft(temple) NOT (noft(head) OR noft(injur*) OR noft(surg*))) OR noft(synagogue) OR noft(chapel)) OR (noft(community) NEAR/4 (noft(hub*) OR noft(centre) OR noft(center) OR noft(event*))) OR (noft(public) NEAR/4 (noft(place*) OR noft(space*))) OR (noft(park or parks) OR noft(play ground*) OR noft(playground*) OR noft(sport field*) OR noft(recreation area*) OR (noft(public) NEAR/4 (noft(ground) OR noft(park*))) OR noft(outdoor*) OR noft(garden*) OR noft(beach*) OR noft(mountain*))) OR (noft("recreation therapy") OR noft("sports") OR noft("recreational facilities") OR noft("leisure activities") OR noft("transportation facilities") OR noft("air travel") OR noft(tourism) OR (noft(leisure*) OR (noft(recreation) NOT (noft(inj*) OR noft(vehicle*))) OR noft(sport*) OR noft(exercise) OR (noft(physical*) NEAR/2 noft(activ*)) OR noft(exercise therapy)) OR (noft(mobility) OR noft(transport) OR noft(train) OR noft(tram) OR noft(taxi) OR noft(bus) OR noft(scooter) OR ((noft(air) OR noft(sea)) AND noft(travel)) OR noft(cruis*) OR noft(ship) OR noft(boat) OR noft(airplane*))) OR (noft("stakeholder participation") OR noft("political activism") OR ((noft(Politic*) OR noft(civi*) OR noft(election)) NEAR/3 (noft(participat*) OR noft(engage*) OR noft(inclu*) OR noft(interven*) OR noft(integrat*) OR noft(involve*))) OR ((noft(election) NEAR/3 noft(voter)) OR noft(voting))) OR (noft("social environment") OR noft("community networks") OR noft("social support") OR noft("public assistance") OR noft("interpersonal relations") OR noft("social integration") OR noft("intergenerational relations") OR noft(sexual partner*) OR noft(sex worker*) OR noft("social isolation") OR noft("loneliness") OR (noft(advisor*) OR noft(befriend*) OR noft(boyfriend) OR noft(broker*) OR noft(buddy) OR noft(buddies) OR noft(coach*) OR noft(co-resident*) OR noft(friend*) OR noft(grandparent*) OR noft(parent*) OR noft(legal guardian*) OR noft(girlfriend) OR noft(housemate*) OR noft(intimate partner) OR noft(mentor*) OR noft(spouse)) OR (noft(peer) NEAR/2 (noft(training) OR noft(mediat*) OR noft(advoca*) OR noft(support) OR noft(advis*))) OR (noft("interpersonal interaction") OR noft("interpersonal interactions")) OR ((noft(family) OR noft(formal) OR noft(informal) OR noft(interpersonal) OR noft(intimate)) NEAR/2 noft(relationship*)) OR (noft(sex*) NEAR/2 (noft(support) OR noft(participa*) OR noft(activity)))) OR ((noft(education) NEAR/4 noft(nonprofessional)) OR (noft(continu*) NEAR/4 noft(learning)) OR (noft(vocational) NEAR/2 (noft(independent*) OR noft(engage*) OR noft(train*) OR noft(program*))))

AND

noft(clinical trial) OR noft(RCT) OR noft(intervention) OR noft(evaluat*) OR noft(assess*) OR noft(trial) OR noft(repeated measure*) OR noft(between group*) OR noft(modulat*) OR noft(effect*)

NOT

noft(dement*) OR noft(Alzheimer*) OR noft(HIV) OR noft(Acquired immunodeficiency syndrome) OR noft(autoimmune disease) OR noft(autoimmune disease*) OR (noft(parkinson*) NEAR/1 noft(disord*)) OR noft("Demyelinating Autoimmune Diseases") OR noft(encephalomyel*) OR noft(immune system disease*) OR noft(multiple sclerosis) OR ((noft(family) or noft(interperson*)) near/1 noft(violen*)) or noft(paediatric) OR noft(deinstitut*) OR noft(eating disorder*)

1. **Search strategy for Cumulative Index of Nursing and Allied Health Literature (CINAHL) via Ebscohost**

- S19 (limits only by English language, publication year, and publication type): 9,377 citations
- S21 (limits also by age: all adult): 4,117

| **Search number** | **Search terms** |
| --- | --- |
|  | **Disability** |
|  | (Autism Spectrum Disorder) |
| S1 | (MH(Autism Spectrum Disorder) or MH(Autistic Disorder) or ((Autis* or Asperg*) N/1 (disord* or disab*))) |
|  | OR (Intellectual disability) |
| S2 | (MH(intellectual disability) or (Intellectual Disab*) or mh(Developmental Disabilities) or (Developmental Disab*) or (Mental* retard*) or (Cognitive impair*) or (mental* disab*) or (mental* handicap*) or (learning disab*) |
|  | OR (Psychosocial Disability) |
| S3 | ((mh(Mentally Ill Persons) or mh(Mental Disorders) or mh(Mentally Disabled Persons) or (Psychiatric disorder*) or (psychological disab*) or (psychiatric disab*) or (psychosocial disab*)) and (disab* or handicap* or impair*)) or (((mental* or psych*) N1 (abnormal* or ailment* or condition* or deficien* or derange* or disab* or disease* or disorder* or handicap* or health or ill* or infirm* or impair* or malad* or problem* or sick* or syndrome* or patholog* or wellbeing)) and (disab* or handicap* or impair*)) |
|  | NOT (ineligible conditions for SR) |
| S4 | (mh(dementia) or dementia OR alzheim* OR noft(HIV) OR noft(Acquired immunodeficiency syndrome) OR mh(Autoimmune Diseases of the Nervous System ) or mh(Parkinsonian Disorders) or (autoimmune disease*) OR ((autoimmune disease*) N4 "Nervous System") OR ((parkinson*) N1 disord*) OR (Demyelinating Autoimmune Disease*) OR (encephalomyel*) OR ("immune system disease*") OR (multiple sclerosis)) ((family or interperson*) N1 violen*) or paediatric OR deinstitut* OR eating disorder* |
|  | **AND (any participation terms)** |
|  | (community/social) |
| S5 | (mh(Community Integration) OR mh(social participation) OR ((access* OR navigat* OR Usability) N2 (service* OR facilities or facility OR resource* OR activit* OR advice OR community OR environment)) OR (sense N3 (belong* OR community)) OR ((psychosocial OR social OR community OR civic) N3 (access* OR activ* OR capital OR cohesion OR contact OR engagement OR function* OR group OR inclu* OR interact* OR intervention OR integrat* OR involve* OR isolation OR life OR navigate* OR network OR participat* OR prescribe* OR program* OR rehabilitat* OR service* OR skills OR support OR ties))) |
|  | OR (communication) |
| S6 | (mh(assistive device) or mh(Social media) or (Blog*) or (Communication aid*) or mh(Communication aids for disabled)) |
|  | OR (arts/theatre etc.) |
| S7 | ((cultur* OR music* OR orchestra OR Art OR arts) N3 (event* OR activit* OR ceremony OR ceremonies OR participat* OR concert*)) |
|  | OR (built environment and public places) |
| S8 | (mh(environment design) OR mh(Built Environment) OR mh(libraries) OR mh(garden) OR garden* OR mh(public facilities) OR mh(toilet facilities) OR mh(restaurants) OR worship* OR (relig* participat*) OR church OR mosque OR park* OR (play ground*) OR playground* OR (sport field*) OR (recreation area*) OR (public N2 (ground* OR park*)) OR synagogue OR chapel OR outdoor* OR beach* OR mountain* OR (temple NOT (head OR injur* OR surg*)) OR (community N4 (hub* OR centre OR center OR event*)) OR (public AND (place* OR space*))) |
|  | OR (leisure or recreation therapy) |
| S9 | (mh(recreation therapy) OR mh(sports) OR mh(recreational facilities) OR mh(leisure activities) OR mh(transportation facilities) OR mh(air travel) OR (sea travel) OR mh(tourism) OR leisure* OR (recreation NOT (inj* OR vehicle*)) OR sport* OR exercise OR (physical* N2 activ*) OR (exercise therapy) OR mobility OR transport OR train OR tram OR taxi OR bus OR scooter OR cruis* OR ship OR boat OR airplane*) |
|  | OR (political and civic participation) |
| S10 | (mh(stakeholder participation) OR mh(political activism) OR ((Politic* OR civi* OR election) N3 (participat* OR engage* OR inclu* OR interven* OR integrat* OR involve*)) OR (election N3 (voter OR voting))) |
|  | OR (social relationships) |
| S11 | (mh(social environment) OR mh(community networks) OR mh(social support) OR mh(public assistance) OR mh(interpersonal relations) OR mh(social integration) OR mh(intergenerational relations) OR mh(social isolation) OR mh(loneliness) OR advisor* OR befriend* OR boyfriend OR broker* or buddy OR buddies OR coach* OR co-resident* OR friend* OR girlfriend OR housemate* OR (intimate partner) OR mentor* OR spouse OR mh(sexual partners) or mh(sex workers) OR parent* or grandparent* OR (interpersonal interaction) OR (interpersonal interactions) OR (peer N2 (training OR mediat* OR advoca* OR support OR advis*)) OR ((family OR formal OR informal OR interpersonal OR intimate) N2 relationship*) OR (sex* N4 (support OR participa* OR activity OR relationship))) |
|  | OR (education) |
| S12 | (mh(education, nonprofessional) OR (education N4 (vocation* or nonprofessional)) OR (continu* N3 (learning or educ*)) OR (vocational N2 (independent* OR engage* OR train* OR program*))) |
|  | **AND (intervention/evaluation terms)** |
| S13 | (mh(clinical trial) OR RCT OR intervention OR evaluat* OR assess* OR trial OR (repeated measure*) OR (between group*) OR (modulat*) OR (effect*)) |
| COMBINING SEARCH GROUPS | |
| DISABILITY | |
| S14 | S1 OR S2 OR S3 |
| S15 | S14 NOT S4 |
| PARTICIPATION (Including education) | |
| S16 | S5 OR S6 OR S7 OR S8 OR S9 OR S10 OR S11 OR S12 |
| DISABILITY AND PARTICIPATION | |
| S17 | S15 AND S16 AND S13 |
| LIMITS (manually select) | |
| S18 | Publication Year: 1986-2021  Academic Journals and Dissertations |
| S19 | Narrow by Language: - English |
| S20 | Age: All adult |

1. **Search strategy for The Campbell Collaboration and Cochrane library**

**The Campbell Collaboration**

- Only 195 Review records currently on the website, including:
  - Disability, Social welfare and Knowledge translation and implementation groups
  - Keyword: “Disability”
- Hand check all of these reviews for inclusion/eligibility screening
- Results: https://campbellcollaboration.org/component/jak2filter/?Itemid=1352&issearch=1&isc=1&category_id=101&ordering=publishUp

**Cochrane Library**

**Note: MeSH term formatting** [mh “TERM PHRASE”] includes mesh term WITH explosion and [mh ^“TERM PHRASE”] includes mesh term WITHOUT explosion

| **Category** | **Step** | COCHRANE search terms - to enter into search engine |
| --- | --- | --- |
| ASD | 1 | [mh ^"Autistic DIsorder"] or [mh ^"autism spectrum disorder"] |
| ASD | 2 | ((Autis* or Asperg*) near/1 (disord* or disab*)) |
| ID | 3 | [mh "intellectual disability"] |
| ID | 4 | [mh ^"Developmental Disabilities"] |
| ID | 5 | (Intellectual disability* or Mental* retard* or Cognitive* impair*) |
| ID | 6 | [mh ^"Learning disabilities"] |
| PD | 7 | (disab* or handicap* or impair*) |
| PD | 8 | [mh ^"Mentally Ill Persons"] |
| PD | 9 | [mh ^"Mental Disorders"] |
| PD | 10 | [mh ^"Mentally Disabled Persons"] |
| PD | 11 | ((mental* or psych*) adj2 (abnormal* or ailment* or condition* or deficien* or derange* or disab* or disease* or disorder* or handicap* or ill* or infirm* or impair* malad* or problem* or sick* or syndrome* or patholog*)) |
| INELIGIBLE DISABILITIES | 12 | [mh dementia] |
| INELIGIBLE DISABILITIES | 13 | [mh "Autoimmune Diseases of the Nervous System"] or [mh "Immunologic Disorders"] or [mh "Multiple Sclerosis"] or [mh HIV] or [mh AIDS] |
| INELIGIBLE DISABILITIES | 14 | [mh "Parkinsonian Disorders"] |
| COMMUNITY | 15 | [mh ^"Community Integration"] |
| COMMUNITY | 16 | [mh ^"social participation"] |
| COMMUNITY | 17 | ((access* or navigat* or Usability) adj2 (service* or facilities or resources or activit* or advice or community or environment)) |
| COMMUNITY | 18 | (sense adj2 (belonging or community)) |
| COMMUNITY | 19 | ((psychosocial or social or community or civic) adj2 (access* or activ* or capital or cohesion or contact or engagement or functioning or group or inclu* or interact* or intervention or integrat* or involve* or isolation or life or navigate* or network or participat* or prescribe* or program* or rehabilitat* or service* or skills or support or ties)) |
| COMMS | 20 | [mh ^"assistive device"] |
| COMMS | 21 | [mh ^"Social media"] |
| COMMS | 22 | [mh ^Blogging] |
| COMMS | 23 | [mh ^"Communication aids for disabled"] |
| ARTS/THEATRE | 24 | ((cultur* or music* or orchestra or Art or arts) near/3 (event or activit* or ceremony* or participat* or concert*)) |
| BUILT ENVIRONMENT | 25 | [mh ^"environment design"] or [mh ^"Built Environment"] |
| BUILT ENVIRONMENT | 26 | [mh ^"information centers"] or [mh ^libraries] |
| BUILT ENVIRONMENT | 27 | [mh ^garden] |
| BUILT ENVIRONMENT | 28 | [mh ^"public facilities"] |
| BUILT ENVIRONMENT | 29 | [mh ^restaurants] |
| BUILT ENVIRONMENT | 30 | [mh ^"toilet facilities"] |
| BUILT ENVIRONMENT | 31 | (worship* or (relig* near/1 participat*) or church or mosque or (temple not (head or injur* or surg*)) or synagogue or chapel) |
| BUILT ENVIRONMENT | 32 | (community adj (hub* or centre or center or event*)) |
| BUILT ENVIRONMENT | 33 | (public near/1 (place* or space*)) |
| BUILT ENVIRONMENT | 34 | (park* or play ground* or playground* or sport field* or recreation area* or (public near/1 (ground or park*)) or outdoor* or garden* or beach* or mountain*) |
| LEISURE/RECREATION | 35 | [mh ^"recreation therapy"] |
| LEISURE/RECREATION | 36 | [mh sports] or [mh ^"recreational facilities"] |
| LEISURE/RECREATION | 37 | [mh "leisure activities"] |
| LEISURE/RECREATION | 38 | [mh "transportation facilities"] |
| LEISURE/RECREATION | 39 | [mh ^"air travel"] |
| LEISURE/RECREATION | 40 | [mh ^expeditions] |
| LEISURE/RECREATION | 41 | [mh ^tourism] |
| LEISURE/RECREATION | 42 | (leisure* or (recreation not (inj* or vehicle*)) or sport* or exercise or (physical* near/1 activ*) or exercise therapy) |
| LEISURE/RECREATION | 43 | (mobility or transport or train or tram or taxi or bus or scooter or ((air or sea) and travel) or cruis* or ship or boat airplane*) |
| POLITICAL/CIVIC | 44 | [mh "stakeholder participation"] |
| POLITICAL/CIVIC | 45 | [mh ^"political activism"] |
| POLITICAL/CIVIC | 46 | ((Politic* or civi* or election) near/3 (participat* or engage* or inclu*or interven* or integrat* or involve*)) |
| POLITICAL/CIVIC | 47 | ((election near/3 voter) or voting) |
| SOCIAL/RELATIONSHIPS | 48 | [mh ^"social environment"] |
| SOCIAL/RELATIONSHIPS | 49 | [mh ^"community networks"] |
| SOCIAL/RELATIONSHIPS | 50 | [mh ^"social support"] |
| SOCIAL/RELATIONSHIPS | 51 | [mh ^"public assistance"] |
| SOCIAL/RELATIONSHIPS | 52 | [mh ^"interpersonal relations"] |
| SOCIAL/RELATIONSHIPS | 53 | [mh ^"social integration"] |
| SOCIAL/RELATIONSHIPS | 54 | [mh ^"intergenerational relations"] |
| SOCIAL/RELATIONSHIPS | 55 | [mh ^"social isolation"] or [mh ^loneliness] |
| SOCIAL/RELATIONSHIPS | 56 | (advisor*or befriend* or boyfriend or broker* buddy or buddies or coach* or co-resident* or friend* or girlfriend or housemate* or intimate partner or mentor* or spouse) |
| SOCIAL/RELATIONSHIPS | 57 | (peer near/2 (training or mediat* or advoca* or support or advis*)) |
| SOCIAL/RELATIONSHIPS | 58 | interpersonal interaction* |
| SOCIAL/RELATIONSHIPS | 59 | ((family or formal or informal or interpersonal or intimate) near/2 relationship*) |
| SOCIAL/RELATIONSHIPS | 60 | (sex* near/1 (support or participa* or activity)) |
| SOCIAL/RELATIONSHIPS | 61 | [mh ^mentoring] |
| SOCIAL/RELATIONSHIPS | 62 | [mh ^friends] |
| SOCIAL/RELATIONSHIPS | 63 | [mh ^grandparents] |
| SOCIAL/RELATIONSHIPS | 64 | [mh ^"legal guardians"] |
| SOCIAL/RELATIONSHIPS | 65 | [mh ^mentors] |
| SOCIAL/RELATIONSHIPS | 66 | [mh ^parents] |
| SOCIAL/RELATIONSHIPS | 67 | [mh ^spouses] |
| SOCIAL/RELATIONSHIPS | 68 | [mh ^"sexual partners"] |
| SOCIAL/RELATIONSHIPS | 69 | [mh ^"sex workers"] |
| SOCIAL/RELATIONSHIPS | 70 | (loneliness or lonely) |
| EDUCATION | 71 | [mh "education, nonprofessional"] |
| EDUCATION | 72 | (continu* near/1 learning) |
| EDUCATION | 73 | (vocational near/1 (independent* or engage* or train* or program*)) |
| INTERVENTION/EVALUATION | 74 | [mh "clinical trial"] |
| INTERVENTION/EVALUATION | 75 | (RCT or intervention or evaluat* or assess* or trial or repeated measure* or between group* or modulat* or effect*) |
| AUTISM | 76 | {OR #1-#2} |
| ID | 77 | {Or #3-#6} |
| PD | 78 | {Or #8-#11} AND #7 |
| INELIGIBLE DISABILITIES | 79 | {Or #12-#14} |
| **ANY DISABILITY AND EVALUATION** | 80 | {Or #76-#78} AND {Or #74-#75} NOT #79 |
| COMMUNITY | 81 | {Or #15-#19} |
| COMMS | 82 | {Or #20-#23} |
| ARTS/THEATRE | 83 | #24 |
| BUILT ENVIRONMENT | 84 | {Or #25-#34} |
| LEISURE/RECREATION | 85 | {Or #35-#43} |
| POLITICAL/CIVIC | 86 | {Or #44-#47} |
| SOCIAL/RELATIONSHIPS | 87 | {Or #48-#70} |
| EDUCATION | 88 | {Or #71-#73} |
| **ANY PARTICIPATION** | 89 | {Or #81-#88} |
| **DISABILITY AND PARTICIPATION AND EVALUATION** | 90 | {AND #80, *#*89} |
| LIMITS | 91 | #90 with Publication Year from 1986 to 2020, with Cochrane Library publication date from Jan 1986 to Dec 2020, in Trials |
