# Supplementary material for: Interventions for social and community participation for adults with intellectual disability, psychosocial disability or on the autism spectrum: An umbrella systematic review
Source: Front Rehabil Sci. 2022 Aug 19;3:935473. doi: 10.3389/fresc.2022.935473 (PMC9397886; doi:10.3389/fresc.2022.935473)
Supplement: Supplementary Table 1 [file Table_4_v1.docx]

## **Supplementary Table 1**. Systematic review characteristics

|  | Included studies | | | | | Heterogeneity | | Intervention effects* | | Review Quality |
| --- | --- | --- | --- | --- | --- | --- | --- | --- | --- | --- |
| Review First author (year)^ref^ | Sample* | Phenomena of interest (Interventions) | Design and Evaluation  *Eligible study period* | Capacity, Participation and Environment Outcomes | Overall study quality | Sample | Methods | C | P | AMSTAR** |
| Anderson (2015)^1^ | 5 studies (100%) N=633 (68.9% male) PSD | Interventions to improve the social networks | Systematic review   - 5 RCTs - Inception to 2014 | **P:** Social network size | H | No | Yes | n/a | + | 57.7 |
| Atkinson-Jones (2019)^2^ | 10 studies (100%)  N=216 (80.6% male) ASD and ID | Social skills interventions | Systematic review   - 4 RCTs, 1 NRCT-CG, 5 NRCT-noCGs - nr to 2017 | **C:** Theory of mind; empathy; emotion recognition and control; social skills and knowledge  **P:** Peer relationships; social functioning; frequency of social engagement; loneliness | nr | Yes | Yes | + | + | 15.4 |
| Bigby (2012)^3^ | 8 studies (57%)  N=69 (21.7% male) ID | Interventions for social inclusion (e.g., positive behavioural support, intensive intervention, personal care planning). | Systematic review   - 2 NRCT-noCGs, 2 descriptive, 4 case study - Inception to 2010 | **P:** Social relationships; community activity participation; time in the community and interactions in public; social participation; communication | nr | No | Yes | n/a | + | 26.9 |
| Bigby (2018)^4^ | 13 studies (100%), N=188 (sex nr) ID | Interventions or programs for community participation (community groups, sports groups), belonging, convivial encounters and leisure participation (art, physical activity) | Scoping review   - 1 descriptive, 8 qualitative, 4 mixed or multi-methods - 2000-2015 | **C:** Self-esteem  **P:** acceptance from group members; social network size; social contacts; leisure and mainstream activity participation; belonging in society; blogging participation. | L | Yes | Yes | + | + | 46.2 |
| Bondár (2020)^5^ | 8 studies (66.7%)  N=419 (sex nr) ID | Physical activity and sport-based interventions | Systematic review   - 5 RCTs, 2 NRCT-noCGs, 2 case study, 1 qualitative - Eligible dates nr | **P:** Community integration; supports to participate in exercise; social support; enjoyment; adaptive behaviours | L | Yes | Yes | n/a | + | 61.5 |
| Bourne (2018)^6^ | 12 studies (100%)  N=196 (sex nr) ID & PSD | Drama therapy group work | Meta-ethnography   - 1 descriptive, 2 case study, 5 qualitative, 4 mixed or multi-methods - Inception to 2017 | **C:** Social competency | nr | Yes | Yes | nr | n/a | 42.3 |
| Bundock (2017)^7^ | 5 studies (100%)  N=46 (69.6% male) ASD & ID | Social skills interventions | Systematic review   - 2 NRCT-noCGs, 3 case study - nr to 2015 | **C:** emotion control; correct communication behaviours; aberrant behaviour; work personality profile | L | Yes | Yes | + | n/a | 42.3 |
| Clatworthy (2013)^8^ | 7 studies (70%)  N= 200 (75.7% male) PSD | Gardening-based interventions | Systematic Review  2 RCT, 4 NRCT-No CG, 1 Multi-methods  2003 to 2011 | **C:** social skills  **P:** social activity level, “social benefits”, “social factors”, sense of belonging, interpersonal relationships, social behaviour | nr | No | Yes | + | + | 3.85 |
| Coren (2011)^9-11^ | 3 studies (100%)  N=107 (4.7% male) ID | Parenting training for parents with ID | Cochrane review   - 3 RCTs - Inception to 2009 | **C:** Daily child care routines; sensitivity and responsiveness to child cues and distress; parenting knowledge | L | No | Yes | + | n/a | 84.6 |
| Exell (2020)^12^ | 5 studies (46%)  N=137 (62.0% male) ASD & ID | Interventions for dating/romantic relationships | Systematic review   - 3 RCTs, 1 NRCT-noCG, 1 case study - Inception to 2020 | **C:** Social skills; avoidance and distress; social competence; autism symptoms; interpersonal violence  **P:** social networks; frequency of opposite sex interactions | L | No | Yes | + | + | 38.5 |
| Fernandez-Sotos (2020)^13^ | 6 studies (86%)  N=191 (sex nr), ASD & PSD | VR-based interventions for psychosocial remediation | Systematic review   - 2 RCTs, 3 NRCT-noCGs, 1 case study - 2000 to 2019 | **C:** Social behaviour; assertiveness; social problem solving; relationship change, social interaction skills; emotion recognition; social cognition and perception; theory of mind, empathy  **P:** social network size | nr | No | No | + | nr | 19.2 |
| Finkel (2020)^14^ | 6 studies (6%)  N=160 (sex nr) PSD | Arts-focused therapy | Scoping review   - Study design nr - Inception to 2019 | **P**: sense of community belonging; intention to join a similar group in the community; social connections formed | nr | Yes | Yes | n/a | + | 42.3 |
| Firth (2015)^15^ | 11 studies (65%)  N=550 (sex nr) PSD | Physical activity participation | Systematic review   - 8 RCTs, 2 NRCT-CGs, 1 NRCT-noCG - 1994 to 2013 | **P:** Psychosocial functioning | M | No | Yes | n/a | o/+ | 40.6 |
| Firth (2016)^16^ | 12 studies (100%)  N=6,181 (sex nr) PSD | Physical activity participation | Systematic review   - Study design nr - nr to 2016 | **P:** Motivators and barriers to physical activity participation | nr | Yes | Yes | n/a | n/a | 34.4 |
| Fortuna (2020)^17^ | 8 studies (27%)  N=959 (40.7% male) PSD | Digital peer support interventions | Systematic Review  5 RCT, 1 NRCT, 1 NRCT-NoCG 1 qualitative  1946 2018 | **P**: role functioning, friendship, loneliness/ isolation, social functioning, perceived alienation, social support | L | No | No | n/a | o/+ | 73.1 |
| Fuhr (2014)^18^ | 5 studies (36%)  N=1,447 (sex nr) PSD | Peer interventions | Meta-analysis  5 RCT  Inception to 2012 | **P**: family and social contacts, social network, social isolation, social functioning, loneliness, environmental quality of life | H | No | No | n/a | o | 68.8 |
| Genter (2015)^19^ | 3 studies (30%)  N=29 (sex nr) PSD | Allotment gardening | Systematic review  1 multi methods, 2 Qualitative  1998 to 2015 | **P**: social interaction, friendships | L | Yes | No | n/a | + | 19.2 |
| Geretsegger (2017)^20^ | 18 studies (100%)  N=1,215 (54.6% male) PSD | Music therapy | Cochrane review   - 18 RCTs - nr to 2015 | **P**: Social functioning | nr | No | No | n/a | + | 93.8 |
| Gonzalvez (2018)^21^ | 6 studies (75.0)  N=227 (36.6% male) ID | Sex education programs | Meta-analysis   - 3 RCTs, 2 NRCT-CGs, 1 NRCT-noCG - Inception to 2017 | **C**: inappropriate behaviours; decision making; sexual abuse; social skills | nr | No | No | + | n/a | 46.9 |
| Grant (2017)^22^ | 32 studies (100%)  N=1,440 (66.0% male) PSD | Social cognition interventions | Systematic review   - 32 RCTs - nr to 2016 | **C:** Theory of mind; affect recognition; attribution style; social perception and knowledge  **P**: social engagement; interpersonal communication | L | No | Yes | + | + | 46.4 |
| Hallett (2019)^23^ | 3 studies (50%) N=93 (sex nr) ASD & ID | Physical activity participation | Systematic review   - 2 NRCT-CGs, 1 case study - nr to 2018 | **C**: maladaptive and stereotypic behaviours; communication regulation; interaction imitation; emotion  **P**: social belonging; community integration | nr | No | Yes | + | + | 11.5 |
| Howarth (2016)^24^ | 8 studies (73%)  N=417 (sex nr) ID | Health and social care interventions to enhance social and community inclusion and participation | Systematic review   - 2 NRCT-CGs, 4 NRCT-noCGs, 1 case study, 1 mixed or multi-methods - Inception to 2014 | **P**: social networks and relationships; type and frequency of contacts; participation in the community; use of community facilities; social changes | M | No | Yes | n/a | + | 38.5 |
| Hutchinson (2015)^25^ | 8 studies (50%) N=27 (37.0% male) ASD & ID | Intensive Interaction | Systematic review   - 7 case study, 1 qualitative - nr to 2013 | **C**: communication and social behaviour; social contact initiation skills | L | No | No | + | n/a | 23.1 |
| Hutzler (2010)^26^ | 9 studies (42.9)  N=1,067 (59.6% male) ID | Physical activity participation | Systematic review   - 1 RCT, 2 NRCT-CGs, 3 descriptive, 3 qualitative - 1980 to 2009 | **C:** social competence  **P**: social integration; social interaction; making new friends; social acceptance; social self-perception; social support | M | No | Yes | + | + | 11.5 |
| Kaltenthaler (2014)^27^ | 13 studies (100%)  N=1,863 (64% male) PSD | Sexual health interventions | Systematic review   - 13 RCTs - Inception to 2012 | **C**: Sex-related attitudes, knowledge, assertiveness and motivation  **P**: Sex risk behaviours | M | Yes | Yes | + | + | 76.9 |
| Kamioka (2014)^28^ | 7 studies (64%)  N=241 (sex nr) PSD | Animal-assisted therapy | Systematic review   - 7 RCTs - 1990 to 2012 | **P**: social interactions; social support and contacts; loneliness; social functioning | M | Yes | Yes | n/a | + | 73.1 |
| Lindsay (2019)^29^ | 11 studies (38%)  N=143 (25.2% male) ASD & ID | Travel training programs and interventions | Systematic review   - 1 RCT, 2 NRCT-CGs, 7 case study, 1 mixed or multi-methods - 1992 to 2017 | **C:** accuracy and capacity to navigate, use bus route; generalisation to actual bus route | L | Yes | Yes | + | n/a | 11.5 |
| Lorenc (2018)^30,31^ | 30 studies (94%)  N=653 (78.7% male) ASD | “supportive interventions” to help adults with ASD in their daily lives (e.g., social skills training, job interview training, employment support, music and dance, support and mentoring for university students) | Systematic review   - 8 RCTs, 6 NRCT-CGs, 16 NRCT-noCGs - Inception to 2016 | **C**: Adaptive behaviour; social skills; social cognition; dating skills; empathy; loneliness; socialisation quality and satisfaction  **P**: social activities; peer relations; socialisation; leisure participation needs; engagement or satisfaction | M | No | Yes | o/+ | o | 42.3 |
| Louw (2019)^32^ | 11 studies (46%)  N=240 (sex nr) ID | Interventions to enhance social inclusion | Systematic review   - 1 NRCT-noCG, 5 case study, 1 qualitative, 4 mixed or multi-methods - 2013 to 2019 | **C**: communication skills; civic rights knowledge; social skills  **P:** social interactions; generalisation of social skills; relationships formed; social media use; social belonging | H | Yes | Yes | + | + | 19.2 |
| Ma (2020)^33^ | 26 studies (87%)  N=2,357 (sex nr) PSD | Interventions to alleviate subjective social isolation (including loneliness and perceived social support) | Systematic review   - 26 RCTs - Inception to 2017 | **P:** loneliness; social support and networks; social interactions; social functioning | H | Yes | Yes | n/a | o/+ | 50.0 |
| Masi (2011)^34^ | 7 studies (14%)  N=464 (45.7% male) PSD | Interventions to alleviate loneliness. | Meta-analysis   - 3 RCTs, 2 NRCT-CGs, 2 NRCT-noCGs - 1970 to 2009 | **P:** Loneliness | nr | Yes | Yes | n/a | o | 34.4 |
| Maujean (2015)^35^ | 4 studies (57%)  N=170 (sex nr) PSD | Animal-assisted therapy | Systematic review   - 4 RCTs - 2008 to 2012 | **P:** social support, contact, relationships and behaviour | nr | Yes | Yes | nr | + | 19.2 |
| McCann (2019)^36^ | 3 studies (38%)  N=70 (sex nr) ID | Sex and relationship education | Systematic review   - 2 descriptive, 1 qualitative - 1998 to 2018 | **C**: relationship knowledge; decision making ability; capacity to consent; safety practices; self-esteem | M | No | No | + | n/a | 46.2 |
| Morin (2017)^37^ | 11 studies (14%)  N=nr PSD | psychosocial interventions (e.g., psychoeducation, cognitive remediation, social skill training, family psychoeducation, cognitive therapy) | Umbrella review   - 11 systematic reviews - 1995 to 2017 | **C**: social skills  **P**: social role functioning | L | No | No | + | + | 19.2 |
| Naslund (2015)^38^ | 6 studies (13%)  N=904 (sex nr) PSD | Mobile and e-Health interventions (most focused on symptom management/medication adherence, but some included social functioning) | Systematic review   - 3 RCTs, 1 NRCT-CG, 2 NRCT-noCGs) - nr to 2014 | **C**: Parenting skills  **P**: social interactions; social support | L | No | Yes | + | o/+ | 42.3 |
| Nguyen (2020)^39^ | 10 studies (100%)  N=213 (sex nr) ASD | Peer mentoring transition programs in college and university settings | Meta-ethnography   - 3 qualitative, 7 mixed or multi-methods - Inception to 2018 | **C**: academic and social skills  **P**: making connections and friends; forming relationships with people with and without disabilities | M | No | No | + | + | 42.3 |
| Palmen (2012)^40^ | 41 studies (100%)  N=1,021 (sex nr) ASD | Behavioural interventions for adaptive skill building | Systematic review   - 11 RCTs, 9 NRCT-CGs, 21 NRCT-noCGs - nr to 2017 | **C**: social, conversation and interpersonal skills | L | No | Yes | + | n/a | 26.9 |
| Pallathra (2019)^41^ | 9 studies (45%)  N=1,021 (75.9% male) ASD | Psychosocial interventions targeting social functioning | Systematic review   - 1 NRCT-CG, 3 NRCT-noCGs, 5 case study - 1980 to 2010 | **C:** social cognition; social skills and social anxiety. | L | No | Yes | + | n/a | 11.5 |
| Pescheny (2020)^42^ | 16 studies (100%)  N=nr PSD | Social prescriber services and programs | Systematic review   - 1 RCT, 1 NRCT-CG, 1 NRCT-noCG, 1 qualitative, 12 mixed or multi-methods - Inception to 2018 | **P:** Social interactions; isolation; loneliness; leisure activities; engagement in the community; established social links/friendships; sense of belonging; group cohesion; opportunities to learn new skills (e.g., art, singing, playing sport). | L | No | No | n/a | + | 26.9 |
| Petroutsou (2018)^43^ | 1 study (17%) N=58 (72.4% male) ID | Loneliness interventions | Systematic review   - 1 NRCT-CGs - 2000 to 2016 | **P:** loneliness; community group participation | L | No | No | n/a | o/+ | 45.8 |
| Picton (2020)^44^ | 18 xtudies (100%)  N=321 (48.3% male) PSD | Outdoor nature-based therapeutic recreation | Meta-ethnography  18 qualitative  Inception to 2019 | **P**: social contact, talking with others, relationships, making new connections, sense of belonging, appreciation and connection, social inclusion | H | No | No | n/a | + | 78.6 |
| Puolakka (2019)^45^ | 12 studies (100%)  N=1,142 (75.0% male) PSD | Psychosocial interventions (family intervention, psychoeducation, social skills training, supported employment) | Systematic review   - 12 RCTs - 1990 to 2018 | **P**: interpersonal relations | M | No | No | n/a | + | 42.3 |
| Quirk (2020)^46^ | 16 studies (100.0)  N=198 (56.1% male) PSD | Community-based group physical activity participation | Meta-ethnography   - 1 case study, 14 qualitative, 1 mixed or multi-methods - nr to 2017 | **P**: experiences of initiating community-based group-based physical activity | M | Yes | Yes | n/a | n/a | 65.6 |
| Ratti (2016)^47^ | 7 studies (44%)  N=180 (6.1% male) ASD, ID & PSD | Person-centred planning | Systematic review   - 1 RCT, 1 NRCT-CG, 1 NRCT-noCG, 1 descriptive, 3 qualitative - 1990 to 2014 | **P**: Community participation (locations attended, activities);  social networks and relationships | M | Yes | Yes | n/a | + | 15.4 |
| Roche (2019)^48^ | 4 studies (50%)  N=nr PSD | Interventions focused on social inclusion | Umbrella review   - 4 systematic reviews - 2000 to 2015 | **P**: mentoring experience; social connectedness and engagement; meaningful participation | nr | Yes | Yes | n/a | + | 19.2 |
| Sala (2019)^49^ | 24 studies (80%)  N=866 (39.1% male) ASD & ID | Sexuality and relationship education interventions | Systematic review   - 6 RCTs, 3 NRCT-CGs, 8 NRCT-noCGs, 7 case study - 1980 to 2018 | **C**: sex and relationship knowledge; social skills; safety behaviours (e.g., birth control use, sexual abuse prevention); inappropriate behaviours  **P**: social functioning | L | Yes | Yes | + | + | 30.8 |
| Schepens (2019)^50^ | 15 studies (21%)  N=576 (sex nr) ID | Support strategies to enhance quality of life in older people with ID | Systematic review   - 1 RCT, 3 NRCT-CGs, 2 NRCT-noCGs, 4 case study, 5 qualitative - 1995 to 2017 | **P**: social inclusion; interpersonal relationships; supports | M | Yes | Yes | n/a | + | 15.4 |
| Siette (2017)^51^ | 6 studies (46%)  N=381 (sex nr) PSD | Befriending interventions | Meta-analysis  6 RCT  Inception to 2017 | **C:** social functioning, loneliness, social support | M | No | No | n/a | o | 53.1 |
| Soundy (2014)^52^ | 11 studies (100%)  N=108 (sex nr) PSD | Physical activity participation | Meta-ethnography   - 11 qualitative - ?? to 2014 | **P**: experiences of participating in physical activity | H | No | Yes | n/a | + | 46.4 |
| Takahashi (2019)^53^ | 4 studies (57%)  N=221 (73.3% male) ASD & ID | Dance therapy | Systematic review   - 1 RCT, 1 NRCT-CG, 2 NRCT-noCGs - 1970 to 2018 | **C**: empathy; social mirroring/imitation; interpersonal synchrony  **P**: spontaneous interactions | L | Yes | No | + | nr | 23.1 |
| Tint (2017)^54^ | 18 studies (39%)  N=1,824 (70.7% male) ID | Special Olympics participation experiences and effects | Systematic review   - 3 NRCT-CGs, 6 descriptive, 2 case study, 7 qualitative - Inception to 2015 | **C:** social behaviour; maladaptive behaviours; communication ability  **P**: social participation; social acceptance or approval; community awareness, involvement, inclusion and access to venues; social networks; inclusive friendships | L | No | Yes | + | + | 42.3 |
| Tobin (2014)^55^ | 6 studies (46%)  N=164 (68.9% male) ASD | Interventions to support social participation | Systematic review   - 2 RCTs, 2 NRCT-noCGs, 1 qualitative, 1 mixed or multi-methods - 1995 to 2013 | **C**: social skills; empathy; conversations elicited; social utterances  **P**: peer relations; loneliness; social support | M | No | Yes | + | + | 38.5 |
| Walker (2013)^56^ | 25 studies (100%)  N=380 (sex nr) PSD | Peer support in mental health services | Meta-ethnography   - 20 qualitative, 5 mixed or multi-methods - 1990 to 2010 | **P**: social networks; confidence; self-esteem reintegration into the community; hope, motivation; rapport with others | L | Yes | Yes | n/a | + | 15.4 |
| Webber (2017)^57^ | 21 studies (95%)  N=nr PSD | Social participation interventions (individual social skills training; group skills training; supported community engagement; group-based community activities; employment interventions; and peer support interventions) | Systematic review   - 6 RCTs, other designs nr - nr to 2016 | **C**: interpersonal skills  **P**: Social relations; role functioning and activities; social networks, capital and relationships; social satisfaction; ability to get along with others; loneliness; community participation; social activities | nr | Yes | Yes | + | + | 15.4 |
| White (2020)^58^ | 11 studies (48%)  N=2,040 (sex nr) PSD | One on one peer support in mental health services | Meta-analysis  11 RCTs  Inception to 2015 | **P:** social functioning, social networks, sense of community | H | Yes | Yes | n/a | o | 50.0 |
| Williams (2018)^59^ | 11 studies (92%)  N=508 (35.0% male) PSD | Group singing in community settings | Systematic Review  1 RCT, 2 NRCT, 2 NRCT-NoCG, 1 multi-methods, 5 qualitative  2002 to 2017 | **P:** belonging, connection to community, social support/ acceptance | L | No | Yes | n/a | + | 46.2 |
| Wilson (2014)^60^ | 7 studies (100%)  N=157 (12.1% male) ID | Interventions to strengthen social relationships parenting skills for parents with ID. | Systematic review   - 1 RCT, 1 NRCT-CGs, 3 NRCT-noCGs, 2 case study - 1999 to 2010 | **C**: social skills; parenting skills, knowledge behaviour and feelings  **P**: relationship quality | L | Yes | Yes | o/+ | + | 3.9 |

*Notes*: Data were not extracted for the reviews by Newlin^61^, Spain^62^ and Pitt^63^ as they completely overlapped with other included reviews, and the review by Spain^64^ identified no eligible randomised controlled trials of family therapy for schizophrenia.

*Abbreviations*: ASD = Autism Spectrum Disorder; C = Capacity outcomes; E = environment/community-focused outcomes; H = high quality or low risk of bias; ID = Intellectual Disability; L = low quality or high risk of bias; M = moderate quality or risk of bias; n/a = not applicable; nr = not reported; P = Participation outcomes; PSD = psychosocial disability

*Symbols*: *: sample characteristics: eligible studies (percent relative to number of studies in the respective systematic review), pooled sample size, percent male, disability types; ** AMSTAR value indicates the proportion of relevant AMSTAR quality domains that the respective systematic review met; - ≥60% of studies had negative (harmful) effect; + ≥60% of studies had positive effect; o ≥60% of studies had null effect; o/+ no effect direction met the ≥60% positive, negative or null effect thresholds

## **Supplementary Table 2**. AMSTAR quality appraisal

| Systematic Review first author (year) | AMSTAR domain* | | | | | | | | | | | | | | | | Percent of relevant quality criteria met |
| --- | --- | --- | --- | --- | --- | --- | --- | --- | --- | --- | --- | --- | --- | --- | --- | --- | --- |
|  | 1 | 2 | 3 | 4 | 5 | 6 | 7 | 8 | 9 | 10 | 11 | 12 | 13 | 14 | 15 | 16 |  |
| Anderson (2015)^1^ | Y | P | N | Y | Y | N | N | Y | Y | N | n/a | n/a | Y | Y | n/a | N | 57.7 |
| Atkinson-Jones (2019)^2^ | N | N | N | Y | N | N | N | N | N | N | n/a | n/a | N | Y | n/a | N | 15.4 |
| Bigby (2018)^4^ | Y | N | N | Y | Y | N | N | Y | N | N | n/a | n/a | Y | Y | n/a | N | 46.2 |
| Bigby (2012)^3^ | N | N | N | P | N | N | N | Y | N | N | n/a | n/a | N | Y | n/a | Y | 26.9 |
| Bondár (2020)^5^ | Y | N | Y | Y | Y | N | N | Y | Y | N | n/a | n/a | Y | N | n/a | Y | 61.5 |
| Bourne (2018)^6^ | Y | N | Y | N | Y | N | N | P | N | N | n/a | n/a | N | Y | n/a | Y | 42.3 |
| Bundock (2017)^7^ | N | P | N | Y | N | N | N | Y | Y | N | n/a | n/a | Y | Y | n/a | N | 42.3 |
| Coren (2011)^9-11^ | Y | P | Y | P | Y | Y | Y | Y | Y | N | n/a | n/a | Y | Y | n/a | Y | 84.6 |
| Crowe (2010)^65^ | N | N | N | P | N | N | N | N | N | N | n/a | n/a | N | N | n/a | Y | 11.5 |
| Exell (2020)^12^ | N | P | Y | P | N | N | N | N | Y | N | n/a | n/a | Y | N | n/a | Y | 38.5 |
| Feng (2020)^66^ | Y | Y | Y | Y | Y | Y | N | P | Y | N | Y | Y | Y | Y | Y | Y | 84.4 |
| Fernandez-Sotos (2020)^13^ | N | N | N | P | Y | N | N | N | N | N | n/a | n/a | N | N | n/a | Y | 19.2 |
| Finkel (2020)^14^ | Y | N | Y | Y | Y | N | N | P | N | N | n/a | n/a | N | N | n/a | Y | 42.3 |
| Firth (2015)^15^ | N | N | N | P | Y | N | N | Y | Y | N | Y | N | N | Y | N | Y | 40.6 |
| Firth (2016)^16^ | Y | N | Y | P | Y | N | N | N | N | N | Y | N | N | N | N | Y | 34.4 |
| Geretsegger (2017)^20^ | Y | Y | Y | Y | Y | Y | Y | Y | Y | N | Y | Y | Y | Y | Y | Y | 93.8 |
| Gonzalvez (2018)^21^ | Y | N | Y | P | N | N | N | Y | N | N | Y | N | N | Y | Y | Y | 46.9 |
| Grant (2017)^22^ | Y | N | N | P | Y | N | N | Y | N | N | n/a | N | Y | Y | n/a | Y | 46.4 |
| Hallett (2019)^23^ | N | N | N | N | N | N | N | N | N | N | n/a | n/a | P | N | n/a | Y | 11.5 |
| Howarth (2016)^24^ | N | N | Y | Y | Y | N | N | N | Y | N | n/a | n/a | Y | N | n/a | N | 38.5 |
| Hutchinson (2015)^25^ | N | N | N | P | N | N | N | P | Y | N | n/a | n/a | Y | N | n/a | N | 23.1 |
| Hutzler (2010)^26^ | N | N | N | P | N | N | N | N | Y | N | n/a | n/a | N | N | n/a | N | 11.5 |
| Kaltenthaler (2014)^27^ | Y | Y | Y | Y | N | N | Y | Y | Y | Y | n/a | n/a | Y | N | n/a | Y | 76.9 |
| Kamioka (2014)^28^ | Y | P | Y | Y | Y | Y | Y | N | Y | Y | n/a | n/a | N | N | n/a | Y | 73.1 |
| Lindsay (2019)^29^ | N | N | N | P | N | N | N | N | N | N | n/a | n/a | N | N | n/a | Y | 11.5 |
| Lorenc (2018)^30,31^ | Y | P | N | Y | Y | N | N | Y | Y | N | n/a | n/a | N | N | n/a | N | 42.3 |
| Louw (2019)^32^ | N | N | N | P | N | Y | N | N | Y | N | n/a | n/a | N | N | n/a | N | 19.2 |
| Ma (2020)^33^ | Y | N | N | P | Y | N | N | Y | Y | N | n/a | n/a | Y | N | n/a | Y | 50.0 |
| Masi (2011)^34^ | Y | N | Y | P | N | N | N | N | N | N | Y | N | N | Y | N | Y | 34.4 |
| Maujean (2015)^35^ | N | N | Y | P | Y | N | N | N | N | N | n/a | n/a | N | N | n/a | N | 19.2 |
| McCann (2019)^36^ | Y | N | N | P | Y | Y | N | P | Y | N | n/a | n/a | N | Y | n/a | N | 46.2 |
| Morin (2017)^37^ | N | N | Y | P | N | N | N | N | N | N | n/a | n/a | N | Y | n/a | N | 19.2 |
| Naslund (2015)^38^ | Y | N | Y | P | N | N | N | Y | N | N | n/a | n/a | Y | N | n/a | Y | 42.3 |
| Nguyen (2020)^39^ | N | N | N | P | Y | Y | N | N | Y | N | n/a | n/a | N | Y | n/a | Y | 42.3 |
| Palmen (2012)^40^ | N | N | Y | P | N | Y | N | N | N | N | n/a | n/a | Y | N | n/a | N | 26.9 |
| Pallathra (2019)^41^ | N | N | N | P | N | N | N | N | N | N | n/a | n/a | N | N | n/a | Y | 11.5 |
| Pescheny (2020)^42^ | N | N | N | P | N | N | N | N | N | N | n/a | n/a | Y | Y | n/a | Y | 26.9 |
| Petroutsou (2018)^43^ | Y | N | N | P | N | N | N | Y | Y | N | n/a | n/a | Y | n/a | n/a | Y | 45.8 |
| Puolakka (2019)^45^ | Y | N | N | P | Y | Y | N | Y | Y | N | n/a | n/a | N | N | n/a | N | 42.3 |
| Quirk (2020)^46^ | Y | Y | Y | P | N | N | N | Y | Y | N | Y | Y | Y | Y | N | Y | 65.6 |
| Ratti (2016)^47^ | N | N | N | P | N | N | N | N | P | N | n/a | n/a | N | N | n/a | Y | 15.4 |
| Roche (2019)^48^ | Y | N | Y | P | N | N | N | N | N | N | n/a | n/a | N | N | n/a | N | 19.2 |
| Sala (2019)^49^ | N | N | N | P | N | N | N | P | Y | N | n/a | n/a | Y | N | n/a | Y | 30.8 |
| Schepens (2019)^50^ | N | N | N | P | N | N | N | N | P | N | n/a | n/a | N | N | n/a | Y | 15.4 |
| Seewooruttun (2014)^67^ | N | N | N | P | N | N | N | P | N | N | n/a | n/a | N | Y | n/a | N | 15.4 |
| Soundy (2014)^52^ | Y | N | Y | P | N | N | N | Y | Y | N | Y | n/a | N | N | n/a | Y | 46.4 |
| Takahashi (2019)^53^ | N | N | N | P | N | N | N | N | P | N | n/a | n/a | y | Y | n/a | N | 23.1 |
| Tint (2017)^54^ | Y | N | N | P | Y | N | N | Y | N | N | n/a | n/a | Y | Y | n/a | N | 42.3 |
| Tobin (2014)^55^ | N | N | Y | P | Y | N | N | P | N | N | n/a | n/a | Y | Y | n/a | N | 38.5 |
| Walker (2013)^56^ | N | N | Y | N | N | N | N | N | Y | N | n/a | n/a | N | N | n/a | N | 15.4 |
| Webber (2017)^57^ | Y | N | N | N | N | N | N | N | N | N | n/a | n/a | N | N | n/a | Y | 15.4 |
| Wilson (2014)^60^ | N | N | N | P | N | N | N | N | N | N | n/a | n/a | N | N | n/a | N | 3.9 |

*AMSTAR quality domains:

1. Did the research questions and inclusion criteria for the review include the components of PICO?
2. Did the report of the review contain an explicit statement that the review methods were established prior to the conduct of the review and did the report justify any significant deviations from the protocol?
3. Did the review authors explain their selection of the study designs for inclusion in the review?
4. Did the review authors use a comprehensive literature search strategy?
5. Did the review authors perform study selection in duplicate?
6. Did the review authors perform data extraction in duplicate?
7. Did the review authors provide a list of excluded studies and justify the exclusions?
8. Did the review authors describe the included studies in adequate detail?
9. Did the review authors use a satisfactory technique for assessing the risk of bias (RoB) in individual studies that were included in the review?
10. Did the review authors report on the sources of funding for the studies included in the review?
11. If meta-analysis was performed did the review authors use appropriate methods for statistical combination of results?
12. If meta-analysis was performed, did the review authors assess the potential impact of RoB in individual studies on the results of the meta-analysis or other evidence synthesis?
13. Did the review authors account for RoB in individual studies when interpreting/ discussing the results of the review?
14. Did the review authors provide a satisfactory explanation for, and discussion of, any heterogeneity observed in the results of the review?
15. If they performed quantitative synthesis did the review authors carry out an adequate investigation of publication bias (small study bias) and discuss its likely impact on the results of the review?
16. Did the review authors report any potential sources of conflict of interest, including any funding they received for conducting the review?

## **Supplementary Table 3**. Intervention or program characteristics and effects

|  | **Intervention type and description** (including first author & year of primary studies)   - Setting - SR first author (publication year; AMSTAR proportion); Number of studies for intervention type | **Disability** (diagnosis)   - Pooled N* - Demographics - Countries | **Duration of intervention, number and frequency of sessions** | **Study designs (Control Group type)** | | **Outcome type and effects** | | **Caveats or limitations of interventions** | **Overall effects and quality of studies** |
| --- | --- | --- | --- | --- | --- | --- | --- | --- | --- |
| *1* | *Intensive tailored support* for people with ID and CB (Lowe, et al. ^68^, ?Q), and interventions centred on *positive behaviour support* using strategies such as behavioural and residential assessment for people with ASD and PSDs (Magito-McLaughlin, et al. ^69^, ?Q), rapport building, functional communication training, tolerance of delay, choice and embedding (Carr, et al. ^70^, ?Q), planning meetings, respite care, crisis response, and environmental modifications (McClean, et al. ^71^, ?Q).   - Integrated and segregated - Bigby**^3^** (2012, A*: 0.27); 4 studies | **ID** with CB X3 and **ASD/PSD**^69^   - N=39 - 14-39 years (nr X3 studies) - Sex nr for N=30 - Countries nr | Support provided and assessed continuously over ~9-13 months,^68^ 2 years,^71^ and 5-years^70^++  A series of PCP meetings^69^++ | - Descriptive case study ^69,71^ - Multi-methods^70^ - NRCT-No CG^68^ | | **Social functioning**   - No ∆ overall Index of Community Involvement scores, although services receiving individual intensive support had ↑ participation in community life and ↑ contact with staff.^68^ - ↑ use of community settings, work, social relationships and choice,^70^ ↑participation in community based activities for 3/5 cases,^71^ and ↑ time spent in community settings by m=20 minutes per month.^69^   **Social capacity**   - +/o effect on challenging behaviours: No ∆ in the largest study (n=30)^68^, but ↓ behaviours in small studies of n=3-5.^70,71^   **Other**   - Appeared to help people participate in employment or training.^69,71^ | | - Existing staffing and policies can affect implementation of intensive support.^68^ | Social functioning: Overall positive effects, although probably for limited domains  Quality nr |
| *2* | *Person Centred Planning* interventions. Most studies were not described in detail (Heller, et al. ^72^, HQ; Magito-McLaughlin, et al. ^73^, MQ; Malette ^74^, MQ; Robertson, et al. ^75^, MQ; Hagner, et al. ^76^, MQ).  Parley (2001^77^, LQ) observed service delivery and gathered nurse views of PCP.  PCP interventions sought to support lifestyle plans, health, and participation led by staff (agent nr for 5/10 studies) in residential or day centre settings (setting nr for 2 studies) (Espiner and Hartnett ^78^, MQ; Smith and Carey ^79^, LQ; Jensen, et al. ^80^, ?Q; Mansell, et al. ^81^, ?Q).   - Integrated and segregated - Bigby**^3^** (2012, A*: 0.27), Howarth**^24^** (2016, A*: 0.38), Ratti**^47^** (2016, A*: 0.15), Schepens**^50^** (2018, A*: 0.15); 10 studies | **ID** (mild-moderate-profound, some with PSD)   - N=195 (169 IGs, 26 CGs) - M=35-57, 13-68 years - Sex nr for N=173 - Canada, Ireland, New Zealand, UK X2, USA X3 (nr X2) | Hagner, et al. ^76^assessed a single PCP meeting,  Robertson, et al. ^75^ measured PCP for 96 people over a period of 2 years, Jensen^80^ included “Several PCP meetings” and 4hrs daily community outings. ++  Espiner^78^ evaluated the PCP planning meeting with adults with ID after staff attended a 2-day training programme. ++  Smith^79^ described a single PCP meeting and action plan with a multidisciplinary team that was then coordinated by a nurse.++  nr^72-74,77,81^ | - Descriptive case study^79,80^ - Qualitative ^74,76-78^ - NRCT-No CG^75,81^ - RCT – “traditional model” CG^73^ or CG not described^72^ | | **Social functioning**   - ↑ social inclusion,^79^ ↑ community participation/involvement^74^ and ↑ acquaintances in community settings (e.g., restaurants, museums)^80^ - ↑ contact with friends and social network size (52% increase in network size but this did not extend to include people other than close family or staff),^75^ and sense of social connection although most individuals continued to have very few friendships with peers.^76^ Magito-McLaughlin, et al. ^73^ found no ∆ in social networks between IG and CG - ↑ number and variety of meaningful^81^ or community activities,^75^ ↑ variety in going to community locations (m=22 per person in IG vs m=5 per person in CG) and ↑ time in inclusive experiences (86% inclusive for IG vs 32% inclusive for CG)^73^ and ↑ access to community settings (m=20 per month)^80^ - ↑ hrs of activities scheduled per week,^75^ ↑ number of activities (m=30 for IG vs m=20 for CG) with people in IG spending more time in active recreation, personal management and community errands vs CG who spend more time in group trips and passive leisure activities.^73^ - The study by Hagner, et al. ^76^ found that at 6-months only a few outcomes had been achieved and “not much had happened”   **Other**   - ↑ self-determination^78^ and ↑ choice,^80^ including 2.8-fold ↑ participating in choice-making vs baseline,^75^ and ↑ choice for specific activities of “how to decorate your room” and jobs in the workplace.^72^ While Parley ^77^ noted an increase in choice with PCP there was no ∆ in PWD planning their own care or making major life decisions for themselves - ↓ challenging behaviours^73^ | | - Few family members and community members that could enable community connections attended PCP meetings, therefore reducing opportunities for further interactions.^78^ - PCP may be more effective for short-term goals (e.g., choice-making and participation in activities) than longer-term changes.^75^ - A plan was not developed for 30% of people within the timeframe of the study, suggesting PCP may face even greater challenges in the absence of project-specific resources.^75^ - Staff emotional and instrumental support were reported to be the single most important facilitator of goal attainment ^72^ - Planning meetings are often overpowered by staff or family members and the contributions of people with ID are often ignored or reinterpreted.^76^ | Social functioning: Overall positive effects, but limited in some settings (not clear why from available data)  Quality: 2 low, 5 mod, 1 high (2 Xnr) |
| 2 | Other *Person Centred Planning* interventions focused on future planning for parents of people with ID (Bigby ^82^, LQ); a multidimensional residential and participation planning/support (Lowe and De Paiva ^83^, MQ), provision of individual and group meetings and individual network for intervention mapping to enhance participation (Broer, et al. ^84^, MQ), or an investigation into changes in social and community participation in different residential settings (Di Terlizzi ^85^, ?Q).   - Integrated and segregated - Bigby**^3^** (2012, A*: 0.27), Howarth**^24^** (2016, A*: 0.38), Schepens**^50^** (2018, A*: 0.15); 4 studies | **ID** living in aged care,^82^ severe ID^85^ or ID and PSD^84^ (nr X1)^83^   - N=445 (420 IGs, 25 CG) - R: 40-85 years - Sex nr consistently - Australia, Wales, Netherlands (nr X1) | Staff attended workshops at national conferences and provided support to clients over time (outcomes assessed 3m and 12m)^84^++  intervention duration n/a ^82,83,85^++ | - Descriptive case study^85^ - Mixed methods^84^ - NRCT-No CG^82^ - NRCT - CG was an area outside of the catchment area^83^ | | **Social functioning**   - ↑ interpersonal relations^82^ - +/o effects on use of community facilities: small proportion in contact with friends, with little ∆ during study, People who moved out of hospital setting ↑ use of community facilities.^83^ - No ∆ social networks, but ↓ loneliness and seeing friends more often^84^ - ↑es in social interaction and engagement associated with lower levels of challenging behaviour^85^   **Other**   - ↑ Self-determination and ↑ emotional wellbeing^82^ - Withdrawal of social participation has ++ impact on QoL^85^ | | Nr | Social functioning: Overall positive effects, although limited/mixed effects on social network  Quality: 1 low, 2 mod (nr X1) |
|  | *Social skills/communication training* |  |  |  | |  | |  |  |
| *3* | *Social skill training* (SST) that did not explicitly define “cognitive” elements were evaluated in 7 meta-analyses (Aleman, et al. ^86,^Bellack ^87,^Elis, et al. ^88,^Kurtz and Mueser ^89,^Mueser and Penn ^90,^Pfammatter, et al. ^91,^Pilling, et al. ^92^; all ?Q) and in studies with varying components of interaction and theory of mind training (Kayser, et al. ^93^, LQ). Social skills training generally targeted interpersonal skills and social problem solving (Hayes, et al. ^94^, MQ), social perception, social information processing, responding and sending skills, affiliative skills, interactional skills, social norms, and applications to everyday situations (Rus-Calafell, et al. ^95^, ?Q; Rus-Calafell, et al. ^96^, MQ; Rus-Calafell, et al. ^97^, ?Q), roleplaying (Marzillier, et al. ^98^, LQ).  SST could also include virtual reality “workouts” and scenarios to practice social interaction skills in simulated everyday contexts (Adery, et al. ^99^, Park, et al. ^100^, both ?Q), case management (Björkman, et al. ^101^, LQ), illness management (Hasson-Ohayon, et al. ^102^, LQ), and family-oriented psychoeducation (Aberg-Wistedt, et al. ^103^, LQ). One study provided in-clinic SST and support to generalise to the community (Glynn, et al. ^104^, ?Q). The design of SST was not described for 2 studies (Bustillo, et al. ^105^, Turner, et al. ^106^, both ?Q).   - Segregated - Fernandez-Sotos**^13^** (2020, A*: 0.19), Grant**^22^** (2017, A*: 0.46), Ma**^33^** (2020, A*: 0.50), Morin**^37^** (2017, A*: 0.19), Puolakka**^45^** (2019, A*: 0.42), Webber**^57^** (2017, A*: 0.15); 7 SR, 14 studies | **PSD** (Schizophrenia, schizoaffective disorders, SMI, psychotic disorders)   - N=549^+^ - M=32-36, R: 17-51 years - 75.8% male - Australia, Israel, UK, Spain, Sweden X2 reported for 7/13 studies   ^+^ N nr for SRs & 3 studies | 2 X 1hr sessions^93^  15 X 45 mins/wk for 3m^98^  1 hr/wk for 8m^102^  1.75hr/wk for 18m^101^  2 1-1.25hr sessions/wk X 18 weeks ^95-97^ plus 9 boosters^94^  10 sessions,^100^ 2X wk^99^  Nr^86-89,91,92,103,105^ | - Meta-analysis^86-92^ & SRs ^105,106^ - NRCT- No CG^95,97,99^ - RCT - TAU/ waitlist CG^93,96,98,101-103^ - RCT - TAU & discussion group CGs^94^ - RCT - active CG: clinic training, but no community support ^100,104^ | | **Social functioning**   - ↑ social role functioning ^87^, moderate ↑ social functioning (effect=0.52)^89^, including role functioning, social relations and overall social adjustment^104^ - No ∆ perceived social support at treatment end and 9m^102^ - Social activities and contacts: ↑ for SST or Systematic Desensitisation vs waitlist group,^98^ and ↑ for SST with family psychotherapy that did not differ from CG^103^ - Social functioning was measured by Rus-Calafell, et al. ^95,97^ and Björkman, et al. ^101^, but nr in the systematic reviews**^13,33^**   **Social skills**   - “little evidence of benefits” (but did not specify the outcome)^92^ - ↑ behavioural skills^87^ - ↑ social skills, including conversation, problem-solving strategies,^91,100^ usually maintained at 2 yrs^90,105^ - ↑ theory of mind^93^ - Minimal ∆ in non-verbal skills^100^   **Other**   - No ∆ relapse,^87,91^ but ↓ emergency visits^103^ - +/o symptoms: ↓ overall symptoms,^99^ ↓ negative symptoms ^86,88,95,97,106^ or no ∆ overall symptoms^87^ - ↑ personal recovery and progress towards goals^102^ - No ∆ quality of life ^94,101^ or physical health ^96^, but improved mental health (post-treatment, Hedges g=2.21; 6-month follow-up, hedges g= 1.23; both p<0.05)^96^ | | - It is important for SST programs to help people to apply the skills to everyday life.**^37^** - In 2017 SST was not recommended by NICE for schizophrenia due to limited robust RCT evidence.**^57^** | Social functioning/ social contacts: Positive effects  Social support: No effect  Social skills: Mostly positive effects  Recovery/ symptoms: Mostly positive effects  Quality: 5 low, 2 mod (nr X 14) |
| *4* | *Social Cognitive Training* that emphasised reframing perception of loneliness and self-control, learning coping strategies, increasing sense of belonging and stress management (Conoley and Garber ^107^, ?Q; Williams, et al. ^108^, ?Q; Horan, et al. ^109^, MQ)   - Clinical/Segregated - Grant**^22^** (2017, A*: 0.46), Masi**^34^** (2011, A*: 0.34); 3 studies | **PSD** (depression, schizophrenia)   - N=269 (nr IG/CG Ns) - M=20-50 years - 64% male (100% female)^107^ - USA X2 (nr X1) | **Length**: 2wks, 9wks, 12wks  **Frequency**: weekly  **Time**: 30mins, 45mins, 1hr | - RCT – “no intervention” CG & “instructed to try harder” CG^107^ - RCT – active CGs: weekly meetings on topics other than social skills^108^ or illness management skills training^109^ | | **Social functioning:**   - +/o for loneliness: No ∆ for low intensity 2w intervention (Effect=-0.32, 95%CI: -0.96, 0.32)^107^ but ↓ after 6w intervention (Effect=–0.36, 95%CI: –0.64, –0.08)^108^   **Capacity**   - No ∆ in theory of mind or attribution style^109^ - ↑ affect recognition^109^ | | Nr | Loneliness: positive effects for longer-term intervention  Moderate quality (nr X2) |
| *5* | *Social Cognition and Interaction Training* (SCIT) targets impairments in social cognition, and typically includes exercises, games, discussions and interactive social stimuli to improve specific areas of social cognitive dysfunction (Roberts, et al. ^110^, MQ; Wang, et al. ^111^, MQ; Combs, et al. ^112^, MQ; Taylor, et al. ^113^, MQ; Hasson-Ohayon, et al. ^114^, MQ; Gohar, et al. ^115^, MQ; Horan, et al. ^116^, MQ; Gil Sanz, et al. ^117^, LQ, Gil-Sanz, et al. ^118^, MQ, Bechi, et al. ^119^, MQ), and could include video-based augmented reality simulation (Bechi, et al. ^120^, MQ), cognitive remediation (Stravynski, et al. ^121^, LQ) and family members (F-SCIT; Tas, et al. ^122^, MQ). SCIT was provided in a group^110,111,114^ or individual program ^121^; however, the format was not described for all other studies.   - Segregated - Anderson**^1^** (2015, A*: 0.58), Grant**^22^** (2017, A*: 0.46), Ma**^33^** (2020, A*: 0.50), Webber**^57^** (2017, A*: 0.15); 13 studies | PSD (schizophrenia, social phobia, avoidant personality disorder)   - N=719 (396 IGs, 291 CGs, nr X1) - M=33-51 years - 68.1% male - Country not consistently reported | Most had 1-2 X 1-1.5hr sessions/wk for 16-24 weeks | - RCTs - TAU or “no intervention” CGs^110,111,113,116,120^ - RCTs - active CG: cognitive training,^118^ Coping group,^112^ emotion training,^117^ social stimulation,^122^ mentoring only;^114^ active CG not defined,^119^ “skills training”,^115^ or SST without cognitive remediation^121^ | | **Social functioning**   - ↑ social performance/functioning scales^110-112,122^ - ↑ social engagement (Effect=0.35) but no ∆ interpersonal communication (Effect=0.01) vs CG^114^ - ↓ social isolation for all groups with no difference between groups^121^   **Social skills**   - Global social cognition/perception/ knowledge: ↑^122^ or no ∆^110,112^ - +/o theory of mind: ↑^111-114,116,118-120,122^ or no ∆ ^110,116^ - Affect recognition: ↑^110-112,115,116,118,122^ or no ∆^114,120^ - Attribution style: ↑^112,113^ or no ∆^110,111,114,122^ or +/o^116^   Other   - ↑ personal care and daily activities ^117^ | | nr | Social functioning: positive effects  Affect recognition: mostly positive effects  Social skills/ Theory of mind: mixed effects  Attribution style: mostly no effect  Quality: 3 low, 10 mod |
| 6a | A *TEACCH-based social skills/ communication training* program targeting negative problem behaviour (Galli Carminati, et al. ^123^, ?Q), a social skill based group program that aims to raise social awareness and enhance interpersonal communications and listening in relationships with a CBT-framework (McGaw, et al. ^124^, MQ). The *Australian Supported Learning Program* (ASLP; McConnell, et al. ^125^, MQ), which supports social skill and goal development of mothers with ID. A social skills and rights program focused on developing social and civic competencies (Iconaru and Ciucurel ^126^, HQ)   - Segregated: Residential or not defined - Bundock^7^ (2017, A*: 0.42), Howarth**^24^** (2016, A*: 0.38), Louw**^32^** (2019, A*: 0.19), Wilson**^60^** (2014, A*: 0.04); 4 studies | **ID** (mild/borderline ID, PDD and “learning disability”)   - N=71 - M=29-39 years, R: 17-48 years - 37.7% male (nr X1) - Australia, Romania, England and Switzerland | **Length**: 7d, 12wks, 14wks  **Time**: 2 hrs/wk  (nr 1 study) | - NRCT-No CG^123,125,126^ - RCT – CG not described^124^ | | **Social functioning**   - ↓social withdrawal^123^ - 54% of IG had ↑ relationships with partners and new friendships, with no ∆ in CG^124^ - Parents valued the support and continued with informal support group after the study.^124^ - ↑ confidence and knowledge about getting about the community, resources for participation, and joining groups or enrolling in courses^125^   **Other**   - ↑ social contact associated with ↓ aberrant behaviours^123^ - ↑ self-concept in IG^124^ - Most popular goals were enjoyment of life and learning about own strengths, and 90-100% at least partially achieved those goals ^125^ - ↑er QoL is associated with participation as a citizen, understanding civil rights and civic engagement ^126^ | | - ↑ participant engagement may have led to ↑ positive and social behaviour initiation, regardless of intervention.^123^ - digital literacy skills negatively impact on connecting with others.^126^ - Single parents with ID are more vulnerable and need additional or tailored support.^124^ | Social functioning: positive effects  Quality: 2 mod, 1 high (nr X1) |
| *6b* | *Group social skills training* programs, including *Social Cognition and Interaction Training (SCIT‐A)* using video cues, and focused on 3 aspects of social cognition (emotion recognition, directing attention in social interactions, perspective taking; Turner-Brown, et al. ^127^, LQ). The *Putting feet on my dreams* included instruction, discussion and video feedback role-play focused on social skills (Fullerton and Coyne ^128^, LQ), *Problem Solving Skills 101* (Pugliese and White ^129^, LQ). The other *social skills training, groups or programmes* focused on similar social and conversational skills and used similar methods (e.g., role-play, discussion group, multimedia) to SCIT-A (Lovett and Rehfeldt ^130^, LQ; Minihan ^131^, ?Q; Howlin and Yates ^132^, ?Q; Ashman, et al. ^133^, LQ)   - Segregated - Atkinson-Jones**^2^** (2019, A*: 0.15), Lorenc**^30,31^** (2018, A*: 0.42), Pallathra**^41^** (2019, A*: 0.12), Palmen**^40^** (2012, A*: 0.27), Tobin**^55^** (2014, A*: 0.38); 7 studies | **ASD** (severity nr)   - N=78 - M=21-36, R: 16-55 years - 85.3% male - Ireland, UK X2, USA X2 (nr X2) | **Length**: 4-6wks, 9wks, 16wks, 18wks, 12m, 10 sessions  **Time**: 30-60 mins/ session,^127,130,133^ 2.5hrs/month,^132^ 3hrs/wk^128^  Nr ^129^ | - NRCT-No CG^128-130,132^ - NRCT – CG not described^131^ - RCT – active CG: social interactions without training^127^ - RCT – CG not described^133^ | | **Social functioning**   - ↑ social functioning and theory of mind skills, but did not differ from CG, which also included social interactions^133^   **Social skills**   - ↑ dyadic conversation skills^128^ and social problem solving skills^129^ - No ∆ in social communication skills (g=0.30, 95%CI: -0.90, 1.50), or social skills performance (g=-0.02, 95%CI: -1.20, 1.17)^127^ - ↑ face emotion identification and theory of mind^127^ - Social skill generalisation to natural social interactions^130^ - ↑ attention (g=0.95, 95%CI: -0.10, 1.99) and feedback questions (g=1.85 (0.69, 3.02), but no ∆ in other skills (eye contact, gestures; conversation initiation, maintenance and handover; perseveration, no interaction, inappropriate statements^131^ - ↑ conversations maintenance/initiation, appropriate responses and ↓ inappropriate utterances/repetitions, but no ∆ in total utterances, appropriate/inappropriate responses^132^   **Acceptability**   - Moderate (70%) to high attendance (92%)^127^   **Cost**   - Social skills group was not expensive or time-consuming^132^ | | - Social opportunities alone may be sufficient to improve social skills and function.^133^ - ‘‘unless great care is taken, problems may arise from providing individuals with training in certain areas of social interaction whilst failing to address more fundamental issues’’ ^132^(p. 305). - Important to match participant expectations with intervention design,^132^ and for participants to establish their own goals.^133^ | Social functioning and skills: Positive effect no greater than social interaction group  Conversation/ social skills: Mixed effects  5 low quality (nr X2) |
| *7* | *Program for the Education and Enrichment of Relational Skills for Young Adults* (PEERS‐YA) is a caregiver-assisted social skills program for adolescents that has been adapted for young adults with ASD (Gantman, et al. ^134^, Laugeson, et al. ^135^, White, et al. ^136^, McVey, et al. ^137^; all LQ). The PEERS-YA program provides instruction and rehearsal of social skills specifically related to building and maintaining peer relationships in separate group sessions for participants and caregivers. Topics include developing friendship networks handling peer pressure, conversation skills, and dating etiquette.   - Segregated - Atkinson-Jones**^2^** (2019, A*: 0.15), Lorenc**^30,31^** (2018, A*: 0.42), Pallathra**^41^** (2019, A*: 0.12), Tobin**^55^** (2014, A*: 0.38); 4 studies | **ASD** without ID   - N=97 (55 IG, 42 CG) - M=20-24 years - 80.4% male - USA X4 | **Length/ frequency**: Weekly for 14-16 wks  **Time**: 1.5 hr/session | - NRCT-No CG^136^ - RCT – CG “no intervention” ^134,135,137^ | | **Social functioning**   - +/o effects on Social Responsiveness Scale: ↑ function (g=1.26, 95%CI: 0.19, 2.32;^135^ g=1.05, 95%CI: 0.02, 2.08)^134^ or no ∆ (g=0.41, 95%CI: -0.17, 0.99)^137^ - +/o effects on quality of socialisation Scale: ↑ socialisation quality (g=1.16, 95%CI 0.11, 2.21, self-rated; g=2.58, 95%CI 1.21, 3.96, carer-rated)^135^ or no ∆ (g=0.38, 95%CI -0.19, 0.96)^137^ - ↑ invited to get-togethers domain of quality of socialisation (g=1.20, 95%CI 0.14, 2.26)^134^ but no change in hosting get-togethers (g=0.83, 95%CI -0.18, 1.83)^134^ - No ∆ loneliness (g=1.00, 95%CI: -0.03, 2.03)^134^ including romantic loneliness (g=0.13, 95%CI: -0.44, 0.71), family loneliness (g=-0.06, 95%CI: -0.63, 0.52) and social loneliness (g=-0.20, 95%CI: -0.77, 0.38)^137^   **Social skills**   - Improved asking questions, topic changes, social involvement, rapport and social anxiety^136^ - +/o effects on social skills rating/improvement system: ↑ social skills (g=1.48, 95%CI: 0.37, 2.59)^134^ but no ∆ (g=0.10, 95%CI: -0.85, 1.05;^135^ g=0.21, 95%CI: -0.37, 0.78)^137^ and no ∆ social behaviour (g=0.57, 95%CI: -0.01, 1.15)^137^ - No ∆ empathy (g=1.02, 95%CI: -0.01, 2.05;^134^ g=1.02, 95%CI: -0.01, 2.05;^135^ g=0.42, 95%CI: -0.16, 1.00)^137^ | | nr | Social skills and function: Mixed effects, but mostly positive  Loneliness, empathy and social skills: No/limited effects  3 low quality (nr X1) |
| *8* | *Individual social skills training* programs (Tiger, et al. ^138^, HQ; Perdue ^139^, ?Q; ^140^, ?Q; Gaylord-Ross, et al. ^141^, ?Q). Strategies included coaching (including virtual reality training; Kandalaft, et al. ^142^, LQ), reinforcement, and the use of concrete organizational skills (e.g., using a planner, inviting peers to activities; Koegel, et al. ^143^, LQ) or psychoeducation and social skills coaching to help with social activity scheduling (White, et al. ^144^, ?Q).   - Segregated - Bundock^7^ (2017, A*: 0.42), Lorenc**^30,31^** (2018, A*: 0.42), Pallathra**^41^** (2019, A*: 0.12), Palmen**^40^** (2012, A*: 0.27); 7 studies | **ASD** (all without ID except Gaylord-Ross, et al. ^141^)   - N=31 - M=20-23, R: 17-20 years - 77.3% male - USA X5 (nr X2) | 1-2 X 5min sessions/ day,^141^ 2 X 1hr sessions/wk,^142^ 1 hr/wk for 33 wks,^143^ 40 mins/wk for a total of 10-14 sessions^144^  Nr ^138-140^ | - Multiple baseline case design^138-141^ - NRCT-No CG^142,143^ - RCT with active CG: VR social skills training using brain computer biofeedback^144^ | | **Social functioning**   - ↑ social event attendance^143^ - ↑ satisfaction with college,^143^ but no ∆ adaptation to college (g=0.05, 95%CI: -1.34, 1.44)^144^ - ↑ satisfaction with peer interactions^143^ - Social skills/behaviour - ↑ initiation and sustained interactions with peers^141^ - ↑ (ns) appropriate verbal/non-verbal engagement or food etiquette^139^ - ↑ (ns) reciprocal social initiation, non-verbal interactions, perceived effectiveness^140^ - ↑ social skill performance^142^ - ↓ latency to question responding^138^   **Other**   - ↑QoL, grade point average, employment,^143^ but no ∆ executive functioning (g=0.18, 95%CI: -1.21, 1.57)^144^ | | nr | Social functioning: Positive effect  Social skills: Mixed effects (ns, but mostly multiple baseline design)  Quality: low X2, high X1 (nr X4) |
| *9a* | *Intensive interaction* support to enhance communication and interaction behaviours that are generally tailored to the PWD impairments, such as hand contact and vocalisations (Elgie and Maguire ^145^, LQ), smiling, eye contact and joint attention (Leaning and Watson ^146^, MQ; ^147^, MQ), initiating social contact (Nind ^148^, MQ) or other social/communicative behaviours not defined (Nind ^149^, HQ; Samuel, et al. ^150^, HQ; Zeedyk, et al. ^151,152^, MQ; Forster and Taylor ^153^, LQ). Most interventions were provided by psychologists or after training residential care staff.   - Segregated – mostly in residential care setting - Bundock^7^ (2017, A*: 0.42), Hutchinson**^25^** (2015, A*: 0.23); 8 studies | **ID** (profound & multiple ID [PMID] X6, severity nr X1) & **ASD** (severity nr)   - N=27 - 28-53 years - 59% male - UK X7 & Australia | Varied duration from 15 mins-2hrs per session for:  17 X 5min sessions over 3 days^147^  Daily for 12-18m^148^  5 X wk for 12m^150^  50m per/wk X 8 wks^146^  3 X wk for 16 wks^145^ | - Descriptive case study^151,152^ - Multiple baseline case design^145-150^ - Qualitative^153^ | | **Social behaviour/participation**   - ↑ sociability,^148^ social contact and new interactions^149^ and ↑ social behaviours^146^ - Targeted social behaviour: ↑ social behaviours,^146^ ↑ hand contact,^145^ ↑ reciprocal “warm” contact,^149^ smiling/laughing ↑^146^ or no ∆,^147^ ↑ looking at face and/or initiating social/ communicative contact^150-153^ - Challenging behaviour: ↓ self-stimulation,^146^ ↓ covering face,^147^ no ∆ to vocalisations^147^ or self-injurious behaviour^145^ | | - Ethical concerns about using multiple baseline approach – providing and withdrawing support for PMID) - Difficult to maintain intervention. - Difficult to ∆ severe and chronic self-injurious behaviour. | Social/ communication behaviour: Positive effects but not consistently maintained post-intervention  Quality: 2 Low, 4 mod, 2 high |
| 9b | Training in *specific conversation or communication skills* with video feedback (Koegel, et al. ^154^, ?Q), including reciprocal conversations (Mason, et al. ^155^, LQ; Trepagnier, et al. ^156^, LQ), reducing negative statements with video feedback (Koegel, et al. ^157^, LQ), asking questions in class (Palmen, et al. ^158^, LQ), or answering social questions (Sperry and Mesibov ^159^, HQ). Using Social Stories to target specific social behaviours (Samuels and Stansfield ^160^, ?Q).   - Segregated - Bundock^7^ (2017, A*: 0.42), Lorenc**^30,31^** (2018, A*: 0.42), Pallathra**^41^** (2019, A*: 0.12), Palmen**^40^** (2012, A*: 0.27), Tobin**^55^** (2014, A*: 0.38); 7 studies | **ASD** (one study included people with ASD & ID)   - N=57 - M=21-34, most samples 17-32 years, - Sex not consistently reported - USA X3 (nr X4) | Usually 1X week for 2wks,^156,160^ 4-8w,^157^ 5w,^155^ 6w,^158^ 5-9w^154^  nr ^159^  sessions were 10-50min per/wk | - Multiple baseline case design^155,157,158,160^ - Qualitative focus groups^159^ - NRCT-No CG^154,156^ | | **Social functioning**   - ↑ socialisation satisfaction^154^ - Participants reported improved personal relationships at work and in the community^159^   **Social skills**   - ↑ targeted social skills, including asking correct questions^158^ and empathic listening, questions and statements^154^ - ↓ problematic behaviours (e.g., bossy talking, inappropriate language, trying to video people), but returned to baseline levels for most behaviours post-intervention^160^   Note: results nr for three studies^155-157^ | | nr | Social/ conversational skills: Positive effects but limited maintenance  Social functioning: Positive effects  Quality: 4 low, 1 high (nr X2) |
| 10a | *Theory of mind training* through discussion, modelling and homework (Newey, et al. ^161^, ?Q); computerised *social cognition and emotion recognition training* (Bölte, et al. ^162^, LQ; Golan and Baron-Cohen ^163^, LQ), training to enhance *comprehension of irony* using video clips, stories and comics (Saban-Bezalel and Mashal ^164^, LQ) and face recognition training with the *Studies To Advance Autism Research & Treatment (STAART)* program (Faja, et al. ^165,166^, LQ) training. The STAART program focused on memory of faces and not emotion recognition per se.   - Segregated - Lorenc**^30,31^** (2018, A*: 0.42), Pallathra**^41^** (2019, A*: 0.12); 6 studies | **ASD**   - N=146 (76 IGs, 80 CGs) - Age, sex and countries nr consistently | **Length**: 5wks (2 studies), 10wks, 5-8 sessions, 8 sessions  Time: 30-60m/session ^164^ Faja 2008), 2 hrs/week^162,163^ (nr X2)  Nr^161^ | - NRCT-No CG^161,166^ - RCT – CGs not described^162-165^ | | **Social skills and functioning**   - The STAART program improved face recognition;^165,166^ however 64% of participants reported no change in social functioning ^166^   Note: results nr for three studies,^162-164^, and considered not reportable for Newey^161^ by Lorenc**^30,31^.** | | nr | Limited effects and unable to determine due to lack of reporting  Low quality |
| *10b* | Interventions specifically targeting *Theory Of Mind* (Mazza, et al. ^167^, Bechi, et al. ^168^; both MQ), *Training in Affect Recognition* (TAR: Habel, et al. ^169^, LQ; Wölwer, et al. ^170^, MQ; Wölwer and Frommann ^171^, HQ; Sachs, et al. ^172^, MQ), *emotion perception* (Combs, et al. ^173^, MQ) or *vigilance for social perception* (Corrigan, et al. ^174^, MQ); *recognition training for facial affect* (Popova, et al. ^175^, MQ; Penn and Combs ^176^, LQ) or *micro-expressions* (^177^, LQ).   - Segregated - Grant**^22^** (2017, A*: 0.46); 11 studies | **PSD** (schizophrenia)   - N=495 (264 IGs, 211 CGs) - M=25-44 years - 64.1% male - Countries nr | 1 X 1 hr session^174^  12 sessions 45-60 mins^169-172^  20 daily 1hr sessions^175^  2 weekly 1hr sessions X 18 weeks^168^  nr^167,173,176,177^ | - RCTs - TAU CGs^169,170,172,175^ - RCTs - active CG: cognitive remediation,^171^ vigilance training,^174^ FEIT,^173^ discussion group,^168^ problems solving group,^167^ repeated exposure^177^ or practice without feedback^176^ | | **Social functioning**   - ↑ social or occupational functioning^167,171^   **Social skills**   - +/o social perception knowledge: ↑^171^ or no ∆^174^ - ↑ theory of mind^167,168,171^ - ↑ affect recognition^167,169,170,172,173,175-177^ | | nr | Social functioning: positive effects  Social skills: mostly positive effects  Quality: 3 low, 7 mod, 1 high |
|  | *Vocational social skills training* |  |  |  | |  | |  |  |
| 11 | The *Empowerment of Mental Illness service users: lifelong Learning, Integration and Empowerment* (EMILIA) project creates occupational opportunities for mental health service users as trainers, educators, researchers, auditors and direct service providers in user-led services and mainstream services (^178^@@author-year and Ramon, et al. ^179^, ?Q). Training includes empowering people in recovery, providing family network support, developing personal development plans, mental health treatment, building social competences (work related) and networks, and focusing on strengths. A *Vocational Rehabilitation Program* provided a 6-month internship in one of 42 participating companies (Bio and Gattaz ^180^, MQ).   - Vocational/integrated (setting nr X1) - Webber**^57^** (2017, A*: 0.15), Puolakka**^45^** (2019, A*: 0.42); 2 studies | **PSD** (schizophrenia, psychosis, bipolar disorder, or mental health problems)   - N=112 - M=28-31 years (nr X1) - 64% male (nr X1) - Brazil (nr X1) | Nr except for the 6m internship program which did not specify frequency or hours worked | - Qualitative^178,179^ - RCT – CG TAU^180^ | | **Social functioning**   - Most people had ↑ social life, social contacts and networks, but maintaining relationships was difficult^178,179^   **Other**   - ↑ QoL post-internship (G=0.21, p=0.036)^180^ | |  | Social functioning: Overall positive effects but limited and potential for negative effects  Mod quality (nr X1) |
| 12 | Social skills training for people with ID for vocational settings in the *Walker Social Skills curriculum* (Walsh, et al. ^181^, HQ) that follows a curriculum and uses video modelling to increase social communication. Video instruction (Gibson and Carter ^182^, HQ) and covert job coaching interventions (Gilson and Carter ^183^, HQ) were used to explicitly prompt social interactions in vocational settings.   - Vocational setting - Louw**^32^** (2019, A*: 0.19); 3 studies | **ID** (severity nr)   - N=15 - R: 18-26 years - 60% male - Ireland, USA X2 | 3hrs per week for 20 weeks^181^++  Job coaches attended a 90m training session and had brief (~10 min) weekly check-ins, participants worked internship 4-8hrs each week^183^++  Nr (v) | - Multiple baseline case design^182,183^ - NRCT-No CG^181^ | | **Social functioning**   - Increased social interactions, maintained post-intervention^182,183^   **Social skills**   - ↑ social competence and ↓ problem behaviour,^181^ including ↑ peer, adult and self-related social skills for all cases^181^   **Other**   - Maintained task engagement^182^ | | Nr | Social skills and functioning: Positive effects  3 high quality |
| 13 | The *Aspirations Program* provides social and vocational skills education and support using video-taped role-play; assistance to find employment; coaching on-site; follow-up support; and discussion groups Hillier, et al. ^184,185,186^. *Aspirations* topics include employment goals, friendship development, skills for navigating social gatherings, and general problem solving.   - Vocational/Integrated - Atkinson-Jones**^2^** (2019, A*: 0.15), Lorenc**^30,31^** (2018, A*: 0.42), Pallathra**^41^** (2019, A*: 0.12), Palmen**^40^** (2012, A*: 0.27), Tobin**^55^** (2014, A*: 0.38); 3 studies | **ASD** (one study included people with ASD & PSD; all excluded ID)   - N=71 - M=19-22 years - 85.8% male - USA | **Frequency & length**: varied, until they had independence in their job (1 day to 6m)  **Time**: 4-20 hrs/wk | - NRCT-No CG ^184-186^ | | **Social functioning**   - Non-significant ↑ in index of peer relations ^185,186^ and socialisation scale ^184^. Anecdotal increase in peer relations, and enjoyment meeting and interacting with others on spectrum^185^   **Social skills**   - ↑ empathy quotient post-intervention^185^   **Other**   - ↑ “skills” from 3m to 12m^185^ - ↓ depression and anxiety symptoms^184^ | | nr | Social functioning: no measured effect  Empathy: positive effect  Low quality |
| 14 | Vocational social skills programs that include supported employment and *supportive employment interpersonal skill training* using role play, cue cards and reinforcements (Shields-Wolfe and Gallagher ^187^, LQ); computer-based training in theory of mind, job interviews, and conversational skills (*JobTIPS*; Strickland, et al. ^188^, LQ), *Supported Employment, Comprehensive Cognitive Enhancement, and Social Skills* (SUCCESS; Baker-Ericzén, et al. ^189^, LQ) and *Workplace Training Program* (Liu, et al. ^190^, ?Q) provided social and cognitive and problem solving skills training. Explicit training in *social and behavioural skills* for vocational social behaviours in Mascot role (Allen, et al. ^191^, MQ). In Burke, ({Burke, 2010 #179@@author-year, LQ), Mascot training involved Behavioural Skills Training (BST; instruction, live and video-modelling practice, feedback, homework and practice log) followed by PDA-based, performance cue system (PCS)   - Vocational/Integrated - Atkinson-Jones**^2^** (2019, A*: 0.15), Bundock^7^ (2017, A*: 0.42), Lorenc**^30,31^** (2018, A*: 0.42), Pallathra**^41^** (2019, A*: 0.12), Palmen**^40^** (2012, A*: 0.27); 7 studies | **ASD** (low/average IQ in 2 studies, all others IQ/function in typical range)   - N=153 - M=20-25, R: 18-27 years - 85.4% male (nr X2) - Country nr consistently | **Length**: 1 session, 10 days, 2-6 sessions, 25 sessions, 8wks, 6m  **Time**: had 6-16mins X1 session (Allen) up to 1-1.5hr/ session per week ^189,192^ daily support for 6m ^190^ or 10 X 3-4 hr sessions^187^  nr^191,193^ | - Multiple baseline design^187,191,192^ - NRCT-No CG^189,190^ - NRCT – CG not described^193^ - RCT - “no intervention” CG^188^ | | **Social skills/behaviours**   - Social behaviour mastery reached very quick, ^191^ or after BST only (n=1), BST plus PCS (n=3) or with additional BST (n=1) ^192^ - ↑ initiating/responding to social greetings^187^ and social interaction/comprehension/expression^190^ - ↑ workplace social behaviours (e.g., appearance, supervision and appearance)^190^ - ↑ adaptive behaviour^193^   **Other**   - ↑ work rate and accuracy^187^ - Employment rates doubled, however, intervention was embedded in broader vocational program so may not be due to social skill training^189^   Note: results nr for Strickland, et al. ^188^ | | - The NICE (2016) guidelines recommend social skills groups as first‐line treatment for people with ASD and mild-mod ID. | Social/ vocational skills and employment: Positive effects  Quality: 5 low, 1 mod (nrX1) |
| 15 | *Job interview skills training* using virtual reality for ASD (Smith, et al. ^194^, MQ) and the *Molly Porter Job Interview VR training program* for mental illness (Humm, et al. ^195^, ?Q). Topics include creating a resume, finding and choosing a job that meets needs and preferences, dressing for an interview, asking appropriate questions, deciding whether or not to disclose a disability, and appropriate follow- up steps. A *Social Skills Curriculum* provided targeted instruction on social-vocational skills, job interview-related social skills (e.g., answering interview questions, conveying oneself as dependable, closing the interview, and following-up with interviewers (Morgan, et al. ^196^, LQ).   - Vocational/Integrated - Fernandez-Sotos**^13^** (2020, A*: 0.19), Lorenc**^30,31^** (2018, A*: 0.42), Pallathra**^41^** (2019, A*: 0.12); 3 studies | **ASD** (all studies), one study also included PSD (schizophrenia, PTSD)   - N=150 (92 IG, 54 CG) - M=24-25 - 85.8% male (nr)^195^ - USA | 5 sessions for total 10 hrs^194,195^  1.5 hr/week for 12 weeks^196^ | - RCT – TAU or waitlist CG ^194,195^ or “no intervention” ^196^ | | **Social skills**   - ↑in interview skills in IG vs CG,^196^ but no ∆ interview performance (g=0.35, 95%CI: -0.45, 1.15^+^)^194^ - No ∆ self-confidence (g=0.46, 95%CI: -0.34, 1.26)^194^, social pragmatic scale (g=0.44, 95%CI: -0.37, 1.25^+^)^196^ or adaptive behaviour (g=0.19, 95%CI: -0.62, 0.99) ^196^ - improvement in roleplay interviews and self-evaluation ^195^   **Other**   - Depression: g=0.28 (-0.52, 1.09)^196^   ^+^ Effect ns, but SRs indicate significant between group difference favouring intervention | |  | Interview skills: mixed  Social skills/ confidence/ depression: no effect  Quality: 2 low, 1 mod |
|  | *Friendships and dating training* |  |  |  | |  | |  |  |
| 16 | Various dating, sex and relationship skills group-based programs, including the *Ready for Love* Relationship Enhancement program for people with ASD and no ID (Cunningham, et al. ^197^, MQ), which assumed some experience in relationships. Other group-based friendship and dating group programs for people with ID include the *Friendships and Dating Program* (Ward, et al. ^198,199^, MQ), Early Dating Skills Training (Hayashi, et al. ^200^, LQ), *Dating Skills Program* (Valenti-Hein, et al. ^201^, MQ), *Not a Child Anymore* (Rushton ^202^, LQ), and *Curriculum Positive Choices* (Graff, et al. ^203^, LQ). The *Living Your Life* (Dukes and McGuire ^204^, LQ) program is delivered individually.   - Segregated - Bigby^4^ (2018, A*: 0.46), Exell**^12^** (2020, A*: 0.38), Gonzalvez**^21^** (2018, A*: 0.47), Howarth**^24^** (2016, A*: 0.38), Lorenc**^30,31^** (2018, A*: 0.42) , McCann**^36^** (2019, A*: 0.46), Pallathra**^41^** (2019, A*: 0.12), Sala**^49^** (2019, A*: 0.31); 7 studies | **ASD with no ID**^197^ or **ID**/learning disorder (mod-sev ID)   - N=201 (IG/CG nr for 3/5 studies) - M=20, R: 18-60 years - 62% male, nr X4 - USAX2 (nr X5) | 1-2 X 1.5-2 hr sessions/week for a total of 8, ^197,200^ 10 ^204^, 20^198,199^ or 24^201^ sessions  3 sessions^202^ or sessions nr^203^ | - Multiple baseline design^204^ - NRCT-No CG^198,199^ - NRCT - CG not described^200^ - RCT - CG received same the same program but no content on flirting, identifying romantic interest, and asking a person on a date^197^ - RCT - CG not described^201-203^ | | **Social functioning**   - ↑ social responsiveness scale scores (Total: Ƞ^2^=0.15; social communication subscale: Ƞ^2^=0.13; Social motivation subscale: Ƞ^2^=0.19), but no ∆ in social provisions scale^197^ - ↑ social functioning in IG, but not in CG^202^ - ↑ of social network size by 2.34 people by end of intervention, but no change in “network composition” ^198,199^   **Social skills/knowledge**   - ↑ Dating and Assertion Questionnaire (Total: Ƞ^2^=0.20)^197^, and knowledge of dating^201-204^ - ↑ “liberal” attitudes towards dating behaviour (e.g., kissing and holdings hands, gay and lesbian relationships, values and morals relating to sexual intercourse, and less endorsement of secrecy surrounding sexual behaviour following intervention)^204^ - ↑ Empathy quotient (Ƞ^2^=0.12)^197^ - ↑ Social skills in IG (d=1.01) but no change in CG^200^ | | - The range of different elements across the interventions suggests poor consensus on what functions are important to dating and relationships. **^12^** - Limited support that relationships are formed between people with and without ID through befriending or community connections programmes. ^4^ | Social functioning, knowledge and skills: Positive effects  Quality: 4 low, 2 mod |
| *17a* | *Sex and Relationship Education* and *family planning* group-based programs for people with ID focus a range of topics including anatomy, puberty, reproduction, STDs, sexual intercourse, relationships, dating/romantic skills, safety/consent/abuse, self/other in sexuality and relationships and private/public appropriate/inappropriate behaviours (Gardiner and Braddon ^205^, Garwood and McCabe ^206^, Penny and Chataway ^207^, Robinson ^208^, Lindsay, et al. ^209,210^, Caspar and Glidden ^211^, Foxx, et al. ^212^, Plaks, et al. ^213^, Haseltine and Miltenberger ^214^; all LQ). Two studies included people with both ASD and ID (Mueser, et al. ^215^, Box and Shawe ^216^; both LQ)   - Segregated - Exell**^12^** (2020, A*: 0.38), McCann**^36^** (2019, A*: 0.46), Sala**^49^** 2019, A*: 0.31); 11 studies | **ID** (mild-moderate nearly all studies; nrX1) and ASD + ID in 2 studies   - N=340 - M=22, R: 12-51 years - Sex nr in 9/11 studies - Ireland, Australia (nr X9) | Programs included 6 X 1-2 hr^207^ or 2.5-3 hr sessions;^211^ 9 X 25-30 minute sessions;^214^ 10 X 2 hr sessions^206^++, 10 X 2hr sessions;^216^ 14-16 X 2.5 hr weekly sessions^205^++  10^208,213^, 12^215^++ or 30 sessions^212^ but session length nr; or ran over 9m but session length nr^209,210^ | - Descriptive cross-sectional^206^ - Multiple baseline design^212,214^ - NRCT-No CG^207,211,213,216^ - RCT – CG not described^208-210,215^ - Qualitative^205^ | | **Social functioning**   - ↑ social entertainment and understanding of friendship^213^ - ↑ opposite-sex interactions in naturalistic observations^215^   **Social skills**   - ↑ dating problem-solving skill performance^215^ - ↑ knowledge of sexuality and rights/responsibilities,^205^ sexual vocabulary (program completion: d=3.26; follow up (d=1.83;^207^ d=2.94)^211^, and more liberal responses about sexuality topics (e.g., dating and sexual orientation; ^209,210^ d=1.82)^211^ - +/o knowledge: ↑ knowledge (d=1.07)^208,216^, including both sexual knowledge/behaviour and friendship knowledge;^213^ or no ∆^215^ - Learned all targeted social skills and ↑ social skills in a generalization test (30% correct at baseline to 55% post-training^212^ - 5/8 people learned self-protection skills after training, 2 required further feedback & 1 did not reach criterion of learning; 6/7 maintained skills at 1-month and 1/7 reached criterion again with further feedback^214^ | | - Practitioners providing SRE need to be self-aware and prepared to challenge prevailing attitudes, assumptions and stereotypes about relationship skills, knowledge, interests etc. of people with ID.^205^ - People with ID can be demonstrate unexpected inconsistencies in knowledge, and know more or less than they seem to, so simplifying language, reading questions aloud and probing may be needed.^206^ | Social functioning: Positive effects  Social skills for dating, relationships and sexuality: mostly positive effects  11 low quality |
| 17b | *Sex and Relationship Education* and *family planning* individual programs for people with ID that focus on similar topics to the group programs for people with ID or ASD. Programs include Family Planning (Zylla and Demetral ^217^, McDermott, et al. ^218^; both LQ), *Being Female: My body My responsibility* (Wells, et al. ^219^, LQ) and *Socialization, Training, Education and Parenting services (STEPS)* (McDermott, et al. ^220^, LQ) programs.   - Segregated - Sala**^49^** (2019, A*: 0.31); 4 studies | **ID** (mild-moderate severity)   - N=382 - M=32.6, R: 18-59 years - Sex and country nr | Programs included 1 X 1.5 hour session, ^219^ 24 1 hr sessions,^217^ up to 30 lessons selected for individual needs^218^  1 year of weekly home visits + 13 lessons^220^++  Session length nr | - Multiple baseline case design^217^ - NRCT-No CG^219,220^ - RCT – CG not described^218^ | | **Social skills**   - ↑ knowledge (measure/domain nr) from 44% to 100%^217^ - ↑ sexual knowledge (d=0.43) and hygiene (d=-0.03), with women who took care of their hygiene, had some experience with sex, and had positive social interactions, having greater ↑ sexual knowledge at 1-year^220^ - ↑ on 7/9 “measures” (domain nr) from “being female” training^219^   **Sexual health behaviour**   - The STEPS program did not ∆ birth control use^218^ | | Nr | Relationships knowledge and attitudes: Positive effects  4 low quality |
| *18* | *Abuse prevention* training for people with ASD in individual (Dekker, et al. ^221^, Lumley, et al. ^222^; both LQ) or group programs (Lee and Tang ^223^, LQ). For people with ID, there is a behavioural skills training for sexual abuse prevention program (Miltenberger, et al. ^224^, Egemo-Helm, et al. ^225^; both LQ) or the *ESCAPE curriculum* (Hickson, et al. ^226^, Khemka ^227,228^; all LQ) teaches women with ID to resist sexual, physical and verbal abuse using effective decision-making strategies.   - Segregated - Gonzalvez**^21^** (2018, A*: 0.47), Sala**^49^** (2019, A*: 0.31); 8 studies | **ID** (mild-moderate), ID and ASD^222,223^ or **ASD** without ID^221^   - N=175 (117 IGs, 58 CGs) - M=35-39, R: 11-57 years - 50% male,^226^ 100% female^227,228^ ^223^ (nrX4) - USA X3, China (nr X4) | Programs included 2,^223^ 3,^225^ 5,^222^ 10^224,227^, 12,^226^ 18,^221^ 40^228^ sessions of 40-60 mins (time nr X4) | - Multiple baseline^222,224,225^ - NRCT – No CG^221,227^ - NRCT - attention control program CG^223^ or inactive CG^228^ - RCT - inactive CG^226^ | | **Social safety skills and knowledge**   - Behavioural skills achieved to criterion for nearly all participants with initial training, but some required in situ training and booster training to maintain criterion and generalise skills to other settings^222,224,225^ - No ∆ in Problem Awareness (SMD=-0.19, 95%CI: -0.71, 0.33), Overall Effective Decision Making (SMD=-0.44, 95%CI: -0.96, 0.08), and “Safe-Now” Effective Decision Making (SMD=-0.19, 95%CI: -0.71, 0.32)^226^ - ↑ knowledge,^221^ including ↑ knowledge of abuse concepts (SMD=-0.99, 95%CI: -1.69, -0.29), empowerment (SMD=-0.70, 95%CI: -1.38, -0.03) and self-decision making (SMD=-1.18, 95%CI: -1.89, -0.46), but no ∆ in stress management (SMD=0.15, 95%CI: -0.51, 0.80)^228^ - ↑ Social/interpersonal decision making and locus of control;^227^ SMD not clearly reported against measures) - No ∆ ability to differentiate appropriate touching requests (SMD=-0.33, 95%CI: -0.80, 0.13), but ↑ recognition of inappropriate (SMD=-0.55, 95%CI: -1.02, -0.08) touching requests^223^ - ↑Knowledge about sexual abuse (SMD=-0.58, 95%CI: -1.05, -0.11)^223^ - No ∆ fear of objects, people and situations (SMD=-0.10 , 95%CI: -0.56, 0.36)^223^ | | - Younger participants and those who found SRE program more difficult made biggest gains (Dekker) | Safety behaviour and knowledge: Mostly positive effects but ongoing booster may be needed to generalise and maintain knowledge and skills  Low quality (nr X4) |
| *19* | The *SexG* group-based interventions for men with PSD, including brief programmes comprising 6 sessions (Berkman ^229^, MQ; Linn, et al. ^230^, HQ) and enhanced programmes with 13-15 sessions (Susser, et al. ^231^, HQ; Berkman, et al. ^232^, MQ). These programs use videos, discussion and role play to increase knowledge of safe sex, sense of personal risk and responsibility, and building confidence and motivation to use condoms.   - Segregated - Kaltenthaler**^27^** (2014, A*: 0.77); 4 studies | PSD (serious and chronic mental illness)   - N=595 (305 IG, 290 CG) - M=37-40 years - 100% male - USA | 6 X 1hr sessions^229,230^  13 X 1hr sessions^232^  15 X 1 hr sessions^231^ | - NRCT - active CGs: a single^229^ or 6-session HIV/STD workshop^230^ - RCT - active CGs: 2 HIV/SD/condom use workshops^231^ money management group^232^ | | **Sexual health and behaviour**   - Sex risk index scores: No ∆^232^ or ↓,^230,231^ including ↓ in people who were sexually active at baseline^229^ | | nr | Sex risks: positive effects overall  Quality: 2 mod, 2 high |
| *20* | *Relationship and AIDS/HIV-prevention interventions* typically focused on enhancing knowledge about HIV transmission and prevention, motivation for behaviour change and strengthening behavioural skills and self-management training, use of problem solving and negotiation skills, condom use, and assertiveness. Programs were presented to men only (The National Institute of Mental Health Multisite HIV Prevention Trial Group ^233^, LQ), women only (Collins, et al. ^234^, MQ; Weinhardt, et al. ^235^, MQ) or to both men and women (Carey, et al. ^236^, MQ; Kalichman, et al. ^237^, MQ; Katz, et al. ^238^, LQ; Kelly, et al. ^239^, LQ; Malow, et al. ^240^, MQ; Otto-Salaj, et al. ^241^, MQ)   - Segregated - Kaltenthaler**^27^** (2014, A*: 0.77); 9 studies | **PSD** (serious and chronic mental illness or mental health problems, and substance use problems)   - N=1,268 (660 IGs, 419 CGs) - M=34-42, R: 22-59 years - 47.2% male - USA | 6-7 X 1.5hr sessions^233,239,240^ including 2 boosters 1 & 2 months later^241^  10 sessions^234-236^  4 X 1.5-2hr sessions^237,238^  nr^234^ | - NRCT – waitlist, “no treatment” or TAU CGs^235-238^ - NRCT - active CGs: 1 educational video,^233^ 60min education session,^239^ Health Promotion program^240,241^ - RCT - active CG: money management group^234^ | | **Sexual health and behaviour**   - No ∆ sex risk index scores at 3m and 6m, ^234^ or motivation^235^ - ↓ risky sex acts^233^ or ↑ safe sex communication^236^ - ↓ casual sex partners, number of partners^236,239^ - ↑ condom use or ↓ levels of unprotected sex^233,234,237,239^ and ↑ positive condom attitudes,^236^ or no ∆ unprotected sex^239^ - ↑ protected sex at 2m but not at 4 months, and no ∆ frequency of unprotected intercourse^235^ - ↑ knowledge and intention to change risk behaviour^237^ - Knowledge about AIDS: ↑ at end of treatment and 2w^238^ or in men^241^ or no ∆ in knowledge^240^ - ↑ confidence to deal with high-risk situations, and coping in high-risk situations^238^ - ↑ sexual assertiveness at all periods^235^ | | Nr | Risky sex behaviours: overall positive effects but not always maintained  Quality: 3 low, 6 mod |
|  | *Life skill focused interventions* |  |  |  | |  | |  |  |
| *21* | *Life Skills Training* (Tungpunkom and Nicol ^242^, Almerie, et al. ^243,244^; all LQ) for people with schizophrenia or psychosis*,* and the group-based *Functional Adaptations and Skills Training* (FAST; Patterson, et al. ^245,246^, both MQ), which focused on six areas of everyday functioning: medication management, social skills, communication skills, organization and planning, transportation and financial management.   - Probably segregated (not clear) - Morin**^37^** (2017, A*: 0.19), Puolakka**^45^** (2019, A*: 0.42); 3 SRs & 2 studies | **PSD** (long-term psychotic disorders, schizophrenia, schizoaffective disorders)   - N=272 (140 IG, 132 CGG; nr 3X SRs) - M=48-52 years - 65.4% male - USA X2 (nr SRs | 2 X 2hr sessions/wk for 12 wks  (nr for SRs) | - SRs^242-244^ - RCT - services randomised to receive the intervention or not^246^ | | **Social skills**   - ↑ social skills vs TAU, but low quality evidence^242-244^   **Other**   - No ∆ in “Quality of Wellbeing” from FAST groups^246^ - ↓ relapse rates vs TAU, but low quality evidence^242-244^ | | nr | Social skills: positive effects  QoL: no effect  Quality: 3 low 2 mod |
| 22 | Programs for mothers or parents, delivered one-on-one, to build parenting skills (Feldman, et al. ^247^, HQ), and training to enhance knowledge and skills to manage home dangers, accidents, and childhood illness using a booklet and home visits (Llewellyn, et al. ^248^, MQ). One study provided a manual to mothers with reading and comprehension ability without further prompting (Feldman and Case ^249,250^; ?Q). The program by Mildon (2008^251^, ?Q) identified priority target skills for each family and tailored the modules to those needs. The *curriculum-based CARE (Care, Attunement, Responsiveness and Empathy) to Parent Program* of parenting skills (Brisson ^252^, ?Q) sought to strengthen mother-child relationships by enhancing the attunement of mothers to their school aged children. The *Support to Access Rural Services* (STARS) program was the only group program for mothers to meet regularly and learn interpersonal skills, information about disability, cultural sensitivity, community liaison skills and setting realistic expectations (Keltner, et al. ^253^, MQ).   - Segregated - Coren**^9-11^** (2011, A*: 0.85), Wilson**^60^** (2014, A*: 0.04); 6 studies | ID (severity nr)   - N=155 - M=26-42, 16-49 years - 95-100% women^247,249,250,252,253^ & 11-21% male - Australia X2, Canada, USA (nr X2) | 4 X 2-3hr modules over 2-3 weeks^252^  5 X 2hr home visits^247^  10,^248^ or 10-26^251^ X 1-1.5 hr sessions. | - Multiple baseline design^249,250^ - NRCT-No CG^251,252^ - RCT with waitlist CG^247^ - RCT with active CG: monthly phone calls and referrals^253^ - RCT with 2 CGs: (1) TAU, (2) active CG with lesson booklets without further training^248^ | | **Social and parenting skills**   - ↑ child care skills from baseline to post-training: 63 to 88% correct for IG but 65 to 61% for CG, with skills maintained for 90%^247^ - Self-instruction effective for 18/20 participants on 11 key skills, and the remaining parents required additional practitioner led training^249,250^ - Skills ↑ from 39% at baseline to 72% at post-training and 86% at follow up (Study 1), and 56% at baseline to 79% and 90% at post-training and follow up, respectively (Study 2)^249,250^ - Child health: ↑ Health comprehension (SMD=-0.70, 95% C: -1.29, -0.11), ↑ Life-threatening emergencies (SMD=1.95, 95%CI: 0.46, 3.44); no ∆ Going to the doctor: (SMD=0.65, 95% CI -0.06, 1.36), ↑ using medicines: (SMD=1.15, 95% CI: 0.51, 1.79)^248^ - Home safety: ↑ recognizing dangers (SMD=20.55, 95%CI: 13.72, 27.38), ↑ identifying precautions (SMD=31.75, 95%CI: 20.36, 43.14), no ∆ home precautions (SMD=7.05, 95%CI: -5.45, 19.55)^248^ - No ∆ parental daily hassles from pre-post, but ↓in frequency of parenting daily hassles (d=0.45)^251^ - No ∆ behaviour problems in children, but ↓intensity of behavioural problems (d=0.55)^251^ - ↑quality of home environment (d=0.72)^251^ - No ∆ parenting sense of competence^251^ - Varied ∆ attunement: 2/3 families ↑ attunement and 1/3 family had no significant ∆ attunement^252^ - Improved responsiveness of children to the parent^253^ | | - Important to adapt the training materials to reading/comprehension abilities of participants^249,250^ and the priority topic areas for families^251^ | Parenting skills: positive effects overall  Quality: 2 mod 1 high (nr X3) |
| *23* | *Digital literacy skills* training to use email (Cihak, et al. ^254^, HQ), support from non-disabled peers to use blog-related technology in a University setting (McClimens and Gordon ^255^, LQ) and experiences with social media use (Shpigelman and Gill ^256^, HQ), including using accessible interface for Facebook (Endeavor Connect; Davies, et al. ^257^, HQ)   - Mostly segregated - Bigby^4^ (2018, A*: 0.46), Louw**^32^** (2019, A*: 0.19); 4 studies | **ID** (severity nr)   - N=67 - R: 18-23 years - Sex nr consistently - USAX3 (nrX1) | 6 meetings^255^++  Frequency & total duration nr, but used least-to-most prompting if a step not initiated within 10s^254^++  intervention duration nr^257^ or n/a^256^++ | - Multiple baseline case design^254^ - Mixed methods^256^ - Qualitative^255^ - Method not clear^257^ | | **Social functioning**   - Email showed promise as a communication strategy to ↓social isolation;^254^ however, while blogging did not ↑social capital per se, the social activity around the exercise was valuable^255^   **Social skills**   - People with ID visit Facebook < other Facebook users.^256^ - Using Endeavor Connect ↑ed number of tasks completed independently and ↓ed errors on Facebook^257^ | | - ↓ Facebook use related to the inaccessibility of digital interface and participant reading skills^256^ - People with ID need literacy support to use digital/social media platforms^254-257^ | Social function and skill: positive effects/ potential  Quality: 1 low, 3 high |
| 24a | Navigation skill training using multimedia and video on an iPod to learn to navigate a route (Kelley, et al. ^258^, ?Q), or classroom computer-based video instruction to learn a bus route (Mechling and O'Brien ^259^, ?Q)   - Segregated/Integrated - Lindsay**^29^** (2019, A*: 0.12); 2 studies | **ID** (moderate or nr)   - N=7 - M=19-22 years - 42.9% male - USA | Total of 17-24 mins;^258^  9 mins/session 2-3 days per wk^259^ | - Multiple baseline design^258,259^ | | **Travel/navigation ability**   - ↑ independent pedestrian navigation skills^258^ - ↑ bus route navigation skills with generalisation to an actual bus route and maintenance over time; however, people were always accompanied by another person so not clear if ↑ capacity for independent bus travel ^259^ | | nr | Travel ability: Overall positive effects, but limited evidence of independence  Quality nr |
| 24b | Learning *pedestrian navigation skills using virtual and augmented reality*, including *3D VIDIA VIRTOOLS programme* (Courbois, et al. ^260^, Mengue-Topio, et al. ^261^; both ?Q), a virtual maze (Purser, et al. ^262^, ?Q), and using augmented reality, google maps and paper maps (McMahon, et al. ^263,264^; both ?Q), and the *heads up navigator app* for iPhone, which combines google maps with augmented reality to enable real-time navigation prompting (Smith, et al. ^265^, ?Q)   - Virtual/Segregated - Lindsay**^29^** (2019, A*: 0.12); 6 studies | **ID** (Down Syndrome or condition/ severity nr) or ID and ASD   - N=119 ID, N=19 Williams syndrome, N=136 typically developing peers - M=19-29 years - 48.5% male (nr for N=86)   France, UK X2, USAX3 | 3 sessions ^263-265^  Total 30-60m^261^  Nr ^260,262^ | - Multiple baseline design ^263,264^ - NRCT – CG included people without disability^260-262^ | | **Travel/navigation ability**   - People with ID made more errors^261^ and required more trials to learn route than controls without disability^260^ or with Williams syndrome,^262^ but did exhibit flexible wayfinding behaviour; ^260^ however augmented reality improved travel planning time and navigation skills^265^ - ↑ errors for augmented reality versus google maps and paper maps;^263^ however augmented reality was the more effective condition for McMahon, et al. ^264^, where students required assistance to use the paper maps and made errors half the time with google maps. | | People with little experience with computers and computers games may find it more difficult to use VR and AR^260^  It is not clear if navigation skills in virtual environment translate to natural environment^261,262^ | Travel ability: Overall positive effects, but limited evidence of generalisation  Quality nr |
| 24c | Applications for without augmented reality included the *AssisT-OUT wayfinding mobile app* (Gómez and Ojala ^266^, Davies, et al. ^267^; both ?Q), which adapts route calculations, instructions and interface designs to user preferences, a desktop, a desktop application with visual assistant that combines photos, videos and verbal instructions (Stock, et al. ^268^, ?Q), and a personal digital assistant that prompts users to a set destination (Mechling and Seid ^269^, ?Q).   - Segregated - Lindsay**^29^** (2019, A*: 0.12); 4 studies | **ID** (Down Syndrome, moderate ID or cognitive disability)   - N=45 - 46.7% male - M=19-32 years - Spain, USAX3 | 1 session per route, for 2 routes^266^  3 X week^269^  1-2 months  frequency or time nr^267,268^ | - Multiple baseline design^268,269^ - Mixed methods – CG used google maps only^266^ - RCT – CG not described^267^ | | **Travel/navigation ability**   - Better effects for google maps than the AssisT-OUT application,^266^ using wayfinding application increased successful completion of bus route (73% correct vs 8% in CG)^267^ - Participants could independently use the devices, but rarely used the video prompts^269^ - ↑ use of public transportation^268^   **Other**   - no ∆ self-determination^268^ | | need to carefully consider developing other essential life skills related to transportation (e.g., literacy, time management, problem-solving and other cognitive process skills) before providing travel training interventions.^267^ | Travel ability: Overall positive effects, but limited evidence of generalisation  Quality nr |
| *25* | *Life Story Work*, which helps people w ID to communicate their life story/history and has been used when people move residential location or attend day centres (Bai, et al. ^270^, MQ; Hamilton and Atkinson ^271^, MQ)   - Segregated - Schepens**^50^** (2018, A*: 0.15); 2 studies | **ID** ( mild-moderate ID )   - N=71 (43 IG, 28 CG) - M=56,^270^ R: >60 years^271^ - Male & female (%nr) - Hong Kong, Ireland | 1 X 1 hr meeting + extended discussion with participant for further alterations^271^++  16 X 1.5–2 hr structured individual or group sessions^270^ ++ | - Qualitative^271^ - NRCT – CG not described^270^ | | **Social functioning**   - ↑ interpersonal relationships, rights, social Inclusion and self-determination^270,271^   **Other**   - +/o effects on emotional and physical wellbeing^270,271^ | | Nr | Social functioning: Positive effects  2 moderate quality |
|  | *Support to increase social network and participation, including peer support and friendships* |  |  |  | |  | |  |  |
| 26 | Strategies used to support convivial encounters in community settings (e.g., shops; Bigby and Wiesel ^272^; ?Q)   - Integrated - Bigby^4^ (2018, A*: 0.46); 1 study | **ID** (mild-mod ID)   - N=26 - Age and sex nr - Australia | Frequency/duration n/a for observational study^272^ | - Observational qualitative study (no CG)^272^ | | **Community Participation**   - Support Workers use Active Support methods to assist PWD (e.g., body language, “translating”) and community members (e.g., reassuring or educating community members about abilities of PWD) to interact^272^ | | Nr | n/a  Quality nr |
| 27 | Supporting choice-making for people with severe ID (Treece, et al. ^273^, MQ) and individualised support to people with PSD using asset-based approaches, enhancing social skills, setting goals and providing peer support (Newlin, et al. ^61^, SR, ?Q).   - Integrated and segregated - Roche**^48^** (2019, UR, A*: 0.19), Schepens**^50^** (2018, A*: 0.15); 1 study & 1 SR | **ID** (severe) & **PSD** (various MH problems)   - N=2 (nr for SR) - 69 years (nr for SR) - Sex nr - USA (nr for SR) | Frequency/duration n/a for observational study^273^ and SR^61^ | - Qualitative^273^ - Systematic Review^61^ | | **Social participation**   - ↑ social inclusion^273^ and connectedness^61^ - Social capacity - +/- self-determination effects^273^ - ↑ self-esteem and health in asset-based approaches^61^   **Other**   - ↓ depression for social skill interventions^61^ - ↑ QoL for social skill and peer interventions^61^ | | Choice-making is often hampered by caregivers influencing the PWD choices. | Social inclusion/ connectedness: Positive effects  1 mod quality (nr X1) |
| 28a | Primary care providers refer people with multiple chronic conditions (often MH conditions) to a “navigator” or *social prescriber program*(Moffatt, et al. ^274^, MQ; Brandling, et al. ^275^, HQ; Carnes, et al. ^276^, HQ; Dayson, et al. ^277^, MQ; Farenden, et al. ^278^, MQ; Vogelpoel and Jarrold ^279^, MQ; Kimberlee, et al. ^280^, MQ; Wigfield, et al. ^281^, MQ; Friedlie, et al. ^282^, LQ; The Health ^283^, LQ; ERS Research Consultancy ^284^, LQ; Baines ^285^, LQ; Age UK and Age Concern ^286^, LQ; Grayer, et al. ^287^, HQ; Loftus, et al. ^288^, MQ; Grant, et al. ^289^, HQ). The navigator typically assesses the person’s social and mental health needs, interests and preferences and links them to programs or activities in community organisations and services.   - Integrated + some segregated activities (e.g., support groups) - Pescheny**^42^** (2020, A*: 0.27); 16 studies | **PSD** (most included people with chronic conditions, loneliness, depression; one study was for people with psychosis)   - Participant number and sex nr - Age nr, but 2 programs only for older people - UK X 16 | All included an assessment and some cited “regular contact”  1 assessment followed by ≥1 support contact^289^  ≥ 1 prescriber sessions over 4-14m & linked to 0-5 services,^274^ followed by ≤3 follow ups from the navigator^285^  1-3 navigator appointments,^287^ or 1-5 consultations with 43% referred to community activities^282^  average of 4 sessions with navigator with average of 3 referrals to social & community services^278^  ≤6 sessions with social prescriber^276^  average of 7 referrals per person^277^  12-week programme with prescriber support^279,280,288^  ++  nr^275,281,283,284,286^ | - Qualitative interviews^274^ - Mixed methods^275-286^ - NRCT-No CG^287^ - NRCT - CG not described^288^ - RCT^289^ | | **Social Participation**   - ↑ friendship scale score^280^ - Social isolation/loneliness: ↓ ^274,275,277,279,280,282,283^ or no ∆^281^ - ↑ interactions beyond the program,^282^ and 60% of lonely participants had ↑ satisfaction with spending time with others^278^ - Perceived social support and social activity participation: no ∆ vs CG^289^   **Other**   - Learning new skills valued outcome for participants^274,277,279,280,282^ - ↑ self-esteem, self-value, confidence, feeling useful or worthwhile ^274,275,277-280,282-285^ - Mental health/wellbeing: ↑ ^274,277,280,283,284,289^, no ∆ ^281^ or did not analyse due to small sample ^286^ - General wellbeing/QoL: ↑ ^277,287,289^or no ∆^283^ - Ability to identify needs and act to improve wellbeing: ↑ ^275 ,278,282^ - No ∆ prescription medications^276,288^ | | One person who did not experience health benefit had lower confidence after the program  An assessment is enough for some people to make health-related changes, but others need continuous need-driven support from navigators  Trust important needed for behaviour change | Social isolation, interactions and wellbeing: Overall positive effects  Quality: 5 low, 7 moderate, 4 high |
| 28b | In the *Connecting people intervention* (Webber, et al. ^290^, ?Q), *Urban Project* (Barbato, et al. ^291^, ?Q) and *Social Network Intervention* (Terzian, et al. ^292^, LQ) mental health professionals worked with PWD to identify interests and social activities, and social, leisure and recreational linkages in local community. One study provided similar support in a residential setting (Middelboe ^293^, ?Q).   - Integrated - Ma**^33^** (2020, A*: 0.50), Anderson**^1^** (2015, A*: 0.58), Webber**^57^** (2017, A*: 0.15); 4 studies | **PSD** (schizophrenia, mental health problems and SMI)   - N=357 (IG: 172, CG: 173; nr for 3 studies) - Age and sex nr - Italy X 2, UK X 1, nr X 1 | Staff at health services received 2 days of training and then provided the intervention to clients^290^ ++  Support provided for 3-6m^292^ or over 12-18m^291^ through existing MH service access++  n/a for observational prospective study^293^ | - NRCT – no CG^291,293^ - RCT – TAU CG^292^ or CG not described^290^ | | **Social Participation**   - 45.5% ↑ social network size,^292^ ↑ social networks links and community participation^291^ [vs CG] - ↑ reciprocal contacts in social network, but no ↑ in total network size or number of friends^293^ [no CG] - Access to social capital and perceived social inclusion ↑^290^   **Social Skills**   - ↑ interpersonal skills^291^   **Mental health**   - No effect on psychiatric symptoms or hospitalisation^292^ | | Teams need to share knowledge of community connections to optimise success of intervention. | Social networks: Positive effect  1 low quality (nr X 3) |
| *29*a | *Befriender intervention*, with volunteer from the community matched with the person with PSD, received €20 monthly stipend, and undertook social or leisure activities of interest to the person with PSD (Sheridan, et al. ^294^, ?Q).   - Integrated - Webber**^57^** (2017, A*: 0.15), Anderson**^1^** (2015, A*: 0.58); 1 study | **PSD** (schizophrenia, schizo-affective disorder, depression, bipolar disorder)   - N=107 (52 IG, 55 CG) - Age & sex nr - Ireland | 2 hrs/wk for 9 months | - RCT - CG received €20 monthly stipend only. | | **Social participation**   - Both IG & CG had similar ↑ social functioning and↓ loneliness. - Non-significant 7% ↑ in social network size at 2 years in IG | | Nr | Social function/ loneliness: no effect  Quality nr |
| *29b* | *Befriender intervention*, with volunteers from the community (Heslop ^295^, LQ) or supported living setting (Hughes and Waldenm ^296^, LQ) and trained to support/ accompany people with ID or to participate socially, develop friendships and increase their social network size.   - Integrated - Bigby^4^ (2018, A*: 0.46), Howarth**^24^** (2016, A*: 0.38); 2 studies | **ID** (severity nr)   - N=38 ID, N=42 befrienders, N=15 staff, N=46 parents/carers - Age & sex nr - UK X 2 | Weekly meetings to prepare people with ID followed by befriending visits (frequency nr) ^296^++  Nr^295^++ | - Descriptive case series (no CG)^296^ - Qualitative interviews (no CG)^295^ | | **Social Participation**   - Limited effects on participation in the community; most activities were home-based, and people with ID had limited choice and control^295^ - Only 1/4 participants in the case series increased their social network size^296^ | | - Potential negative impact on existing social networks - Difficult to recruit, train and retain volunteer befrienders | Participation: Limited effects  Low quality |
| 30a | Consumer *Buddy Care* intervention for homosexual adults with PSD to enlarge social networks and self-management (Fokkema and van Tilburg ^297^, ?Q); or group meetings in integrated community settings (Bøen, et al. ^298^, LQ). Two studies provided support for socialisation from consumer peers versus non-consumer peers, case management and crisis care, and support to attend activities (Rivera, et al. ^299^, MQ; Solomon and Draine ^300,301^, LQ).   - Clinical; integrated and segregated - Ma**^33^** (2020, A*: 0.50), Masi**^34^** (2011, A*: 0.34); 4 studies | **PSD** (Mental health diagnoses, depression, psychotic, mood disorders)   - N=489 (IG/CG nr) - M=67 (1 study), Age>18 - 60% male (2 studies nr) - USA X 2, Norway, nr X 1 | 35-38 sessions X 3 hrs each^298^  nr ^297,299-301^ | - NRCT - CG not described^297^ - RCT – TAU CG^299^ - RCT – Active CG: non-consumer support^298,300,301^ | | **Social participation**   - No effect on loneliness (effect=0.00, 95%CI: -0.55, 0.55)^297^ - Both groups had an increased level of social support;^298^ Ma**^33^** reported that there was greater improvement in the intervention group than the control group (d=0.12, 95%CI:−0.47, 0.81), but confidence interval shows this was not significant - Consumer support vs non-consumer support had ↑ social contacts at 12m (effect size=0.11)^299^ but no ∆ social networks^300,301^ - Group meetings ↑ social support at end of program in IG vs CG (d=0.12, 95% CI: − 0.47, 0.81).^298^   **Mental health**   - Peer group had ↑ mental health,^299^ but no ↓ psychiatric symptoms or service use^300,301^ | | nr | Loneliness: no effect  Social contacts/ support: positive effect  Quality: 2 Low 1 mod (nr X1) |
| 30b | In the *Friends Intervention* Mental health professionals met with friends of person with PSD to educate about PSD and re-establish shared activities; plan support and discuss emotions (Harrop, et al. ^302^, ?Q).   - Clinical; integrated and segregated - Webber**^57^** (2017, A*: 0.15); 1 study | **PSD** (psychosis)   - N=nr - Age nr - Sex nr - Country nr | nr | - Descriptive study with no CG | | - Social Participation - Successfully increased contact with friends and re-established social networks | | nr | Friendship: Positive effect  Quality nr, (appears low) |
| *31* | *Peer support* programs for people with ASD (Jantz ^303^, HQ), ID (Wilson, et al. ^304^, MQ) or PSD included programs with web-based *peer support* with most including elements of education (Kaplan, et al. ^305^, MQ; Kaplan, et al. ^306^, ?Q; Proudfoot, et al. ^307^, ?Q; Alvarez-Jimenez, et al. ^308^ and Gleeson, et al. ^309^, ?Q), or face-to-face programs that included MH profession facilitators, treatment as usual and case or crisis management (Castelein, et al. ^310^, HQ; Rivera, et al. ^299^, ?Q; Gammonley and Luken ^311^, ?Q), and segregated group membership in support groups to discuss past experiences and feelings (Rosen and Rosen ^312^, ?Q).   - Segregated - Anderson**^1^** (2015, A*: 0.58), Louw**^32^** (2019, A*: 0.19), Ma**^33^** (2020, A*: 0.50), Masi**^34^** (2011, A*: 0.34), Naslund**^38^** (2015, A*: 0.42), Walker**^56^** (2013, A*: 0.15), Webber**^57^** (2017, A*: 0.15), Tobin**^55^** (2014, A*: 0.38); 9 studies | **PSD** (schizophrenia/ psychosis disorders, SMI, bipolar, FEP, depression and trauma), **ID** or **ASD**   - N=855 PSD, N=35 ASD, N=10 ID - Age & sex nr (PSD) - R:24-77 years & 69% male (ASD) - R: 19-48 years & 30% male (ID) - Australia X3, Netherlands X1, USA X3 (nr X3) | 4wks, 8wks, 3m, 6m, 8m, 12m (nr X1) long  Total 24-30 hrs (1.5hr/ session), nr for 6 studies  2 hrs per session^312^  2 hrs p/wk^304^  nr^303^ | - Qualitative interviews^304^ - NRCT-No CG^303,308,309^ - NRCT – CG not described^311,312^ - RCT – waitlist CG^305^ or CG not described^307,310{Rivera, 2007 #258^ - RCT – active CG: lifestyle intervention^306^ | | **Social participation**   - Supported engagement fosters well‐being, and helps PWD develop social belonging and connectedness^304^ - PWD viewed support groups as a welcoming community where they could be themselves, share coping strategies, to fill free time, and to interact with others^303^ - ↑ social satisfaction and ability to get along with others post-group but no ↑ at 6m^311^ - No ∆ in social isolation/support vs CG at 4m and 12m ^305,306^ - No ∆ loneliness (effect=-0.59, 95%CI: -0.88, -0.30)^312^ - ↑ social relationships with peers but no ∆ with other friends/family ^310^, and no ∆ vs CG^299^ - Online peer networking platform had high acceptability with 95% of participants using it^308,309^   **Other:**   - QoL +/o effects: ↑ for people who regularly attended,^310^ and no ∆^305^ - Psychological wellbeing: ↓ depression,^308^ ↓parental stress,^306^ but no ∆ in wellbeing vs CG^307^ | | - People who had higher attendance at internet peer support had higher levels of distress.^305^ - Social benefits don’t appear to extend outside of peer group.^310^ - Groups need skilled facilitators, and to minimise participant turn-over^303^ | Social relationships/ satisfaction: Overall positive effects, but limited to peer group  Quality: 2 mod, 1 high (nr X6) |
| *32* | *Peer support programs* in statutory mental health services, and experiences from the perspective of peer support workers, their non-peer colleagues, and the recipients of peer support services were reviewed by Walker and Bryant ^56^ (?Q). While specific programs were not defined, this SR aimed to identify the “active ingredients” of peer support programs and services.   - Segregated - Walker**^56^** (2013, A*: 0.15); 25 studies | **PSD** (MH service users)   - N=340 PSD mentors, N=253 PSD mentees/ participants, N=138 staff - Age & sex nr - Australia X2, Canada X4, UK X2, USA X17 | Nr | - Meta-ethnography of qualitative studies | | **Positive/beneficial/rewarding themes:**   - **Peer support mentors with PSD**: being a peer support worker helps with your own PSD recovery, ↑ your social network, and can be a stepping stone to other opportunities. - **MH service users**: peer support workers are role models, and easier to build rapport with than non-peer staff because they have less professional distance, and are “street smart” (i.e., they know where a person would likely go after absconding from hospital, where a person’s money may go, and the effect of environment on drug use). Receiving peer support increases wellness (↑ hope & motivation, ↑ friends and social network, and ↑ illness management skills), and ↑ reintegration into the community by interacting with others, including non-peer staff, on an equal footing. - **Non-peer staff**: engaging with peer support workers with PSD offers unique learning opportunities - **MH services**: Peer support workers help people with PSD find a place in the community beyond being a “patient” | | Negative/challenging themes:  **Peer support mentors with PSD**: low pay, few hours, exclusion and rejection in the workplace (e.g., not invited to social work events), facing prejudicial attitudes from paid staff & being treated like a patient by clinical staff. There can be role confusion when asked to do multiple things (e.g., friendship, listener, lay expert)  **MH service users**: peer support workers are not always seen to be good role models as they don’t have “formal” training, *because* they have a PSD, or because service users believe they will be “ineffective helpers”  **Non-peer staff**: worry that their jobs may be replaced by the cheaper peer support roles because they offered cheaper labour.  **MH services**: peer support works take more sick leave than non-peer staff, and there can be tension about the professionalism of peer support workers.  **Other**: “black humour” was used by non-peer staff when talking about people in recovery during data collection, but was also expressed by some peer support workers | Positive and rewarding aspects for mentors and mentees with PSD, but important to address challenges for all stakeholders  Quality nr |
|  | *Transition programs (15 studies)* |  |  |  | |  | |  |  |
| 33a | Group *transition programs* for young adult transition to independence (Schneider and Hattie ^313^, HQ) including a *Camp Campus* before commencing post-secondary education (Retherford and Schreiber ^314^, LQ), and a one-on-one peer mentoring to support academic skills and goals (Ness ^315^, HQ).   - Integrated - Louw**^32^** (2019, A*: 0.19), Pallathra**^41^** (2019, A*: 0.12), Lorenc**^30,31^** (2018, A*: 0.42); 3 studies | **ID or ASD**   - N=79 - M=22, R: 17-31 years - Sex was nr consistently - Canada, USA (nr X1) | 7 days full-time^314^ or 10m  nr^313^ | - Mixed methods^313^ - NRCT-No CG^314,315^ | | **Social functioning**   - ↑ spending time with friends and participating in leisure (84% spent time with friends and 76% engaged in leisure activities in previous month) ^313^   **Other**   - ↑ grade point average^315^ - Results not clearly reported for Retherford^314^ | | Nr | Social functioning: positive effects  Quality: 1 low, 1 high (nr X1) |
| 33b | Peer mentorship programs for people with ASD transitioning post-secondary school (Ashburner, et al. ^316^, MQ; Hotez, et al. ^317^, MQ; Curtin, et al. ^318^, LQ), in college (Gillespie-Lynch, et al. ^319^, HQ; Ness ^315^, HQ) or university (Ames, et al. ^320^, HQ; Hamilton, et al. ^321^, MQ; Roberts and Birmingham ^322^, HQ; Siew, et al. ^323^, HQ), or in a setting not described (Martin, et al. ^324^, HQ)   - integrated - Nguyen**^39^** (2020, A*: 0.42); 10 studies | **ASD**   - N=131 people with ASD, N=82 mentors - R:17-33 years - Sex nr - Australia X4, Canada X2, UK X1, USA X4 | Nr | - Meta-ethnography of qualitative studies | | **Social participation and functioning**^315-324^   - ↑ social participation, ↑ friends with shared interests, and ↑ relationships with people with or without disabilities. The peer group gave opportunities to interact with mentors and mentee peers. - ↑ transition success by offering a safe space to be supported, to experience personal growth and to learn their strengths and areas for improvement. - ↑ practical life skills (e.g., time management) and ↑ self-advocacy skills (e.g., to access resources, request consideration), and had a safe environment to practice those skills with their mentors and peers. | | Mentor partnerships required clear communication and social connection to develop an open and comfortable relationship and to establish goals. | Social functioning: Positive effects  Quality: low X1, mod X3, high X6 |
| *34* | *Transition to retirement* from vocational programs for older people with ID (Bigby, et al. ^325^, MQ; Craig and Bigby ^326^, MQ).   - Integrated - Bigby^4^ (2018, A*: 0.46), Schepens**^50^** (2018, A*: 0.15); 2 studies | ID   - N=17 older adults - M=56, R: 48-62 years - Australia X2 | people participated weekly in existing community groups for 5-10 months^326^ or for a period/frequency nr^325^++ | - Qualitative^325,326^ | | **Social functioning**   - +/o effects on interpersonal relationships, social inclusion, self-determination especially if not supported to maintain old social networks or build new networks^325^ - ↑ effect on intimate relationships, social inclusion and rights awareness^326^ - More positive effects for people with ID who had friendly dispositions and relatively good social skills^326^   **Other**   - +/o effects on emotional wellbeing^325^ | | nr | Social functioning: mostly positive, but support needed for social networks  Moderate quality |
|  | *Community group participation* |  |  |  | |  | |  |  |
| 35 | The review by Farrell (2009^327^, ?Q) examined the impacts of *volunteer work* where adults with mental health problems freely and regularly do activities for another person, group or organisation, other than family or friends   - Volunteering - Roche**^48^** (2019, UR, A*: 0.19) | **PSD** (schizophrenia, psychosis, bipolar disorder, or mental health problems)   - N, age and sex nr |  | - Systematic Review^327^ | | **Social functioning**   - Some studies report ↑ social inclusion and social ties, or opportunities for social engagement, but limited evidence^327^ | | - Evidence on volunteering is not conclusive in relation to the promotion of social inclusion, and data remains limited at best^327^ - Barriers and risks to volunteering include prejudice/stigma and strain from over-commitment. Moreover, volunteering could increase experience of exclusion if activities are regarded as low status or participants have lower confidence, or could negatively interfere with access to other income support programs^327^ | Quality nr |
| *36* | *Community linkage* for people with ID (Harlan-Simmons, et al. ^328^, MQ) and *Independence through Community Access and Navigation* (I-CAN; Snethen, et al. ^329^, ?Q) interventions for people with PSD match the PWD with community based recreation or interest-based activities.  Integrated *community inclusion interventions* for people with ID aimed increase social network size and inclusion in community settings through outings (Saxby, et al. ^330^, ?Q), or education, activity identification, matching and scheduling to the interests of the PWD (^331^, MQ).   - Integrated - Bigby**^3^** (2012, A*: 0.27), Bigby**^4^** (2018, A*: 0.46), Howarth**^24^** (2016, A*: 0.38), Webber**^57^** (2017, A*: 0.15); 4 studies | **PSD** (schizophrenia) or **ID** (severe ID or in supported living X 2)   - N=13 (nr for 2 studies) - Age, sex and country nr 1 study, “older” 1 study, R: 24-58 years (ID) - Sex & country nr | Approximately 30 hrs meetings with recreational therapist over 9-10-weeks^329^++  DSW staff had a 30min introduction, activity participation then measured at 6m^331^++  3 year duration (session number & length nr)^328^++  observational descriptive study (hrs n/a; Saxby)++ | - Qualitative (no CGs)^328,329^ - Descriptive cross-sectional,^330^ - NRCT-No CG^331^ | | **Social and community participation**   - Participants reported ↑ community involvement^329^ and positive social network impacts^328^ - ↑involvement in community activities,^330,331^ although low levels of substantive engagement in social activity and interactions in community settings even though proprietors were accepting of PWD^330^ - 49% ↑ social network size of non-paid people^331^.   **Cost impacts**   - Increasing frequency of target activities did not ↑ support costs^331^ | | - Interactions in shops and public facilities are only momentary^330^ | Participation: Positive effect  Belonging: Positive effect  Community activities & interactions: Mixed  2 mod quality (nr X 2) |
| *37* | *Community membership* support interventions through membership or attendance at mainstream *community groups* (e.g., Men’s Shed or other groups matching PWD interests; Wilson, et al. ^332^, ?Q; Bigby, et al. ^333^ and Stancliffe, et al. ^334^, LQ; Wilson, et al. ^335^, ?Q). Existing group members were trained to mentor the PWD.   - Integrated - Schepens**^50^** (2018, A*: 0.15), Bigby**^4^** (2018, A*: 0.46), Roche**^48^** (2019, UR, A*: 0.19), Petroutsou**^43^** (2007, A*: 0.46); 3 studies | **ID** (majority mild-mod severity & nr X2) and PSD (various)   - N=58 (+1 SR) - M=56-59 years - 72.4-100% male - USA & Australia | 1 day per week for 1-6 hours (mean = 3.6 hours; ^332-334^, or men’s shed experiences were evaluated for sheds that were open 1-3 days per week over 6-months^335^++ | - Mixed or multi-methods^332-334^ - Umbrella review**^48^** | | **Social participation**   - No ∆ loneliness^333,334^ - ↑ social satisfaction (Hedges g=0.83, 95%CI: 0.29, 1.36), and community group participation, time spent with new social contacts, and average new social contacts post-group (all p<0.001, stats nr)^333,334^   **Community participation**   - Community group ↑ acceptance of PWD, and mentors reported enjoying the experience^335^ - Men’s sheds offer opportunity for meaningful participation, adult learning, and health and wellbeing^332^   **Other**   - No ∆ depression, physical health or QoL^333,334^ | | - May need to be multicomponent, and to provide support outside group setting, to impact on loneliness. - Men less willing to attend Men’s shed if potential health benefits are the focus. | Relationships, inclusion, satisfaction, networks: Positive effects  Loneliness: no positive effect  1 low quality (nr X 2) |
| 38 | Segregated group membership in *day centres* (Temple and Walkley ^336^, ?Q)*.*   - Segregated - Hutzler**^26^** (2010, A*: 0.12); 1 study. | **ID** ( severity nr)   - N=40 + 31 staff/parents - M=25, R:18-58 years - 67% male - Country nr | Hours nr | - Qualitative focus groups | | **Social participation**   - Effects not reported | | - Predisposing (e.g. social support), enabling (e.g. skills) & reinforcing factors (e.g. rewards) must be addressed in program design | Quality nr |
|  | *Sports and physical activity participation* |  |  |  | |  | |  |  |
| 39 | Motivations and barriers to *physical activity and exercise* participation for people with schizophrenia (?Q)**^16^** experiences in starting community-based group physical activity for SMI (Quirk, et al. ^46^, MQ) or participating in physical activity, exercise or lifestyle programs (Soundy, et al. ^52^, HQ).   - Nr if integrated or segregated - Firth**^16^** (2016, A*: 0.34), Quirk**^46^** (2020, A*: 0.66), Soundy**^52^** (2014, A*: 0.46); 37 studies | **PSD** (FEP, schizophrenia, schizoaffective disorder, bipolar, major depression, affective disorders, psychosis, PTSD, SMI)   - N=6,466 PSD, N=80 clinicians - M=20-55, R: 19-67 years - 70.8% male (nr for N=6,272) - Australia X3, Canada X3, Italy, Sweden, UK X2, USA X4 (nr X9) | Nr for all but 5 studies that referred to 1-2 weekly sessions of 45 mins to 2hrs | - Qualitative methods analysed with meta-ethnography methods | | **Social and functional effects of physical activity participation**   - Positive aspects of participation included ↑ socialisation, flexibility with schedule or impact of fluctuations in symptoms, have a sense of control, ↑ mood, relaxation, sense of achievement and self-appreciation.**^46^** - ↓ weight was motivating and considered “a yardstick for recovery”, ↓ voices/hallucinations and other symptoms, ↑ sleep and positive socialisations.**^52^** - Physical activity ↑autonomy, ↑focus on future gains, ↑ confidence/self-esteem to engage in the community, led to encouraging peers to participate.**^52^** - ↑ social and emotional support between peers and from staff ↑ empathy, sense of warmth and companionship.**^52^** - Programs that have a sense of cohesion and that enhance relatedness between participants ↓ anxiety.**^52^**   **Factors impacting on participation in physical activity:**   - **Physical factors**. Motivators included to improve general physical health (N = 790, 6 studies; 91% agree, 95% CI 80–94), to lose weight (N=169, 3 studies; 83% agree, 95% CI: 54–99), to improve appearance (3 studies, N=465; 77% agree, 95% CI: 64–88), increasing fitness/energy (N=549, 5 studies; 75% agree, 95% CI 64.9–83.4). Being tired or having low energy was the most common physical barrier (N=6080, 5 studies; 45% agree, 95% CI 25–67), but on 25% endorsed physical health as a barrier.**^16^** - **Psychological factors**. Motivators included improving overall mental health (N=788, 6 studies; 80% agree, 95% CI: 62–93), managing mood (N=464, 3 studies; 81% agree, 95% CI: 62–93), reduce stress (N=520, 4 studies; 78% agree, 95% CI: 59–92), improving sleep (N=464, 3 studies; 72% agree, 95% CI: 55.6–86). The most common barriers was stress or depression (N=5646, 3 studies; 61% agree, 95% CI: 43–77), and <33% of people did not participate due to a ‘disinterest in exercise’, Feeling unsafe, fears of injury, low motivation, low confidence.**^16^** - **Socio-ecological factors**. There were no common socioecological motivators, with only 27% (95%CI: 23-32; N=452, 3 studies) endorsing the social aspect of exercise as a motivator. A lack of support was a barrier endorsed by 50% of people (95% CI 15–86; N=5646, 3 studies).**^16^** - **From qualitative research**: ↑ intent to initiate participation with prior positive experiences, felt “well enough”, the activity was affordable and in a location that was perceived to be appropriate for “people like us”, had positive expectations about the effects of participation (e.g., controlling symptoms, ↑ health & weight control, access to support, talking with others with similar experiences, seeing/making friends, a reason to get out of the house)**.^46^** | | - ↓ participation of people with PSD had: intrusive/fluctuating symptoms, fatigue, low self-esteem, social anxiety and apprehension to be around strangers, feeling dependent on others (e.g., needing reminders, transport/financial help, or needing a support person), negative expectations (e.g., feeling vulnerable, embarrassed, disliking feeling controlled by others, having to interact with others, pain), having conflicting personal commitments.**^46^** - Some people are hesitant to join groups and preferring one-one format, and can find new environments challenging.**^52^** - Lifestyle factors influence physical activity participation ( e.g., smoking, diet, sleeping patterns, fitness level and confidence).**^52^** - stability of symptoms and sedative effects of medication can be a barrier to participation.**^52^** | Generally positive effects on social interactions, but important to be aware of barriers and need for individual flexibility and accommodations  Quality: 4 low, 15 mod, 6 high (nr X12) |
| 40 | Effects of physical activity participation for people with schizophrenia. Activities included a *National Fitness Corps Programme for yoga* under supervision for 2-4 weeks followed by unsupervised practice (Duraiswamy, et al. ^337^, MQ; Behere, et al. ^338^, LQ; Varambally, et al. ^339^, MQ; Manjunath, et al. ^340^, MQ), soccer training and games (Battaglia, et al. ^341^, LQ), aerobic exercise (Acil, et al. ^342^, LQ), interval (Abdel-Baki, et al. ^343^, Heggelund, et al. ^344^, both ?Q) resistance (Marzolini, et al. ^345^, LQ; Scheewe, et al. ^346,347,348^, HQ) or strength training (Heggelund, et al. ^349^, ?Q) programs.   - Nr if integrated or segregated - Firth**^15^** (2015, A*: 0.41); 11 studies | **PSD** (FEP, schizophrenia)   - N=552 (269 IGs, 283 CGs) - M=25-45 years - Sex nr - Countries nr | 3 X week for 8-10 wks (total 60-75 mins/wk) ^342,344,349^  2 X wk for 12-14 wks for 40m,^343^ 1.5hr^345^ or 2hr^341^ sessions  2 X wk for 24 wks for 1hr per session^346-348^  5 X wk for 16 wks for 1hr per session^337-340^ | - NRCT - active CG: playing computer games^344,349^ - RCT - TAU or waitlist CG^338,339,341,342,345^ - RCT - active CG: OT ^346-348^ or exercise group/program^337,340^ | | **Social functioning**   - No ∆ psychosocial functioning^343^ - Yoga led to improved socio-occupational functioning by 17%^339^ and 23%^337^   **Other**   - The ‘high-intensity’ training interventions with short bursts of strenuous physical activity did not affect symptoms or functioning, despite improving physical strength/fitness^349^ - No ∆ mental health for low dose activity (<90 minutes/week; ^343,344,349^ but significant ↑ for programs with ≥90 minutes/week moderate-vigorous exercise^341,345^ - QoL: ↑ 10.6% with soccer training,^341^ ↑14% with aerobic exercise,^342^ No ∆ ^344,349^ - Depression: ↓ 56%,^340^ ↓21%,^337^ no ∆ ^342,344,345^; ↓ 36.6% vs 4.4% in CG (Scheewe) - Schizophrenia symptoms: No ∆ ^344,349^, no ∆ (SMD=0.31, 95%CI: -0.20, 0.83)^340^, no ∆ (SMD=-0.05, 95%CI: -0.52, 0.43)^339^; no ∆ (SMD=0.21, 95%CI: -0.34, 0.76);^338^ no ∆ positive (SMD=-0.40, 95%CI: 1.12, 0.33), negative (SMD=-0.70, 95%CI: -1.44, 0.04), or total symptoms (SMD=-0.52, 95%CI: -1.25, 0.21);^342^ 24% ↓ positive and 18% negative symptoms;^337^ ↓ 20.7% in psychotic symptoms vs ↑ by 3.3% in CG^346-348^ | | - Significant improvements in fitness, psychiatric symptoms and overall functioning only occurred in participants who attended >=50% of exercise sessions^346-348^ | Psychosocial functioning: Mixed effects  Schizophrenia symptoms: No effect in 6/8 studies  Positive effects in studies with more intense exercise and higher attendance  Quality: 4 low, 3 mod 1 high (nr X3) |
| *41* | *Integrated sports programs or physical activity* in the community (van Schijndel-Speet, et al. ^350^, MQ; van Schijndel-Speet, et al. ^351^, HQ). The *Creating a Sporting Chance Program* included team sports, exercise and active recreation (Lante, et al. ^352^, LQ). The *Walk Well Program* matched people with a walking adviser for regularly walks in the community (Matthews, et al. ^353^ and Melville, et al. ^354^, MQ), or with age-matched peers without disability for team sports (Bota, et al. ^355^, MQ).   - Integrated - Louw**^32^** (2019, A*: 0.19), Schepens**^50^** (2018, A*: 0.15), Bigby**^4^** (2018, A*: 0.46), Bondar**^5^** (2020, A*: 0.62); 5 studies | **ID** (mild to severe ID)   - N=356 (239 IG, 118 CG) - R: 11-83 years - Sex nr consistently - Netherlands X2, Romania (nrX2) | Nr for all but 2 studies, which referred to “3 semesters”^352^ and 3 consultations with walking adviser^353,354^ | - Descriptive case study^352^ - Mixed methods^355^ - Qualitative^350^ - RCT^351,353,354^ – CGs not described or “no intervention” | | **Social functioning**   - ↑social inclusion, positive self‐concept and self‐esteem perception^355^ - ↑ opportunities for social contact with other gym users^352^ - +/o effects on interpersonal relations: ↑,^351^ but +/o^350^   **Other**   - Participants enjoyed playing a new sport, ↑ health and fitness, ↑ praise and acknowledgement from others^352^ - ↑ emotional wellbeing^351^ - No ∆ QoL, self-efficacy or “subjective vitality”^353,354^ - ↑ physical activity levels^352^ and steps per day^353,354^ | | - People may not want to participate if they will miss out on other valued activities,^352^ and need activities to be matched with preference and ability^350^ | Social functioning: overall positive effects  Quality: 1 low, 3 mod, 1 high |
| *42* | *Unified Special Olympics (SO) programs* bring together athletes with ID and age and ability matched people without ID from the community to play on the same sport teams (Harada, et al. ^356^, ?Q). Two studies were based in USA (Riggen and Ulrich ^357^, MQ; Rosegard, et al. ^358^, LQ) and three examined SO across multiple European countries (McConkey, et al. ^359^, Wilski, et al. ^360^, Hassan, et al. ^361^, all ?Q).   - Integrated - Bigby**^4^** (2018, A*: 0.46), Tint**^54^** (2017, A*: 0.42), Hutzler**^26^** (2010, A*: 0.12); 6 studies | ID (severity nr)   - N not clear, included athletes with/without ID, parents, coaches community representatives - M=25-31, 11-68 years - Sex nr consistently - USA X2, Serbia, Poland, Ukraine, Germany, Hungary, Italy | nr | - Qualitative (^359-361^ - Descriptive cross-sectional^356,358^ - NRCT (CG people with ID not in SO)^357^ | | **Social functioning**   - ↑ friendship and community belonging,^360^ to build alliances within local communities,^359^ and giving a “chance to play sport… be a part of society... a platform for the development of social relationships"^356^ - ↑ social inclusion^361^ - ↑ access to community venues^360^   **Capacity**   - ↑ social self-perception/self-acceptance^357^ - ↓ maladaptive behaviours^358^   **Other**   - No ∆ physical health,^357^ but improved sport skills^360^ | | - The studies provide little evidence to substantiate claims that SO ↑ “access to the community” or development of “social relationships with their teammates” and whether these “carry over into their lives off the playing field” (p.1135-36)^356^ | Social functioning/ capacity: Positive effects  Low-mod quality (nrX4) |
| *43* | *Segregated exercise or physical activity programs* for people with ID including treadmill (Carmeli, et al. ^362^, HQ), balance (Carmeli, et al. ^363^, MQ) and/or strength (Carmeli, et al. ^364,365^ , both MQ), gym-based physical activity program (Tomporowski and Ellis ^366,367^, MQ and LQ respectively) and combined *physical activity and health education programs* (Pérez-Cruzado and Cuesta-Vargas ^368^, LQ; Heller, et al. ^369^, LQ; Marks, et al. ^370^, LQ).  Three studies in people with ASD evaluated exercise (Elliott, et al. ^371^, ?Q) or leisure programs (García-Villamisar and Dattilo ^372^, MQ) that included homework, skill development, behavioural practice (Palmen, et al. ^373^, ?Q).   - Segregated - Schepens**^50^** (2018, A*: 0.15), Bondar**^5^** (2020, A*: 0.62), Hutzler**^26^** (2010, A*: 0.12), Hallett**^23^** (2019, A*: 0.12), Tobin**^55^** (2014, A*: 0.38); 12 studies | **ID** (mild X4, mild-mod X2, mod-severe 2, down syndrome) & **ASD**   - N=448 ID (243 IG, 169 CG, nr N=12), N=89 ASD (43IG, 34 CG) - M=21-45, R: 13-77 years (1 study in older people with ID) - 56.3% male (nr X7 studies) - Israel X4 (nr X8) | 6 months^362,363^  40-60min/session 2X week for 8wks,^368^ or 3 X wk for 12wks^369,370^ or 10m ^365^  3 hr/day 5 X wk for 7m^366,367^ or 5 X 2hrs per wk X 12m^372^  (nr X2) | - NRCT-No CG^368,371^ - NRCT – “no intervention” CG^373^ or CG not described^364^ - NRCT – active CG: strength training,^363^ vocational training^365^ - RCT – “no intervention” or waitlist^369,370,372^ or CG not described^362^ - RCT – active CG: attention training,^366^ placebo pills ^367^ | | **Social participation**   - ↑ Interpersonal relations^362-365^ - ↑ in social support (professional support, family support, peers support)^368^ - ↑ social belonging^372^ - No ∆ community integration^369,372^ - No ∆ leisure activity support needs (g=0.58, 95%CI: −0.60, 1.76), leisure engagement (g=0.32; 95%CI: −0.84, 1.48) or satisfaction with leisure lifestyle (g=0.64; 95%CI: −0.55, 1.83)^373^   **Other**   - +/o adaptive behaviour: No ∆,^366,367^ but ↓ ‘‘maladaptive and stereotypic behaviours’’ after vigorous exercise^371^ - ↑ life satisfaction^369^ - ↑ QoL, self-efficacy,^368^ and psychosocial wellbeing^370^ - ↑ transition to employment^371^ - ↑ QoL and empowerment/independence and ↓ stress^372^ - Leisure program led to ↓er stress, and ↑ QoL^372^ | | - Within 6m of intervention ending people discontinued exercise, largely due to lack of transport and carer support, so ongoing support is needed^369^ - Understanding participant goals is important when delivering the group program^372^ | Interpersonal relations: Positive effects  QoL and psychosocial wellbeing: Positive effects  Community integration: no effect, but not measured by most studies  Quality: 4 low, 4 mod, 1 high (nr X3) |
| *44* | *Segregated Special Olympics* training (Lynnes, et al. ^374^, LQ) or participation in regional, national and international competitions for people with ID, with more than 30 Olympic-style individual and team-based sports (Tedrick ^375^, ?Q; Glidden, et al. ^376^, LQ; Válková ^377,378^, both LQ; Werner ^379^, ?Q; Marks, et al. ^380^, LQ)   - Segregated - Bondar**^5^** (2020, A*: 0.62), Bigby**^4^** (2018, A*: 0.46), Tint**^54^** (2017, A*: 0.42); 7 studies | ID (severity nr)   - N=181 ID, 101 parents, siblings, coaches or caregivers - M=21-32, R: 12-50 years - 52.4% male - USA X3, Czech Republic X2, Canada, Israel | Training: 3 X 1.5hr sessions per wk (2 at home) for 10 wks  Nr for competitions | - Qualitative^374,375,379^ - Descriptive cross-sectional^376-378^ or case study^380^ | | **Social functioning**   - ↑ social, self-perception, participation of people with ID^376^ - ↑ meeting people, friendships, mentoring others with ID^375^ - ↑ number of friends^380^ - ↑ community awareness and inclusion^379^ - ↑ social behaviour vs people with ID not in SO,^377,378^ and ↓ maladaptive behaviour^377^   **Social skills**   - ↑ communication skills in SO participants than non-participants (not clear if effect of participation or selection bias)^377,378^   **Other**   - No ∆ self-efficacy^374^ - ↑ happiness^376^ - ↑ physical condition/health^380^, fitness & sport skills^375,377,378^ - Enjoyment of planning, recreational games and music components of the programme^374^ | | - Parents overstated the emotional benefits relative to participants^376^ | Social functioning/ skills/ health: Positive effects  all low quality |
| 45 | Other evaluations of *Special Olympics programs* where the setting (i.e., segregated or integrated) is not described (Farrell, et al. ^381^, ?Q; Ghasemia, et al. ^382^, LQ; Goodwin, et al. ^383^, ?Q; Harada and Siperstein ^384^, ?Q; Weiss, et al. ^385,386^, both LQ; Wilhite and Kleiber ^387^, LQ; Shapiro ^388^, ?Q; Dykens and Cohen ^389^, ?Q; Frey, et al. ^390^, ?Q)   - Nr if integrated or segregated - Hutzler**^26^** (2010, A*: 0.12), Tint**^54^** (2017, A*: 0.42); 10 studies | ID (severity nr consistently)   - N=1247 ID, 746 others - M=22-37, R: 9-69 years - 49.6% male - Canada X4, Iran, USAX4 (nr X1) | Nr | - Descriptive cross-sectional^382,384,385,387-389^ - Descriptive case study^386^ - Qualitative^381,383,390^ | | **Social functioning**   - Having fun,^384^ exercising choice, making friends, receiving “social approval”^381^ or acceptance,^383^ and having social interactions^384,388^ are key reasons and benefits of participating - Social acceptance not predicted by changes in SO participation,^386^ but were predicted by the number of medals^385^ - ↑er community involvement (e.g. increased independence - in the community) for SO participants with severe ID, but ↓er for people with mild-moderate ID^387^   Social skills   - ↑ social competence associated with longer time participating in SO^389^ - Other - ↑er self-efficacy and interpersonal dependency than non-participants^382^ - Changes in SO participation associated with self-worth^386^ | | - People with ID dislike it when there are conflicts, disorganised rules or policies^381^ - Most common reasons for discontinuing are change of interests and programmes becoming unavailable^384^ - Barriers to SO participation include: lack of guidance, negative supports, mostly sedentary leisure-time choices^390^ | Social functioning/ skills: Overall positive effects  Low quality (nr X6) |
|  | *Animal interventions (9 studies)* |  |  |  | |  | |  |  |
| *46* | *A “visiting dog”* for walking in the community on convivial encounters (Bould, et al. ^391^, HQ).   - Integrated - Louw**^32^** (2019, A*: 0.19), 1 study | **ID** (severity nr)   - N=16 - R: 18-64 years - Sex nr - Australia | 14 X 1 hr sessions | | - Mixed methods | | **Social functioning**   - ↑ convivial social encounters & ↑ confidence to engage in social exchanges |  | Social functioning: positive effects  Quality: 1 high |
| *47* | *Animal Assisted Therapy*, including passive avian companionship in a residential setting (Jessen, et al. ^392^, MQ), walking dogs over obstacles, carrying them and playing fetch games in older people with schizophrenia (Chu, et al. ^393^, MQ), choosing a cat or dog which they could pet, feed, groom, bathe, and teach to walk on a lead (Barak, et al. ^394^, LQ).   - Segregated - Maujean**^35^** (2015, A*: 0.19), Kamioka**^28^** (2014, A*: 0.73); 3 studies | PSD (schizophrenia or depression)   - N=90 (45 IG, 45 CG) - Age & sex nr consistently - Israel, Taiwan, USA | 10 days of “continuous” companionship^392^  50 mins weekly for 2m^393^  3 hr per/wk for 1 year^394^ | - NRCT – TAU CG^392^ - NRCT – active CG: reading and discussion group without animals CG^394^ - RCT - TAU CG^393^ | | **Social functioning**   - No ∆ loneliness^392^ - No ∆ social support^393^ - ↑ social-adaptive functioning^394^   **Other**   - ↓ depression^392^ - Improved psychiatric and emotional symptoms^393^ - ↑ self-esteem and self-determination^393^ | | - 3/30 participants were afraid of the dog and did not interact with it^393^ - It is not clear if the positive effects were due to the animal, or simply having a structured activity^393^ | Social functioning: mostly null effects  Psychiatric symptoms: Positive effects  Quality: 1 low, 2 mod |
| *48* | *Group therapy programs that with or without animals*, including Integrated Psychological Treatment (IPT) with or without a dog, in which the therapy follows five hierarchical modules on cognitive differentiation, social perception, verbal communication, social skills training, and interpersonal problem solving (Villalta-Gil, et al. ^395^, MQ), or regular inpatient group therapy programs with rabbits, guinea pigs, dogs, ferrets brought into the room that participants could hold, pet or play with as long as they did not disrupt the group (Marr, et al. ^396^, LQ)   - Clinical/segregated - Anderson**^1^** (2015, A*: 0.58), Maujean**^35^** (2015, A*: 0.19), Kamioka**^28^** (2014, A*: 0.73); 2 studies | **PSD** (psychiatric inpatients and people with chronic schizophrenia unable to live in the community)   - N=61 (30 IG, 31 CG) - Age & sex nr consistently - Spain, USA | 2 X 45 min sessions per/wk for 25 wks^395^  nr clearly for ^396^“each day” | - NRCT – CG not described^396^ - RCT – CG had IPT with no dogs in the sessions^395^ | | **Social functioning**   - Patients in the AAT group interacted more with other patients, and had ↑ social behaviour (e.g., smiling and showing pleasure) over time^396^ - ↑ social competence^395^   **Other**   - ↓ psychiatric symptoms & ↑ QoL in IG ^395^ | | - It may be that longer duration treatment with AAI (>=25 sessions) is needed to achieve significant change^395^ - Compelling evidence that reductions in the persistent negative symptoms of schizophrenia could be attributed to the dog because an active control group was used^395^ | Social & psych functioning: Positive effects  1 mod & 1 high quality |
| *49* | *Farm animal assisted therapy* with the opportunity to work with a farmer in the cowshed, choosing work such as grooming, feeding, taking care of the calves, and milking (Pedersen, et al. ^397^, MQ), or to choose to pat, wash, groom a broader range of animals (cows, sheep, horses), or to ride the horses (Berget, et al. ^398,399^, ?Q).   - Segregated (non-disabled farmer) - Maujean**^35^** (2015, A*: 0.19), Kamioka**^28^** (2014, A*: 0.73); 2 studies | **PSD** (psychiatric disorders or depression)   - N=119 (76 IG, 43 IG) - Age and sex nr - Norway (nr X1) | 2 X wk for 12 wks for sessions 1.5-3hr long | - RCT - waitlist CG^397-399^ | | **Other**   - ↑ self-efficacy (p=0.045, η2=0.24),^397^ including self-efficacy at 6m (p=0.001, η2=0.25)^398^ - ↑ coping abilities at 6m in IG (p=0.03, η2=0.11)^398^ - +/o effects on anxiety: ↓ (p=0.002, η2=0.21)^399^ and no ∆ (p=0.059, η2=0.22)^397^ - ↓ depression (p=0.003, η2=0.45)^397^ at 6m in the IG (p=0.002, η2=0.21) and the CG (p=0.0005, η2=0.37)^399^ | | - These were the only AAI studies to measure longer-term effects | Psychological functioning: Overall positive effects  Moderate quality (nr X1) |
| *50* | *Dolphin therapy* (Antonioli and Reveley ^400^, HQ) in which participants could play, swim with (snorkelling), touch and take care of the dolphins. The sessions included a structured component led by the trainer in which they learned about dolphin behaviour and safety, the trainer gave the dolphin commands to perform specific behaviours (e.g., jumps) and were supported by the trainer when in the water. The second part of the trial included unstructured free and spontaneous play opportunities with the dolphins.   - Segregated - Kamioka**^28^** (2014, A*: 0.73); 1 study | **PSD** (mild-moderate depression)   - N=30 (15 IG, 15 CG) - Age & sex nr - USA and Honduras | 1 hr daily for two wks | - RCT – active CG: Outdoor Nature Program with similar water-based activities | | **Other**   - ↓ depression | | nr | Psychological functioning: Overall positive effects  High quality |
|  | *Art participation or therapy* |  |  |  | |  | |  |  |
| 51a | Participation in *arts-based activities* in day programs for people with ID, which included the development of artistic skills and the opportunity to display or sell work through exhibitions with the person with ID presenting their own work (Darragh, et al. ^401^, LQ).   - Segregated and integrated - Bigby**^4^** (2018, A*: 0.46); 1 study | ID (severity nr)   - N=5 - 21-27 years - Sex nr - Australia | Arts program operated 2 days/week, participants had attended for 2-6 years++ | - Qualitative | | **Social functioning**   - Participants enjoyed the activities, including public praise of their work, - ↑ development of friendships with others at the group and expanded individual social networks   **Other**   - ↑ happiness and confidence | | - little evidence for longer-term outcomes (e.g., increased opportunities for social interactions or convivial encounters with community members without disability) | Social function: positive effects, but limited to group setting/ participants  Low quality |
| *51b* | *Open studio art programs* or therapy programs used a recovery approach where the facilitator could be equal to that of participant where they are working side by side (Allan, et al. ^402^, ?Q). Programs were generally open to anyone with mental health problems who wished to participate (Chiu, et al. ^403^, ?Q), and were run in a range of settings including community centres^402^ (Bungay and Clift ^404^; Hacking, et al. ^405^, both ?Q), the researcher artist’s own home (Stokrocki, et al. ^406^, ?Q), psychiatric rehabilitation or mental health centres (Howells and Zelnik ^407^, ?Q; Thompson ^408^, ?Q), centres for homeless people (Griffith, et al. ^409^, ?Q). One program was specifically for homeless women who had experience domestic violence (Stokrocki, et al. ^406^, ?Q). Other programs were classified as ‘arts on prescription’ courses, workshops, studios or courses for people with mental health problems Hacking, et al. ^405^ ({Spandler, 2007 #64, ?Q). The SR by Bungay^404^ that was included in the umbrella review by Roche, et al. ^48^ included 12 arts on prescription projects in the UK that were delivered via referral to non-medical community and voluntary sector resources.   - Integrated^405,407^, segregated^402,403,406,408-410^, or both integrated and segregated^404^ - Finkel**^14^** (2020, A*: 0.42), Webber**^57^** (2017, A*: 0.15), Roche**^48^** (2019, UR, A*: 0.19); 8 studies, 1 SR | **PSD** (psychiatric disorders, SMI, mental health problems, homelessness)   - N=60 (nr for 2 studies one SR) - Age and sex nr consistently except one program for women only^406^ - Canada, UK X2, USA X4 (nr X2) | 1 X 2hr sessions per week^402,403^  2 X wk for 6 wks^408^  Unlimited access to the studio for 1 year^409^  (nr or n/a X 4) | - NRCT-No CG^405^ - Qualitative^406-408,410^ - Mixed methods^402,403,409^ - Systematic Review^404^ | | **Social participation or functioning**   - Participating in an open studio created a “community of artists” and was perceived as a bridge to the broader community^407^ - ↑ social inclusion,^402,405^ ↑ sense of community belonging,^403^ ↑ social engagement,^404^ ↑ mutual support^410^ and social connections, meaning and self-esteem^406^ - 81% wanted to have ongoing participation in a similar group after completing the current art program^403^ - People who could sell their artwork in the gallery had a more meaningful increase in life achievements than the participants who only took part in the open studio,^409^ and participation contributed positively to forming new identities and roles,^407^ and “formation of a new sense of self and the discovery of empowering new self-narratives”^408^   **Other**   - positive correlation between art group participation and increased life achievements (e.g., finding work or housing, recovery)^409^ - ↓ distress,^402^ ↓ negative mood,^403^ ↓ negative psychiatric symptoms^408^ - ↑ QoL (Allan) and ↑ health and wellbeing^404^ | | - It is not clear if beneficial effects on social inclusion are unique to arts-based activities, or if they are due to the participation in group activities^404^ | Social functioning and identity: Positive effects  Psychological functioning: Positive effects  Quality nr |
|  | *Dance therapy/programs* |  |  |  | |  | |  |  |
| 52 | Dance movement therapy programs including the *We Dance and Find each other* intervention (Hildebrandt, et al. ^411^, HQ), which focus on mirroring/imitation, synchronisation and interaction (Koch, et al. ^412^, HQ; Koehne, et al. ^413^, HQ; Mastrominico, et al. ^414^, MQ). The content of two interventions was not clearly described (Mateos-Moreno and Atencia-Doña ^415^, ?Q; ^416^, MQ).   - Segregated - Pallathra**^41^** (2019, A*: 0.12), Takahashi**^53^** (2019, A*: 0.42), Lorenc**^30,31^** (2018, A*: 0.42), Hallett**^23^** (2019, A*: 0.12), Soundy**^52^** (2014, A*: 0.46); 6 studies | ASD without ID,^412,413^ ASD with ID^411^,^414^ “Severe autism”^415^ or PSD (schizophrenia)^416^   - N=82 (ASD, no ID), N=151 (ASD + ID or “severe”), N=3 (PSD) - M=22-33, R: 14-53 years - 71.9% male (nr X3) - Germany X2 (nr X4) | Weekly for 7^412^ or 10 weeks/sessions^411,413,414^ for 1-1.5hr per session (nr X 2) | - Qualitative^416^ - NRCT – “no intervention” CG^412^, or CG not described^415^ - NRCT – active CG: control movement intervention focused on dexterity, balance and endurance^413^ - RCT – CG not described^411,414^ | | **Social skills**   - People reported feeling valued by others for having specific roles and responsibility within the sessions, and that emotional support provided them with empathy^416^ - ↑ interaction, imitation, emotion, instinct and regulation in communication^415^ - No ∆ social skills (g=0.35, 95%CI: -0.36, 1.06)^412^ - No ∆ self-other awareness (g=0.44, 95%CI: -0.27, 1.16)^412^ - No ∆ empathy^414^: Emotional empathy scale (g=0.29, 95%CI: -0.42, 0.99), multifaceted empathy test (g=0.03, 95%CI: -0.52, 0.58), perspective taking (g=0.05, 95%CI: -0.50, 0.60) and empathic concern (g=-0.05, 95%CI: -0.60, 0.50)^413^   Note: Koch^412^ reported to show positive effects on social skills, self-other awareness and wellbeing, but the 95%CIs are all ns.  **Other**   - No ∆ attention, perception, association, intention, muscle tone, motility, contact, or communication regulation^415^ - No ∆ psychological wellbeing (Heidelberger State Inventory; g=0.34, 95%CI: -0.37, 1.05)^412^ - ↓ negative affect and improved emotion expression with a partner without autism^411^ | | Nr | Social skills: Limited/no effects  Quality: 2 mod, 3 high (nr X1) |
|  | *Drama therapy/programs* |  |  |  | |  | |  |  |
| 53 | There were 13 *drama therapy group programs* for people with ID (Stickley, et al. ^417^, HQ), ID and PSD (Hackett and Bourne ^418^, Foloştină, et al. ^419^, Gardner-Hynd ^420^; all ?Q) or PSD (Grainger ^421^, Jaaniste ^422^, Jacques ^423^, Lahad ^424^, McAlister ^425^, Orkibi, et al. ^426^, Ramsden, et al. ^427^, Holloway ^428^, Dent-Brown and Wang ^429^; all ?Q).  The focus of these programs included developing performances presented to mainstream students^417^ or the public ^423^, using drama for storytelling, ^418-420,422,424,429^, drama therapy to enhance self-awareness and insights into self and others,^421^ or to create positive relationships with others.^428^ Programs with offenders with PSD focused on recreating positive interactions^425^ or working on increasing feeling of remorse.^427^ One program included people with and without mental illness to facilitate social interactions and reduce stigma.^426^   - Mostly segregated, but with some opportunity to perform for people without disability - Bigby**^4^** (2018, A*: 0.46), Bourne**^6^** (2018, A*: 0.42); 13 studies | **ID** (severity nr), Learning Disorders + mental health conditions, or **PSD** (thought disorders, schizophrenia, PTSD, suicide survivor, bipolar disorder or various mental health disorders)   - N=31 ID (+7 staff & 20 family), N=171 PSD (+43 staff) - Age nr - Sex nr consistently, but 4 programs with 100% men^420,422,425,427^ - Australia, Israel X2, Romania, UK X8 (nrX1) | 4d,^427^ 5 wks,^424^ 10-11 sessions^421,422^, 20 wks,^426^ 4m^419^, 5m^423^, 6m^428^  Session length was nr in all SRs | - Descriptive case study^424,425^ - Qualitative^417,419,420,422{Ramsden, 2010 #468,423,428^ - Mixed methods^418,421,426,429^ | | **Social participation**   - Being creative together led to ↑ mutual support, social inclusion and acceptance,^423^ and ↓ isolation ^421^ - ↑ relationships and friendships with other participants^417,418,420-422,424,426-428^ and members of the community/people without disability^417,426^ - Participants established a peer support group after completing the program^418^ - ↑ leisure activity participation^418^   **Social skills**   - ↑ communication and social skills^419,422,426^ and ↑ engagement with others^420,421^ - ↑ self-awareness and awareness of others,^421,425,426,429^ ↑ impulse control and ↓ challenging behaviours^419^ - ↑ feelings of remorse and empathy for victims of their offending^427^ and trust to share with others^421^   **Other**   - ↑ creativity,^423^ ↑ empowerment,^418,429^ ↑ confidence,^417^ ↑ feelings of self-worth,^418^ ↑ self-esteem^417,422,426^ and ↑ resilience^424^ - ↑ QoL,^418^ ↑ mood^419^ and mental health^428^ - ↑ recovery from substance use (50% recovery rate)^422^ - ↓ perceived discrimination and internalised stigma^426^ - Participants were generally positive about their experiences^422,423,425^ which they described as being ‘powerful’^427^ | | - Trusting others can be difficult. Developing and sharing personal stories in a group setting can lead to discomfort and feeling “exposed”.^429^ Important to build trust within the group^425^ - No studies included a control group, so difficult to determine whether it is a group effect or due to the drama therapy component. | Social functioning and skills: Positive effects  1 high quality (nr X13) |
|  | *Music therapy/interventions – mostly in PSD* |  |  |  | |  | |  |  |
| 54a | The music program *Soundscape* encouraged creative sound exploration, composition and improvisation for people with ASD (Hillier, et al. ^430^, ?Q). The intervention aimed to improve social functioning and general wellbeing.   - Segregated - Lorenc**^30,31^** (2018, A*: 0.42); 1 study | **ASD** without ID   - N=22 - Age & sex nr - USA | Nr | - NRCT – No CG | | **Social functioning**   - ↑ peer relations   **Other**   - ↑ self-esteem and ↓ anxiety | | Nr | Social functioning: Positive effects  Quality nr |
| 54b | Music therapy focused on *receptive processes* (Liu, et al. ^431^, LQ) such as the Sound Training Attention and Memory (STAM) program (Ceccato, et al. ^432^, MQ), and included listening to music with a focus on music appreciation and discussion (Qu, et al. ^433^, LQ; Cha, et al. ^434^, MQ), the emotional aspects and dancing (Wen, et al. ^435^, LQ; He, et al. ^436^, LQ), with some programs including additional content specifically on coping and social support (Silverman ^437^, LQ), or positive hypnosis and imagery (Li, et al. ^438^, LQ).  Programs with a primary focus on *active music process*es, including music improvisation (Fu and Zhang ^439^, MQ; Talwar, et al. ^440^, MQ), playing instruments, singing and dancing (Mohammadi, et al. ^441^, LQ), and used music to deliver psychotherapeutic treatment in individual (e.g., focusing on strengths and potential; Gold, et al. ^442^, HQ), or group settings (Ulrich, et al. ^443^, HQ).  The remaining programs included *both active and receptive music therapy element*s (Lu, et al. ^444^, LQ; Chang, et al. ^445^, MQ; Mao, et al. ^446^, LQ; Tang, et al. ^447^, LQ; Wang ^448^, LQ; Yang, et al. ^449^, LQ).  Two programs were provided individually^440,442^, with all others provided in group settings.   - Clinical/segregated, with all studies with inpatients, two of which also included outpatients^432,442^ - Geretsegger**^20^** (2017, A*: 0.94), Ma**^33^** (2020, A*: 0.50); 19 studies | **PSD** (schizophrenia, psychotic disorders, and axis 1 mental health conditions)   - N=1311 (597 IGs, 618 CGs, nr X1) - M=25-43, 15-60 years - 66.5% male (nr for N=232) - Country nr for 18/19 studies | 1 X 45-50 min sessions/wk for 12,^440^ 16^432,450^ or 24 wks^437^  2 X 45-60min sessions/wk for 5,^444^ 12,^442^ 16 wks^431^  3 X 45min sessions/ wk for 4 (Mohammad, Fu) or 12 wks^448^  2 X 45min - 1.5hr daily sessions for 16 wks,^433^ or for 5 days/wk for 6m^446^  5 X 40-1hr sessions/wk for a total of 19,^447^ 30^435,436,438^ or 40 sessions^445^  6 sessions per/wk for 2hr,^449^ 4 wks^434^ or m=7.5 sessions of 60-105 mins^443^ | - RCT - TAU CG^431-436,438-440,442-450^ - RCT - active CG: scripted education on support/coping or playing games,^437^ or passive music therapy^441^ | | **Social functioning**   - ↑ social functioning in short term (SMD=-1.25, 95%CI: -1.94,-0.57),^439^ medium term (SMD=-1.20, 95%CI: -1.65,-0.75;^446^ SMD=-0.78, 95%CI: -1.27,-0.28)^449^ and long term (SMD=-1.80, 95%CI: -2.29,-1.3)^446^ - No ∆ perceived overall social support between coping/social support intervention with live or recorded music and CGs without music or support/coping education; ↑ perceived support from friends for people in live music + coping/support group versus group with no education component (SMD=5.34, 95% CI: 0.47, 10.40).^437^ - QoL perceived social support (SMD=0.73, 95%CI: 0.26,1.21)^445^   Other   - Anxiety: ↓ in short-term (SMD=-0.61, 95%CI: -1.13,-0.09)^438^ - Depression: No ∆,^435^ ↓ in short-term (SMD=-0.68, 95%CI: -1.21,-0.16;^438^ SMD=-0.73, 95%CI: -1.2,-0.26)^444^ - Cognitive function: No ∆ attention, vigilance, memory or abstract thinking in the medium term,^432,450^ ↑ memory in the short term (SMD=0.58, 95%CI: 0.06, 1.06)^434^ and ↑ abstract thinking in long-term (SMD=1.18, 95%CI: 0.33, 2.03)^431^ but not in the short-term^431,434^ - **Psychiatric symptoms**: No ∆,^436,440^ ↓ in short term (SMD=-0.69, 95%CI: -1.16,-0.23)^444^ and ↓ in medium term (SMD=-1.00, 95%CI: -1.55,-0.45;^433^ SMD=-1.55, 95%CI: -2.02,-1.07;^446^ SMD=-1.25, 95%CI: -1.77,-0.73)^449^ and ↓ in the long term (SMD=-3.41, 95%CI: -4.07,-2.76),^446^ including negative symptoms with no ∆,^442,443^ or ↓ in short-term (SMD=-0.63, 95%CI: -1.06,-0.2;^441^ SMD=-1.07, 95%CI: -1.55,-0.58)^447^ or ↓ medium term (SMD=-0.97, 95%CI: -1.47,-0.47)^449^, and positive symptoms with ↓ in short-term (SMD=-0.18, 95%CI: -0.6,0.24)^441^ - No ∆ in **QoL^443^** or **global state^449^** or general functioning,^440,442^ but ↑ QoL in short term (SMD=1.82, 95%CI: 1.27,2.38)^445^ - ↑ in general behaviour in short term (SMD=0.68, 95%CI: 0.04,1.32)^439^ and medium term (SMD=0.69, 95%CI: 0.18, 1.20)^448^ | | Nr | Social functioning: overall positive effects, but inconsistent effects on perceived social support  Psychiatric functioning: Mixed effects, but generally positive effects for negative symptoms  Quality: 12 low, 5 mod, 2 high |
|  | *Ecotherapy/horticulture groups* |  |  |  | |  | |  |  |
|  | *Horticulture group* in an urban farm (Gonzalez, et al. ^451^, ?Q) *and ecotherapy group* in a public green space working on environmental conservation (O'Brien, et al. ^452^, ?Q). *Social recreation or leisure programs* with information sessions, personal development workshops, self-help groups, community walks and forums for people with PSD (Petryshen, et al. ^453^, ?Q).   - Segregated - Webber**^57^** (2017, A*: 0.15), Masi**^34^** (2011, A*: 0.34); 3 studies | **PSD** (depression, severe and persistent mental health conditions).   - N=36 PSD (2 studies nr) - R:18-65,^453^ nr X2 - 20-39% male PSD (2 studies nr) - Canada (2 studies nr) | 2-3 hrs per session X 1-3 sessions/week (PSD) | - Qualitative^452^ - NRCT-No CG^451,453^ | | **Social participation**   - ↓ loneliness (effect=-0.59, 95%CI: -0.88, -0.30)^453^ - ↑ social activity for 38 and 31% of participants at group end and 3-month follow-up, respectively^451^ - ↑ relationships within group, and wider community^452^ | |  | Loneliness: positive effects  Social activity & relationships: Positive effects for PSD  Moderate quality (nr X2) |
|  | *Clinical interventions* |  |  |  | |  | |  |  |
|  | Clinical support from a social worker using telehealth (Morrow-Howell, et al. ^454^, ?Q) and automated (Pijnenborg, et al. ^455^, ?Q) or tailored (Granholm, et al. ^456^, ?Q) SMS messaging for people with PSD to manage medication adherence, socialization, or hallucinations. A stress management program on a palm pilot device was evaluated for people with ASD (Gracey ^457^, ?Q)   - Segregated - Naslund**^38^** (2015, A*: 0.42), Masi**^34^** (2011, A*: 0.34), Lorenc**^30,31^** (2018, A*: 0.42); 4 studies | **PSD** (Schizophrenia/ Schizoaffective disorder; suicidal, depressed or socially isolated) or **ASD** (with no ID)   - N=178 PSD, N=9 ASD - M=31^457^, R:61-92 years^454^ (nrX2) - 15-56% male (nr X2) - Netherlands X1, UK X1, USA X2 | 12wks, 18wks, 8m long  Frequency/ time nr | - NRCT-No CG,^456,457^ RCT waitlist CG) ^454^ - NRCT - CG “no intervention” ^455^ | | **Social participation**   - ↑ social interactions^456^ - No ∆ in loneliness (Effect=-0.09, 95%CI: -0.59, 0.41)^454^ - ↑ leisure activity participation when prompted via SMS^455^   **Other**   - ↑ goal achievement during SMS prompting, but decay after SMS ceased^455^ - ↑ appointment adherence^455^ - +/o for medication adherence: ↑ adherence and ↓ hallucination severity with tailored SMSs^456^ but no ∆ adherence with automated SMS prompts^455^ | | - Increased anxiety from palm pilot intervention^457^ | Social activity/ interactions: positive effects  Quality nr |
|  | *Psychoeducation programs* for people with PSD with a computer-based course (Pitkänen, et al. ^458^, MQ) or telehealth and online programs (Rotondi, et al. ^459^, LQ), or group-based programs with an asset-based focus on recovery (*TREE model*; Boevink, et al. ^460^, HQ) and comprehensive programs focused on problem solving, coping skills, illness management, social participation (Aho‐Mustonen, et al. ^461^, HQ; Vreeland, et al. ^462^, MQ; Walker, et al. ^463^, MQ; Haslam, et al. ^464^, ?Q; Hesselmark, et al. ^465^, HQ; Atkinson, et al. ^466^, LQ).   - Segregated - Ma**^33^** (2020, A*: 0.50), Puolakka**^45^** (2019, A*: 0.42), Webber**^57^** (2017, A*: 0.15), Pallathra**^41^** (2019, A*: 0.12), Lorenc**^30,31^** (2018, A*: 0.42); 9 studies | **PSD** (schizophrenia or psychosis, mental illness, depression or anxiety) or ASD+PSD   - N=912 (233 IGs, 311 CGs), nr 4 studies - m=32-38, > 14 years or “young adults” (nr 3 studies) - 85% male (nr 4 studies) - Sweden, Finland X2, Netherlands, UK, Scotland, USA X2 (nr 1 study) | Length varied: 5 sessions (length nr X 2 studies), 8wks, 11wks, 20wks, 24wks, 36wks, 1-2yrs (nr 3 studies)  **Frequency**: 1-2 p/wk or fortnight (nr 3 studies)  **Time,** varied: 30-40m, 45-60m, 60-75m, 2hr p/session or 1.5-3h p/week | - NRCT - CG not described^464^ - RCT with active CG: traditional education,^458^ unstructured recreation activity^465^ - RCT vs TAU or waitlist CG^459-463,466^ | | **Social participation**   - +/o for loneliness: ↓ for social connectedness focused-program at program end,^464^ but no ∆ for fortnightly TREE recovery-oriented program at 12m and 24m between groups (B=− 0.053, 95% CI: − 0.31, 0.30)^460^ - ↑ social functioning at program end,^464^ and ↑ social contacts in IG at program end and follow up^466^ - No ∆ in perceived social support at 3 or 6m^459^   **Other**   - +/o for QoL: ↑,^460,466^ or no ∆ (g=0.20 (-0.31, 0.70),^465^ at post-treatment^461,463^ and 3m, 6m or 12m^458^ - No ∆ in psychological wellbeing at 8wks or 24wks,^462^ or depression (Hedges g=0.06, 95%CI: -0.46, 0.58)^465^ | | Nr | Loneliness: Mixed short-term effects  Social functioning: positive effects  Quality: 2 low, 3 mod, 3 high (nrX1) |
|  | *Family therapy and psychoeducation* group and family sessions (Bradley, et al. ^467^, HQ; Ngoc, et al. ^468^, MQ), with individual counselling in one study (Girón, et al. ^469^, HQ).   - Clinical/segregated - Puolakka**^45^** (2019, A*: 0.42); 3 studies | **PSD** (schizophrenia or schizophreniform disorder)   - N=159 (80 IG, 79 CG) - M=25-34 years - 51.6% male - Vietnam, Spain, Australia | **Length & time**: nr  **Frequency**: 3 sessions^468^ to 28-34 sessions^467^ ^469^ | - RCT – TAU CG,^468^ standard case management CG,^467^ individual counselling CG^469^ | | **Quality of life**   - +/o effect on QoL, with no ∆ post-treatment,^467^ but ↑ QoL adjusting for baseline differences of Hedges g=0.29,^468^ with g=0.40 at 9m, and g=0.87 at 24m^469^ | | nr | QoL: positive longer term effects  Quality: 1 mod, 2 high |
|  | Group *Mindfulness Therapy* (Kiep, et al. ^470^, LQ), including focus on anxiety (Spek, et al. ^471^, MQ) for people with ASD.   - Segregated - Pallathra**^41^** (2019, A*: 0.12); 2 studies | **ASD**   - N=91 (70 IG, 21 CG) - Demographics & countries nr | 2.5 hrs/wk for 9 wks | - NRCT-No CG^470^ - RCT - CG not described^471^ | | **Social & psychological functioning**   - ↓ anxiety & depression symptoms, ↓ rumination, and ↑ positive affect^471^ - ↓ agoraphobia and somatisation^470^ | | nr | Functioning: Positive effects  Quality: 1 low, 1 mod |
|  | *Cognitive Behavioural therapy (CBT)* individual therapy including behaviour activation for depression (Mendelson, et al. ^472^, LQ) or social interaction and communication visualisation to reduce social anxiety for ASD (Ekman and Hiltunen ^473^, LQ). Group-based CBT programs targeted social and relationship skills for birth-related trauma (Sorenson ^474^, ?Q), social anxiety for ASD (Sizoo and Kuiper ^475^, Spain, et al. ^476^; both LQ) or targeting depression and stress (McGillivray and Evert ^477^, HQ). Veltro (2011^478^, MQ) evaluated *cognitive emotional rehabilitation* for schizophrenia.   - Segregated - Ma**^33^** (2020, A*: 0.50), Pallathra**^41^** (2019, A*: 0.12), Masi**^34^** (2011, A*: 0.34), Grant**^22^** (2017, A*: 0.46); 7 studies | **PSD** (trauma, depression, schizophrenia) and ASD   - N=11 PSD, N=123 ASD (85 IG, 48 CG) - M=33-39, R:14-45 years (nr 4 studies) - Sex nr (100% female)^472,474^ - USAX2, nr X5 | 6wks, 9wks, 11wks, 13wks, 15 or 24 sessions, 5m long  **Frequency**: weekly (4 studies), monthly (1 study), nr X2  45- 60 mins/session (1 study), 1.5-2 hrs/session (5 studies), 4 hrs/session (1 study) | - NRCT-No CG^473,476^ - NRCT – CG not described ^474^ - RCT – CG not described^477^ - RCT with active CG: home visit & education,^472^ MBSR,^475^ problem solving training^478^ | | **Social participation**   - No ∆ in perceived social support at 3m and 6m (β=6.67, SE=0.03, p < 0.10)^472^ - ↓ loneliness (Effect=-4.81, 95%CI: -7.09, -2.53)^474^ - ↑ Personal and Social Performance Scale^478^   **Other**   - ↓ anxiety and depression symptoms in both IG and MBSR CG^475^   Note: Results not reported for three studies^473,476,477^ in SRs. Type of effect size for Sorenson^474^ not reported. | | Nr | Loneliness and social performance: Positive effect  Social support: no effect  Quality: 4 low, 1 mod, 1 high |
|  | *Cognitive reframing* for depression to enable people to understanding through reflective responses, followed by use of 3–5 positive reframing directives for reframing subjects (Conoley and Garber ^107^, ?Q). The *Family of Heroes* intervention provided psychoeducation and encouraged cognitive changes in people with PTSD after deployment (Interian, et al. ^479^, LQ). *Cognitive remediation* programs for schizophrenia taught participants to develop strategies to analyse and interact in social situations by focusing on social cognitive structures (Van der Gaag, et al. ^480^, MQ; Peyroux and Franck ^481^, ?Q).   - Clinical/Segregated - Ma**^33^** (2020, A*: 0.50), Fernandez-Sotos**^13^** (2020, A*: 0.19), Grant**^22^** (2017, A*: 0.46); 4 studies   *NOTE: a separate review is required for a comprehensive overview of cognitive remediation* | **PSD** (depression, PTSD, schizophrenia)   - N=204 - Age & sex nr consistently - USA X2 (nr X2) | 1-2 sessions for total of 1hr^107,479^  14-22 sessions up to 2 hrs/week ^480,481^ | - NRCT-No CG^481^ - RCT with waitlist or TAU CG^107,480^ - RCT - CG not described^479^ | | **Social functioning:**   - No ∆ in loneliness after 2 X 30 min cognitive reframing sessions^107^ - ↓ perceived social support at 2m^479^   **Capacity**   - ↑ social cognitive processes, including emotion recognition, attribution style, social perception/knowledge and empathy,^481^ and theory of mind^480,481^   **Other**   - ↓ schizophrenia symptoms and ↑ daily functioning^481^ | | - Psychoeducation and simulated conversations led to perceptions of less social support^479^ | Social capacity: positive effects  Social support: negative impact  Loneliness: No effect from short-term intervention  Quality: 2 low, 1 mod (nr X1) |
|  | *Cognitive Enhancement Therapy* (CET) for people with schizophrenia or ASD involves individual and group components to treat impairments in social and non-social information processing and problems solving (Eack, et al. ^482^, MQ; Eack, et al. ^483^, LQ; Eack, et al. ^484^, MQ; Eack, et al. ^485^, MQ; Choi and Kwon ^486^, LQ). *Meta-cognitive training* was used to change cognitive structure in people with schizophrenia (Roncone, et al. ^487^, MQ)   - Clinical/segregated - Grant**^22^** (2017, A*: 0.46), Pallathra**^41^** (2019, A*: 0.12), Lorenc**^30,31^** (2018, A*: 0.42); 6 studies | **PSD** (schizophrenia) or **ASD**   - N=143 PSD (80 IG, 63 CG), N=68 ASD (35 IG, 19 CG) - M=25-39 years - 67.6% male - USA X2, nr X4 | **Length:** 18m-2y  **Sessions:** total of 36-45 sessions; weekly for 18m (nr X1)  **Time**: 60-75min (1 study), 1.5 hr/session (3 studies), total 22 hrs^487^ | - NRCT-No CG^483^ - RCT - TAU CG,^484^ standard psychiatric rehabilitation^486^ or CG not described^485^ - RCT with active CG: medication & psychotherapy if needed,^487^ Enriched Supportive Therapy^482^ | | **Social skills**   - ↑ global social composite score^482^ - ↑ social perception knowledge^486^ - +/o effect on theory of mind: ↑,^487^ No ∆^484^ - ↑ cognitive style and social cognition^483^ - +/o effect on affect recognition: ↑ post-treatment,^484^ or no ∆^486^   **Other**   - ↓ disability^487^ and ↑ function^484^ - ↑ likelihood of employment^485^ | | Nr | General social perception/ function: positive effects  Theory of mind & affect recognition: Mixed effects  Quality: 2 low, 4 mod |
|  | *Behaviour Activation* (BA) (Gawrysiak, et al. ^488^, MQ) interventions included an online program with individual telephone support and group chatroom moderated by peers training in low intensity BA (*NetmumsHWD*; O'Mahen, et al. ^489^, MQ). BA interventions involved assessing values and goals, creating an activity hierarchy, and selecting value-based behaviours and goals.   - Clinical/segregated - Ma**^33^** (2020, A*: 0.50); 2 studies | **PSD** (depression)   - N=113 - R:Aged >18 years - Sex nr except 100% female for O'Mahen, et al. ^489^ - USA & UK | 1 X 1.5hr session^488^  12-session + 20-30min/wk telephone support^489^ | - RCT with no treatment CG^488^ or TAU CG^489^ | | **Social functioning**   - No ∆ perceived social support after a single BA session^488^ or 12-week online program at end of program or 6m (effect=0.50, 95% CI − 0.02, 1.02)^489^   **Other**   - ↓ depression symptoms, no ∆ anxiety^488^ | | Nr | Perceived social support: no effect  Psychological functioning: Mixed  2 mod quality |
|  | *Narrative Exposure Therapy* (NET) for PTSD in which participants create a chronological report of biography with a focus on traumatic experiences (Zang, et al. ^490,491^; both LQ)   - Clinical/segregated - Ma**^33^** (2020, A*: 0.50); 2 studies | **PSD** (PTSD)   - N=52 (IG/CG Ns nr) - R:28-80 years - Sex nr - China | **Frequency**: ≥4 sessions twice wk X2 wks  **Time**: 1-1.5 hr/session | - RCT - waitlist CG, ^490,491^ also had an earthquake narrative + autobiographical narrative (NET-R)^491^ | | **Social functioning**   - No ∆ in perceived social support vs CG at program end, 2wks and 2m (p=0.05, d=0.33)^490^ - ↑ social support for both traditional NET and NET-R, no difference between NET and NET-R groups, or with waitlist control at 2wks and 3m^491^ | | nr | Social support: No effect  2 low quality |
|  | *Morita therapy* is a Japanese psychotherapy with a therapeutic principle of ‘let it be as it should be’, with four treatment phases: the bed phase, the light work phase, the heavy work phase and the social rehabilitation phase. Morita therapy aims to let patients reach a state of mind to carry on with work or study even while they exhibit some mental problems.^492-502^   - Clinical/segregated - Feng**^66^** (2020, A*: 0.84); 11 studies | **PSD** (schizophrenia)   - N=992, (IG/CG nr consistently) - M=37-48, R:18-60 years - 61.1% male - China | **Length**: 19-35 days, 6wks (4 studies), 7wks, 8wks, 10wks (3 studies), 6m  **Frequency & time**: nr | - RCT – All CGs TAU including pharmacotherapy | | **Social disability**   - +/o effects for Social Disability Screening Schedule: Improved (SMD=-0.67, 95%CI:-1.19,-0.15;^502^ SMD=-5.00, 95%CI:-5.87,-4.13)^494^ or no ∆ (SMD=-0.27, 95%CI:-0.73, 0.18)^497^ - ↑ on Inpatient Psychiatric Rehabilitation Outcome Scale (SMD=-1.33, 95%CI: -1.67, -0.98;^498^ SMD=-1.32, 95%CI: -1.69, -0.98;^492^ SMD=-1.33, 95%CI: -1.73, -0.94;^493^ SMD=-4.14, 95%CI: -4.93, -3.35)^500^   **Other: psychological functioning**   - ↓ psychiatric symptoms in all studies | | Nr | Social disability: positive effects  Mental health: positive effects  Quality: 9 low, 2 mod |
|  | *Interpersonal and social rhythm therapy* (ISRT) is delivered by mental health nurses and combines techniques of interpersonal psychotherapy with a social rhythm matrix to promote lifestyle regularity (Frank, et al. ^503^; Frank, et al. ^504,505^; ?Q). Treatment focuses on mood symptoms, social relationship quality/roles, and daily routines.   - Clinical/segregated - Crowe**^65^** (2010, A*: 0.12); 2 studies | **PSD** (affective disorders)   - N=213 (IG/CG Ns nr) - Age & sex nr - USA | **Frequency & Length**: Weekly until stable (acute) + monthly for 2yrs (maintenance)  **Time**: nr | - RCT – active CG: CSSRT (abbreviation not defined),^503^ or providing ISRT vs intensive clinical management (ICM) in acute versus maintenance stage with cross over of ISRT/ICM between acute/maintenance phases^504,505^ | | **Social functioning**   - IG had ↑ daily routines stability, but CG had no ∆ in daily routine stability^503^   **Other**   - ISRT in acute phase took longer for new affective episode relapse^504^ - ↑social rhythm regularity in acute phase associated with ↓ likelihood of episode recurrence in maintenance phase^504^ - Quicker ↑ occupational functioning with ISRT in acute phase vs ICM^505^ | | nr | Daily routine & relapse: Positive effects  Quality nr |
|  | *Integrated Psychological Therapy for Schizophrenia* (IPTS; García, et al. ^506^, LQ) and *Interpersonal Community Psychiatric Treatment* (ICPT; Koekkoek, et al. ^507^, ?Q) are clinical treatment models that focus on recovery and community participation.   - Clinical/segregated - Grant**^22^** (2017, A*: 0.46), Webber**^57^** (2017, A*: 0.15); 2 studies | **PSD** (schizophrenia)   - N=56^+^ - M=37-43 years - 44.6% male - Netherlands, Spain++ | 20-45 mins fortnightly,^507^ 2 X wk for 30 minutes for first 5 sessions, then 1 hr for remaining sessions  (total 21 sessions)^506^ | - NRCT – CG not described^507^ - RCT - CG no intervention^506^ | | **Social functioning**   - ↑ social perception knowledge^506^ - ↑ social networks and social activity^507^ - Goal setting was universally helpful for recovery motivation, but not when staff preferences were emphasised too strongly^507^ | | - Structured skills training is not acceptable to everyone^507^ | Social functioning:  Positive effects  Low quality (nr X1) |
|  | *Community-focused interventions: changing attitudes in the community (21 studies)* |  |  |  | |  | |  |  |
|  | Interventions that aim to *increase knowledge/attitudes about ID without direct contact* with people with ID, including lectures on ID to student teachers (Campbell, et al. ^508^, ?Q), vignettes and education on ED for students (MacDonald and MacIntyre ^509^, ?Q), training teachers (Rae, et al. ^510^, ?Q), residential staff (Wong and Wong ^511^, ?Q) and police officers (Bailey, et al. ^512^, ?Q) in ID diagnostic criteria or key characteristics.   - Segregated - Seewooruttun**^67^** (2014, A*: 0.15); 5 studies | Community members knowledge/attitudes about ID   - N=584 - Age and sex nr consistently - Australia, Canada, Hong Kong, UK X2 | 1 half-day session^510^ or 6 sessions^511^  13 wk course + field work^508^  nr ^509,512^ | - NRCT-No CG^508-510^ - NRCT – CG not exposed to interventions^511,512^ | | **Knowledge**   - ↑ knowledge about ID^510,511^ and Down Syndrome^508^ - ↓ endorsement of stereotypes^508^ - +/o effect on views/attitudes: ↑ positive views about Down Syndrome^508^ and ↑ attitudes by police officers^512^ and towards facilitating self-determination for people with ID for residential staff,^511^ but no ∆ attitudes in students exposed to vignettes^509^ | | nr | Knowledge and attitudes to ID: positive effects in all more substantial interventions  Quality nr |
|  | Interventions that aim to *increase knowledge/ attitudes about ID with direct or indirect (e.g., through videos or images) contact with people with ID*. Interventions with healthcare providers or trainees, including nurses attending workshops co-facilitated by a person with ID (Melville, et al. ^513^, Tracy and Iacono ^514^, both ?Q), experiential learning for medical students through interactions with families with a child with ID (Sharma, et al. ^515^, ?Q), attending a workshop on ID (Hall and Hollins ^516^, ?Q), and psychology undergraduates watching documentary and dramatic films about a man’s life with down syndrome (Hall and Minnes ^517^, ?Q), or a 10 week course that included contact with people with ID (Kobe and Mulick ^518^, ?Q).  Interventions delivered to other tertiary students examined the effect of a 2.5 day “direct contact experience” with people with ID (Nosse and Gavin ^519^, ?Q), intentions to tutor a child with an ID (Rimmerman, et al. ^520^, ?Q), and the effect of a documentary film about people with ID as part of a broader training package (Iacono, et al. ^521^, ?Q).  Several studies based at the *Special Olympics* included ophthalmologists (Adler, et al. ^522^, ?Q), healthcare students (Freudenthal, et al. ^523^, ?Q), college students (Li and Wang ^524^, ?Q) and other volunteers (Roper ^525,526^, ?Q).  Interventions with members of the general public examined the effect of viewing pictures of a person with ID (Varughese and Luty ^527,528^, ?Q), or viewing films on ID (Walker and Scior ^529^, ?Q)   - Seewooruttun**^67^** (2014, A*: 0.15); 16 studies | Community members knowledge/attitudes about ID   - N=3,154 - Age and sex nr consistently - Australia X2, Canada, China, Israel, UK X6, USA X5 | 1 session for 10 mins,^529^ 2 hrs,^515,516^ 3 hrs,^513,514^ or nr^517,527,528^  2-4 days,^522,523,525,526^ 2.5 days^519^ and 1 week during working hours^524^  10 wks for total of 20 hrs^518^  4m, frequency nr^520^  nr^521^ | - Qualitative^515^ - Mixed methods with no CG^514,521,523^ - NRCT – No CG^516,518,525,526^ - NRCT with “no intervention”,^524^ “control” condition,^513^ a control film,^517^ students from business degrees,^519^ or classified by prior contact and intent to tutor a child^520^ - NRCT with active control: written vignette describing a man with Down Syndrome,^527^ lecture with no practical training^522^ - NRCT – CG not described clearly^528,529^ | | **Knowledge and attitudes**   - +/o effect on attitudes: ↑ positive or inclusive attitudes,^514,516,519,524,529^ including 52% attitude change in IG vs 30% change in CG,^522^ but no ∆ after a 10 week course including contact with people with ID^518^ or no ∆ in attitude ratings (but improved attitudes in qual themes) after viewing a documentary.^521^ Change in attitudes greater when viewing smartly dressed man with ID.^527,528^ - ↑ knowledge,^513,518,522^ ↑ self-efficacy or confidence to work with people with ID,^513^ affect towards people with ID,^517^ and expectations for people with severe ID^523^ - Positive impact on clinical practice (e.g., longer appointments by 67% and increased use of visual aids by 56%)^513^ - People noted that education was insightful recognised obstacles (e.g., hardship, financial strain) and understanding of how to adjust for people with ID, but wanted more information from health teams^515^ - Frequent sustained contact led to ↓ positive perceptions^525,526^ | | - Inclusive beliefs and attitudes were influenced more by prior exposure than the intervention,^517^ consistent with study that found people with prior experience had ↑ willingness to tutor a child with ID.^520^ - Some studies may have been self-selecting as beliefs were already essentially at ceiling before the intervention ^519,523^ - While longer contact may be necessary for sustained shifts in attitudes,^520^ medium levels of contact may lead to more positive attitudes given that extended contact was associated with worse perceptions/attitudes^525,526^ - Females tend to have more favourable attitudes^524^ | Knowledge and attitudes to ID: Overall positive effects, although some mixed effects on attitudes, with extended contact had negative impact on attitudes in 1 study  Quality nr |

*Symbols*: +/o : mixed effects (positive and negative effects on the respective outcome); ∆: change; ↑: increase or positive effect; ↓: reduction or negative effect; Ƞ2: partial eta; Note: for some SMD statistics a positive or a negative value could favour the intervention, so the upwards or downwards arrows have been used to indicate a positive/helpful effect or negative/unhelpful effect, respectively

*Abbreviations:* A*: AMSTAR (A MeaSurement Tool to Assess systematic Review); AAT: Animal Assisted Therapy; AIDS: acquired immunodeficiency syndrome; AR: Augmented Reality; ASD: Autism Spectrum Disorder; ASLP: Australian Supported Learning Program; BA: Behaviour Activation; BST : Behaviour Support Training; CBT: Cognitive Behavioural Therapy; CET: Cognitive Enhancement Therapy; CG: control group; CI: Confidence Interval; d: days; d=: Cohen's d; EMILIA: Empowerment of Mental Illness service users: lifelong Learning, Integration and Empowerment; ESCAPE: Effective Strategy-Based Curriculum for Abuse Prevention and Empowerment; FAST: Functional Adaptations and Skills Training; FEP: First Episode Psychosis; g=: Hedges g; HIV: human immunodeficiency viruses; hrs: hours; I-CAN: Independence through Community Access and Navigation; ICM: intensive clinical management; ICPT: Interpersonal Community Psychiatric Treatment; ID: intellectual disability; IG: intervention group; IPT: Integrated Psychological Treatment; IPTS: Integrated Psychological Therapy for Schizophrenia; IQ: Intelligence Quotient; ISRT: Interpersonal and social rhythm therapy; M: Mean; m: Month; MBSR : Mindfulness-based stress reduction; MH: mental health; mins: minutes; mod: moderate; N: Number; n/a: not applicable; NET: Narrative Exposure Therapy; NICE: National Institute for Health and Care Excellence; nr: not reported; NRCT: Non-Randomised Controlled Trial; NRCT-No CG: Non-Randomised Controlled Trial with no control group (i.e., pre-post); PCP: Person Centred Planning; PCS: performance cue system; PSD: Psychosocial disability; PDD : Pervasive Developmental Disorder; PEERS‐YA: Program for the Education and Enrichment of Relational Skills for Young Adults; PMID: profound & multiple ID; PTSD: Posttraumatic Stress Disorder; PWD: people with disability; QoL: Quality of Life; R: Range; RCT: Randomised Controlled Trial; SCIT: Social Cognition and Interaction Training; SCIT-A: Social Cognition and Interaction Training for Autism; sev: severe; SMD: Standardised Mean Difference; SO: Special Olympics; SR; Systematic Review; SST: Social Skills Training; STAART: Studies To Advance Autism Research & Treatment; STD: Sexually Transmitted Disease; STEPS: Socialization, Training, Education and Parenting services; SUCCESS: Supported Employment, Comprehensive Cognitive Enhancement, and Social Skills; TAR: Training in Affect Recognition; TAU: treatment as usual; TEACCH: Treatment and Education of Autistic and related Communications Handicapped Children; UK: United Kingdom; USA: United States of America; VR: Virtual Reality; wk/wks: week/weeks; yr/yrs: year/years.

*Notes*: * The pooled sample is specific to the participants with disability unless otherwise specified; ++ details checked manually from the primary studies

## **Supplementary Table 4**. Key Facilitators and Barriers to participation

|  | Facilitators | Barriers |
| --- | --- | --- |
| 1. Program attributes | Program design   - Participation in community groups was more successful if there were multiple types of support, particularly active mentoring to ensure that the person with intellectual disability could actively participate in activities and social interactions,^334^ if they had a positive leadership response to inclusion of the person with disability, the participant was accepted warmly into the community group, there were opportunities to be integrated into the group activity, and the group was flexible in dealing with differences among members.^4^ - Peer support groups for people with PSD need to be carefully facilitated, follow a structured approach, and aim for a homogeneous group composition with minimal turnover to enhance group cohesion and belonging.^303^ - Sport programs for people with ID should follow a routine structure, include breaks, and provide rewards or positive feedback, identify appropriate activities and offer alternative options if the chosen activity proves to be inappropriate.^350^ | Program features   - Special Olympics programs with disorganised rules or policies,^381^ wide age ranges of participants,^383^ were a barrier for people with intellectual disability. - People with intellectual disability disliked being given meaningless activities in community groups, and community group participation was less successful if there was a lack of support to maintain existing networks or to build new networks.^325^ - Some people with psychosocial disability prefer one-on-one physical activity programs to group formats and can find attending new environments challenging.^52^ |
|  | Person-centred, strengths-based programs or supports that support choice, goals and reviewing needs and priorities to ensure participation opportunities remain relevant over time   - Acknowledging freedom of choice,^353,354^ and using person-centred planning to identify programs and supports enhanced leisure and physical activity participation^372^ - Goal setting and self-monitoring were key supports for participation in physical activity,^353,354^ vocational and social skills group programs,^133^ and peer support groups.^303^ - Reviewing plans regularly to maintain participation as lifestyle and needs change over the lifespan^70^ - Tailoring interventions to participant needs, involving their partners,^197^ and adapting or selecting content to priority topic area for each person.^251^ | Participant choice is not respected   - Choice making can be hampered by caregivers who influence the choices of the person with intellectual disability, as captured by the title of one paper “I always do what they tell me to do”,^76,273^ and planning meetings can be overpowered by staff or family. - The wishes of people with intellectual disability are sometimes ignored, reinterpreted or misinterpreted, which negatively impacts on choice in decision making.^76^ - In befriending programs people with intellectual disability reported having little choice in the nature and frequency of their interactions with their volunteer.^295^ |
|  | Opportunities to practice and generalise skills learned in the community and in real life   - Programs were generally more successful if they provided people with frequent contact with the therapist,^92^ and included opportunities to practice and apply the skills being learned in real life community settings^88-92,104,185^with the support of a trained facilitator,^88,185^ and if the intervention blended real world and digital information in a meaningful way (e.g., for travel training).^263,264^ - Homework was considered to be helpful,^252^ and helped to generalise skills into real life.^249,250^ - Providing robust opportunities to build social networks with peer support, including participating in activities in the community together and through home visits and telephone contacts was considered to be a key driver of the strengths-based case management model evaluated by Rivera^299^. | Travel and support needs are not met   - Difficulties with transportation, staff or psychosocial support to encourage participation, and financial resources were a barrier for people with intellectual disability^350,369^ and psychosocial disability^46,52^ to participate in sport. - Low participation in physical activity was also associated with feeling dependent on others (e.g., needing reminders) for people with psychosocial disability^46,52^ - Lack of travel and insufficient money were a barrier for people with intellectual disability to participate in community groups^325^ |
|  | Access to booster support or training if needed   - To maintain effects, some people may need additional training and support.^225^ |  |
|  | Group programs allow participants to meet others with similar disabilities or experiences   - People attending group social skills^134^ and drama programs^422,424^ enjoyed being around others with a similar story and experiences, which gave them a valued sense of community and opportunity to interact with and learn from other adults with similar disabilities and experiences - Adherence to education programs can be enhanced when there is co-facilitation or peer support from someone with lived experiences of the same disability.^307^ |  |
| 1. Carers, staff, facilitator or peer mentor attributes | Carer, staff, facilitator or peer mentor attributes:   - Consistent input and engagement from carers encourages participants’ behaviour change and participation in sport programs^353,354^ - Staff were identified as one of the most important sources of emotional and instrumental support to facilitate goal attainment in PCP^72^ - A moderator who was equal to the participant, working side by side, was highlighted a facilitator of inclusion and belonging in art-based programs.^402^ - Successful peer mentors were understanding, empathetic, punctual, flexible and professional and established a comfortable relationship with their mentee in which they made time for getting to know their mentee,^322^ and the mentor and mentee were matched across multiple attributes including personality, hobbies and interests (e.g., sports), age, gender,^321^ and preference to have a mentor with or without the same disability.^317^ - Goal setting interventions rely heavily on the nature of the relationship between clinical staff and study participants^61^ | Negative/unhelpful staff/carer attitudes   - A lack of support to participate in physical activity has been reported to be a barrier to physical activity participation for 50% of people with psychosocial disability.^16^ - The people who should be encouraging healthy behaviour and active lifestyles actually enable and reinforce sedentary behaviour for people with intellectual disability.^350,390^ |
|  | Programs and facilities establish trust and safety   - Trust was integral to establishing relationship with social prescribers,^282^ and to build confidence to engage with the referred services.^274,275^ - In drama programs being around others with a similar story allowed group members to build trust so that experiences and behaviour could be safely disclosure and explored (this was a program for offenders).^425^ | Safety/vulnerability   - People with psychosocial disability report safety-related barriers to physical activity participation, including feeling unsafe, having fears of injury,^16^ social anxiety and apprehension to be around strangers and having negative expectations (e.g., feeling vulnerable, embarrassed, disliking feeling controlled by others, having to interact with others, pain).^46,52^ - People with psychosocial disability can feel vulnerable in group programs in which they share their personal experiences (e.g., in drama therapy), especially people with borderline personality disorder who reported finding to more difficult to build trust and participate in role play.^429^ |
| 1. Participant attributes | - People with intellectual disability who had friendly dispositions and relatively good social skills tended to have more positive experiences in their transition to retirement and community participation.^4^ | Participant attributes/skills   - Participation in social skills interventions was reported to be hampered for people with intellectual disability who had low levels of “social capital”, which was described as the background characteristics of poor education, youth, and low wealth.^255^ - Digital literacy and experience can hamper participation, and people with intellectual disability with no experience with computers or gaming may find it difficult to use virtual and augmented reality^260^ |
|  |  | Participants have incompatible expectations or conflicts   - In social prescribing interventions that focus broadly on improving health, reducing loneliness and enhancing social connectedness, it is important that realistic expectations are established. In one study participants who did not experience expected health improvements had reduced confidence after the program.^284^ - Conflicts with existing personal commitments or valued activities can impede physical activity program participation for people with intellectual disability^352^ and psychosocial disability.^46,52^ |
|  |  | Interpersonal clashes or social conflicts   - Participation in sport (Special Olympics) by people with intellectual disability was hampered by social conflicts with other participants and teasing from others;^381^ and being teased by peers at school.^384^ |
|  |  | Participant health or general wellbeing   - People with psychosocial disability have lower participation in physical activity if they have certain lifestyle factors (e.g., smoking, diet, sleeping patterns, fitness level and confidence), intrusive or fluctuating psychiatric symptoms, fatigue and sedative effects of medications and low self-esteem^16,46,52^ - Continued physical activity participation for people with intellectual disability can be hampered by age-related decline in health and ability for both the person with intellectual disability and their parents who may be required to support their aging child’s involvement.^375^ |
| 1. Community factors |  | Poor engagement from the community   - It can be challenging to engage family and community members who could facilitate community connections in PCP meetings^78^ - Befriending programs faced difficulties with recruiting, training and retaining the volunteer befrienders from the community.^295^ |
|  |  | Negative attitudes from the community   - Volunteers with psychosocial disability are at risk of experiencing prejudice or stigma in the community, strain from over-commitment, and social exclusion if activities are regarded as low status, especially for participants with lower confidence.^327^ - Volunteering can negatively interfere with access to other income support programs.^327^ |

## **References**

1 Anderson, K., Laxhman, N. & Priebe, S. Can mental health interventions change social networks? A systematic review. *BMC Psychiatry* **15**, 297, doi:10.1186/s12888-015-0684-6 (2015).

2 Atkinson-Jones, K. & Hewitt, O. Do group interventions help people with autism spectrum disorder to develop better relationships with others? A critical review of the literature. *British Journal of Learning Disabilities* **47**, 77-90, doi:<http://dx.doi.org/10.1111/bld.12258> (2019).

3 Bigby, C. Social inclusion and people with intellectual disability and challenging behaviour: A systematic review. *Journal of Intellectual and Developmental Disability* **37**, 360-374, doi:<http://dx.doi.org/10.3109/13668250.2012.721878> (2012).

4 Bigby, C., Anderson, S. & Cameron, N. Identifying conceptualizations and theories of change embedded in interventions to facilitate community participation for people with intellectual disability: A scoping review. *Journal of Applied Research in Intellectual Disabilities* **31**, 165-180, doi:<http://dx.doi.org/10.1111/jar.12390> (2018).

5 Bondár, R. Z. *et al.* The effects of physical activity or sport‐based interventions on psychological factors in adults with intellectual disabilities: a systematic review. *Journal of Intellectual Disability Research* **64**, 69-92, doi:10.1111/jir.12699 (2020).

6 Bourne, J., Andersen-Warren, M. & Hackett, S. A systematic review to investigate dramatherapy group work with working age adults who have a mental health problem. *The Arts in Psychotherapy* **61**, 1-9, doi:<http://dx.doi.org/10.1016/j.aip.2018.08.001> (2018).

7 Bundock, K. E. & Hewitt, O. A review of social skills interventions for adults with autism and intellectual disability. *Tizard Learning Disability Review* **22**, 148-158, doi:10.1108/TLDR-05-2016-0015 (2017).

8 Clatworthy, J., Hinds, J. & M. Camic, P. Gardening as a mental health intervention: a review. *Mental Health Review Journal* **18**, 214-225, doi:10.1108/MHRJ-02-2013-0007 (2013).

9 Coren, E., Hutchfield, J., Thomae, M. & Gustafsson, C. Parent training support for intellectually disabled parents. *Cochrane Database of Systematic Reviews*, N.PAG-N.PAG (2010).

10 Coren, E., Ramsbotham, K. & Gschwandtner, M. Parent training interventions for parents with intellectual disability. *Cochrane Database of Systematic Reviews* **7**, doi:<http://dx.doi.org/10.1002/14651858.CD007987.pub3> (2018).

11 Coren, E., Thomae, M. & Hutchfield, J. Parenting training for intellectually disabled parents: A Cochrane systematic review. *Research on Social Work Practice* **21**, 432-441, doi:<http://dx.doi.org/10.1177/1049731511399586> (2011).

12 Exell, R., Hilari, K. & Behn, N. Interventions that support adults with brain injuries, learning disabilities and autistic spectrum disorders in dating or romantic relationships: a systematic review. *Disability and Rehabilitation.*, doi:<http://dx.doi.org/10.1080/09638288.2020.1845824> (2020).

13 Fernandez-Sotos, P., Fernandez-Caballero, A. & Rodriguez-Jimenez, R. Virtual reality for psychosocial remediation in schizophrenia: A systematic review. *The European Journal of Psychiatry* **34**, 1-10, doi:<http://dx.doi.org/10.1016/j.ejpsy.2019.12.003> (2020).

14 Finkel, D. & Bat Or, M. The Open Studio Approach to Art Therapy: A Systematic Scoping Review. *Frontiers in Psychology* **11**, doi:10.3389/fpsyg.2020.568042 (2020).

15 Firth, J., Cotter, J., Elliott, R., French, P. & Yung, A. A systematic review and meta-analysis of exercise interventions in schizophrenia patients. *Psychological Medicine* **45**, 1343-1361, doi:<http://dx.doi.org/10.1017/S0033291714003110> (2015).

16 Firth, J. *et al.* Motivating factors and barriers towards exercise in severe mental illness: a systematic review and meta-analysis. *Psychol Med* **46**, 2869-2881, doi:10.1017/s0033291716001732 (2016).

17 Fortuna, K. L. *et al.* Digital Peer Support Mental Health Interventions for People With a Lived Experience of a Serious Mental Illness: Systematic Review. *JMIR Ment Health* **7**, e16460, doi:10.2196/16460 (2020).

18 Fuhr, D. C. *et al.* Effectiveness of peer-delivered interventions for severe mental illness and depression on clinical and psychosocial outcomes: a systematic review and meta-analysis. *Soc Psychiatry Psychiatr Epidemiol* **49**, 1691-1702, doi:10.1007/s00127-014-0857-5 (2014).

19 Genter, C., Roberts, A., Richardson, J. & Sheaff, M. The contribution of allotment gardening to health and wellbeing: A systematic review of the literature. *British Journal of Occupational Therapy* **78**, 593-605, doi:10.1177/0308022615599408 (2015).

20 Geretsegger, M. *et al.* Music therapy for people with schizophrenia and schizophrenia-like disorders. *Cochrane Database of Systematic Reviews*, doi:10.1002/14651858.CD004025.pub4 (2017).

21 Gonzalvez, C. *et al.* Efficacy of sex education programs for people with intellectual disabilities: A meta-analysis. *Sexuality and Disability* **36**, 331-347, doi:<http://dx.doi.org/10.1007/s11195-018-9545-9> (2018).

22 Grant, N., Lawrence, M., Preti, A., Wykes, T. & Cella, M. Social cognition interventions for people with schizophrenia: a systematic review focussing on methodological quality and intervention modality. *Clin Psychol Rev* **56**, 55-64, doi:10.1016/j.cpr.2017.06.001 (2017).

23 Hallett, R. Physical activity for autistic adults: Recommendations for a shift in approach. *Autism in Adulthood* **1**, 173-181, doi:<http://dx.doi.org/10.1089/aut.2019.0016> (2019).

24 Howarth, S., Morris, D., Newlin, M. & Webber, M. Health and social care interventions which promote social participation for adults with learning disabilities: A review. *British Journal of Learning Disabilities* **44**, 3-15, doi:<http://dx.doi.org/10.1111/bld.12100> (2016).

25 Hutchinson, N. & Bodicoat, A. The effectiveness of intensive interaction, a systematic literature review. *Journal of Applied Research in Intellectual Disabilities* **28**, 437-454, doi:<http://dx.doi.org/10.1111/jar.12138> (2015).

26 Hutzler, Y. & Korsensky, O. Motivational correlates of physical activity in persons with an intellectual disability: A systematic literature review. *Journal of Intellectual Disability Research* **54**, 767-786, doi:<http://dx.doi.org/10.1111/j.1365-2788.2010.01313.x> (2010).

27 Kaltenthaler, E., Pandor, A. & Wong, R. The effectiveness of sexual health interventions for people with severe mental illness: A systematic review. *Health Technology Assessment* **18**, 1-73, doi:<http://dx.doi.org/10.3310/hta18010> (2014).

28 Kamioka, H. *et al.* Effectiveness of animal-assisted therapy: A systematic review of randomized controlled trials. *Complement Ther Med* **22**, 371-390, doi:10.1016/j.ctim.2013.12.016 (2014).

29 Lindsay, S. & Lamptey, D. L. Pedestrian navigation and public transit training interventions for youth with disabilities: a systematic review. *Disability and Rehabilitation* **41**, 2607-2621, doi:<http://dx.doi.org/10.1080/09638288.2018.1471165> (2019).

30 Lorenc, T. *et al.* Support for adults with autism spectrum disorder without intellectual impairment: Systematic review. *Autism* **22**, 654-668, doi:<http://dx.doi.org/10.1177/1362361317698939> (2018).

31 Lorenc, T. *et al.* Preventative co-ordinated low-level support for adults with high-functioning autism: systematic review and service mapping. (EPPI-Centre, Social Science Research Unit, UCL Institute of Education, University College London, London, 2016).

32 Louw, J. S., Kirkpatrick, B. & Leader, G. Enhancing social inclusion of young adults with intellectual disabilities: A systematic review of original empirical studies. *Journal of Applied Research in Intellectual Disabilities.*, doi:<http://dx.doi.org/10.1111/jar.12678> (2019).

33 Ma, Z., Dhir, P., Perrier, L., Bayley, M. & Munce, S. The Impact of Vocational Interventions on Vocational Outcomes, Quality of Life, and Community Integration in Adults with Childhood Onset Disabilities: A Systematic Review. *Journal of Occupational Rehabilitation* **30**, 1-21, doi:10.1007/s10926-019-09854-1 (2020).

34 Masi, C. M., Chen, H.-Y., Hawkley, L. C. & Cacioppo, J. T. A meta-analysis of interventions to reduce loneliness. *Pers Soc Psychol Rev* **15**, 219-266, doi:10.1177/1088868310377394 (2011).

35 Maujean, A., Pepping, C. A. & Kendall, E. A systematic review of randomized controlled trials of animal-assisted therapy on psychosocial outcomes. *Anthrozoös* **28**, 23-36, doi:10.2752/089279315X14129350721812 (2015).

36 McCann, E., Marsh, L. & Brown, M. People with intellectual disabilities, relationship and sex education programmes: A systematic review. *Health Education Journal* **78**, 885-900, doi:10.1177/0017896919856047 (2019).

37 Morin, L. & Franck, N. Rehabilitation interventions to promote recovery from schizophrenia: A systematic review. *Frontiers in Psychiatry Vol 8 2017, ArtID 100* **8**, doi:<http://dx.doi.org/10.3389/fpsyt.2017.00100> (2017).

38 Naslund, J. A., Marsch, L. A., McHugo, G. J. & Bartels, S. J. Emerging mHealth and eHealth interventions for serious mental illness: A review of the literature. *Journal of Mental Health* **24**, 320-331, doi:<http://dx.doi.org/10.3109/09638237.2015.1019054> (2015).

39 Nguyen, L. *et al.* Understanding the essential components and experiences of youth with autism spectrum disorders in peer mentorship programmes during the transition to adulthood: A qualitative meta‐ethnography. *Child Care, Health and Development* **46**, 667-681, doi:<http://dx.doi.org/10.1111/cch.12804> (2020).

40 Palmen, A., Didden, R. & Lang, R. A systematic review of behavioral intervention research on adaptive skill building in high-functioning young adults with autism spectrum disorder. *Research in Autism Spectrum Disorders* **6**, 602-617, doi:<http://dx.doi.org/10.1016/j.rasd.2011.10.001> (2012).

41 Pallathra, A. A., Cordero, L., Wong, K. & Brodkin, E. S. Psychosocial Interventions Targeting Social Functioning in Adults on the Autism Spectrum: a Literature Review. *Current Psychiatry Reports* **21**, 5, doi:<http://dx.doi.org/10.1007/s11920-019-0989-0> (2019).

42 Pescheny, J. V., Randhawa, G. & Pappas, Y. The impact of social prescribing services on service users: a systematic review of the evidence. *Eur J Public Health* **30**, 664-673, doi:10.1093/eurpub/ckz078 (2020).

43 Petroutsou, A., Hassiotis, A. & Afia, A. Loneliness in people with intellectual and developmental disorders across the lifespan: A systematic review of prevalence and interventions. *Journal of Applied Research in Intellectual Disabilities* **31**, 643-658, doi:<http://dx.doi.org/10.1111/jar.12432> (2018).

44 Picton, C., Fernandez, R., Moxham, L. & Patterson, C. F. Experiences of outdoor nature-based therapeutic recreation programs for persons with a mental illness: a qualitative systematic review. *JBI Evidence Synthesis* **18** (2020).

45 Puolakka, K. & Pitkänen, A. Effectiveness of psychosocial interventions on quality of life of patients with schizophrenia and related disorders: A systematic review. *Arch Psychiatr Nurs* **33**, 73-82, doi:10.1016/j.apnu.2019.07.001 (2019).

46 Quirk, H., Crank, H., Harrop, D., Hock, E. & Copeland, R. Understanding the experience of initiating community-based physical activity and social support by people with serious mental illness: A systematic review using a meta-ethnographic approach. *Systematic Reviews* **6**, 214, doi:10.1186/s13643-017-0596-2 (2017).

47 Ratti, V. *et al.* The effectiveness of person-centred planning for people with intellectual disabilities: A systematic review. *Research in Developmental Disabilities* **57**, 63-84, doi:<http://dx.doi.org/10.1016/j.ridd.2016.06.015> (2016).

48 Roche, B., Tuck, A., Ware, E. & McKenzie, K. Promoting Health and Well-Being through Social Inclusion in Toronto: A Scoping Review of Literature Reviews of Interventions to Promote Social Inclusion. (Wellesley Institute and Toronto Public Health, Toronto, Canada, 2019).

49 Sala, G., Hooley, M., Attwood, T., Mesibov, G. B. & Stokes, M. A. Autism and Intellectual Disability: A Systematic Review of Sexuality and Relationship Education. *Sexuality and Disability* **37**, 353-382, doi:<http://dx.doi.org/10.1007/s11195-019-09577-4> (2019).

50 Schepens, H. R. M. M., Van Puyenbroeck, J. & Maes, B. How to improve the quality of life of elderly people with intellectual disability: A systematic literature review of support strategies. *Journal of Applied Research in Intellectual Disabilities* **32**, 483-521, doi:10.1111/jar.12559 (2019).

51 Siette, J., Cassidy, M. & Priebe, S. Effectiveness of befriending interventions: a systematic review and meta-analysis. *BMJ Open* **7**, e014304, doi:10.1136/bmjopen-2016-014304 (2017).

52 Soundy, A. *et al.* The transcending benefits of physical activity for individuals with schizophrenia: a systematic review and meta-ethnography. *Psychiatry Res* **220**, 11-19, doi:10.1016/j.psychres.2014.07.083 (2014).

53 Takahashi, H., Matsushima, K. & Kato, T. The effectiveness of dance/movement therapy interventions for autism spectrum disorder: A systematic review. *American Journal of Dance Therapy* **41**, 55-74, doi:<http://dx.doi.org/10.1007/s10465-019-09296-5> (2019).

54 Tint, A., Thomson, K. & Weiss, J. A systematic literature review of the physical and psychosocial correlates of Special Olympics participation among individuals with intellectual disability. *Journal of Intellectual Disability Research* **61**, 301-324, doi:<http://dx.doi.org/10.1111/jir.12295> (2017).

55 Tobin, M. C., Drager, K. D. & Richardson, L. F. A systematic review of social participation for adults with autism spectrum disorders: Support, social functioning, and quality of life. *Research in Autism Spectrum Disorders* **8**, 214-229, doi:<http://dx.doi.org/10.1016/j.rasd.2013.12.002> (2014).

56 Walker, G. & Bryant, W. Peer support in adult mental health services: A metasynthesis of qualitative findings. *Psychiatric Rehabilitation Journal* **36**, 28-34, doi:<http://dx.doi.org/10.1037/h0094744> (2013).

57 Webber, M. & Fendt-Newlin, M. A review of social participation interventions for people with mental health problems. *Soc Psychiatry Psychiatr Epidemiol* **52**, 369-380, doi:10.1007/s00127-017-1372-2 (2017).

58 White, S. *et al.* The effectiveness of one-to-one peer support in mental health services: a systematic review and meta-analysis. *BMC Psychiatry* **20**, 534, doi:10.1186/s12888-020-02923-3 (2020).

59 Williams, E., Dingle, G. A. & Clift, S. A systematic review of mental health and wellbeing outcomes of group singing for adults with a mental health condition. *European Journal of Public Health* **28**, 1035-1042, doi:10.1093/eurpub/cky115 (2018).

60 Wilson, S., McKenzie, K., Quayle, E. & Murray, G. A systematic review of interventions to promote social support and parenting skills in parents with an intellectual disability. *Child: Care, Health and Development* **40**, 7-19, doi:<http://dx.doi.org/10.1111/cch.12023> (2014).

61 Newlin, M., Webber, M., Morris, D. & Howarth, S. Social Participation Interventions for Adults with Mental Health Problems: A Review and Narrative Synthesis. *Social Work Research* **39**, 167-180, doi:10.1093/swr/svv015 (2015).

62 Spain, D. & Blainey, S. H. Group social skills interventions for adults with high-functioning autism spectrum disorders: A systematic review. *Autism* **19**, 874-886, doi:<http://dx.doi.org/10.1177/1362361315587659> (2015).

63 Pitt, V. *et al.* Consumer-providers of care for adult clients of statutory mental health services. *Cochrane Database Syst Rev*, Cd004807, doi:10.1002/14651858.CD004807.pub2 (2013).

64 Spain, D. *et al.* Family therapy for autism spectrum disorders. *Cochrane Database of Systematic Reviews* **5**, CD011894, doi:<https://dx.doi.org/10.1002/14651858.CD011894.pub2> (2017).

65 Crowe, M. *et al.* Disorder-specific psychosocial interventions for bipolar disorder-A systematic review of the evidence for mental health nursing practice. *International Journal of Nursing Studies* **47**, 896-908, doi:<http://dx.doi.org/10.1016/j.ijnurstu.2010.02.012> (2010).

66 Feng, X. *et al.* Morita therapy for schizophrenia: An updated meta-analysis. *Asian Journal of Psychiatry* **53**, doi:<http://dx.doi.org/10.1016/j.ajp.2020.102169> (2020).

67 Seewooruttun, L. & Scior, K. Interventions aimed at increasing knowledge and improving attitudes towards people with intellectual disabilities among lay people. *Research in Developmental Disabilities* **35**, 3482-3495, doi:<http://dx.doi.org/10.1016/j.ridd.2014.07.028> (2014).

68 Lowe, K., Felce, D. & Blackman, D. Challenging behaviour: the effectiveness of specialist support teams. *Journal of Intellectual Disability Research* **40**, 336-347 (1996).

69 Magito-McLaughlin, D., Mullen-James, K., Anderson-Ryan, K. & Carr, E. G. Best Practices: Finding a New Direction for Christos. *Journal of Positive Behavior Interventions* **4**, 157-165, doi:10.1177/10983007020040030401 (2002).

70 Carr, E. G. *et al.* Comprehensive Multisituational Intervention for Problem Behavior in the Community: Long-Term Maintenance and Social Validation. *Journal of Positive Behavior Interventions* **1**, 5-25, doi:10.1177/109830079900100103 (1999).

71 McClean, B., Grey, I. M. & McCracken, M. An evaluation of positive behavioural support for people with very severe challenging behaviours in community-based settings. *Journal of Intellectual Disabilities* **11**, 281-301, doi:10.1177/1744629507080791 (2007).

72 Heller, T., Miller, A. B., Hsieh, K. & Sterns, H. Later-life planning: Promoting knowledge of options and choice-making. *Mental Retardation* **38**, 395-406, doi:10.1352/0047-6765(2000)038<0395:LPPKOO>2.0.CO;2 (2000).

73 Magito-McLaughlin, D., Spinosa, T. & Marsalis, M. Overcoming the barriers: moving towards a service model that is conducive to person-centred planning. *Person-Centred Planning: research, practice and future directions, Baltimore, MD: Paul H. Brookes Publishing* (2002).

74 Malette, P. in *Person-centred planning. Research, practice, and future directions* (eds S. Holburn & Vietze P.M.) (Paul H. Brookes Publishing Co., 2002).

75 Robertson, J. *et al.* Longitudinal Analysis of the Impact and Cost of Person-Centered Planning for People With Intellectual Disabilities in England. *American Journal on Mental Retardation* **111**, 400-416, doi:10.1352/0895-8017(2006)111[400:Laotia]2.0.Co;2 (2006).

76 Hagner, D., Helm, D. T. & Butterworth, J. "This is Your Meeting": A Qualitative Study of Person-Centered Planning. *Mental Retardation* **34**, 159 (1996).

77 Parley, F. F. Person-Centred Outcomes: Are Outcomes Improved Where a Person-Centred Care Model is Used? *Journal of Learning Disabilities* **5**, 299-308, doi:10.1177/146900470100500402 (2001).

78 Espiner, D. & Hartnett, F. M. ‘I felt I was in control of the meeting’: facilitating planning with adults with an intellectual disability. *British Journal of Learning Disabilities* **40**, 62-70, doi:<https://doi.org/10.1111/j.1468-3156.2011.00684.x> (2012).

79 Smith, D. & Carey, E. Person-centred care planning for clients with complex needs. *Learning Disability Practice* **16**, 20– 23., doi:10.7748/ldp2013.12.16.10.20.e1465 (2013).

80 Jensen, C. C., McConnachie, G. & Pierson, T. Long-Term Multicomponent Intervention to Reduce Severe Problem Behavior: A 63-Month Evaluation. *Journal of Positive Behavior Interventions* **3**, 225-236, doi:10.1177/109830070100300404 (2001).

81 Mansell, J., McGill, P. & Emerson, E. in *International Review of Research in Mental Retardation* Vol. 24 245-298 (Academic Press, 2001).

82 Bigby, C. Transferring responsibility: The nature and effectiveness of parental planning for the future of adults with intellectual disability who remain at home until mid-life. *Journal of Intellectual & Developmental Disability* **21**, 295-312, doi:10.1080/13668259600033211 (1996).

83 Lowe, K. & De Paiva, S. Clients' community and social contacts: results of a 5‐year longitudinal study. *Journal of Intellectual Disability Research* **35**, 308-323 (1991).

84 Broer, T., Nieboer, A. P., Strating, M. M. H., Michon, H. W. C. & Bal, R. A. Constructing the social: an evaluation study of the outcomes and processes of a ‘social participation’improvement project. *Journal of Psychiatric and Mental Health Nursing* **18**, 323-332 (2011).

85 Di Terlizzi, M. Life History: The Impact of a Changing Service Provision on an Individual with Learning Disabilities. *Disability & Society* **9**, 501-517, doi:10.1080/09687599466780481 (1994).

86 Aleman, A. *et al.* Treatment of negative symptoms: Where do we stand, and where do we go? *Schizophrenia Research* **186**, 55-62, doi:<https://doi.org/10.1016/j.schres.2016.05.015> (2017).

87 Bellack, A. S. Skills Training for People with Severe Mental Illness. *Psychiatric Rehabilitation Journal* **27**, 375-391, doi:10.2975/27.2004.375.391 (2004).

88 Elis, O., Caponigro, J. M. & Kring, A. M. Psychosocial treatments for negative symptoms in schizophrenia: Current practices and future directions. *Clinical Psychology Review* **33**, 914-928, doi:<https://doi.org/10.1016/j.cpr.2013.07.001> (2013).

89 Kurtz, M. M. & Mueser, K. T. A meta-analysis of controlled research on social skills training for schizophrenia. *Journal of Consulting and Clinical Psychology* **76**, 491-504, doi:10.1037/0022-006X.76.3.491 (2008).

90 Mueser, K. T. & Penn, D. L. Pilling and colleagues (2002) recently published a meta-analysis examining the effects of social skills training on schizophrenia (this review also included a meta-analysis of research on cognitive remediation for schizophrenia which is not discussed in this comment). *Psychological Medicine* **34**, 1365-1367, doi:10.1017/S0033291704213848 (2004).

91 Pfammatter, M., Junghan, U. M. & Brenner, H. D. Efficacy of Psychological Therapy in Schizophrenia: Conclusions From Meta-analyses. *Schizophrenia Bulletin* **32**, S64-S80, doi:10.1093/schbul/sbl030 (2006).

92 Pilling, S. *et al.* Psychological treatments in schizophrenia: II. Meta-analyses of randomized controlled trials of social skills training and cognitive remediation. *Psychological Medicine* **32**, 783-791, doi:10.1017/S0033291702005640 (2002).

93 Kayser, N., Sarfati, Y., Besche, C. & Hardy-Baylé, M. C. Elaboration of a rehabilitation method based on a pathogenetic hypothesis of "theory of mind" impairment in schizophrenia. *Neuropsychological Rehabilitation* **16**, 83-95, doi:10.1080/09602010443000236 (2006).

94 Hayes, R. L., Halford, W. K. & Varghese, F. T. Social skills training with chronic schizophrenic patients: Effects on negative symptoms and community functioning. *Behavior Therapy* **26**, 433-449, doi:<https://doi.org/10.1016/S0005-7894(05)80092-9> (1995).

95 Rus-Calafell, M., Gutiérrez-Maldonado, J. & Ribas-Sabaté, J. Improving social behaviour in schizophrenia patients using an integrated virtual reality programme: a case study. *Stud Health Technol Inform* **181**, 283-286 (2012).

96 Rus-Calafell, M., Gutiérrez-Maldonado, J., Ortega-Bravo, M., Ribas-Sabaté, J. & Caqueo-Urízar, A. A brief cognitive-behavioural social skills training for stabilised outpatients with schizophrenia: a preliminary study. *Schizophr Res* **143**, 327-336, doi:10.1016/j.schres.2012.11.014 (2013).

97 Rus-Calafell, M., Gutiérrez-Maldonado, J. & Ribas-Sabaté, J. A virtual reality-integrated program for improving social skills in patients with schizophrenia: a pilot study. *J Behav Ther Exp Psychiatry* **45**, 81-89, doi:10.1016/j.jbtep.2013.09.002 (2014).

98 Marzillier, J. S., Lambert, C. & Kellett, J. A controlled evaluation of systematic desensitisation and social skills training for socially inadequate psychiatric patients. *Behav Res Ther* **14**, 225-238, doi:10.1016/0005-7967(76)90015-2 (1976).

99 Adery, L. H. *et al.* The acceptability and feasibility of a novel virtual reality based social skills training game for schizophrenia: Preliminary findings. *Psychiatry Research* **270**, 496-502, doi:<https://doi.org/10.1016/j.psychres.2018.10.014> (2018).

100 Park, K.-M. *et al.* A virtual reality application in role-plays of social skills training for schizophrenia: A randomized, controlled trial. *Psychiatry Research* **189**, 166-172, doi:<https://doi.org/10.1016/j.psychres.2011.04.003> (2011).

101 Björkman, T., Hansson, L. & Sandlund, M. Outcome of case management based on the strengths model compared to standard care. A randomised controlled trial. *Soc Psychiatry Psychiatr Epidemiol* **37**, 147-152, doi:10.1007/s001270200008 (2002).

102 Hasson-Ohayon, I., Roe, D. & Kravetz, S. A randomized controlled trial of the effectiveness of the illness management and recovery program. *Psychiatr Serv* **58**, 1461-1466, doi:10.1176/ps.2007.58.11.1461 (2007).

103 Aberg-Wistedt, A., Cressell, T., Lidberg, Y., Liljenberg, B. & Osby, U. Two-year outcome of team-based intensive case management for patients with schizophrenia. *Psychiatr Serv* **46**, 1263-1266, doi:10.1176/ps.46.12.1263 (1995).

104 Glynn, S. M. *et al.* Supplementing clinic-based skills training with manual-based community support sessions: effects on social adjustment of patients with schizophrenia. *Am J Psychiatry* **159**, 829-837, doi:10.1176/appi.ajp.159.5.829 (2002).

105 Bustillo, J. R., Lauriello, J., Horan, W. P. & Keith, S. J. The Psychosocial Treatment of Schizophrenia: An Update. *American Journal of Psychiatry* **158**, 163-175, doi:10.1176/appi.ajp.158.2.163 (2001).

106 Turner, D. T., van der Gaag, M., Karyotaki, E. & Cuijpers, P. Psychological Interventions for Psychosis: A Meta-Analysis of Comparative Outcome Studies. *American Journal of Psychiatry* **171**, 523-538, doi:10.1176/appi.ajp.2013.13081159 (2014).

107 Conoley, C. W. & Garber, R. A. Effects of reframing and self-control directives on loneliness, depression, and controllability. *Journal of Counseling Psychology* **32**, 139-142, doi:10.1037/0022-0167.32.1.139 (1985).

108 Williams, A. *et al.* Psychosocial effects of the BOOT STRAP intervention in Navy recruits. *Mil Med* **169**, 814-820, doi:10.7205/milmed.169.10.814 (2004).

109 Horan, W. P. *et al.* Social cognitive skills training in schizophrenia: An initial efficacy study of stabilized outpatients. *Schizophrenia Research* **107**, 47-54, doi:10.1016/j.schres.2008.09.006 (2009).

110 Roberts, D. L. *et al.* A randomized, controlled trial of Social Cognition and Interaction Training (SCIT) for outpatients with schizophrenia spectrum disorders. *British Journal of Clinical Psychology* **53**, 281-298, doi:10.1111/bjc.12044 (2014).

111 Wang, Y. *et al.* Social cognition and interaction training for patients with stable schizophrenia in Chinese community settings. *Psychiatry Research* **210**, 751-755, doi:10.1016/j.psychres.2013.08.038 (2013).

112 Combs, D. R. *et al.* Social Cognition and Interaction Training (SCIT) for inpatients with schizophrenia spectrum disorders: Preliminary findings. *Schizophrenia Research* **91**, 112-116, doi:10.1016/j.schres.2006.12.010 (2007).

113 Taylor, R. *et al.* Tackling Social Cognition in Schizophrenia: A Randomized Feasibility Trial. *Behav Cogn Psychother* **9**, 1-12 (2015).

114 Hasson-Ohayon, I., Mashiach-Eizenberg, M., Avidan, M., Roberts, D. L. & Roe, D. Social cognition and interaction training: preliminary results of an RCT in a community setting in Israel. *Psychiatr Serv* **65**, 555-558, doi:10.1176/appi.ps.201300146 (2014).

115 Gohar, S. M., Hamdi, E., El Ray, L. A., Horan, W. P. & Green, M. F. Adapting and evaluating a social cognitive remediation program for schizophrenia in Arabic. *Schizophrenia Research* **148**, 12-17, doi:10.1016/j.schres.2013.05.008 (2013).

116 Horan, W. P. *et al.* Efficacy and specificity of Social Cognitive Skills Training for outpatients with psychotic disorders. *Journal of Psychiatric Research* **45**, 1113-1122, doi:10.1016/j.jpsychires.2011.01.015 (2011).

117 Gil Sanz, D. *et al.* Efficacy of a social cognition training program for schizophrenic patients: a pilot study. *Span J Psychol* **12**, 184-191 (2009).

118 Gil-Sanz, D., Fernández-Modamio, M., Bengochea-Seco, R., Arrieta-Rodríguez, M. & Pérez-Fuentes, G. Efficacy of the social cognition training program in a sample of outpatients with schizophrenia. *Clinical schizophrenia & related psychoses* **10**, 154-162 (2016).

119 Bechi, M. *et al.* Combined social cognitive and neurocognitive rehabilitation strategies in schizophrenia: Neuropsychological and psychopathological influences on Theory of Mind improvement. *Psychological Medicine* **45**, 3147-3157, doi:10.1017/S0033291715001129 (2015).

120 Bechi, M. *et al.* Theory of mind and emotion processing training for patients with schizophrenia: Preliminary findings. *Psychiatry Research* **198**, 371-377, doi:10.1016/j.psychres.2012.02.004 (2012).

121 Stravynski, A., Marks, I. & Yule, W. Social skills problems in neurotic outpatients. Social skills training with and without cognitive modification. *Arch Gen Psychiatry* **39**, 1378-1385, doi:10.1001/archpsyc.1982.04290120014003 (1982).

122 Tas, C., Danaci, A. E., Cubukcuoglu, Z. & Brüne, M. Impact of family involvement on social cognition training in clinically stable outpatients with schizophrenia - A randomized pilot study. *Psychiatry Research* **195**, 32-38, doi:10.1016/j.psychres.2011.07.031 (2012).

123 Galli Carminati, G., Gerber, F., Baud, M. A. & Baud, O. Evaluating the effects of a structured program for adults with autism spectrum disorders and intellectual disabilities. *Research in Autism Spectrum Disorders* **1**, 256-265, doi:<https://doi.org/10.1016/j.rasd.2006.11.001> (2007).

124 McGaw, S., Ball, K. & Clark, A. The effect of group intervention on the relationships of parents with intellectual disabilities. *Journal of Applied Research in Intellectual Disabilities* **15**, 354-366 (2002).

125 McConnell, D., Dalziel, A., Llewellyn, G., Laidlaw, K. & Hindmarsh, G. Strengthening the social relationships of mothers with learning difficulties. *British Journal of Learning Disabilities* **37**, 66-75 (2009).

126 Iconaru, E. I. & Ciucurel, C. Developing Social and Civic Competencies in People with Intellectual Disabilities from a Family Center through an Adapted Training Module. *Procedia - Social and Behavioral Sciences* **116**, 3303-3307, doi:<https://doi.org/10.1016/j.sbspro.2014.01.752> (2014).

127 Turner-Brown, L. M., Perry, T. D., Dichter, G. S., Bodfish, J. W. & Penn, D. L. Brief report: feasibility of social cognition and interaction training for adults with high functioning autism. *Journal of autism and developmental disorders* **38**, 1777-1784, doi:10.1007/s10803-008-0545-y (2008).

128 Fullerton, A. & Coyne, P. Developing Skills and Concepts for Self-Determination in Young Adults with Autism. *Focus on Autism and Other Developmental Disabilities* **14**, 42-52, doi:10.1177/108835769901400106 (1999).

129 Pugliese, C. E. & White, S. W. Brief report: Problem solving therapy in college students with autism spectrum disorders: Feasibility and preliminary efficacy. *Journal of autism and developmental disorders* **44**, 719-729 (2014).

130 Lovett, S. & Rehfeldt, R. A. An evaluation of multiple exemplar instruction to teach perspective-taking skills to adolescents with Asperger Syndrome. *Behavioral Development Bulletin* **19**, 22 (2014).

131 Minihan, A. *Social Skills Training for Adults with Asperger Syndrome-: Programme Design, Implementation and Evaluation*, Trinity College Dublin, (2007).

132 Howlin, P. & Yates, P. The Potential Effectiveness of Social Skills Groups for Adults with Autism. *Autism* **3**, 299-307, doi:10.1177/1362361399003003007 (1999).

133 Ashman, R., Banks, K., Philip, R. C. M., Walley, R. & Stanfield, A. C. A pilot randomised controlled trial of a group based social skills intervention for adults with autism spectrum disorder. *Research in Autism Spectrum Disorders* **43-44**, 67-75, doi:<https://doi.org/10.1016/j.rasd.2017.08.001> (2017).

134 Gantman, A., Kapp, S. K., Orenski, K. & Laugeson, E. A. Social skills training for young adults with high-functioning autism spectrum disorders: a randomized controlled pilot study. *J Autism Dev Disord* **42**, 1094-1103, doi:10.1007/s10803-011-1350-6 (2012).

135 Laugeson, E. A., Gantman, A., Kapp, S. K., Orenski, K. & Ellingsen, R. A randomized controlled trial to improve social skills in young adults with autism spectrum disorder: The UCLA PEERS® program. *Journal of autism and developmental disorders* **45**, 3978-3989 (2015).

136 White, S. W., Scarpa, A., Conner, C. M., Maddox, B. B. & Bonete, S. Evaluating Change in Social Skills in High-Functioning Adults With Autism Spectrum Disorder Using a Laboratory-Based Observational Measure. *Focus on Autism and Other Developmental Disabilities* **30**, 3-12, doi:10.1177/1088357614539836 (2014).

137 McVey, A. J. *et al.* A Replication and Extension of the PEERS® for Young Adults Social Skills Intervention: Examining Effects on Social Skills and Social Anxiety in Young Adults with Autism Spectrum Disorder. *J Autism Dev Disord* **46**, 3739-3754, doi:10.1007/s10803-016-2911-5 (2016).

138 Tiger, J. H., Bouxsein, K. J. & Fisher, W. W. Treating excessively slow responding of a young man with Asperger syndrome using differential reinforcement of short response latencies. *J Appl Behav Anal* **40**, 559-563, doi:10.1901/jaba.2007.40-559 (2007).

139 Perdue, J. *The effectiveness of video modeling to teach social skills to young adults with autism spectrum disorder*. (University of Nebraska at Omaha, 2015).

140 Shireman, M. L., Lerman, D. C. & Hillman, C. B. Teaching social play skills to adults and children with autism as an approach to building rapport. *Journal of applied behavior analysis* **49**, 512-531 (2016).

141 Gaylord-Ross, R. J., Haring, T. G., Breen, C. & Pitts-Conway, V. The training and generalization of social interaction skills with autistic youth. *J Appl Behav Anal* **17**, 229-247, doi:10.1901/jaba.1984.17-229 (1984).

142 Kandalaft, M. R., Didehbani, N., Krawczyk, D. C., Allen, T. T. & Chapman, S. B. Virtual reality social cognition training for young adults with high-functioning autism. *Journal of autism and developmental disorders* **43**, 34-44 (2013).

143 Koegel, L. K., Ashbaugh, K., Koegel, R. L., Detar, W. J. & Regester, A. Increasing socialization in adults with Asperger's syndrome. *Psychology in the Schools* **50**, 899-909 (2013).

144 White, S. W. *et al.* Psychosocial and computer-assisted intervention for college students with autism spectrum disorder: Preliminary support for feasibility. *Education and training in autism and developmental disabilities* **51**, 307 (2016).

145 Elgie, S. & Maguire, N. Intensive interaction with a woman with multiple and profound disabilities: A case study. *Tizard Learning Disability Review* (2001).

146 Leaning, B. & Watson, T. From the inside looking out–an Intensive Interaction group for people with profound and multiple learning disabilities. *British Journal of Learning Disabilities* **34**, 103-109 (2006).

147 Lovell, D. M., Jones, R. S. P. & Ephraim, G. The effect of intensive interaction on the sociability of a man with severe intellectual disabilities. (1998).

148 Nind, M. Intensive interaction and autism: a useful approach? *British Journal of Special Education* **26**, 96-102 (1999).

149 Nind, M. Efficacy of Intensive Interaction: developing sociability and communication in people with severe and complex learning difficulties using an approach based on caregiver‐infant interaction. *European Journal of Special Needs Education* **11**, 48-66, doi:10.1080/0885625960110104 (1996).

150 Samuel, J., Nind, M., Volans, A. & Scriven, I. An evaluation of Intensive Interaction in community living settings for adults with profound intellectual disabilities. *Journal of Intellectual Disabilities* **12**, 111-126 (2008).

151 Zeedyk, M. S., Caldwell, P. & Davies, C. E. How rapidly does intensive interaction promote social engagement for adults with profound learning disabilities? *European Journal of Special Needs Education* **24**, 119-137 (2009).

152 Zeedyk, M. S., Davies, C., Parry, S. & Caldwell, P. Fostering social engagement in Romanian children with communicative impairments: The experiences of newly trained practitioners of Intensive Interaction. *British Journal of Learning Disabilities* **37**, 186-196 (2009).

153 Forster, S. & Taylor, M. Using intensive interaction: a case study. *Acquiring Knowledge in Speech, Language and Hearing* **8**, 12-15 (2006).

154 Koegel, L. K., Ashbaugh, K., Navab, A. & Koegel, R. L. Improving empathic communication skills in adults with autism spectrum disorder. *Journal of autism and developmental disorders* **46**, 921-933 (2016).

155 Mason, R. A., Rispoli, M., Ganz, J. B., Boles, M. B. & Orr, K. Effects of video modeling on communicative social skills of college students with Asperger syndrome. *Dev Neurorehabil* **15**, 425-434, doi:10.3109/17518423.2012.704530 (2012).

156 Trepagnier, C. Y., Olsen, D. E., Boteler, L. & Bell, C. A. Virtual conversation partner for adults with autism. *Cyberpsychol Behav Soc Netw* **14**, 21-27, doi:10.1089/cyber.2009.0255 (2011).

157 Koegel, L. K., Navab, A., Ashbaugh, K. & Koegel, R. L. Using Reframing to Reduce Negative Statements in Social Conversation for Adults With Autism Spectrum Disorder. *Journal of Positive Behavior Interventions* **18**, 133-144, doi:10.1177/1098300715596136 (2015).

158 Palmen, A., Didden, R. & Arts, M. Improving question asking in high-functioning adolescents with autism spectrum disorders: effectiveness of small-group training. *Autism* **12**, 83-98, doi:10.1177/1362361307085265 (2008).

159 Sperry, L. A. & Mesibov, G. B. Perceptions of social challenges of adults with autism spectrum disorder. *Autism* **9**, 362-376, doi:10.1177/1362361305056077 (2005).

160 Samuels, R. & Stansfield, J. The effectiveness of social stories™ to develop social interactions with adults with characteristics of autism spectrum disorder. *British Journal of Learning Disabilities* **40**, 272-285, doi:<https://doi.org/10.1111/j.1468-3156.2011.00706.x> (2012).

161 Newey, I., Collins, S. & Fowler, D. Evaluating psychological interventions for young men with Asperger syndrome: Cognitive behavioural therapy to address anxiety and teaching theory of mind to address social functioning. *Journal of Intellectual Disability Research* **48** (2004).

162 Bölte, S. *et al.* The development and evaluation of a computer-based program to test and to teach the recognition of facial affect. *Int J Circumpolar Health* **61 Suppl 2**, 61-68, doi:10.3402/ijch.v61i0.17503 (2002).

163 Golan, O. & Baron-Cohen, S. Systemizing empathy: teaching adults with Asperger syndrome or high-functioning autism to recognize complex emotions using interactive multimedia. *Dev Psychopathol* **18**, 591-617, doi:10.1017/s0954579406060305 (2006).

164 Saban-Bezalel, R. & Mashal, N. The effects of intervention on the comprehension of irony and on hemispheric processing of irony in adults with ASD. *Neuropsychologia* **77**, 233-241, doi:10.1016/j.neuropsychologia.2015.09.004 (2015).

165 Faja, S., Aylward, E., Bernier, R. & Dawson, G. Becoming a face expert: a computerized face-training program for high-functioning individuals with autism spectrum disorders. *Dev Neuropsychol* **33**, 1-24, doi:10.1080/87565640701729573 (2008).

166 Faja, S. *et al.* The effects of face expertise training on the behavioral performance and brain activity of adults with high functioning autism spectrum disorders. *J Autism Dev Disord* **42**, 278-293, doi:10.1007/s10803-011-1243-8 (2012).

167 Mazza, M. *et al.* Could schizophrenic subjects improve their social cognition abilities only with observation and imitation of social situations? *Neuropsychological Rehabilitation* **20**, 675-703, doi:10.1080/09602011.2010.486284 (2010).

168 Bechi, M. *et al.* Theory of Mind intervention for outpatients with schizophrenia. *Neuropsychological Rehabilitation* **23**, 383-400, doi:10.1080/09602011.2012.762751 (2013).

169 Habel, U. *et al.* Training of affect recognition in schizophrenia: Neurobiological correlates. *Social Neuroscience* **5**, 92-104, doi:10.1080/17470910903170269 (2010).

170 Wölwer, W. *et al.* Remediation of impairments in facial affect recognition in schizophrenia: Efficacy and specificity of a new training program. *Schizophrenia Research* **80**, 295-303, doi:10.1016/j.schres.2005.07.018 (2005).

171 Wölwer, W. & Frommann, N. Social-cognitive remediation in schizophrenia: Generalization of effects of the training of affect recognition (TAR). *Schizophrenia Bulletin* **37**, S63-S70, doi:10.1093/schbul/sbr071 (2011).

172 Sachs, G. *et al.* Training of affect recognition (TAR) in schizophrenia—Impact on functional outcome. *Schizophrenia Research* **138**, 262-267, doi:<https://doi.org/10.1016/j.schres.2012.03.005> (2012).

173 Combs, D. R. *et al.* Attentional-shaping as a means to improve emotion perception deficits in schizophrenia. *Schizophrenia Research* **105**, 68-77, doi:10.1016/j.schres.2008.05.018 (2008).

174 Corrigan, P. W., Hirschbeck, J. N. & Wolfe, M. Memory and vigilance training to improve social perception in schizophrenia. *Schizophrenia Research* **17**, 257-265, doi:10.1016/0920-9964(95)00008-9 (1995).

175 Popova, P. *et al.* Changing facial affect recognition in schizophrenia: Effects of training on brain dynamics. *NeuroImage: Clinical* **6**, 156-165, doi:10.1016/j.nicl.2014.08.026 (2014).

176 Penn, D. L. & Combs, D. Modification of affect perception deficits in schizophrenia. *Schizophrenia Research* **46**, 217-229, doi:10.1016/S0920-9964(00)00005-0 (2000).

177 Russell, T. A., Green, M. J., Simpson, I. & Coltheart, M. Remediation of facial emotion perception in schizophrenia: Concomitant changes in visual attention. *Schizophrenia Research* **103**, 248-256, doi:10.1016/j.schres.2008.04.033 (2008).

178 Nieminen, I. *et al.* Experiences of Social Inclusion and Employment of Mental Health Service Users in a European Union Project. *International Journal of Mental Health* **41**, 3-23, doi:10.2753/IMH0020-7411410401 (2012).

179 Ramon, S., Griffiths, C. A., Nieminen, I., Pedersen, M. & Dawson, I. Towards social inclusion through lifelong learning in mental health: analysis of change in the lives of the EMILIA project service users. *Int J Soc Psychiatry* **57**, 211-223, doi:10.1177/0020764009354943 (2011).

180 Bio, D. S. & Gattaz, W. F. Vocational rehabilitation improves cognition and negative symptoms in schizophrenia. *Schizophr Res* **126**, 265-269, doi:10.1016/j.schres.2010.08.003 (2011).

181 Walsh, E., Holloway, J. & Lydon, H. An Evaluation of a Social Skills Intervention for Adults with Autism Spectrum Disorder and Intellectual Disabilities preparing for Employment in Ireland: A Pilot Study. *Journal of Autism and Developmental Disorders* **48**, 1727-1741, doi:10.1007/s10803-017-3441-5 (2018).

182 Gibson, C. B. & Carter, E. W. Video-based instruction to promote employment-related social behaviors for high school students with intellectual disability. *Inclusion* **6**, 175-193 (2018).

183 Gilson, C. B. & Carter, E. W. Promoting Social Interactions and Job Independence for College Students with Autism or Intellectual Disability: A Pilot Study. *Journal of Autism and Developmental Disorders* **46**, 3583-3596, doi:10.1007/s10803-016-2894-2 (2016).

184 Hillier, A. *et al.* Two-year evaluation of a vocational support program for adults on the autism spectrum. *Career Development for Exceptional Individuals* **30**, 35-47 (2007).

185 Hillier, A., Fish, T., Cloppert, P. & Beversdorf, D. Q. Outcomes of a Social and Vocational Skills Support Group for Adolescents and Young Adults on the Autism Spectrum. *Focus on Autism and Other Developmental Disabilities* **22**, 107-115, doi:10.1177/10883576070220020201 (2007).

186 Hillier, A. J., Fish, T., Siegel, J. H. & Beversdorf, D. Q. Social and Vocational Skills Training Reduces Self-reported Anxiety and Depression Among Young Adults on the Autism Spectrum. *Journal of Developmental and Physical Disabilities* **23**, 267-276, doi:10.1007/s10882-011-9226-4 (2011).

187 Shields-Wolfe, J. & Gallagher, P. A. Functional Utilization of Splinter Skills for the Employment of a Young Adult with Autism. *Focus on Autistic Behavior* **7**, 1-16, doi:10.1177/108835769200700401 (1992).

188 Strickland, D. C., Coles, C. D. & Southern, L. B. JobTIPS: a transition to employment program for individuals with autism spectrum disorders. *J Autism Dev Disord* **43**, 2472-2483, doi:10.1007/s10803-013-1800-4 (2013).

189 Baker-Ericzén, M. J. *et al.* Development of the Supported Employment, Comprehensive Cognitive Enhancement, and Social Skills program for adults on the autism spectrum: Results of initial study. *Autism* **22**, 6-19, doi:10.1177/1362361317724294 (2018).

190 Liu, K. P. *et al.* Effectiveness of a workplace training programme in improving social, communication and emotional skills for adults with autism and intellectual disability in Hong Kong--a pilot study. *Occup Ther Int* **20**, 198-204, doi:10.1002/oti.1356 (2013).

191 Allen, K. D., Wallace, D. P., Greene, D. J., Bowen, S. L. & Burke, R. V. Community-Based Vocational Instruction Using Videotaped Modeling for Young Adults With Autism Spectrum Disorders Performing in Air-Inflated Mascots. *Focus on Autism and Other Developmental Disabilities* **25**, 186-192, doi:10.1177/1088357610377318 (2010).

192 Burke, R. V., Andersen, M. N., Bowen, S. L., Howard, M. R. & Allen, K. D. Evaluation of two instruction methods to increase employment options for young adults with autism spectrum disorders. *Res Dev Disabil* **31**, 1223-1233, doi:10.1016/j.ridd.2010.07.023 (2010).

193 Bonete, S., Calero, M. D. & Fernández-Parra, A. Group training in interpersonal problem-solving skills for workplace adaptation of adolescents and adults with Asperger syndrome: A preliminary study. *Autism* **19**, 409-420 (2015).

194 Smith, M. J. *et al.* Virtual reality job interview training in adults with autism spectrum disorder. *Journal of autism and developmental disorders* **44**, 2450-2463 (2014).

195 Humm, L. B., Olsen, D., Be, M., Fleming, M. & Smith, M. Simulated job interview improves skills for adults with serious mental illnesses. *Stud Health Technol Inform* **199**, 50-54 (2014).

196 Morgan, L., Leatzow, A., Clark, S. & Siller, M. Interview skills for adults with autism spectrum disorder: A pilot randomized controlled trial. *Journal of Autism and Developmental Disorders* **44**, 2290-2300 (2014).

197 Cunningham, A., Sperry, L., Brady, M. P., Peluso, P. R. & Pauletti, R. E. The effects of a romantic relationship treatment option for adults with autism spectrum disorder. *Counseling Outcome Research and Evaluation* **7**, 99-110, doi:<http://dx.doi.org/10.1177/2150137816668561> (2016).

198 Ward, K. M., Windsor, R. & Atkinson, J. P. A process evaluation of the Friendships and Dating Program for adults with developmental disabilities: Measuring the fidelity of program delivery. *Research in developmental disabilities* **33**, 69-75 (2012).

199 Ward, K. M., Atkinson, J. P., Smith, C. A. & Windsor, R. A friendships and dating program for adults with intellectual and developmental disabilities: A formative evaluation. *Intellectual and developmental disabilities* **51**, 22-32 (2013).

200 Hayashi, M., Arakida, M. & Ohashi, K. The effectiveness of a sex education program facilitating social skills for people with intellectual disability in Japan. *Journal of Intellectual and Developmental Disability* **36**, 11-19 (2011).

201 Valenti-Hein, D. C., Yarnold, P. R. & Mueser, K. T. Evaluation of the dating skills program for improving heterosocial interactions in people with mental retardation. *Behav Modif* **18**, 32-46, doi:10.1177/01454455940181003 (1994).

202 Rushton, J. Learning together. *Nurs Times* **90**, 44-46 (1994).

203 Graff, H. J. *et al.* Relationships and sexuality: How is a young adult with an intellectual disability supposed to navigate? *Sexuality and Disability* **36**, 175-183 (2018).

204 Dukes, E. & McGuire, B. E. Enhancing capacity to make sexuality-related decisions in people with an intellectual disability. *J Intellect Disabil Res* **53**, 727-734, doi:10.1111/j.1365-2788.2009.01186.x (2009).

205 Gardiner, T. & Braddon, E. ‘A Right to Know’. Facilitating a relationship and sexuality programme for adults with intellectual disabilities in Donegal. *British Journal of Learning Disabilities* **37**, 327-329, doi:<https://doi.org/10.1111/j.1468-3156.2009.00591.x> (2009).

206 Garwood, M. & McCabe, M. P. Impact of Sex Education Programs on Sexual Knowledge and Feelings of Men with a Mild Intellectual Disability. *Education and Training in Mental Retardation and Developmental Disabilities* **35**, 269-283 (2000).

207 Penny, R. E. C. & Chataway, J. E. Sex Education for Mentally Retarded Persons. *Australia and New Zealand Journal of Developmental Disabilities* **8**, 204-212, doi:10.3109/13668258209018763 (1982).

208 Robinson, S. Effects of a Sex Education Program on Intellectually Handicapped Adults. *Australia and New Zealand Journal of Developmental Disabilities* **10**, 21-26, doi:10.3109/13668258409018663 (1984).

209 Lindsay, W. R., Bellshaw, E., Culross, G., Staines, C. & Michie, A. Increases in knowledge following a course of sex education for people with intellectual disabilities. *Journal of Intellectual Disability Research* **36**, 531-539, doi:<https://doi.org/10.1111/j.1365-2788.1992.tb00571.x> (1992).

210 Lindsay, W. R., Michie, A. M., Staines, C., Bellshaw, E. & Culross, G. Client Attitudes Towards Relationships: Changes Following a Sex Education Programme. *British Journal of Learning Disabilities* **22**, 70-73, doi:<https://doi.org/10.1111/j.1468-3156.1994.tb00119.x> (1994).

211 Caspar, L. A. & Glidden, L. M. Sexuality Education for Adults with Developmental Disabilities. *Education and Training in Mental Retardation and Developmental Disabilities* **36**, 172-177 (2001).

212 Foxx, R. M., McMorrow, M. J., Storey, K. & Rogers, B. M. Teaching social/sexual skills to mentally retarded adults. *Am J Ment Defic* **89**, 9-15 (1984).

213 Plaks, M. M. D. *et al.* Social-Sexual Education in Adolescents with Behavioral Neurogenetic Syndromes. *The Israel Journal of Psychiatry and Related Sciences* **47**, 28-24 (2010).

214 Haseltine, B. & Miltenberger, R. G. Teaching self-protection skills to persons with mental retardation. *Am J Ment Retard* **95**, 188-197 (1990).

215 Mueser, K. T., Valenti-Hein, D. & Yarnold, P. R. Dating-skills groups for the developmentally disabled: Social skills and problem-solving versus relaxation training. *Behavior Modification* **11**, 200-228, doi:10.1177/01454455870112005 (1987).

216 Box, M. & Shawe, J. The experiences of adults with learning disabilities attending a sexuality and relationship group: <em>“</em>I want to get married and have kids”. *Journal of Family Planning and Reproductive Health Care* **40**, 82-88, doi:10.1136/jfprhc-2012-100509 (2014).

217 Zylla, T. & Demetral, G. D. A behavioral approach to sex education. *Sexuality and Disability* **4**, 40-48, doi:10.1007/BF01102463 (1981).

218 McDermott, S., Kelly, M. & Spearman, J. Evaluation of a family planning program for individuals with mental retardation. *Sexuality and Disability* **12**, 307-317, doi:10.1007/BF02575322 (1994).

219 Wells, J., Clark, K. D. & Sarno, K. A computer-based interactive multimedia program to reduce HIV transmission for women with intellectual disability. *J Intellect Disabil Res* **56**, 371-381, doi:10.1111/j.1365-2788.2011.01482.x (2012).

220 McDermott, S., Martin, M., Weinrich, M. & Kelly, M. Program evaluation of a sex education curriculum for women with mental retardation. *Research in Developmental Disabilities* **20**, 93-106, doi:<https://doi.org/10.1016/S0891-4222(98)00035-3> (1999).

221 Dekker, L. P. *et al.* Improving Psychosexual Knowledge in Adolescents with Autism Spectrum Disorder: Pilot of the Tackling Teenage Training Program. *Journal of Autism and Developmental Disorders* **45**, 1532-1540, doi:10.1007/s10803-014-2301-9 (2015).

222 Lumley, V. A., Miltenberger, R. G., Long, E. S., Rapp, J. T. & Roberts, J. A. EVALUATION OF A SEXUAL ABUSE PREVENTION PROGRAM FOR ADULTS WITH MENTAL RETARDATION. *J Appl Behav Anal* **31**, 91-101, doi:<https://doi.org/10.1901/jaba.1998.31-91> (1998).

223 Lee, Y. K.-s. & Tang, C. S.-k. Evaluation of a sexual abuse prevention program for female Chinese adolescents with mild mental retardation. *American journal on mental retardation* **103**, 105-116 (1998).

224 Miltenberger, R. G. *et al.* TRAINING AND GENERALIZATION OF SEXUAL ABUSE PREVENTION SKILLS FOR WOMEN WITH MENTAL RETARDATION. *J Appl Behav Anal* **32**, 385-388, doi:<https://doi.org/10.1901/jaba.1999.32-385> (1999).

225 Egemo-Helm, K. R. *et al.* An evaluation of in situ training to teach sexual abuse prevention skills to women with mental retardation. *Behavioral Interventions* **22**, 99-119, doi:<https://doi.org/10.1002/bin.234> (2007).

226 Hickson, L., Khemka, I., Golden, H. & Chatzistyli, A. Randomized controlled trial to evaluate an abuse prevention curriculum for women and men with intellectual and developmental disabilities. *American journal on intellectual and developmental disabilities* **120**, 490-503 (2015).

227 Khemka, I. Increasing independent decision-making skills of women with mental retardation in simulated interpersonal situations of abuse. *American Journal on Mental Retardation* **105**, 387-401 (2000).

228 Khemka, I., Hickson, L. & Reynolds, G. Evaluation of a decision-making curriculum designed to empower women with mental retardation to resist abuse. *American Journal on Mental Retardation* **110**, 193-204 (2005).

229 Berkman, A. Reducing Sexual Risk Behaviors of Men With Severe Mental Illness. *Psychiatric Services* **57**, 417-417, doi:10.1176/appi.ps.57.3.417 (2006).

230 Linn, J. G. *et al.* Reaching impaired populations with HIV prevention programs: a clinical trial for homeless mentally ill African-American men. *Cell Mol Biol (Noisy-le-grand)* **49**, 1167-1175 (2003).

231 Susser, E. *et al.* Human Immunodeficiency Virus Sexual Risk Reduction in Homeless Men With Mental Illness. *Archives of General Psychiatry* **55**, 266-272, doi:10.1001/archpsyc.55.3.266 (1998).

232 Berkman, A. *et al.* HIV prevention with severely mentally ill men: A randomised controlled trial. *AIDS Care* **19**, 579-588, doi:10.1080/09540120701213989 (2007).

233 The National Institute of Mental Health Multisite HIV Prevention Trial Group. HIV prevention with persons with mental health problems. *Psychol Health Med* **11**, 142-154, doi:10.1080/13548500500445094

10.1080/13548500500445094. (2006).

234 Collins, P. Y. *et al.* Adding the Female Condom to HIV Prevention Interventions for Women with Severe Mental Illness: A Pilot Test. *Community Mental Health Journal* **47**, 143-155, doi:10.1007/s10597-010-9302-8 (2011).

235 Weinhardt, L. S., Carey, M. P., Carey, K. B. & Verdecias, R. N. Increasing assertiveness skills to reduce HIV risk among women living with a severe and persistent mental illness. *Journal of Consulting and Clinical Psychology* **66**, 680-684, doi:10.1037/0022-006X.66.4.680 (1998).

236 Carey, M. P. *et al.* Reducing HIV-Risk Behavior Among Adults Receiving Outpatient Psychiatric Treatment: Results From a Randomized Controlled Trial. *Journal of Consulting and Clinical Psychology* **72**, 252-268, doi:10.1037/0022-006X.72.2.252 (2004).

237 Kalichman, S. C., Sikkema, K. J., Kelly, J. A. & Bulto, M. Use of a brief behavioral skills intervention to prevent HIV infection among chronic mentally ill adults. *Psychiatr Serv* **46**, 275-280, doi:10.1176/ps.46.3.275 (1995).

238 Katz, R. C., Westerman, C., Beauchamp, K. & Clay, C. Effects of AIDS counseling and risk reduction training on the chronic mentally ill. *AIDS Education and Prevention* **8**, 457-463 (1996).

239 Kelly, J. A. *et al.* Reduction in risk behavior among adults with severe mental illness who learned to advocate for HIV prevention. *Psychiatr Serv* **48**, 1283-1288, doi:10.1176/ps.48.10.1283 (1997).

240 Malow, R. M. *et al.* Cognitive Behavioral HIV Risk Reduction in Those Receiving Psychiatric Treatment: A Clinical Trial. *AIDS and Behavior* **16**, 1192-1202, doi:10.1007/s10461-011-0104-y (2012).

241 Otto-Salaj, L. L., Kelly, J. A., Stevenson, L. Y., Hoffmann, R. & Kalichman, S. C. Outcomes of a randomized small-group HIV prevention intervention trial for people with serious mental illness. *Community Ment Health J* **37**, 123-144, doi:10.1023/a:1002709715201 (2001).

242 Tungpunkom, P. & Nicol, M. Life skills programmes for chronic mental illnesses. *Cochrane Database of Systematic Reviews*, doi:10.1002/14651858.CD000381.pub2 (2008).

243 Almerie, M. Q. *et al.* Social skills programmes for schizophrenia. *Cochrane Database of Systematic Reviews*, doi:10.1002/14651858.CD009006 (2011).

244 Almerie, M. Q. *et al.* Social skills programmes for schizophrenia. *Cochrane Database of Systematic Reviews*, doi:10.1002/14651858.CD009006.pub2 (2015).

245 Patterson, T. L. *et al.* Functional adaptation skills training (FAST): a pilot psychosocial intervention study in middle-aged and older patients with chronic psychotic disorders. *Am J Geriatr Psychiatry* **11**, 17-23 (2003).

246 Patterson, T. L. *et al.* Functional adaptation skills training (FAST): a randomized trial of a psychosocial intervention for middle-aged and older patients with chronic psychotic disorders. *Schizophr Res* **86**, 291-299, doi:10.1016/j.schres.2006.05.017 (2006).

247 Feldman, M. A., Case, L. & Sparks, B. Effectiveness of a child-care training program for parents at-risk for child neglect. *Canadian Journal of Behavioural Science / Revue canadienne des sciences du comportement* **24**, 14-28, doi:10.1037/h0078698 (1992).

248 Llewellyn, G., McConnell, D., Honey, A., Mayes, R. & Russo, D. Promoting health and home safety for children of parents with intellectual disability: a randomized controlled trial. *Res Dev Disabil* **24**, 405-431, doi:10.1016/j.ridd.2003.06.001 (2003).

249 Feldman, M. A. & Case, L. Teaching child-care and safety skills to parents with intellectual disabilities through self-learning. *Journal of Intellectual and Developmental Disability* **24**, 27-44 (1999).

250 Feldman, M. A., Ducharme, J. M. & Case, L. Using self-instructional pictorial manuals to teach child-care skills to mothers with intellectual disabilities. *Behav Modif* **23**, 480-497, doi:10.1177/0145445599233007 (1999).

251 Mildon, R., Wade, C. & Matthews, J. Considering the contextual fit of an intervention for families headed by parents with an intellectual disability: An exploratory study. *Journal of Applied Research in Intellectual Disabilities* **21**, 377-387 (2008).

252 Brisson, N. A. *Parent training and its effect on attunement of mothers with intellectual disabilities*. (Union Institute & University, 2009).

253 Keltner, B., Finn, D. & Shearer, D. Effects of family intervention on maternal-child interaction for mothers with developmental disabilities. *Family & Community Health: The Journal of Health Promotion & Maintenance* **17**, 35-49, doi:10.1097/00003727-199501000-00006 (1995).

254 Cihak, D. F., McMahon, D., Smith, C. C., Wright, R. & Gibbons, M. M. Teaching individuals with intellectual disability to email across multiple device platforms. *Research in developmental disabilities* **36C**, 645-656, doi:10.1016/j.ridd.2014.10.044 (2015).

255 McClimens, A. & Gordon, F. People with intellectual disabilities as bloggers: what's social capital got to do with it anyway? *J Intellect Disabil* **13**, 19-30, doi:10.1177/1744629509104486 (2009).

256 Shpigelman, C.-N. & Gill, C. J. How do adults with intellectual disabilities use Facebook? *Disability & Society* **29**, 1601-1616, doi:10.1080/09687599.2014.966186 (2014).

257 Davies, D. K. *et al.* An Interface to Support Independent Use of Facebook by People With Intellectual Disability. *Intellectual and Developmental Disabilities* **53**, 30-41, doi:10.1352/1934-9556-53.1.30 (2015).

258 Kelley, K. R., Test, D. W. & Cooke, N. L. Effects of picture prompts delivered by a video iPod on pedestrian navigation. *Exceptional Children* **79**, 459-474 (2013).

259 Mechling, L. & O'Brien, E. Computer-Based Video Instruction to Teach Students with Intellectual Disabilities to Use Public Bus Transportation. *Education and Training in Autism and Developmental Disabilities* **45**, 230-241 (2010).

260 Courbois, Y. *et al.* Wayfinding behaviour in Down syndrome: A study with virtual environments. *Research in Developmental Disabilities* **34**, 1825-1831, doi:<https://doi.org/10.1016/j.ridd.2013.02.023> (2013).

261 Mengue-Topio, H., Courbois, Y., Farran, E. K. & Sockeel, P. Route learning and shortcut performance in adults with intellectual disability: A study with virtual environments. *Research in Developmental Disabilities* **32**, 345-352, doi:<https://doi.org/10.1016/j.ridd.2010.10.014> (2011).

262 Purser, H. R. M. *et al.* The development of route learning in Down syndrome, Williams syndrome and typical development: investigations with virtual environments. *Developmental Science* **18**, 599-613, doi:10.1111/desc.12236 (2015).

263 McMahon, D., Cihak, D. F. & Wright, R. Augmented reality as a navigation tool to employment opportunities for postsecondary education students with intellectual disabilities and autism. *Journal of Research on Technology in Education* **47**, 157-172 (2015).

264 McMahon, D. D., Smith, C. C., Cihak, D. F., Wright, R. & Gibbons, M. M. Effects of Digital Navigation Aids on Adults With Intellectual Disabilities: Comparison of Paper Map, Google Maps, and Augmented Reality. *Journal of Special Education Technology* **30**, 157-165, doi:10.1177/0162643415618927 (2015).

265 Smith, C. C., Cihak, D. F., Kim, B., McMahon, D. D. & Wright, R. Examining augmented reality to improve navigation skills in postsecondary students with intellectual disability. *Journal of Special Education Technology* **32**, 3-11 (2017).

266 Gómez, J. & Ojala, T. in *Assistive Technologies for Physical and Cognitive Disabilities* 173-190 (IGI Global, 2015).

267 Davies, D. K., Stock, S. E., Holloway, S. & Wehmeyer, M. L. Evaluating a GPS-based transportation device to support independent bus travel by people with intellectual disability. *Intellectual and developmental disabilities* **48**, 454-463 (2010).

268 Stock, S. E., Davies, D. K., Wehmeyer, M. L. & Lachapelle, Y. Emerging new practices in technology to support independent community access for people with intellectual and cognitive disabilities. *NeuroRehabilitation* **28**, 261-269 (2011).

269 Mechling, L. C. & Seid, N. H. Use of a hand-held personal digital assistant (PDA) to self-prompt pedestrian travel by young adults with moderate intellectual disabilities. *Education and Training in Autism and Developmental Disabilities*, 220-237 (2011).

270 Bai, X. *et al.* Effectiveness of a life story work program on older adults with intellectual disabilities. *Clin Interv Aging* **9**, 1865-1872, doi:10.2147/cia.S56617 (2014).

271 Hamilton, C. & Atkinson, D. ‘A Story to Tell’: learning from the life-stories of older people with intellectual disabilities in Ireland. *British Journal of Learning Disabilities* **37**, 316-322, doi:<https://doi.org/10.1111/j.1468-3156.2009.00588.x> (2009).

272 Bigby, C. & Wiesel, I. Mediating community participation: Practice of support workers in initiating, facilitating or disrupting encounters between people with and without intellectual disability. *Journal of Applied Research in Intellectual Disabilities* **28**, 307-318 (2015).

273 Treece, A., Gregory, S., Ayres, B. & Mendis, K. 'I Always Do What They Tell Me To Do': Choice-making opportunities in the lives of two older persons with severe learning difficulties living in a community setting. *Disability & Society* **14**, 791-804, doi:10.1080/09687599925894 (1999).

274 Moffatt, S., Steer, M., Lawson, S., Penn, L. & O’Brien, N. Link Worker social prescribing to improve health and well-being for people with long-term conditions: qualitative study of service user perceptions. *BMJ Open* **7**, e015203, doi:10.1136/bmjopen-2016-015203 (2017).

275 Brandling, J., House, W., Howitt, D. & Sansom, A. ‘New Routes’: Pilot Research Project of a New Social Prescribing Service Provided in Keynsham. 76 (2011).

276 Carnes, D. *et al.* The impact of a social prescribing service on patients in primary care: a mixed methods evaluation. *BMC Health Serv Res* **17**, 835, doi:10.1186/s12913-017-2778-y (2017).

277 Dayson, C., Bashir, N., Bennett, E. & Sanderson, E. The Rotherham Social Prescribing Service for People with Long-Term Health Conditions: Annual Evaluation Report. 67 (2016).

278 Farenden, C., Mitchell, C., Feast, S. & Verdenicci, S. Community Navigation in Brighton & Hove. Evaluation of a Social Prescribing Pilot. 68 (2015).

279 Vogelpoel, N. & Jarrold, K. Social prescription and the role of participatory arts programmes for older people with sensory impairments. *Journal of Integrated Care* **22**, 39-50, doi:10.1108/JICA-01-2014-0002 (2014).

280 Kimberlee, R., Ward, R., Jones, M. & Powell, J. Measuring the Economic Impact of Wellspring Healthy Living Centre’s Social Prescribing Wellbeing Programme for Low Level Mental Health Issues Encountered by GP Services. 111 (2014).

281 Wigfield, A., Kispeter, E., Alden, S. & Turner, R. Age UK’s Fit for the Future Project: Evaluation Report. (2015).

282 Friedlie, L., Themessl-huber, M. & Butchart, M. Evaluation of Dundee Equally Well Sources of Support: Social Prescribing in Maryfield. 42 (2012).

283 The Health, F. Shine 2014 Final Report Social Prescribing: Integrating GP and Community Assets for Health. 33 (2015).

284 ERS Research Consultancy. Newcastle Social Prescribing Project. Final Report. 1–78 (2013).

285 Baines, A. Rugby Social Prescribing Project ConnectWELL. 45 (2015).

286 Age UK & Age Concern. Social Prescribing. A Model for Partnership Working Between Primary Care and the Voluntary Sector. (2012).

287 Grayer, J., Cape, J., Orpwood, L., Leibowitz, J. & Buszewicz, M. Facilitating access to voluntary and community services for patients with psychosocial problems: a before-after evaluation. *BMC Fam Pract* **9**, 27, doi:10.1186/1471-2296-9-27 (2008).

288 Loftus, A. M., McCauley, F. & McCarron, M. O. Impact of social prescribing on general practice workload and polypharmacy. *Public Health* **148**, 96-101, doi:10.1016/j.puhe.2017.03.010 (2017).

289 Grant, C., Goodenough, T., Harvey, I. & Hine, C. A randomised controlled trial and economic evaluation of a referrals facilitator between primary care and the voluntary sector. *Bmj* **320**, 419-423, doi:10.1136/bmj.320.7232.419 (2000).

290 Webber, M. *et al.* Effect of the Connecting People Intervention on Social Capital: A Pilot Study. *Research on Social Work Practice* **29**, 483-494, doi:10.1177/1049731517753685 (2018).

291 Barbato, A. *et al.* Outcome of community-based rehabilitation program for people with mental illness who are considered difficult to treat. *J Rehabil Res Dev* **44**, 775-783, doi:10.1682/jrrd.2007.02.0041 (2007).

292 Terzian, E. *et al.* Social network intervention in patients with schizophrenia and marked social withdrawal: a randomized controlled study. *Can J Psychiatry* **58**, 622-631, doi:10.1177/070674371305801108 (2013).

293 Middelboe, T. Prospective study of clinical and social outcome of stay in small group homes for people with mental illness. *Br J Psychiatry* **171**, 251-255, doi:10.1192/bjp.171.3.251 (1997).

294 Sheridan, A. J. *et al.* Improving social functioning and reducing social isolation and loneliness among people with enduring mental illness: Report of a randomised controlled trial of supported socialisation. *Int J Soc Psychiatry* **61**, 241-250, doi:10.1177/0020764014540150 (2015).

295 Heslop, P. Good practice in befriending services for people with learning difficulties. *British Journal of Learning Disabilities* **33**, 27-33, doi:<https://doi.org/10.1111/j.1468-3156.2004.00310.x> (2005).

296 Hughes, A. & Waldenm, S. Befriending: a note of caution. *British Journal of Learning Disabilities* **27**, 88-92 (1999).

297 Fokkema, C. M. & van Tilburg, T. G. [Loneliness interventions among older adults: sense or nonsense?]. *Tijdschr Gerontol Geriatr* **38**, 185-203 (2007).

298 Bøen, H., Dalgard, O. S., Johansen, R. & Nord, E. A randomized controlled trial of a senior centre group programme for increasing social support and preventing depression in elderly people living at home in Norway. *BMC Geriatr* **12**, 20, doi:10.1186/1471-2318-12-20 (2012).

299 Rivera, J. J., Sullivan, A. M. & Valenti, S. S. Adding consumer-providers to intensive case management: does it improve outcome? *Psychiatr Serv* **58**, 802-809, doi:10.1176/ps.2007.58.6.802 (2007).

300 Solomon, P. & Draine, J. One-year outcomes of a randomized trial of consumer case management. *Evaluation and Program Planning* **18**, 117-127, doi:<https://doi.org/10.1016/0149-7189(95)00003-T> (1995).

301 Solomon, P. & Draine, J. The efficacy of a consumer case management team: 2-year outcomes of a randomized trial. *J Ment Health Adm* **22**, 135-146, doi:10.1007/bf02518754 (1995).

302 Harrop, C., Ellett, L., Brand, R. & Lobban, F. Friends interventions in psychosis: a narrative review and call to action. *Early Interv Psychiatry* **9**, 269-278, doi:10.1111/eip.12172 (2015).

303 Jantz, K. M. Support groups for adults with asperger syndrome. *Focus on Autism and Other Developmental Disabilities* **26**, 119-128, doi:10.1177/1088357611406903 (2011).

304 Wilson, N. J., Jaques, H., Johnson, A. & Brotherton, M. L. From Social Exclusion to Supported Inclusion: Adults with Intellectual Disability Discuss Their Lived Experiences of a Structured Social Group. *Journal of Applied Research in Intellectual Disabilities* **30**, 847-858, doi:<https://doi.org/10.1111/jar.12275> (2017).

305 Kaplan, K., Salzer, M. S., Solomon, P., Brusilovskiy, E. & Cousounis, P. Internet peer support for individuals with psychiatric disabilities: A randomized controlled trial. *Soc Sci Med* **72**, 54-62, doi:10.1016/j.socscimed.2010.09.037 (2011).

306 Kaplan, K., Solomon, P., Salzer, M. S. & Brusilovskiy, E. Assessing an Internet-based parenting intervention for mothers with a serious mental illness: A randomized controlled trial. *Psychiatric Rehabilitation Journal* **37**, 222-231, doi:10.1037/prj0000080 (2014).

307 Proudfoot, J. *et al.* Effects of adjunctive peer support on perceptions of illness control and understanding in an online psychoeducation program for bipolar disorder: a randomised controlled trial. *J Affect Disord* **142**, 98-105, doi:10.1016/j.jad.2012.04.007 (2012).

308 Alvarez-Jimenez, M. *et al.* On the HORYZON: Moderated online social therapy for long-term recovery in first episode psychosis. *Schizophrenia Research* **143**, 143-149, doi:<https://doi.org/10.1016/j.schres.2012.10.009> (2013).

309 Gleeson, J. F. *et al.* Safety and privacy outcomes from a moderated online social therapy for young people with first-episode psychosis. *Psychiatr Serv* **65**, 546-550, doi:10.1176/appi.ps.201300078 (2014).

310 Castelein, S. *et al.* The effectiveness of peer support groups in psychosis: a randomized controlled trial. *Acta Psychiatr Scand* **118**, 64-72, doi:10.1111/j.1600-0447.2008.01216.x (2008).

311 Gammonley, D. & Luken, K. Peer education and advocacy through recreation and leadership. *Psychiatr Rehabil J* **25**, 170-178, doi:10.1037/h0095028 (2001).

312 Rosen, C. E. & Rosen, S. Evaluating an intervention program for the elderly. *Community Ment Health J* **18**, 21-33, doi:10.1007/bf00757109 (1982).

313 Schneider, C. & Hattie, B. Exploring the social lives of young adults with disabilities. *Alter* **10**, 236-247, doi:<https://doi.org/10.1016/j.alter.2016.02.003> (2016).

314 Retherford, K. S. & Schreiber, L. R. Camp Campus: College Preparation for Adolescents and Young Adults With High-Functioning Autism, Asperger Syndrome, and Other Social Communication Disorders. *Topics in Language Disorders* **35** (2015).

315 Ness, B. M. Supporting Self-Regulated Learning for College Students with Asperger Syndrome: Exploring the “Strategies for College Learning” Model. *Mentoring & Tutoring: Partnership in Learning* **21**, 356-377, doi:10.1080/13611267.2013.855865 (2013).

316 Ashburner, J. K., Bobir, N. I. & van Dooren, K. Evaluation of an Innovative Interest-Based Post-School Transition Programme for Young People with Autism Spectrum Disorder. *International Journal of Disability, Development and Education* **65**, 262-285, doi:10.1080/1034912X.2017.1403012 (2018).

317 Hotez, E. *et al.* Designing a Summer Transition Program for Incoming and Current College Students on the Autism Spectrum: A Participatory Approach. *Front Psychol* **9**, 46, doi:10.3389/fpsyg.2018.00046 (2018).

318 Curtin, C. *et al.* Expanding Horizons: A Pilot Mentoring Program Linking College/Graduate Students and Teens With ASD. *Clinical Pediatrics* **55**, 150-156, doi:10.1177/0009922815588821 (2015).

319 Gillespie-Lynch, K. *et al.* "For a Long Time Our Voices have been Hushed": Using Student Perspectives to Develop Supports for Neurodiverse College Students. *Frontiers in psychology* **8**, 544-544, doi:10.3389/fpsyg.2017.00544 (2017).

320 Ames, M. E., McMorris, C. A., Alli, L. N. & Bebko, J. M. Overview and Evaluation of a Mentorship Program for University Students With ASD. *Focus on Autism and Other Developmental Disabilities* **31**, 27-36, doi:10.1177/1088357615583465 (2015).

321 Hamilton, J., Stevens, G. & Girdler, S. Becoming a Mentor: The Impact of Training and the Experience of Mentoring University Students on the Autism Spectrum. *PLOS ONE* **11**, e0153204, doi:10.1371/journal.pone.0153204 (2016).

322 Roberts, N. & Birmingham, E. Mentoring University Students with ASD: A Mentee-centered Approach. *Journal of Autism and Developmental Disorders* **47**, 1038-1050, doi:10.1007/s10803-016-2997-9 (2017).

323 Siew, C. T., Mazzucchelli, T. G., Rooney, R. & Girdler, S. A specialist peer mentoring program for university students on the autism spectrum: A pilot study. *PLOS ONE* **12**, e0180854, doi:10.1371/journal.pone.0180854 (2017).

324 Martin, N. *et al.* Does “mentoring” offer effective support to autistic adults? A mixed-methods pilot study. *Advances in Autism* **3**, 229-239, doi:10.1108/AIA-06-2017-0013 (2017).

325 Bigby, C., Wilson, N. J., Balandin, S. & Stancliffe, R. J. Disconnected expectations: Staff, family, and supported employee perspectives about retirement. *Journal of Intellectual & Developmental Disability* **36**, 167-174, doi:10.3109/13668250.2011.598852 (2011).

326 Craig, D. & Bigby, C. “She's been involved in everything as far as I can see”: Supporting the active participation of people with intellectual disability in community groups. *Journal of Intellectual and Developmental Disability* **40**, 12-25 (2015).

327 Farrell, C. & Bryant, W. Voluntary Work for Adults with Mental Health Problems: An Exploration of the Perspectives of Recruiters. *British Journal of Occupational Therapy* **72**, 188-196, doi:10.1177/030802260907200502 (2009).

328 Harlan-Simmons, J. E., Holtz, P., Todd, J. & Mooney, M. F. Building social relationships through valued roles: Three older adults and the community membership project. *Mental retardation* **39**, 171-180 (2001).

329 Snethen, G., McCormick, B. P. & Van Puymbroeck, M. Community involvement, planning and coping skills: pilot outcomes of a recreational-therapy intervention for adults with schizophrenia. *Disabil Rehabil* **34**, 1575-1584, doi:10.3109/09638288.2011.650315 (2012).

330 Saxby, H., Thomas, M., Felce, D. & de Kock, U. The use of shops, cafes and public houses by severely and profoundly mentally retarded adults. *British Journal of Mental Subnormality* **32**, 82-92 (1986).

331 Ouellette, L., Horner, R. H. & Stephen Newton, J. Changing activity patterns to improve social networks: A descriptive analysis. *Behavioral Interventions* **9**, 55-66 (1994).

332 Wilson, N. J. *et al.* Mentors' experiences of using the Active Mentoring model to support older adults with intellectual disability to participate in community groups. *Journal of Intellectual and Developmental Disability* **38**, 344-355 (2013).

333 Bigby, C. *et al.* An Effective Program Design to Support Older Workers With Intellectual Disability to Participate Individually in Community Groups. *Journal of Policy and Practice in Intellectual Disabilities* **11**, 117-127, doi:<https://doi.org/10.1111/jppi.12080> (2014).

334 Stancliffe, R. J., Bigby, C., Balandin, S., Wilson, N. J. & Craig, D. Transition to retirement and participation in mainstream community groups using active mentoring: A feasibility and outcomes evaluation with a matched comparison group. *Journal of Intellectual Disability Research* **59**, 703-718 (2015).

335 Wilson, N. J. *et al.* A case study about the supported participation of older men with lifelong disability at Australian community-based Men's Sheds. *Journal of Intellectual and Developmental Disability* **40**, 330-341 (2015).

336 Temple, V. A. & Walkley, J. W. Perspectives of constraining and enabling factors for health‐promoting physical activity by adults with intellectual disability. *Journal of Intellectual and Developmental Disability* **32**, 28-38 (2007).

337 Duraiswamy, G., Thirthalli, J., Nagendra, H. R. & Gangadhar, B. N. Yoga therapy as an add-on treatment in the management of patients with schizophrenia – a randomized controlled trial. *Acta Psychiatrica Scandinavica* **116**, 226-232, doi:<https://doi.org/10.1111/j.1600-0447.2007.01032.x> (2007).

338 Behere, R. V. *et al.* Effect of yoga therapy on facial emotion recognition deficits, symptoms and functioning in patients with schizophrenia. *Acta Psychiatrica Scandinavica* **123**, 147-153, doi:<https://doi.org/10.1111/j.1600-0447.2010.01605.x> (2011).

339 Varambally, S. *et al.* Therapeutic efficacy of add-on yogasana intervention in stabilized outpatient schizophrenia: Randomized controlled comparison with exercise and waitlist. *Indian Journal of Psychiatry* **54**, 227-232, doi:10.4103/0019-5545.102414 (2012).

340 Manjunath, R. B., Varambally, S., Thirthalli, J., Basavaraddi, I. V. & Gangadhar, B. N. Efficacy of yoga as an add-on treatment for in-patients with functional psychotic disorder. *Indian journal of psychiatry* **55**, S374 (2013).

341 Battaglia, G. *et al.* Soccer practice as an add-on treatment in the management of individuals with a diagnosis of schizophrenia. *Neuropsychiatr Dis Treat* **9**, 595-603, doi:10.2147/ndt.S44066 (2013).

342 Acil, A. A., Dogan, S. & Dogan, O. The effects of physical exercises to mental state and quality of life in patients with schizophrenia. *Journal of Psychiatric and Mental Health Nursing* **15**, 808-815, doi:<https://doi.org/10.1111/j.1365-2850.2008.01317.x> (2008).

343 Abdel-Baki, A., Brazzini-Poisson, V., Marois, F., Letendre, É. & Karelis, A. D. Effects of aerobic interval training on metabolic complications and cardiorespiratory fitness in young adults with psychotic disorders: A pilot study. *Schizophrenia Research* **149**, 112-115, doi:<https://doi.org/10.1016/j.schres.2013.06.040> (2013).

344 Heggelund, J., Nilsberg, G. E., Hoff, J., Morken, G. & Helgerud, J. Effects of high aerobic intensity training in patients with schizophrenia—A controlled trial. *Nordic Journal of Psychiatry* **65**, 269-275, doi:10.3109/08039488.2011.560278 (2011).

345 Marzolini, S., Jensen, B. & Melville, P. Feasibility and effects of a group-based resistance and aerobic exercise program for individuals with severe schizophrenia: A multidisciplinary approach. *Mental Health and Physical Activity* **2**, 29-36, doi:<https://doi.org/10.1016/j.mhpa.2008.11.001> (2009).

346 Scheewe, T. W., Takken, T. I. M., Kahn, R. S., Cahn, W. & Backx, F. J. G. Effects of Exercise Therapy on Cardiorespiratory Fitness in Patients with Schizophrenia. *Medicine & Science in Sports & Exercise* **44**, 1834-1842, doi:10.1249/MSS.0b013e318258e120 (2012).

347 Scheewe, T. W. *et al.* Exercise therapy improves mental and physical health in schizophrenia: a randomised controlled trial. *Acta Psychiatrica Scandinavica* **127**, 464-473, doi:10.1111/acps.12029 (2013).

348 Scheewe, T. W. *et al.* Exercise therapy, cardiorespiratory fitness and their effect on brain volumes: A randomised controlled trial in patients with schizophrenia and healthy controls. *European Neuropsychopharmacology* **23**, 675-685, doi:10.1016/j.euroneuro.2012.08.008 (2013).

349 Heggelund, J., Morken, G., Helgerud, J., Nilsberg, G. E. & Hoff, J. Therapeutic effects of maximal strength training on walking efficiency in patients with schizophrenia – a pilot study. *BMC Research Notes* **5**, 344, doi:10.1186/1756-0500-5-344 (2012).

350 van Schijndel-Speet, M., Evenhuis, H. M., van Wijck, R., van Empelen, P. & Echteld, M. A. Facilitators and barriers to physical activity as perceived by older adults with intellectual disability. *Intellect Dev Disabil* **52**, 175-186, doi:10.1352/1934-9556-52.3.175 (2014).

351 van Schijndel-Speet, M., Evenhuis, H. M., van Wijck, R., van Montfort, K. C. & Echteld, M. A. A structured physical activity and fitness programme for older adults with intellectual disabilities: results of a cluster-randomised clinical trial. *J Intellect Disabil Res* **61**, 16-29, doi:10.1111/jir.12267 (2017).

352 Lante, K. A., Walkley, J. W., Gamble, M. & Vassos, M. V. An initial evaluation of a long-term, sustainable, integrated community-based physical activity program for adults with intellectual disability. *Journal of Intellectual and Developmental Disability* **36**, 197-206 (2011).

353 Matthews, L. *et al.* Process evaluation of the Walk Well study: a cluster-randomised controlled trial of a community based walking programme for adults with intellectual disabilities. *BMC public health* **16**, 1-11 (2016).

354 Melville, C. A. *et al.* Effectiveness of a walking programme to support adults with intellectual disabilities to increase physical activity: walk well cluster-randomised controlled trial. *International Journal of Behavioral Nutrition and Physical Activity* **12**, 1-11 (2015).

355 Bota, A., Teodorescu, S. & Şerbănoiu, S. Unified Sports – A Social Inclusion Factor in School Communities for Young People with Intellectual Disabilities. *Procedia - Social and Behavioral Sciences* **117**, 21-26, doi:<https://doi.org/10.1016/j.sbspro.2014.02.172> (2014).

356 Harada, C. M., Siperstein, G. N., Parker, R. C. & Lenox, D. Promoting social inclusion for people with intellectual disabilities through sport: Special Olympics International, global sport initiatives and strategies. *Sport in Society* **14**, 1131– 1148 (2011).

357 Riggen, K. & Ulrich, D. The effects of sport participation on individuals with mental retardation. *Adapted Physical Activity Quarterly* **10**, 42-51 (1993).

358 Rosegard, E., Pegg, S. & Compton, D. M. Effect of Unified Sport on Maladaptive Behaviors Among Special Olympics Athletes. *World Leisure Journal* **43**, 39-48, doi:10.1080/04419057.2001.9674229 (2001).

359 McConkey, R., Dowling, S., Hassan, D. & Menke, S. Promoting social inclusion through unified sports for youth with intellectual disabilities: a five‐nation study. *Journal of intellectual disability research* **57**, 923-935 (2013).

360 Wilski, M., Nadolska, A., Dowling, S., Mcconkey, R. & Hassan, D. Personal development of participants in Special Olympics unified sports teams. *Human Movement* **13**, 271-279 (2012).

361 Hassan, D., Dowling, S., McConkey, R. & Menke, S. The inclusion of people with intellectual disabilities in team sports: Lessons from the Youth Unified Sports programme of Special Olympics. *Sport in Society* **15**, 1275-1290 (2012).

362 Carmeli, E., Kessel, S., Coleman, R. & Ayalon, M. Effects of a Treadmill Walking Program on Muscle Strength and Balance in Elderly People With Down Syndrome. *The Journals of Gerontology: Series A* **57**, M106-M110, doi:10.1093/gerona/57.2.M106 (2002).

363 Carmeli, E., Merrick, J. & Berner, Y. N. Effect of training on health and functional status in older adults with intellectual disability. *International Journal of Therapy and Rehabilitation* **11**, 481-485, doi:10.12968/ijtr.2004.11.10.17189 (2004).

364 Carmeli, E., Zinger-Vaknin, T., Morad, M. & Merrick, J. Can physical training have an effect on well-being in adults with mild intellectual disability? *Mech Ageing Dev* **126**, 299-304, doi:10.1016/j.mad.2004.08.021 (2005).

365 Carmeli, E., Orbach, I., Zinger‐Vaknin, T., Morad, M. & Merrick, J. Physical training and well‐being in older adults with mild intellectual disability: A residential care study. *Journal of Applied Research in Intellectual Disabilities* **21**, 457-465 (2008).

366 Tomporowski, P. D. & Ellis, N. R. Effects of exercise on the physical fitness, intelligence, and adaptive behavior of institutionalized mentally retarded adults. *Applied Research in Mental Retardation* **5**, 329-337 (1984).

367 Tomporowski, P. D. & Ellis, N. R. The effects of exercise on the health, intelligence, and adaptive behavior of institutionalized severely and profoundly mentally retarded adults: a systematic replication. *Applied Research in Mental Retardation* **6**, 465-473 (1985).

368 Pérez-Cruzado, D. & Cuesta-Vargas, A. I. Changes on quality of life, self-efficacy and social support for activities and physical fitness in people with intellectual disabilities through multimodal intervention. *European Journal of Special Needs Education* **31**, 553-564 (2016).

369 Heller, T., Hsieh, K. & Rimmer, J. H. Attitudinal and psychosocial outcomes of a fitness and health education program on adults with Down syndrome. *American Journal on Mental Retardation* **109**, 175-185 (2004).

370 Marks, B., Sisirak, J. & Chang, Y. C. Efficacy of the HealthMatters program train‐the‐trainer model. *Journal of Applied Research in Intellectual Disabilities* **26**, 319-334 (2013).

371 Elliott, R. O., Jr., Dobbin, A. R., Rose, G. D. & Soper, H. V. Vigorous, aerobic exercise versus general motor training activities: effects on maladaptive and stereotypic behaviors of adults with both autism and mental retardation. *J Autism Dev Disord* **24**, 565-576, doi:10.1007/bf02172138 (1994).

372 García-Villamisar, D. A. & Dattilo, J. Effects of a leisure programme on quality of life and stress of individuals with ASD. *J Intellect Disabil Res* **54**, 611-619, doi:10.1111/j.1365-2788.2010.01289.x (2010).

373 Palmen, A., Didden, R. & Korzilius, H. An outpatient group training programme for improving leisure lifestyle in high-functioning young adults with ASD: A pilot study. *Developmental Neurorehabilitation* **14**, 297-309 (2011).

374 Lynnes, M. D., Nichols, D. & Temple, V. A. Fostering independence in health-promoting exercise. *Journal of Intellectual Disabilities* **13**, 143-159 (2009).

375 Tedrick, T. Growing older in Special Olympics: Meaning and benefits of participation—Selected case studies. *Activities, Adaptation & Aging* **33**, 137-160 (2009).

376 Glidden, L. M., Bamberger, K. T., Draheim, A. R. & Kersh, J. Parent and athlete perceptions of special olympics participation: utility and danger of proxy responding. *Intellect Dev Disabil* **49**, 37-45, doi:10.1352/1934-9556-49.1.37 (2011).

377 Válková, H. The differences in behaviour indices of participants and non-participants in Special Olympics movement. *Acta Universitatis Palackianae Olomucensis Gymnica* **26**, 39–46 (1996).

378 Válková, H. The development of indices of motor competence and social behavior of participants and non-participants in the Special Olympics movement. *Acta Universitatis Palackianae Olomucensis Gymnica* **28**, 53-59 (1998).

379 Werner, S. Athletes’, parents’, and siblings’ experiences from the Special Olympics World Games. *Journal of Intellectual & Developmental Disability* **40**, 167-178, doi:10.3109/13668250.2015.1010148 (2015).

380 Marks, B., Sisirak, J., Heller, T. & Wagner, M. Evaluation of community‐based health promotion programs for Special Olympics athletes. *Journal of Policy and Practice in Intellectual Disabilities* **7**, 119-129 (2010).

381 Farrell, R. J., Crocker, P. R. E., McDonough, M. H. & Sedgwick, W. A. The driving force: motivation in special Olympians. *Adapted physical activity quarterly* **21**, 153-166 (2004).

382 Ghasemia, A., Atashib, V. & Momenic, M. Special Olympics and down syndrome: the investigate of self-efficacy and interpersonal dependency. *Advances in Environmental Biology*, 2439-2446 (2012).

383 Goodwin, D., L. , Fitzpatrick, D., A., Thurmeier, R. & Hall, C. The Decision to Join Special Olympics: Parents? Perspectives. *Adapted Physical Activity Quarterly* **23**, 163-183, doi:10.1123/apaq.23.2.163 (2006).

384 Harada, C. M. & Siperstein, G. N. The sport experience of athletes with intellectual disabilities: A national survey of Special Olympics athletes and their families. *Adapted Physical Activity Quarterly* **26**, 68-85 (2009).

385 Weiss, J., Diamond, T., Demark, J. & Lovald, B. Involvement in Special Olympics and its relations to self-concept and actual competency in participants with developmental disabilities. *Res Dev Disabil* **24**, 281-305, doi:10.1016/s0891-4222(03)00043-x (2003).

386 Weiss, J. & Bebko, J. M. Participation in Special Olympics and change in athlete self-concept over 42 months. (2008).

387 Wilhite, B. & Kleiber, D. A. The effect of Special Olympics participation on community integration. *Therapeutic Recreation Journal* **26**, 9-20 (1992).

388 Shapiro, D. R. Participation motives of Special Olympics athletes. *Adapted Physical Activity Quarterly* **20**, 150-165 (2003).

389 Dykens, E. M. & Cohen, D. J. Effects of Special Olympics International on social competence in persons with mental retardation. *Journal of the American Academy of Child & Adolescent Psychiatry* **35**, 223-229 (1996).

390 Frey, G. C., Buchanan, A. M. & Rosser Sandt, D. D. “I'd rather watch TV”: An examination of physical activity in adults with mental retardation. *Mental retardation* **43**, 241-254 (2005).

391 Bould, E., Bigby, C., Bennett, P. C. & Howell, T. J. ‘More people talk to you when you have a dog’ – dogs as catalysts for social inclusion of people with intellectual disabilities. *Journal of Intellectual Disability Research* **62**, 833-841, doi:<https://doi.org/10.1111/jir.12538> (2018).

392 Jessen, J., Cardiello, F. & Baun, M. M. Avian companionship in alleviation of depression, loneliness, and low morale of older adults in skilled rehabilitation units. *Psychological reports* **78**, 339-348 (1996).

393 Chu, C.-I., Liu, C.-Y., Sun, C.-T. & Lin, J. The effect of animal-assisted activity on inpatients with schizophrenia. *Journal of psychosocial nursing and mental health services* **47**, 42-48 (2009).

394 Barak, Y., Savorai, O., Mavashev, S. & Beni, A. Animal-assisted therapy for elderly schizophrenic patients: a one-year controlled trial. *The American journal of geriatric psychiatry* **9**, 439-442 (2001).

395 Villalta-Gil, V. *et al.* Dog-Assisted Therapy in the Treatment of Chronic Schizophrenia Inpatients. *Anthrozoös* **22**, 149-159, doi:10.2752/175303709X434176 (2009).

396 Marr, C. A. *et al.* Animal-assisted therapy in psychiatric rehabilitation. *Anthrozoös* **13**, 43-47 (2000).

397 Pedersen, I., Martinsen, E. W., Berget, B. & Braastad, B. O. Farm animal-assisted intervention for people with clinical depression: A randomized controlled trial. *Anthrozoös* **25**, 149-160 (2012).

398 Berget, B., Ekeberg, O. & Braastad, B. O. Animal-assisted therapy with farm animals for persons with psychiatric disorders: effects on self-efficacy, coping ability and quality of life, a randomized controlled trial. *Clin Pract Epidemiol Ment Health* **4**, 9-9, doi:10.1186/1745-0179-4-9 (2008).

399 Berget, B., Ekeberg, Ø., Pedersen, I. & Braastad, B. O. Animal-Assisted Therapy with Farm Animals for Persons with Psychiatric Disorders: Effects on Anxiety and Depression, a Randomized Controlled Trial. *Occupational Therapy in Mental Health* **27**, 50-64, doi:10.1080/0164212X.2011.543641 (2011).

400 Antonioli, C. & Reveley, M. A. Randomised controlled trial of animal facilitated therapy with dolphins in the treatment of depression. *Bmj* **331**, 1231 (2005).

401 Darragh, J. A., Ellison, C. J., Rillotta, F., Bellon, M. & Crocker, R. Exploring the impact of an arts-based, day options program for young adults with intellectual disabilities. *Research and Practice in Intellectual and Developmental Disabilities* **3**, 22-31 (2016).

402 Allan, J., Barford, H., Horwood, F., Stevens, J. & Tanti, G. ATIC: Developing a recovery-based art therapy practice. *International Journal of Art Therapy* **20**, 14-27, doi:10.1080/17454832.2014.968597 (2015).

403 Chiu, G., Hancock, J. & Waddell, A. Expressive Arts Therapy Group Helps Improve Mood State in an Acute Care Psychiatric Setting (Une thérapie de groupe ouverte en studio basée sur les arts de la scène améliore l'humeur des patients en psychiatrie dans un établissement de soins intensifs). *Canadian Art Therapy Association Journal* **28**, 34-42, doi:10.1080/08322473.2015.1100577 (2015).

404 Bungay, H. & Clift, S. Arts on prescription: a review of practice in the U.K. *Perspect Public Health* **130**, 277-281, doi:10.1177/1757913910384050 (2010).

405 Hacking, S., Secker, J., Spandler, H., Kent, L. & Shenton, J. Evaluating the impact of participatory art projects for people with mental health needs. *Health Soc Care Community* **16**, 638-648, doi:10.1111/j.1365-2524.2008.00789.x (2008).

406 Stokrocki, M., Sutton Andrews, S. & Saemundsdottir, S. The Role of Art for Homeless Women and Survivors of Domestic Violence. *Visual arts research* **30**, 73-82 (2004).

407 Howells, V. & Zelnik, T. Making art: A qualitative study of personal and group transformation in a community arts studio. *Psychiatric Rehabilitation Journal* **32**, 215-222, doi:10.2975/32.3.2009.215.222 (2009).

408 Thompson, G. *Aesthetic Action and Self-Construction of an Artist Identity: The Impact of Art and Art Therapy on Subjectivity and Mental Illness in Qualitative Research*, Saybrook University, (2016).

409 Griffith, F. J., Seymour, L. & Goldberg, M. Reframing art therapy to meet psychosocial and financial needs in homelessness. *The Arts in Psychotherapy* **46**, 33-40, doi:<https://doi.org/10.1016/j.aip.2015.09.007> (2015).

410 Spandler, H., Secker, J., Kent, L., Hacking, S. & Shenton, J. Catching life: the contribution of arts initiatives to recovery approaches in mental health. *J Psychiatr Ment Health Nurs* **14**, 791-799, doi:10.1111/j.1365-2850.2007.01174.x (2007).

411 Hildebrandt, M. K., Koch, S. C. & Fuchs, T. "We Dance and Find Each Other"1: Effects of Dance/Movement Therapy on Negative Symptoms in Autism Spectrum Disorder. *Behav Sci (Basel)* **6**, 24, doi:10.3390/bs6040024 (2016).

412 Koch, S. C., Mehl, L., Sobanski, E., Sieber, M. & Fuchs, T. Fixing the mirrors: A feasibility study of the effects of dance movement therapy on young adults with autism spectrum disorder. *Autism* **19**, 338-350 (2015).

413 Koehne, S., Behrends, A., Fairhurst, M. T. & Dziobek, I. Fostering social cognition through an imitation-and synchronization-based dance/movement intervention in adults with autism spectrum disorder: A controlled proof-of-concept study. *Psychotherapy and psychosomatics* **85**, 27-35 (2016).

414 Mastrominico, A. *et al.* Effects of Dance Movement Therapy on Adult Patients with Autism Spectrum Disorder: A Randomized Controlled Trial. *Behav Sci (Basel)* **8**, doi:10.3390/bs8070061 (2018).

415 Mateos-Moreno, D. & Atencia-Doña, L. Effect of a combined dance/movement and music therapy on young adults diagnosed with severe autism. *Arts in Psychotherapy* **40**, 465-472 (2013).

416 Sandel, S. L. The process of individuation in dance-movement therapy with schizophrenic patients. *The Arts in Psychotherapy* **9**, 11-18, doi:10.1016/0197-4556(82)90022-3 (1982).

417 Stickley, T., Crosbie, B. & Hui, A. The Stage Life: promoting the inclusion of young people through participatory arts. *British Journal of Learning Disabilities* **40**, 251-258, doi:<https://doi.org/10.1111/j.1468-3156.2011.00703.x> (2012).

418 Hackett, S. & Bourne, J. The Get Going Group: Dramatherapy with adults who have learning disabilities and mental health difficulties. *Dramatherapy* **36**, 43-50 (2014).

419 Foloştină, R., Tudorache, L., Michel, T., Erzsébet, B. & Duţă, N. Using drama therapy and storytelling in developing social competences in adults with intellectual disabilities of residential centers. *Procedia-Social and Behavioral Sciences* **186**, 1268-1274 (2015).

420 Gardner-Hynd, N. in *Drama as Therapy Volume 2* 192-208 (Routledge, 2010).

421 Grainger, R. Dramatherapy and thought-disorder. *Dramatherapy: Theory and Practice 2* **3**, 164 (1992).

422 Jaaniste, J. A New Beginning–A Dramatherapy Group for Participants with Co-Occurring Mental Illness and Substance Abuse in a Mental Health Setting. *Dramatherapy* **30**, 17-22 (2008).

423 Jacques, J.-F. The impact of a theatre performance on mental health service delivery in the context of user involvement. *Dramatherapy* **33**, 87-100 (2011).

424 Lahad, M. The use of drama therapy with crisis intervention groups, following mass evacuation. *The Arts in psychotherapy* **26**, 27-33 (1999).

425 McAlister, M. From transitional object to symbol: Spiderman in a dramatherapy group with mentally disordered offenders. *Dramatherapy and Destructiveness. London, Routledge* (2011).

426 Orkibi, H., Bar, N. & Eliakim, I. The effect of drama-based group therapy on aspects of mental illness stigma. *The Arts in Psychotherapy* **41**, 458-466, doi:<https://doi.org/10.1016/j.aip.2014.08.006> (2014).

427 Ramsden, E., Guarnieri, M. & Jones, P. Dramatherapy and victim empathy: A workshop approach in a forensic setting. *Drama as therapy: Clinical work and research into practice* **2**, 152-171 (2010).

428 Holloway, P. in *Dramatherapy and Destructiveness* 166-184 (Routledge, 2012).

429 Dent-Brown, K. & Wang, M. The mechanism of storymaking: a grounded theory study of the 6-part story method. *The Arts in Psychotherapy* **33**, 316-330 (2006).

430 Hillier, A., Greher, G., Poto, N. & Dougherty, M. Positive outcomes following participation in a music intervention for adolescents and young adults on the autism spectrum. *Psychology of Music* **40**, 201-215 (2012).

431 Liu, S. J., Wu, H. L. & Shi, L. The impact of music therapy on cognitive function for patients with chronic schizophrenia in recession [yin yue zhi liao dui jing shen fen lie zheng man xing shuai tui qi huan zhe ren zhi gong neng de ying xiang]. *China Rehabilitation* **28**, 230‐231. (2013).

432 Ceccato, E. *et al.* A multicentre study to test the effectiveness of the STAM (Sound Training Attention and Memory) protocol in the rehabilitation of patients with schizophrenia: a single blind, randomized control trial. *Giorn Ital Psicopat* **15**, 395-400 (2009).

433 Qu, Y. N., Huang, Y. F., Zhou, Z. X. & Li, L. H. The rehabilitation care for elderly patients with chronic schizophrenia in long‐term hospitalization [chang qi zhu yuan man xing jing shen fen lie zheng huan zhe lao nian qi de kang fu hu li]. *Guide of Chinese Medicine* **10**, 552‐554 (2012).

434 Cha, Z. Q. *et al.* The effect of receptive music therapy on cognitive function of schizophrenia patients in remission [jie shou xing yin yue zhi liao dui jing shen fen lie huan jie qi ren zhi gong neng de ying xiang]. *China Health Psychology* **20**, 1039‐1040 (2012).

435 Wen, S. R., Cao, G. Y. & Zhou, H. S. The effect of music therapy on the depressive position of patients with schizophrenia [yin yue zhi liao dui jing shen fen lie huan zhe de yi yu zhuang tai de ying xiang]. *Chinese Journal of Clinical Rehabilitation* **9**, 195 (2005).

436 He, F. R., Liu, R. K. & Ma, L. Influence of musical therapy on serum PRL of patients with schizophrenia, type II [yin yue xin li zhi liao dui xing jing shen fen lie huan zhe xue qing cui ru su shui ping de ying xiang]. *Shandong Archives of Psychiatry* **18**, 78-79 (2005).

437 Silverman, M. J. Effects of a live educational music therapy intervention on acute psychiatric inpatients' perceived social support and trust in the therapist: a four-group randomized effectiveness study. *J Music Ther* **51**, 228-249, doi:10.1093/jmt/thu011 (2014).

438 Li, Y. M., Ren, X., Li, C. P. & Li, Z. Q. The correct effect of language guided music therapy on patients with schizophrenia [yu yan you dao shi yin yue liao fa dui jing shen fen lie huan zhe de xin li jiao zhi xiao guo]. *International Nurses Journal* **26**, 917-918 (2007).

439 Fu, W. J. & Zhang, L. F. The rehabilitation effect of improvisational music therapy on the treatment of social disability in patients with schizophrenia in recovery period [ji xing yan zou shi yin yue zhi liao dui kang fu qi jing shen fen lie huan zhe she hui gong neng que sun de kang fu xiao ying]. *Sichuan Mental Health* **26**, 215‐218 (2013).

440 Talwar, N. *et al.* Music therapy for in-patients with schizophrenia: exploratory randomised controlled trial. *The british journal of psychiatry* **189**, 405-409 (2006).

441 Mohammadi, A. Z., Minhas, L. S., Haidari, M. & Panah, F. M. A study of the effects of music therapy on negative and positive symptoms in schizophrenic patients. *German Journal of Psychiatry* **15**, 56‐62 (2012).

442 Gold, C. *et al.* Individual music therapy for mental health care clients with low therapy motivation: multicentre randomised controlled trial. *Psychother Psychosom* **82**, 319-331, doi:10.1159/000348452 (2013).

443 Ulrich, G., Houtmans, T. & Gold, C. The additional therapeutic effect of group music therapy for schizophrenic patients: a randomized study. *Acta Psychiatrica Scandinavica* **116**, 362-370 (2007).

444 Lu, S. F. *et al.* Effects of group music intervention on psychiatric symptoms and depression in patient with schizophrenia. *Complement Ther Med* **21**, 682-688, doi:10.1016/j.ctim.2013.09.002 (2013).

445 Chang, C. H., Liu, F. F., Xu, T. H. & Jiao, T. L. A research of music therapy on subjective well‐being and social support for patients with schizophrenia [yin yue zhi liao dui jing shen fen lie huan zhe zhu guan xing fu gan yu she hui zhi chi de xiang guan yan jiu]. *National Medicine 2013; Vol. 25, issue 11:* **25**, 25‐27 (2013).

446 Mao, Z. Q., Li, D., Zhang, G. F., Cha, Z. Q. & Rong, J. K. The effects of music therapy on the rehabilitation of patients with chronic schizophrenia [yin yue zhi liao dui man xing jing shen fen lie zheng huan zhe de kang fu xiao guo guan cha]. *China Journal of Health Psychology* **21**, 56‐57 (2013).

447 Tang, W., Yao, X. & Zheng, Z. Rehabilitative effect of music therapy for residual schizophrenia. *The British journal of psychiatry* **165**, 38-44 (1994).

448 Wang, S. The effects of music therapy on improving social function in patients with chronic schizophrenia [man xing jing shen fen lie huan zhe fu yi yin yue zhi liao dui qi she hui gong neng de gai shan]. *The National Medicine* **25**, 51‐52 (2013).

449 Yang, W.-Y., Li, Z., Weng, Y.-Z. & Zhang, H.-Y. Psychosocial rehabilitation effects of music therapy in chronic schizophrenia. *Hong Kong Journal of Psychiatry* **8**, 38 (1998).

450 Ceccato, E., Caneva, P. & Lamonaca, D. Music Therapy and Cognitive Rehabilitation in Schizophrenic Patients: A Controlled Study. *Nordic Journal of Music Therapy* **15**, 110-120, doi:10.1080/08098130609478158 (2006).

451 Gonzalez, M. T., Hartig, T., Patil, G. G., Martinsen, E. W. & Kirkevold, M. A prospective study of group cohesiveness in therapeutic horticulture for clinical depression. *Int J Ment Health Nurs* **20**, 119-129, doi:10.1111/j.1447-0349.2010.00689.x (2011).

452 O'Brien, L., Burls, A., Townsend, M. & Ebden, M. Volunteering in nature as a way of enabling people to reintegrate into society. *Perspect Public Health* **131**, 71-81, doi:10.1177/1757913910384048 (2011).

453 Petryshen, P. M., Hawkins, J. D. & Fronchak, T. A. An evaluation of the social recreation component of a community mental health program. *Psychiatr Rehabil J* **24**, 293-298, doi:10.1037/h0095083 (2001).

454 Morrow-Howell, N., Becker-Kemppainen, S. & Judy, L. Evaluating an intervention for the elderly at increased risk of suicide. *Research on Social Work Practice* **8**, 28-46 (1998).

455 Pijnenborg, G. H. *et al.* The efficacy of SMS text messages to compensate for the effects of cognitive impairments in schizophrenia. *Br J Clin Psychol* **49**, 259-274, doi:10.1348/014466509x467828 (2010).

456 Granholm, E., Ben-Zeev, D., Link, P. C., Bradshaw, K. R. & Holden, J. L. Mobile Assessment and Treatment for Schizophrenia (MATS): A Pilot Trial of An Interactive Text-Messaging Intervention for Medication Adherence, Socialization, and Auditory Hallucinations. *Schizophrenia Bulletin* **38**, 414-425, doi:10.1093/schbul/sbr155 (2011).

457 Gracey, C. D. *Anxiety and Asperger's syndrome: an investigation into the delivery of a novel real-time stress management approach*. (The University of Manchester (United Kingdom), 2011).

458 Pitkänen, A. *et al.* Patient education methods to support quality of life and functional ability among patients with schizophrenia: a randomised clinical trial. *Qual Life Res* **21**, 247-256, doi:10.1007/s11136-011-9944-1 (2012).

459 Rotondi, A. J. *et al.* A Clinical Trial to Test the Feasibility of a Telehealth Psychoeducational Intervention for Persons With Schizophrenia and Their Families: Intervention and 3-Month Findings. *Rehabil Psychol* **50**, 325-336, doi:10.1037/0090-5550.50.4.325 (2005).

460 Boevink, W., Kroon, H., van Vugt, M., Delespaul, P. & van Os, J. A user-developed, user run recovery programme for people with severe mental illness: A randomised control trial. *Psychosis* **8**, 287-300, doi:10.1080/17522439.2016.1172335 (2016).

461 Aho‐Mustonen, K. *et al.* Group psychoeducation for long‐term offender patients with schizophrenia: An exploratory randomised controlled trial. *Criminal behaviour and mental health* **21**, 163-176 (2011).

462 Vreeland, B. *et al.* Efficacy of the team solutions program for educating patients about illness management and treatment. *Psychiatr Serv* **57**, 822-828, doi:10.1176/ps.2006.57.6.822 (2006).

463 Walker, H. *et al.* A randomised controlled trial to explore insight into psychosis; effects of a psycho-education programme on insight in a forensic population. *The Journal of Forensic Psychiatry & Psychology* **24**, 756-771 (2013).

464 Haslam, C., Cruwys, T., Haslam, S. A., Dingle, G. & Chang, M. X. Groups 4 Health: Evidence that a social-identity intervention that builds and strengthens social group membership improves mental health. *J Affect Disord* **194**, 188-195, doi:10.1016/j.jad.2016.01.010 (2016).

465 Hesselmark, E., Plenty, S. & Bejerot, S. Group cognitive behavioural therapy and group recreational activity for adults with autism spectrum disorders: A preliminary randomized controlled trial. *Autism* **18**, 672-683 (2014).

466 Atkinson, J. M., Coia, D. A., Gilmour, W. H. & Harper, J. P. The impact of education groups for people with schizophrenia on social functioning and quality of life. *Br J Psychiatry* **168**, 199-204, doi:10.1192/bjp.168.2.199 (1996).

467 Bradley, G. M. *et al.* Multiple-family group treatment for English- and Vietnamese-speaking families living with schizophrenia. *Psychiatr Serv* **57**, 521-530, doi:10.1176/ps.2006.57.4.521 (2006).

468 Ngoc, T. N., Weiss, B. & Trung, L. T. Effects of the family schizophrenia psychoeducation program for individuals with recent onset schizophrenia in Viet Nam. *Asian J Psychiatr* **22**, 162-166, doi:10.1016/j.ajp.2016.06.001 (2016).

469 Girón, M. *et al.* Efficacy and effectiveness of individual family intervention on social and clinical functioning and family burden in severe schizophrenia: a 2-year randomized controlled study. *Psychol Med* **40**, 73-84, doi:10.1017/s0033291709006126 (2010).

470 Kiep, M., Spek, A. A. & Hoeben, L. Mindfulness-based therapy in adults with an autism spectrum disorder: Do treatment effects last? *Mindfulness* **6**, 637-644 (2015).

471 Spek, A. A., van Ham, N. C. & Nyklíček, I. Mindfulness-based therapy in adults with an autism spectrum disorder: a randomized controlled trial. *Res Dev Disabil* **34**, 246-253, doi:10.1016/j.ridd.2012.08.009 (2013).

472 Mendelson, T., Leis, J. A., Perry, D. F., Stuart, E. A. & Tandon, S. D. Impact of a preventive intervention for perinatal depression on mood regulation, social support, and coping. *Arch Womens Ment Health* **16**, 211-218, doi:10.1007/s00737-013-0332-4 (2013).

473 Ekman, E. & Hiltunen, A. J. Modified CBT using visualization for Autism Spectrum Disorder (ASD), anxiety and avoidance behavior--a quasi-experimental open pilot study. *Scand J Psychol* **56**, 641-648, doi:10.1111/sjop.12255 (2015).

474 Sorenson, D. S. Healing traumatizing provider interactions among women through short-term group therapy. *Arch Psychiatr Nurs* **17**, 259-269, doi:10.1053/j.apnu.2003.10.002 (2003).

475 Sizoo, B. B. & Kuiper, E. Cognitive behavioural therapy and mindfulness based stress reduction may be equally effective in reducing anxiety and depression in adults with autism spectrum disorders. *Res Dev Disabil* **64**, 47-55, doi:10.1016/j.ridd.2017.03.004 (2017).

476 Spain, D., Blainey, S. H. & Vaillancourt, K. Group cognitive behaviour therapy (CBT) for social interaction anxiety in adults with autism spectrum disorders (ASD). *Research in Autism Spectrum Disorders* **41-42**, 20-30, doi:<https://doi.org/10.1016/j.rasd.2017.07.005> (2017).

477 McGillivray, J. A. & Evert, H. T. Group cognitive behavioural therapy program shows potential in reducing symptoms of depression and stress among young people with ASD. *J Autism Dev Disord* **44**, 2041-2051, doi:10.1007/s10803-014-2087-9 (2014).

478 Veltro, F. *et al.* A comparison of the effectiveness of problem solving training and of Cognitive-Emotional Rehabilitation on neurocognition, social cognition and social functioning in people with schizophrenia. *Clinical Practice and Epidemiology in Mental Health* **7**, 123-132, doi:10.2174/1745017901107010123 (2011).

479 Interian, A. *et al.* Randomized controlled trial of a brief Internet-based intervention for families of Veterans with posttraumatic stress disorder. *J Rehabil Res Dev* **53**, 629-640, doi:10.1682/jrrd.2014.10.0257 (2016).

480 Van der Gaag, M., Kern, R. S., Van den Bosch, R. J. & Liberman, R. P. A controlled trial of cognitive remediation in schizophrenia. *Schizophrenia Bulletin* **28**, 167-176, doi:10.1093/oxfordjournals.schbul.a006919 (2002).

481 Peyroux, E. & Franck, N. Improving Social Cognition in People with Schizophrenia with RC2S: Two Single-Case Studies. *Frontiers in Psychiatry* **7**, doi:10.3389/fpsyt.2016.00066 (2016).

482 Eack, S. M. *et al.* Cognitive enhancement therapy for early-course schizophrenia: Effects of a two-year randomized controlled trial. *Psychiatric Services* **60**, 1468-1476, doi:10.1176/ps.2009.60.11.1468 (2009).

483 Eack, S. M. *et al.* Cognitive enhancement therapy for adults with autism spectrum disorder: results of an 18-month feasibility study. *Journal of autism and developmental disorders* **43**, 2866-2877 (2013).

484 Eack, S. M. *et al.* Cognitive enhancement therapy in substance misusing schizophrenia: Results of an 18-month feasibility trial. *Schizophrenia Research* **161**, 478-483, doi:10.1016/j.schres.2014.11.017 (2015).

485 Eack, S. M. *et al.* Cognitive enhancement therapy for adult autism spectrum disorder: Results of an 18-month randomized clinical trial. *Autism Res* **11**, 519-530, doi:10.1002/aur.1913 (2018).

486 Choi, K. H. & Kwon, J. H. Social cognition enhancement training for schizophrenia: A preliminary randomized controlled trial. *Community Mental Health Journal* **42**, 177-187, doi:10.1007/s10597-005-9023-6 (2006).

487 Roncone, R. *et al.* Rehabilitation of theory of mind deficit in schizophrenia: A pilot study of metacognitive strategies in group treatment. *Neuropsychological Rehabilitation* **14**, 421-435, doi:10.1080/09602010343000291 (2004).

488 Gawrysiak, M., Nicholas, C. & Hopko, D. R. Behavioral activation for moderately depressed university students: Randomized controlled trial. *Journal of Counseling Psychology* **56**, 468-475, doi:10.1037/a0016383 (2009).

489 O'Mahen, H. A. *et al.* Netmums: a phase II randomized controlled trial of a guided Internet behavioural activation treatment for postpartum depression. *Psychol Med* **44**, 1675-1689, doi:10.1017/s0033291713002092 (2014).

490 Zang, Y., Hunt, N. & Cox, T. A randomised controlled pilot study: the effectiveness of narrative exposure therapy with adult survivors of the Sichuan earthquake. *BMC Psychiatry* **13**, 41, doi:10.1186/1471-244x-13-41 (2013).

491 Zang, Y., Hunt, N. & Cox, T. Adapting narrative exposure therapy for Chinese earthquake survivors: a pilot randomised controlled feasibility study. *BMC Psychiatry* **14**, 262, doi:10.1186/s12888-014-0262-3 (2014).

492 Cao, H. *et al.* A comparative study of new morita therapy and rehabilitation therapy on schizophrenia rehabilitation. *China J. Health Psychol.*, 1058-1060 (2008).

493 Cao, H. j., Wang, S. h. & You, H. f. Comparative study of new Morita therapy and rehabilitation therapy on recovery of schizophrenia. *Modern Journal of Integrated Chinese Traditional and Western Medicine* **12**, 2270-2272 (2003).

494 Huang, Y. Clinical observation on 41 cases of negative symptoms of schizophrenia treated by Morita therapy combined with low dose olanzapine. *Chinese J. Ethnomed. Ethnopharm.* **25**, 85-87 (2016).

495 Lv, J. J., Bai, Y. G., Huang, S. H., Ye, C. Q. & Zhou, T. T. Long-term effects of modified Morita therapy on chronic schizophrenia. *Journal Of Clinical Psychiatry* **20**, 238-239 (2010).

496 Sheng, J. *et al.* The efficacy of modified morita therapy for chronic schizophrenia patients with residual symptoms. *Chinese J. Rehabil. Med.* **21**, 834-839 (2006).

497 Shi, B. *et al.* Study on the rehabilitation effect of Morita therapy social adaptation training on community schizophrenia. *Med. J. Chinese People’s Health, Beijing China* (2013).

498 Tang, W. & Wang, Z. Observation on the effect of improved Morita therapy on the rehabilitation of schizophrenia.". *Shanghai Arch. Psychiatry* **14**, 88-90 (2002).

499 Tian, C. The effect of Morita therapy on the treatment of chronic schizophrenia. *Contemp. Med. Forum* **16**, 207-209 (2018).

500 Tian, Z., Li, X., Sun, L. & Han, L. Effect of Modified Morita therapy on schizophrenia. *China J. Health Psychol.* **22**, 1877-1878 (2014).

501 Wang, X. Application of Morita Therapy in chronic schizophrenic. *Health Psychology* **1**, 47-49 (1994).

502 Zhao, H. Rehabilitation effect of Modified Morita therapy on chronic schizophrenia in community. *China J. Health Psychol.* **27**, 1446-1451 (2019).

503 Frank, E. *et al.* Inducing lifestyle regularity in recovering bipolar disorder patients: results from the maintenance therapies in bipolar disorder protocol. *Biological Psychiatry* **41**, 1165-1173 (1997).

504 Frank, E. *et al.* Two-year outcomes for interpersonal and social rhythm therapy in individuals with bipolar I disorder. *Archives of general psychiatry* **62**, 996-1004 (2005).

505 Frank, E. *et al.* The role of interpersonal and social rhythm therapy in improving occupational functioning in patients with bipolar I disorder. *American Journal of Psychiatry* **165**, 1559-1565 (2008).

506 García, S., Fuentes, I., Ruíz, J. C., Gallach, E. & Roder, V. Application of the IPT in a Spanish sample: Evaluation of the. (2003).

507 Koekkoek, B. *et al.* Interpersonal Community Psychiatric Treatment for non-psychotic chronic patients and nurses in outpatient mental health care: A controlled pilot study on feasibility and effects. *International Journal of Nursing Studies* **49**, 549-559, doi:10.1016/j.ijnurstu.2011.11.003 (2012).

508 Campbell, J., Gilmore, L. & Cuskelly, M. Changing student teachers' attitudes towards disability and inclusion. *Journal of Intellectual and Developmental Disability* **28**, 369-379, doi:10.1080/13668250310001616407 (2003).

509 MacDonald, J. D. & MacIntyre, P. D. A rose is a rose: Effects of label change, education, and sex on attitudes toward mental disabilities. *Journal of Developmental Disabilities* **6**, 15-31 (1999).

510 Rae, H., McKenzie, K. & Murray, G. The impact of training on teacher knowledge about children with an intellectual disability. *Journal of Intellectual Disabilities* **15**, 21-30, doi:10.1177/1744629511401168 (2011).

511 Wong, P. K. S. & Wong, D. F. K. Enhancing staff attitudes, knowledge and skills in supporting the self-determination of adults with intellectual disability in residential settings in Hong Kong: A pretest-posttest comparison group design. *Journal of Intellectual Disability Research* **52**, 230-243, doi:10.1111/j.1365-2788.2007.01014.x (2008).

512 Bailey, A., Barr, O. & Bunting, B. Police attitudes toward people with intellectual disability: An evaluation of awareness training. *Journal of Intellectual Disability Research* **45**, 344-350, doi:10.1046/j.1365-2788.2001.00339.x (2001).

513 Melville, C. A. *et al.* The outcomes of an intervention study to reduce the barriers experienced by people with intellectual disabilities accessing primary health care services. *Journal of Intellectual Disability Research* **50**, 11-17, doi:10.1111/j.1365-2788.2005.00719.x (2006).

514 Tracy, J. & Iacono, T. People with developmental disabilities teaching medical students - Does it make a difference? *Journal of Intellectual and Developmental Disability* **33**, 345-348, doi:10.1080/13668250802478633 (2008).

515 Sharma, N., Lalinde, P. S. & Brosco, J. P. What do residents learn by meeting with families of children with disabilities? A qualitative analysis of an experiential learning module. *Pediatric Rehabilitation*, 185-189, doi:10.1080/13638490600570606 (2006).

516 Hall, I. & Hollins, S. Changing medical students' attitudes to learning disability. *Psychiatric Bulletin* **20**, 429-430, doi:10.1192/pb.20.7.429 (1996).

517 Hall, H. & Minnes, P. Attitudes Toward Persons with Down Syndrome: The Impact of Television. *Journal of Developmental and Physical Disabilities* **11**, 61-76, doi:10.1023/A:1021812702337 (1999).

518 Kobe, F. H. & Mulick, J. A. Attitudes toward mental retardation and eugenics: The role of formal education and experience. *Journal of Developmental and Physical Disabilities* **7**, 1-9, doi:10.1007/BF02578710 (1995).

519 Nosse, L. J. & Gavin, K. J. Influence of direct contact on college students' attitude toward adults with mental handicaps. *College Student Journal* **25**, 201-206 (1991).

520 Rimmerman, A., Hozmi, B. & Duvdevany, I. Contact and attitudes toward individuals with disabilities among students tutoring children with developmental disabilities. *Journal of Intellectual and Developmental Disability* **25**, 13-18, doi:10.1080/132697800112758 (2000).

521 Iacono, T. *et al.* DVD-based stories of people with developmental disabilities as resources for inter-professional education. *Disability and Rehabilitation* **33**, 1010-1021, doi:10.3109/09638288.2010.520802 (2011).

522 Adler, P., Cregg, M., Duignan, A., Ilett, G. & Woodhouse, J. M. Effect of training on attitudes and expertise of optometrists towards people with intellectual disabilities. *Ophthalmic and Physiological Optics* **25**, 105-118, doi:10.1111/j.1475-1313.2004.00253.x (2005).

523 Freudenthal, J. J., Boyd, L. D. & Tivis, R. Assessing change in health professions volunteers' perceptions after participating in special olympics healthy athlete events. *Journal of Dental Education* **74**, 970-979 (2010).

524 Li, C. & Wang, C. K. J. Effect of Exposure to Special Olympic Games on Attitudes of Volunteers towards Inclusion of People with Intellectual Disabilities. *Journal of Applied Research in Intellectual Disabilities* **26**, 515-521, doi:10.1111/jar.12053 (2013).

525 Roper, P. Changing Perceptions through Contact. *Disability, Handicap & Society* **5**, 243-255, doi:10.1080/02674649066780251 (1990).

526 Roper, P. Special Olympics volunteers' perceptions of people with mental retardation. *Education and Training in Mental Retardation*, 164-175 (1990).

527 Varughese, S. J. & Luty, J. Stigmatised attitudes towards intellectual disability: A randomised crossover trial. *Psychiatrist* **34**, 318-322, doi:10.1192/pb.bp.109.027789 (2010).

528 Varughese, S. J., Mendes, V. & Luty, J. Impact of positive images of a person with intellectual disability on attitudes: Randomised controlled trial. *Psychiatrist* **35**, 404-408, doi:10.1192/pb.bp.110.032425 (2011).

529 Walker, J. & Scior, K. Tackling stigma associated with intellectual disability among the general public: A study of two indirect contact interventions. *Research in Developmental Disabilities* **34**, 2200-2210, doi:10.1016/j.ridd.2013.03.024 (2013).
